# Non-cuttable material created through local resonance and strain rate effects

**DOI:** 10.1038/s41598-020-65976-0

**Published:** 2020-07-20

**Authors:** Stefan Szyniszewski, Rene Vogel, Florian Bittner, Ewa Jakubczyk, Miranda Anderson, Manuel Pelacci, Ajoku Chinedu, Hans-Josef Endres, Thomas Hipke

**Affiliations:** 1 Durham University, Durham, United Kingdom; 2 Fraunhofer Institute for Machine Tools and Forming Technology IWU, Chemnitz, Germany; 3 Fraunhofer Institute for Wood Research, Wilhelm-Klauditz-Institut WKI, Hannover, Germany; 4 Leibniz University Hannover, Institute of Plastics and Circular Economy IKK, Garbsen, Germany; 5 University of Surrey, Guildford, United Kingdom; 6 University of Stirling, Stirling, United Kingdom

**Keywords:** Mechanical engineering, Mechanical properties

## Abstract

We have created a new architected material, which is both highly deformable and ultra‐resistant to dynamic point loads. The bio-inspired metallic cellular structure (with an internal grid of large ceramic segments) is non-cuttable by an angle grinder and a power drill, and it has only 15% steel density. Our architecture derives its extreme hardness from the local resonance between the embedded ceramics in a flexible cellular matrix and the attacking tool, which produces high-frequency vibrations at the interface. The incomplete consolidation of the ceramic grains during the manufacturing also promoted fragmentation of the ceramic spheres into micron-size particulate matter, which provided an abrasive interface with increasing resistance at higher loading rates. The contrast between the ceramic segments and cellular material was also effective against a waterjet cutter because the convex geometry of the ceramic spheres widened the waterjet and reduced its velocity by two orders of magnitude. Shifting the design paradigm from static resistance to dynamic interactions between the material phases and the applied load could inspire novel, metamorphic materials with pre-programmed mechanisms across different length scales.

## Introduction

Nature uses hierarchical structures for protection from extreme loads. The freefall of grapefruit from 10 m does not damage the pulp^1^ because pomelo peel consists of vascular bundles and an open-pored cellular structure with the struts made of parenchymatic cells. Arapaimas fish living in the Amazon resist the attack of piranhas’ triangular teeth arrays through the hierarchical design of their scales^2^. The highly mineralized external layer of each scale has sinusoidal grooves, with a periodicity that is out-of-phase with that of piranha teeth spacing. The external interface is strengthened by an underlying hierarchical structure consisting of a cross-lamellar arrangement of collagen fibers. Biological shells also owe their hardness to hierarchical structures^3^, which evolved over hundreds of millions of years. Nacre of Abalon is made of hard aragonite tiles with an organic, flexible interlayer. Its fracture toughness is 3000 times higher than that of single aragonite crystals^4^. Gao *et al*.^5,6^ proposed a one-dimensional model to study the interplay between high volume fraction of mineral tiles and flexible protein layers. Metals, which require processing temperatures in excess of 300 °*C* are largely absent in biological structures; i.e., nature has only been able to create materials and architectures prone to transformations at earthbound environmental conditions.

Multi-scale material manufacturing technologies have benefited from recent developments such as self-propagating photopolymer waveguides to achieve unprecedented properties^7^. Pham *et al*.^8^ translated the known benefits of dislocations (local imperfections) in metals into 3D printed, polymeric lattices with unit cells at millimeter scale. Their work demonstrated that one needs to consider the system level interactions and that focusing only on a repetitive unit cell may be self-limiting. Shape-reconfigurable materials (SRMs) were developed to allow for significant morphological changes upon application of relatively small loads and the maintenance of the desired shape when the loading was removed^9^. Novel architectures were proposed to convert the energy of the incoming mechanical waves into a local resonance. Embodiments of that concept range from the atomistic scale^10^, through 3D printed structures^11^ up to infrastructure level, such as seismic metabarriers^12^. Topology optimization algorithms have produced material patterns capable of focusing, turning or dispersion of blast waves^13^. Our recent work has shown that locally self-impacting structures can excite natural frequencies of the material to interfere with the oscillatory loads and consequently minimize vibrations^14,15^.

## Results

### Metallic-ceramic hierarchical material

**Figure 1:**
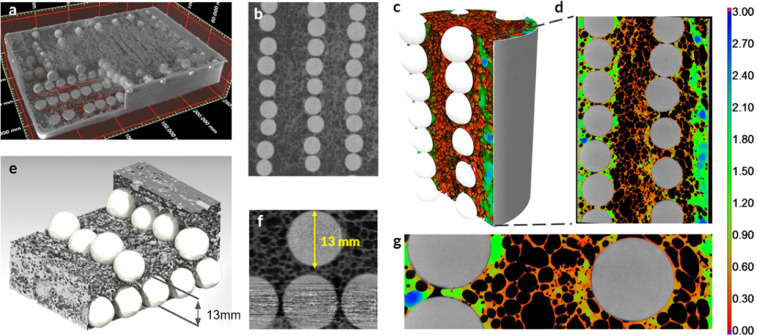
Hierarchical structure of the proposed metallic-ceramic meta-material. (**a**) Sandwich panel sample (245 mm × 172 mm × 40 mm), (**b**) ceramic spheres are organized in lines, (**c**) cylindrical specimens (60 mm diameter × 150 mm) had a vertical organization of ceramic spheres, (**d**) cross-section of the cylinder with colors corresponding to the wall thickness of cellular aluminum, (**e**) ceramic spheres are not in contact with one another but are separated by aluminum cells, (**f**) foam cells are an order of magnitude smaller than ceramic spheres, (**g**) thickness of aluminum cell walls varied mostly from 0.2 to 0.4 mm.

Our study created a new metallic-ceramic, hierarchical structure (Fig. 1), which is susceptible to internal vibrations under localized loads. These oscillations are designed to occur when a rotating cutting tool encounters a ceramic sphere on its path. The contact with the ceramic segment produces a localized load on the rim of a rotating disc, which leads to high-frequency, out-of-plane vibrations^16^. The second aspect of the resistance mechanism was inspired by the vision of Francis Bacon^17^, who wrote ‘this Proteus of Matter, being held by the Sleeves, will turn and change into many Metamorphoses.’ He believed that matter could not be destroyed or annihilated but only reshaped, reformed or transformed into a new geometry, state, or composition. Thus, we did not attempt to prevent fragmentation of the ceramic segments through the use of the hardest available material (i.e. with the highest purity). Conversely, we employed alumina spheres with suboptimal densification of the grains to promote the fragmentation into fine particulate matter because particles are useful for abrasion of the cutting disc. Notably clusters of small particles become more resistant to penetration under fast moving load due to the increase of their system level resistance under high strain rates^18^. In other words, our design did not attempt to prevent the material transformations but embraced them and selected the base materials to achieve the most favorable evolution of the constituents.

We produced sandwich plate specimens made of a cellular aluminum core (EN AW-6060) with an orthogonal layout of ceramic spheres (Fig. 1a,b,e,f) with steel alloy (DC01) faceplates. The ceramic spheres had 13 mm mean diameter, while aluminum foam had on average 2.1 mm pore size only and the wall thickness ranging from 30 µm to about 1000 µm. Thus, the ceramic inclusions were an order of magnitude larger than ceramic particulate matter or whiskers used for reinforcement of metallic foams. The ceramic spheres were sufficiently large to interact with a drill or a cutting blade and occupied only 14% volume fraction of the cellular core.

The aluminum foam matrix had a density of *ρ*_*f*_ = 730 *kg*/*m*^3^ and 73% porosity (air content), which ensured sufficient flexibility of the matrix. Our metallic-ceramic hierarchical structure had *ρ*_*f*_ = 1140 *kg*/*m*^3^ density. Sandwich panels with two 2 mm steel face plates had 1780 *kg*/*m*^3^ density in a 40 mm thick panel configuration. We also manufactured cylindrical specimens to demonstrate that our technology can achieve high aspect ratio components such as beams or columns (Fig. 1c,d,g and CT scans in Supplementary Information). The cylinders were ideal for computed tomography (CT) because the X-ray travel path through the foam was equal around the circumference, which reduced measuring artifacts. See Methods, section 7 for the description of our CT setup.

### Manufacturing of the material

**Figure 2:**
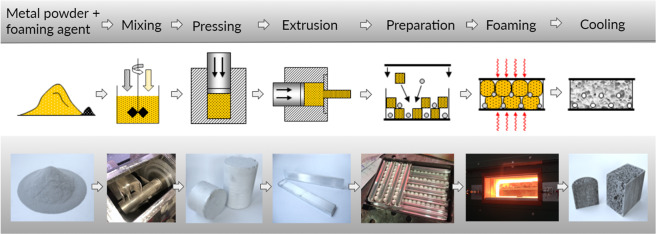
Manufacturing steps include mixing metal powder with small amount of foaming agent. It is followed by pressing the mix and extrusion into preform shapes. The compressed powder bars enable precise placement of ceramic segments in the orthogonal pattern and manufacturing of the end product in an industrial furnace.

Our material consists of metallic and ceramic ingredients and consequently requires metallurgical processes to manufacture the end parts. Firstly, the aluminum powder is mixed with titanium dihydride, TiH_2_ (foaming agent) utilizing a rotating impeller to ensure a uniform mixture (Fig. 2). After the mixing stage, the powder mixture is consolidated via cold compaction in a compressor and then extruded through an extrusion die resulting in dense rods of material, which are cut into smaller pieces. Next, ceramic spheres and compressed aluminum powder rods are stacked in an orthogonal, grillage pattern and enclosed in a steel box using spot welds. The structure is then heated in a furnace to ca. 760 °C (depending on the melting range of the used aluminum alloy) for between 15 and 20 minutes. The titanium dihydride begins to decompose at approximately 470 °C, releasing hydrogen gas. The release of the high-pressure hydrogen expands the molten aluminum, creating voids. The components are subsequently cooled in calm air to produce a stable cellular structure with embedded ceramic components.

### Mechanical properties

**Figure 3:**
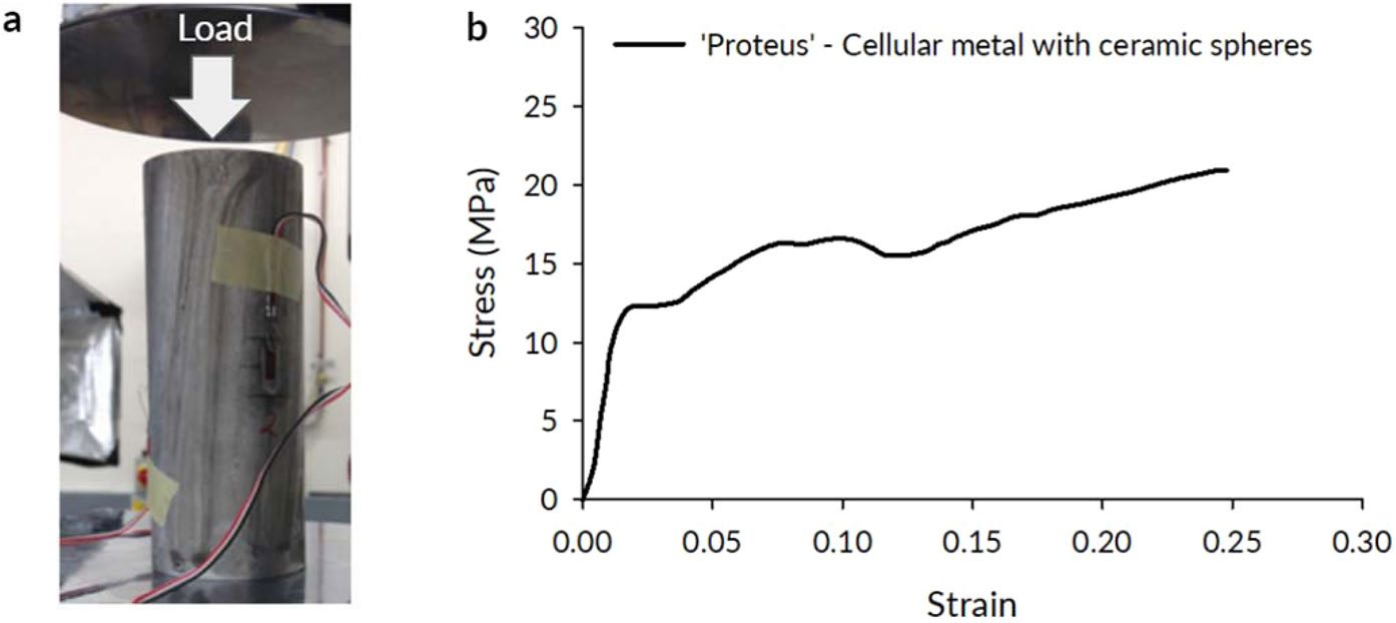
Compressive testing of cylindrical samples. (**a**) quasi-static compressive test of a cylindrical sample, (**b**) metallic foam with ceramic spheres showed distinct plateau and the ability for compressive deformation up to 25%.

We subjected cylindrical samples (60 mm diameter × 150 mm height) to compressive loading in order to characterize mechanical properties of our meta-material (Fig. 3). The Young’s modulus, from loading-unloading tests, was *E* = 5.5 *GPa* and yield stress, *σ*_*y*_ = 8.1 *MPa* (measured according to ISO/DIS 13314 standard). The Poisson’s ratio of the material was approximately 0.0 in the early stages of the loading and began ascending toward 0.5 from 10% engineering strain onward (see Methods, section 1). The cellular structure showed significant deformability, exceeding 20% of engineering strain as expected from previous studies of cellular metals^19–23^ with the densification strain of *ε*_*d*_ ≈ 0.25. The inclusion of ceramic spheres did not have a significant effect under static loading because the deformations followed the paths of the least resistance via the cellular matrix in-between the spheres.

### Resistance to angle grinder cutting

Relatively low plateau stress (compared to solid steel) and ability for large compressive deformations is excellent for energy dissipation under extreme loads. However, protective systems usually also require hardness to withstand point loads. Ballistic resistance is often achieved with ceramic materials such as high-purity alumina (*Al*_2_*O*_3_) or high-hardness, armor steels. The multiplicity of the hardness measuring instruments with various indenter shapes and test protocols^24^ indicate that hardness may not be a fundamental property of a material but rather a composite one including yield strength, work hardening, true tensile strength, modulus of elasticity, and micro properties such as strength of atomic bonds. We were not aiming in our study at low depths of Brinell, Rockwell or Knoop indentations but at resistance to penetration under real-life extreme loads. We concentrated on developing resistance to the severe localized loads such as an angle grinder, power drill, and water jet cutter, which are examples of extreme loads. Focusing on these specific, end-user features allowed us to depart from the established paradigm of designing for the high hardness, i.e. low indentation resistance.

**Figure 4:**
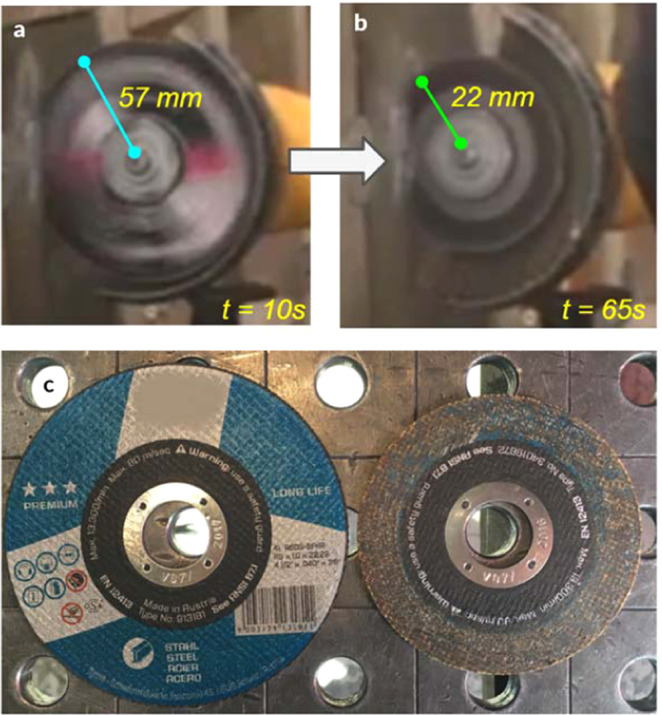
No complete penetration was achieved in any of our cutting tests. In the same time, excessive wear of the discs, (**a,b**) Marcrist and (**c**) Tyrolit occurred within one minute of the cutting attacks and rendered angle grinders ineffective.

The angle grinder attack represented the first highly localized load applied to penetrate our architected material. We used 115- and 125-mm diameter cutting discs with a sapphire finish to cut our panel samples (see Fig. 4 and Methods section 2 for disc descriptions). The angle grinder revolved at up to 180 revolutions per second, with rim velocity of up to 80 m/s. The angle grinder achieved only a partial incision and subsequently experienced high wear. Its external diameter reduced rapidly from 115 mm to 44 mm (Marcrist disc) and from 125 mm to 100 mm (Tyrolit disc) after 60–65 sec. At that point, the cutting discs became ineffective (Fig. 4b and Movie S1). Exact cutting attack times for the performed tests are tabulated in Methods, section 2. To compare our results with existing armor materials, we also tested one of the best currently available rolled-homogeneous armor (RHA) steels, distributed under MARS 220 trademark. The steel is quenched and tempered at temperatures below 200 °C to give it 440 Brinell hardness. The angle grinder completely penetrated the 10 mm MARS 220 plate in 45 sec (see Methods section 2 and Movie S2).

**Figure 5:**
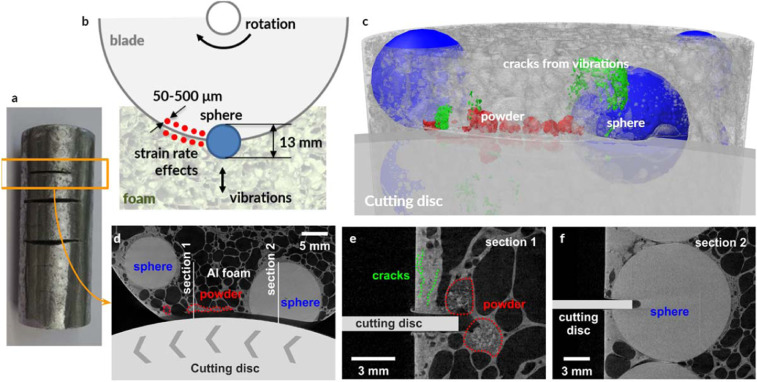
Resistance mechanism to angle grinder cutting. (**a**) cylindrical sample for CT study, (**b**) ceramic sphere applied point load to the rim of the rotating disc, inducing vibrations and dynamic interface, (**c**) CT image (color-coded) showing the interaction between the partially cut sphere and the rotating disc, (**d**) rotating disc cut the sphere partially and then the cutting ceased to progress, (**e**) low purity employed alumina ensured sphere fragmentation into particles in the range of 50–500 µm, (**f**) ceramic sphere applied localized load to the tip of the rotating disc resulting in disc vibrations.

The resistance mechanism of our architected material exploited the contrast in stiffness between the ceramic spheres and the flexible aluminum cellular structure. Thereby, the energy of the rotating angle grinder was partially transferred into the mechanical vibrations of the spinning disc (Fig. 5) and subsequently into vibrations of the ceramic sphere (nested in the flexible cellular matrix), which interacted with the cutting disc (Fig. 5b,c). The tool operator reported vibrations, and they are also evident in Movie 1. Computed tomography scans also detected cracks in the aluminum foam structure (Fig. 5c,e, Supplementary Information A and Movies 3–5). The working principle of the vibrational interface is shown schematically in Fig. 5b. Localized contact of the rotating disc with the ceramic sphere (blue) generated broadband vibrations. Such behavior is consistent with previous work by Campbell^16^, who measured multiple vibrational modes, including four nodal waves, after application of a rubbing contact to a rim of a rotating disc.

Dynamic vibrations had not been effective when the disc only touched the external surface of the ceramic sphere because the contact area on the sides of the cutting disc was insufficient to excite considerable disc oscillations. However, as the cutting progressed, it increased the contact region with the disc’s rim (Fig. 5f). Consequently, lateral disc vibrations were generated, which created a dynamic interface with the partially cut ceramic sphere. In addition to the case where the cutting discs were applied to the equator of the sphere, we also tested cases, where the angle grinder cut on the side of a sphere, in the proximity of the equator and closer to the sphere’s pole (see SI with CT scans of these cases and Movies 6–9). We observed the same outcome, i.e., the cutting disc diameter reduced rapidly in just over a minute, and the angle grinder became inoperative. Interestingly, the vibrational mechanism was equally effective against the power drill, where the geometry of the drill bits differed significantly from the flat, cutting discs of angle grinders. In all instances when a drill encountered a ceramic sphere, the drilling progress was arrested (see Movie 10). Hence, the vibrational interface mechanism did not appear sensitive to the aspect ratio of the rotating cutting tools. The experimental procedure and detailed results of cutting and drilling tests are explained in Methods, section 2. The CT-scans of these tests are included in Supplementary Information C.

**Figure 6:**
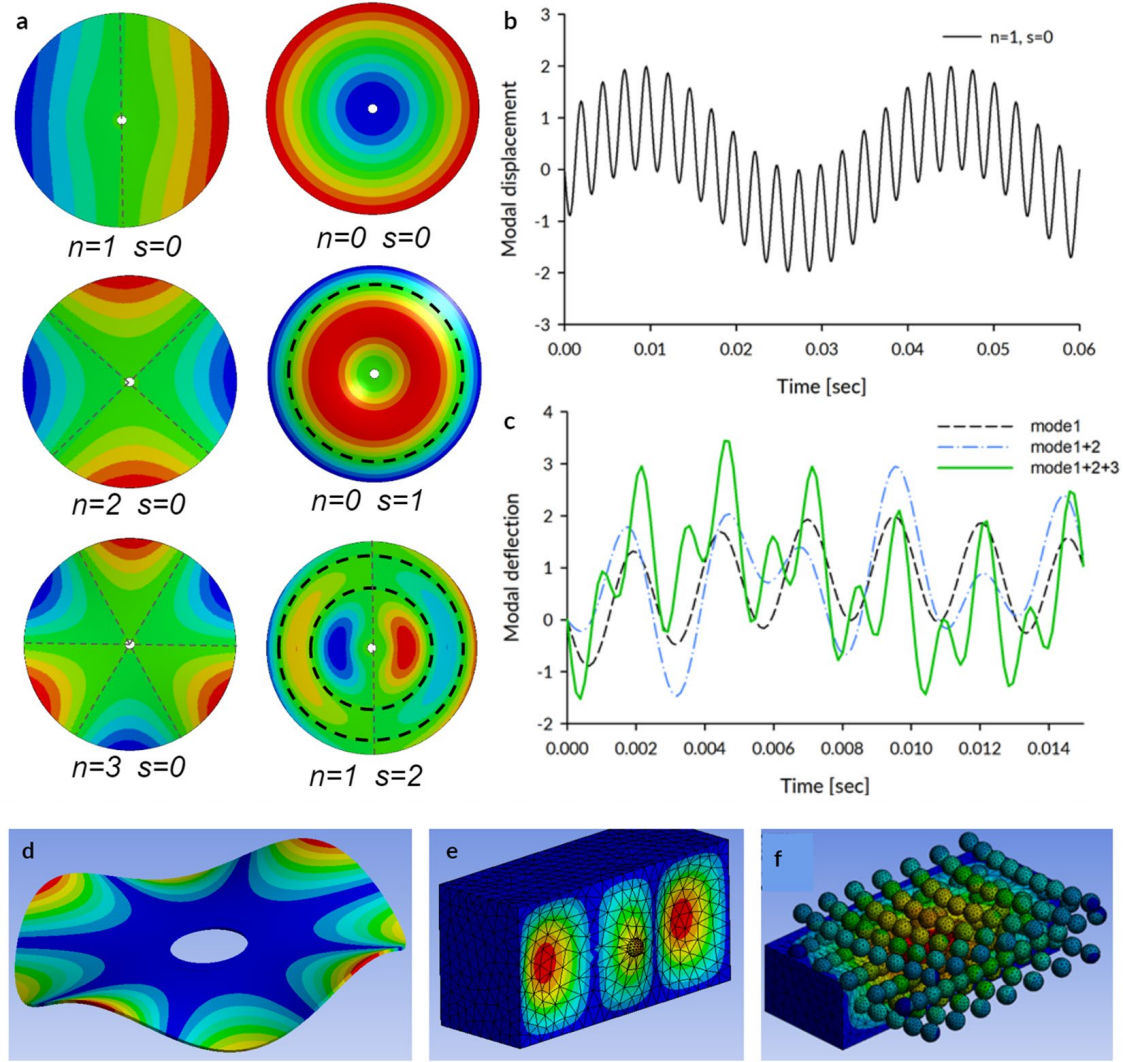
(**a**) Vibration modes consist of angular waves denoted with n = 1 for one wave, n = 2 for two waves, n = 3 for three waves, as well as radial waves propagating from the center of the disc toward the circumference, which are denoted with s = 0 (resembling deflected shape under uniform pressure, s = 1 for one axisymmetric wave). Combination of both radial and angular components appears at higher frequencies (e.g. n = 1 and s = 2). (**b**) Vibrational signal transferred to the sphere by the first set of the forward and backward travelling waves in the rotating disc is bi-modal. (**c**) Cumulative effect of the first three modes (f_1,0_ + f_0,0_ + f_2,0_) compared with the superposition of the first two modes and the first mode signal only. When multiple travelling waves are excited, the excitation becomes multi-modal and the signal increasingly irregular. (**d**) Computational vibrational mode of the actual cutting disc with a central hole (n = 4, s = 0 with ${f}_{B}=713\,Hz$ and ${f}_{F}=2175\,Hz$), (**e**) Vibrational mode of a ceramic sphere in a flexible continuum (9340 Hz), (**f**) metallic foam vibrational mode accounting for the interactions between multiple embedded spheres.

Theoretical modes of vibrations^25,26^ consist of shape variations along the angular direction, with waveform denoted by *n* (see Fig. 6). The first angular wave corresponds to a single sinusoidal wave along the circumference of the disc denoted with *n* = 1. For *n* = 2 deflections form a train of two waves, for *n* = 3 a train of three waves and so on. In addition to circumferential modes, waveforms can also propagate radially from the center of the disc. Such waves are like ripples observed after dropping a pebble into a lake. The fundamental radial mode, corresponding to *s* = 0, resembles deflection under uniform pressure (see Fig. 6a). Higher radial modes with *s* = 1, 2, *etc* form ‘sinusoidal’ waves in the radial direction.

Timoshenko^26^ expressed free vibrations of a rotating disc as a product of both radial and angular components varying in time, as:

1 $$z(r,\theta ,t)={z}_{s}(r)\cdot (\sin (n\theta )\cos ({\omega }_{ns}t)\pm \,\cos (n\theta )\sin ({\omega }_{ns}t))$$

where *ω*_*ns*_ = 2*π f*_*ns*_ is the natural frequency of vibrations corresponding to the superposition of angular, *n* and radial, *s* modes. Using trigonometric identities, equation (1) can be simplified to:

2 $$z(r,\theta ,t)={z}_{s}(r)\cdot \,\sin (n\theta \pm {\omega }_{ns}t)$$

One should note that natural frequency of the disc depends on the spinning speed, namely *ω*_*ns*_ = *f*(*ω*_*disc*_) is a function of the disc rotational velocity because spinning produces centrifugal tension in the disc, and consequently stiffens it. A careful reader might also recognize that the modal shapes are given in the disc frame of reference, which is rotating with the disc. An external observer measuring deflections of a fixed point in the contact with the rim, such as an embedded ceramic sphere, traces a point moving counterclockwise relatively to the disc spinning clockwise. The coordinate of such a point in the spinning frame of reference is *θ*_point_ (*t*) = *θ*_0_ − *ω*_*disc*_*t*, where *θ*_0_ is the initial angular position. Thus, a point, with *R*_*po*int_, *θ*_*po*int_ coordinates in the stationary frame of reference, experiences the following excitation:

3 $$\begin{array}{rcl}z({R}_{point},{\theta }_{point}(t),t) & = & {z}_{s}({R}_{point})\cdot \,\sin (n({\theta }_{0}-{\omega }_{disc}t)\pm {\omega }_{ns}t)\\  & = & {z}_{s}({R}_{point})\cdot [\sin (n({\theta }_{0}-{\omega }_{disc}t)+{\omega }_{ns}t)\\  &  & +\,\sin (n({\theta }_{0}-{\omega }_{disc}t)-{\omega }_{ns}t)]\\  & = & {z}_{s}({R}_{point})\cdot [\,\sin (({\omega }_{ns}-n{\omega }_{disc})t+n{\theta }_{0})\\  &  & -\,\sin (({\omega }_{ns}+n{\omega }_{disc})t-n{\theta }_{0})]\\  & = & {z}_{s}({R}_{point})\cdot [\sin \left(n\left[\left(\frac{{\omega }_{ns}}{n}-{\omega }_{disc}\right)t+{\theta }_{0}\right]\right)\\  &  & -\,\sin \left(n\left[\left(\frac{{\omega }_{ns}}{n}+{\omega }_{disc}\right)t+{\theta }_{0}\right]\right)]\end{array}$$

Thereby, a stationary point in space such as a ceramic sphere at the rim of the rotating disc senses two waves, namely the (B)ackward and the (F)orward travelling waves, with rotational frequencies:

4 $$\begin{array}{ccc}{\omega }_{ns\,F}^{sphere} & = & n\left(\frac{{\omega }_{ns}}{n},+,{\omega }_{disc}\right)\\ {\omega }_{ns\,B}^{sphere} & = & n\left(\frac{{\omega }_{ns}}{n},-,{\omega }_{disc}\right)\end{array}$$

where $\frac{{\omega }_{ns}}{n}$ is the speed of the forward and backward moving wave in the disc frame of reference. Although both waves have the same speed, they move in the opposite directions, and their overlap produces a standing wave in the disc frame of reference. Whereas *ω*_*disc*_ is the spinning velocity of the disc, and *n* is a number of angular waves in the fundamental modes as shown in Fig. 6a. The number of the angular waveforms, n increases the frequency of the wave contact with the stationary ceramic sphere. This result is consistent with independent derivation by Campbell^16^ (see Eqs. 6 and 7 of the  seperate Campbell derivations, based on the requirements for standing waves, attached in Supplementary Information B).

Number of waves, n has an effect on the excitation frequency experienced by a ceramic sphere. For example, two waves, n = 2 in a train of waves increase the excitation frequency applied to the ceramic sphere by the factor of two. It is useful to note that (4) can also be written in terms of frequencies in cycles per second [Hz], and for the (F)orward travelling wave it is:

5 $${f}_{n{s}_{F}}^{sphere}=n\left(\frac{{f}_{ns}}{n}+{f}_{disc}\right)$$

Similarly, the (B)ackward travelling wave, which travels against the direction of the disc rotation, has a frequency of:

6 $${f}_{n{s}_{B}}^{sphere}=n\left(\frac{{f}_{ns}}{n}-{f}_{disc}\right)$$

The analytical time history produced by the first mode of forward and backward moving disc waves is shown in Fig. 6b (see Methods, section 3 for the numerical calculations). Campbell^16^ measured disc vibrations with both stationary electromagnetic sensors and the probes rotating with the disc. His stationary sensors recorded the forward (higher frequency) and backwards (lower frequency) waves travelling in the opposite directions. The oscillograph record shown in Fig. 33 of Campbell’s article (in Supplementary Information B) resembles Fig. 6b derived from the analytical considerations shown here. While inspecting Campbell’s results, it is necessary to keep in mind, however, that in an oscillograph record the amplitude is dependent on the induced voltage, which, in turn, depends on both the amplitude and the frequency of the vibration. Thus, higher frequencies have magnified amplitudes in proportion to the frequency. Campbell’s experimental work also demonstrated that a prolonged resonance with the backward travelling wave led to disc failure. These observations established that disc vibrations can be destructive. Irregular contact of the cutting disc with the sphere, due to manual operation of the angle grinder, compounded by potential interactions between the forward travelling waves and higher natural modes of the disc, may excite a couple of the natural frequencies at the same time. Multiple natural modes are usually observed during impact tests^27^. Cumulative excitation produced by the three lowest modes (see Methods, section 3 for the values of the natural frequencies) form a more complicated signal (shown with bold green color in Fig. 6c).

On the one hand, the analytical natural frequencies for the idealized disc (without any central hole) range from 231 Hz to 3341 Hz for the first eight modes (see Methods, section 3). The frequencies with circumferential waveform(s), i.e. with $n\ge 1$, generate forward and backward travelling waves. Numerical natural frequencies for a composite disc corresponding to dimensions and material properties of 125 mm Tyrolit disc were also computed using ANSYS software (see Methods, section 4). These models include a central opening for fixing the disc to the shaft of the angle grinder. The first 6 natural frequencies produced backward and forward travelling waves in the range from 246 Hz to 2991 Hz (see Fig. 6d). On the other hand, analysis of natural frequencies of the sphere embedded in a continuum with mechanical properties corresponding to our metallic foam resulted in the range of natural frequencies from 5300 Hz to 9300 Hz (Fig. 6e).

Thus, one can expect the vibrational interaction between higher disc frequencies (with n > 5) and local oscillations of the ceramic sphere, which is embedded in a flexible, cellular matrix. These vibrations can be local, or if enough energy is injected, they can encompass more spheres (Fig. 6f). Interestingly, fixing a point on a rim during an eigenanalysis (to approximate the effect of the locally applied load) results in more irregular eigenmodes, as shown in Movie 11. Since the disc is rotating, the contact point is moving across the circumference of the disc (as noted above). Such modal analysis cannot be performed using an eigensolver because the boundary conditions are changing with time. It would require a transient, time history analysis with post-processing of the time signals to identify the natural frequencies and their corresponding waveforms, which is beyond the scope of this article. Irrespectively of the modelling nuances, it is broadly clear that forward and backward moving components of the higher natural disc frequencies will interact with the embedded spheres. Such higher order waves are consistent with the gradual grinding of the discs as opposed to their catastrophic failure into a few, large pieces, which would be more consistent with lower vibrational modes.

**Figure 7:**
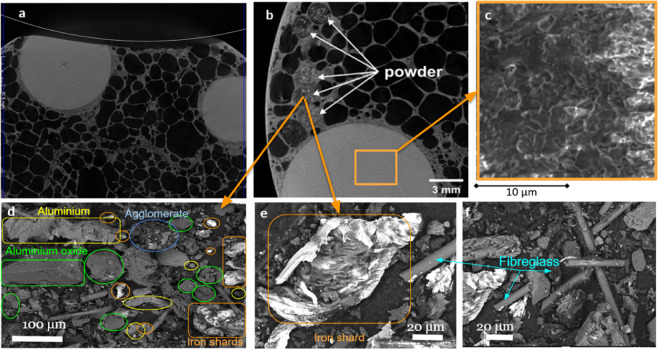
(**a,b**) Computed tomography scans of the cross sectional area of the cylindrical sample; (**c**) SEM image of ceramic spheres with the surface morphology consistent with low densification ceramic structure; (**d–f**) SEM images of the particulate, which included aluminum oxide, aluminum, iron shards, and the fractional content of the blade composition such as fiberglass fragments, titanium dioxide, calcium oxide carbonate, magnesium oxide, graphite as well as fluorides.

The interface between the disc and the ceramic spheres was not only oscillating but also very abrasive due to the fragmentation of the alumina spheres into particulate matter (see Figs. 5c–e, 7). The ceramic spheres exhibited a very rough surface (Fig. 7c), consistent with a low densification morphology, which promoted the fragmentation into particulate matter shown in Fig. 7d–f. The particles created a sandpaper-like, vibrating interface with the cutting disc. We extracted the powder following the cutting tests for further inspection as explained in Methods, section 6 and Supplementary information C. Scanning electron microscopy (SEM) images revealed a variety of particles with a myriad of compositions and shapes (Fig. 7d–f). The size distribution of the particulate matter (50–500 µm) from both the cutting disc (such as fiberglass fragments or iron shards) and ceramic sphere (aluminum oxide) confirmed the abrasive wear of the cutting disc, rather than a fragmentation caused by dynamic cracking of the disc into large fragments.

**Figure 8:**
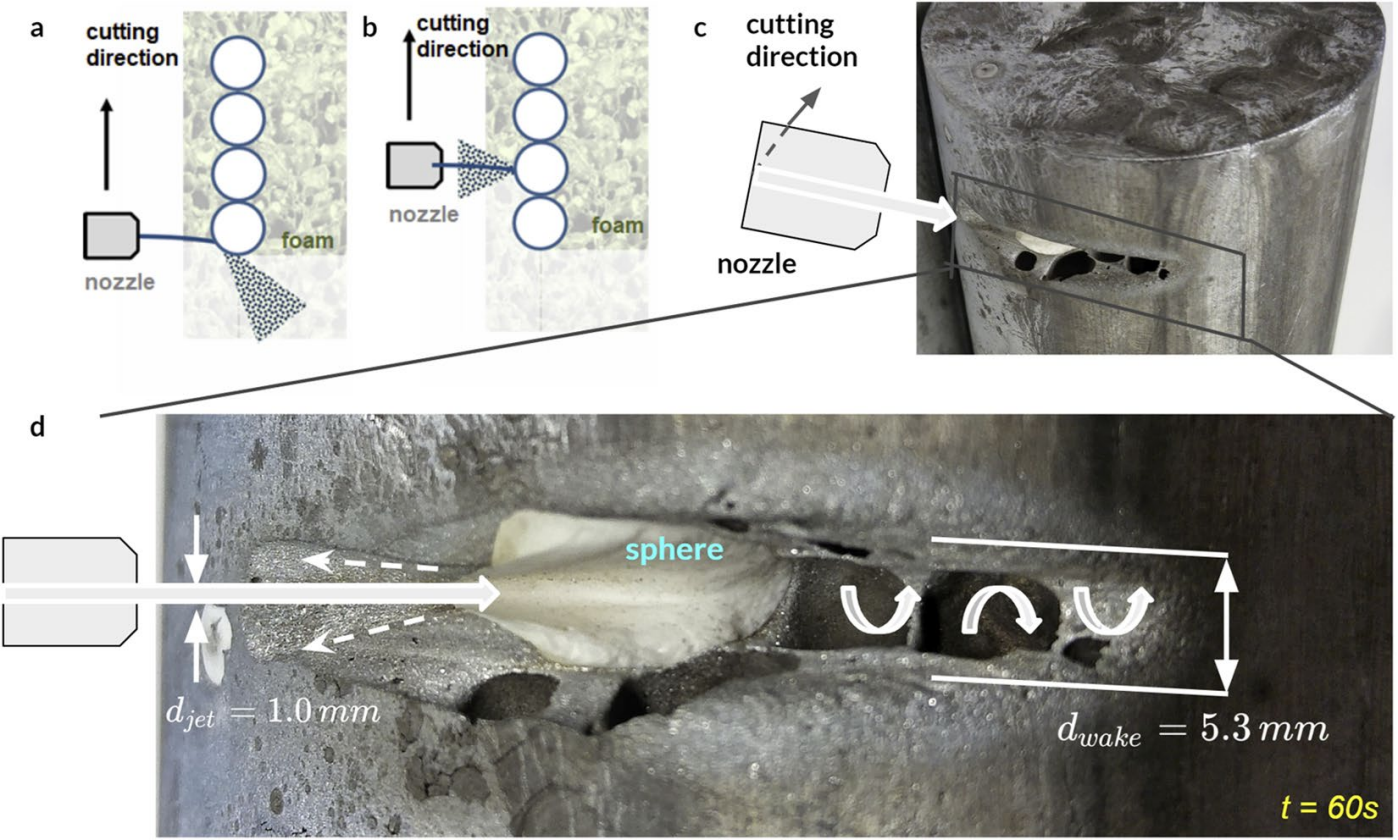
Influence of nested ceramic spheres on the hydrodynamics of impacting water jet. (**a**) jet widening upon contact with the convex surface of the sphere, (**b**) fast cutting resulting in superficial penetration and jet reflection, (**c**) test performed on a cylinder to examine the erosion of the sphere under abrasive jet action, (**d**) abraded geometry indicated a significant widening of the jet. Note that the transverse curvature of the interface with the jet was maintained throughout the cutting process.

The contrast between the stiffness of the ceramics and cellular metallic foam matrix as well as the susceptibility of the ceramic spheres to fragmentation were also effective against the waterjet cutter. The abrasive jet cut through the metallic foam but was deflected from the first encountered ceramic sphere (Fig. 8a,b) and accompanied by reverse splashing toward the nozzle. We also conducted tests on a cylindrical sample to enable examination of the sphere erosion (Fig. 8c,d). The curvature of the ceramic obstacle was maintained as the sphere was eroding and it was responsible for widening the jet, which slowed its velocity by two orders of magnitude.

Scrutiny of the final eroded geometry revealed that the jet diameter increased from *d*_*jet*_ = 1.0 *mm* to *d*_*wake*_ = 5.3 *mm* when exiting the material sample (Fig. 8d). Considering mass flow rate as constant, *m* = *const*, the velocity in the wake, *V*_*wake*_ can be computed from:

7 $$\rho {A}_{jet}{V}_{jet}=\rho {A}_{wake}{V}_{wake}$$

where the *V*_*jet*_ is the initial, optimal cutting velocity of the jet and it is given by the manufacturer, while cross-sectional areas *A*_*jet*_ and *A*_*wake*_ are measured following a series of cutting tests.

If one assumes that the density of the jet is constant (despite the debris), the wake velocity is:

8 $${V}_{wake}=\frac{{A}_{wake}}{{A}_{jet}}{V}_{jet}=\frac{{d}_{wake}^{2}}{{d}_{jet}^{2}}={V}_{jet}\approx 2.3 \% {V}_{jet}$$

Thus, the cutting speed in the wake dropped off 50 times from the jet velocity of 821 *m*/*s* at the nozzle to 19 *m*/*s* inside our architected material. The cutting progress slowed considerably due to the limited abrasive effectiveness of the much slower jet. The velocity, *V*_*wake*_ appeared to be below the limit of active, fast abrasion. It was closer to the regime of slow erosion approaching geological time scales observed in riverbeds and rocks.

We used cut area per unit time to quantify the resistance to waterjet cutting experimentally. The spherical segment (shown in Fig. 8c,d, with 15 mm depth) was cut over 60 *s*, which corresponded to a cutting rate of *rate*_*hierarch*_ = 5.5 *cm*^2^/*min*. We compared that cutting rate with a stainless steel plate with 20 *mm* thickness and areal density, *ρ*_*steel*_ = 157 *kg*/*m*^2^, which was more than double that of our 40 *mm* metallic foam ceramic structure with *ρ*_*hierarch*_ *= 71 kg*/*m*^2^. In spite of the higher areal density, the water jet cutting rate of the solid steel plate was two orders of magnitudes faster than that of our metallic foam ceramic sphere architecture at *rate*_*steel*_ *= 17*0 *cm*^2^/*min*. Considering the waterjet cutter effectively stopped at the 15 mm incision, our architected material can be considered non-cuttable by a waterjet cutter.

## Discussion

Our experimental observations are consistent with previous studies of waterjets impacting convex obstacles by Wilson and Goldstein^28^ and later Fujisawa and Kobayashi^29^. The interaction of the mean flow and the turbulence strengthened as a function of *d*_*jet*_/(2*R*), where *d*_*jet*_ was the diameter of the jet, and *R* was the radius of the circular obstacle, but more importantly the measure of its curvature^29^. The ratio of our jet diameter to the sphere radius was approximately 0.15 and corresponded to case No 2 in their experimental work on cylindrical obstacles. Fujisawa and Kobayashi reported that upon contact with the curved surface the jet diameter increased largely. We also noticed the waterjet widening as a result of the contact with a strongly convex ceramic sphere in our tests. The waterjet resembled an inverted funnel-shape, which abraded the sphere such that the cut geometry maintained the convex curvature in the wake region throughout the cutting (Fig. 8d). That convex curvature in the lee side of the eroded sphere continued to widen the jet during the cutting due to a stable, funnel shape cut plane.

The angle grinder and power drill were also defeated, even though ceramic spheres were partially cut before the cutting attacks were stopped. Thus, we created a resistance mechanism, which is not based on the hardness of the ceramic segments but on vibrations induced in the cutting discs, which have a complex nature and consist of both forward and backward travelling waves, across a broad range of frequencies. Considering the vibrations from the perspective of a ceramic sphere (Fig. 5f), both radial and traveling waves impact the sphere, whenever they pass through the cut. Thus, several natural frequencies of the disc might interact with the metallic foam ceramic material. We expect higher natural frequencies of the disc to interact with the fundamental, natural frequency of the sphere nested in the cellular metal. However, it might be also possible for several frequencies to interact simultaneously as the point load from the sphere is moving around the disc with a noticeable rotational velocity.

Varying the cutting disc diameter, its thickness, and the rotational speed of the disc are expected to produce a range of vibrational responses. At the same time, we have experimentally shown efficacy against cutting discs of various diameters (115 mm and 125 mm) as well as against power drills, which might indicate that the dynamic resistance mechanism is not sensitive to variations in disc geometries. In other words, application of a point load to a rim of a rotating disc always induces vibrations in the disc, irrespectively of its diameter and material. Once travelling waves are present in the disc, they interact with a ceramic sphere in the metallic foam leading to the gradual destruction of the cutting disc. The multitude of waves traveling with various radial and angular frequencies overlay or diverge in time, depending on their relative phase lags and the movement of the point load on the rim. Thus, an irregular wave with varying amplitude is expected, which might help to explain sudden jolts experienced by an angle grinder operator, which occurred at apparently aberrant intervals during the cutting tests (as seen in the Movie 1). Thus, the contact between the disc and the ceramic sphere might get tighter or looser, and considering the variability of the applied pressure by a human operator, varying inclination angle and other real-life random imperfections, the contact forces and vibration waves are expected to contain significant, non-vanishing transient components and exhibit a stochastic nature.

Our resistance mechanism was also likely benefiting from the strain rate effects of granular matter, which are known to increase the fracture toughness of particulate matter exponentially. The tensile strength of sand grains has been shown to double already at 15 m/s loading velocity^18^. Sandbags that are encountered in conflict zones are effective in stopping bullets mainly due to the strain rate enhancement. Thus, we expect the contact stresses between the vibrating disc and the particles in the cut to increase largely with the rotational velocity of the cutting blade and the frequencies of the disc vibrations because faster loading rates increase strain rate related fracture toughness of the particulate matter.

Significantly, the eroded geometry of a ceramic sphere under the waterjet abrasion was different from the partially cut sphere with an angle grinder, which was accompanied by particulate matter. Thus, our material ‘metamorphosed’ differently under different cutting loads to render them ineffective in all cases. Additionally, local changes in the microstructure of the ceramic sphere material resulting from localized heating (due to friction with the cutting blade) could enable a phase change and densification of alumina under certain conditions. Such material hardening on the surface of the spheres is likely to further enhance the cutting resistance of our architected material and should be investigated in future studies.

In summary, we created a hierarchical architected material by combining hard ceramic spheres and highly compressible aluminum foam matrix and called it ‘Proteus’ due to its metamorphic nature. Ceramic inclusions had no detrimental effect on compressibility under quasi-static load. However, their spatial hierarchy had significant ramifications on performance under dynamic load. In the case of an angle grinder attack, vibrations of the disc, manifested as contact pressure oscillations, coupled with the abrasive action of ceramic particles resulted in extraordinary cutting resistance, consistent with a non-cuttable material. The waterjet cutter was rendered equally ineffective by the curvature of the spheres in the lee side region, which widened the jet cross-section and consequently reduced the jet velocity fifty times according to our estimate.

The configuration of our new architected material can be tailored to a broad range of applications. One can tune foam porosity, alter base materials, adjust the size of spheres, and their packing pattern. We can also modify the thickness of the sandwich panels, the diameter of the cylinders, the thickness of faceplates and manufacture curvilinear geometries effectively. The demonstrated components can be welded and bonded with other materials to form larger structural systems. Security applications such as doors or barriers (as protection from forcible entry attacks) are obvious ones. However, our material technology could also be useful for enhancing the cutting resistance of shoe soles or protective clothing. Workers could benefit from non-cuttable elbow pads or forearm guards in environments with industrial tools. In addition to vibrational inputs, wave propagation via multiple media and chemical transformations at the metallic-ceramic interfaces require further investigation.

## Methods

### Section 1. mechanical testing of material properties

**Figure 9:**
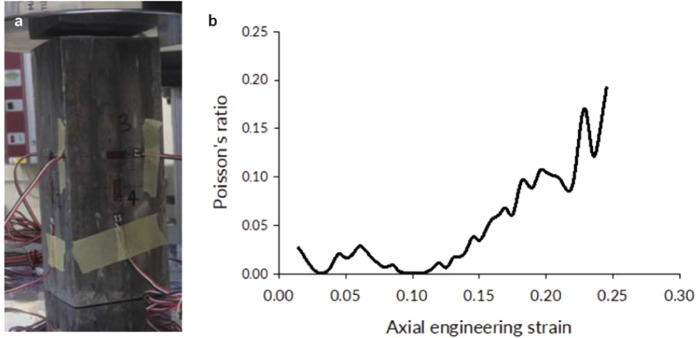
Poisson ratio of Proteus, cellular metal with ceramic spheres, remained close to zero initially, which indicated a high degree of material compressibility. Incompressible fluids or steel under loading exceeding its yield stress have a Poisson ratio of 0.5. At large deformations, the Poisson ratio of our cellular metallic architecture began increasing as the material densified and was becoming less compressible.

We manufactured six cylindrical compression specimens with 150 mm height and 60 mm diameter. From these, three aluminum foam baseline specimens and three samples with ceramic spheres embedded in the foam were compressed axially. Three strain gauges spaced equally around the circumference of each of these specimens (120 degrees apart) and placed at mid-height measured the strains. Multiple loading-unloading cycles gave information on Young’s modulus. The same cylindrical samples that were used in the determination of the elastic modulus were loaded to failure to determine the stress-strain response of the material. The data logged from the testing included the compressive force, the readings from each of the applied strain gauges and the crosshead displacement of the testing machine. Yield stress values were determined from the procedure described in ISO/DIS 13314 standard titled “Mechanical testing of materials – Ductility testing – Compression test for porous and cellular materials.” The testing demonstrated that the inclusion of the ceramic spheres did not have a significant effect on the mechanical properties of the foam composite as compared to pure aluminum foam. The loads followed the path of least resistance via the foam matrix, which controlled the compliance. Evolution of material compressibility as manifested by its Poisson ratio is shown in Fig. 9.

### Section 2. angle grinder cutting and power drill tests

We tested the resistance of the panels against an angle grinder and a power drill. Three timed attacks were performed with an angle grinder for a duration of one minute. After the time limit had been achieved, the damage was assessed in each case. If complete penetration of the material was seen then the time at which this occurred was noted, in other cases the depth of the final penetration was measured. The test results of the angle grinder attacks are given in Table 1 for our material.

**Table 1:** Angle grinder attack resistance.

Material	Test sample	Duration	Testing Outcome	Penetration
Our material, aluminum foam with ceramic spheres. 40 mm thick panel ${\rho }_{Proteus}^{areal}$ = 71 *kg*/*m*^2^	#4	78 sec	Significant disc wear with small penetration progress.	6–7 mm (18%)
	#5	75 sec	Significant disc wear with small cutting progress	6–7 mm (18%)
	#6	65 sec	Significant disc wear with minimal cutting progress.	7–8 mm (20%)

We have also tested resistance of our material samples to a 36 V cordless drill (see Table 2). When the drill encountered a ceramic sphere on its path, the drilling stopped to progress indefinitely. The power drill missed a ceramic sphere in only one case. Whereas it is much harder for the angle grinder to miss a ceramic sphere because it cuts a line segment through the material, it is possible for the drill bit not to encounter a ceramic sphere when their packing is imperfect or only one layer of ceramics is used. If the ceramic spheres are not packed sufficiently tightly, it might be possible for a 10 mm drill to find a spot between the spheres, and drill through the metallic foam easily. One way to ensure that does not happen is to have many layers of the spheres, which are offset relative to each other. Thereby, even if one layer is missed, there is another layer or two behind the first one to ensure that there are no gaps. If the structure is stochastic, it becomes a statistical problem. If the structure is strictly architected, it is a matter of quality control and precision of the manufacturing. In our initial configuration we only had two sparse layers with associated likelihood of ‘missing’ a sphere approximately 1/9 based on our limited tests.

**Table 2:** Cordless drill resistance.

Material	Test sample	Duration	Testing Outcome	Penetration	Notes
Our material, aluminum foam with ceramic spheres. 40 mm thick panel ${\rho }_{Proteus}^{areal}$ = 71 *kg*/*m*^2^	#10	64 sec	Significant progress	35 mm (88%)	
	#11	30 sec	Complete penetration	40 mm (100%)	The drill did not encounter any ceramic sphere on its path.
	#12	66 sec	Significant progress	30 mm (75%)	

**Figure 10:**
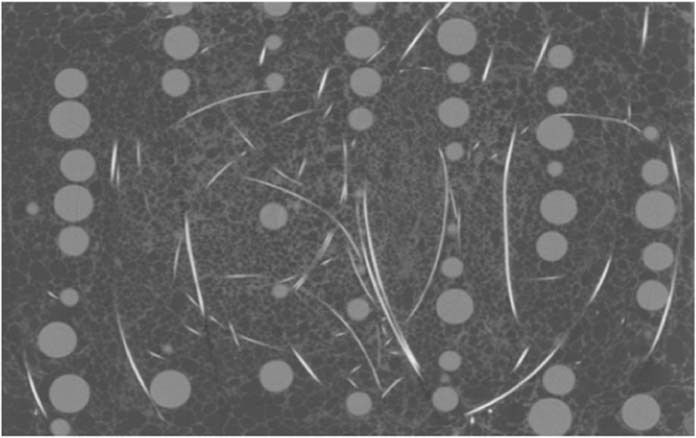
CT cross section of a sandwich material panel with NiCr wires reinforcing the hierarchical cellular structure.

Addition of metallic short fibers (NiCr) in order to reinforce the cellular matrix (as shown in Fig. 10) improved both the cutting and drilling resistance considerably. Embedded wires improved tensile resistance of the cellular material under vibrational load imparted by the rotating disc. Cutting and drilling resistance improved as shown in Tables 3 and 4.

**Table 3:** Angle grinder attack resistance of the panel with NiCr reinforcement.

Material	Test sample	Duration	Testing Outcome	Penetration
Our material, aluminum foam with ceramic spheres. 40 mm thick panel ${\rho }_{Proteus\,NiCr}^{areal}$ = 73 *kg*/*m*^2^	#13	62 sec	Significant disc wear with small penetration progress.	4–5 mm (12%)
	#14	47 sec	Significant disc wear with small cutting progress	5–6 mm (15%)
	#15	57 sec	Significant disc wear with minimal cutting progress.	4–5 mm (12%)

**Table 4:** Cordless drill resistance of the panel with NiCr short fibers.

Material	Test sample	Duration	Testing Outcome	Penetration
Our material, aluminum foam with ceramic spheres. 40 mm thick panel	#16	65 sec	Little progress	7 mm (19%)
	#17	63 sec	Little progress	5 mm (14%)
	#18	64 sec	Little progress	7.5 mm (21%)

**Figure 11:**
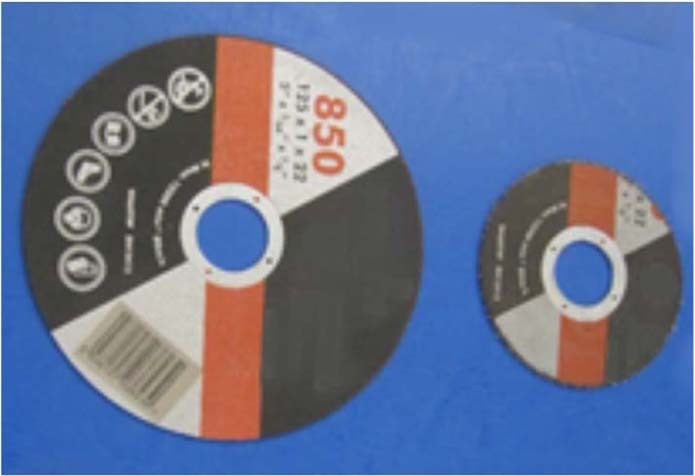
125 mm cutting disc from Marcrist, before and after the cutting tests on our material. Note the massive reduction in the disc diameter from 125 mm to 40 mm (35% of the original dimension).

**Figure 12:**
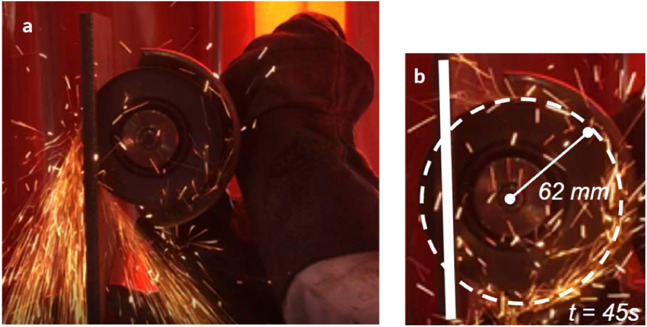
10 mm ultra-high hardness steel plate was cut through with an angle disc (Tyrolit) in under 45 sec.

We compared the cutting resistance of our hierarchical structure with a state-of-the-art armor steel plate distributed by ArcelorMittal under Mars 220 trade name. The rolled-homogeneous armor (RHA) steel has 440 Brinell hardness according to the manufacturer data sheet and is used in protective structures and military hardware. We selected 10 mm thick plate with an areal density of ${\rho }_{Mars}^{areal}$ = 78.5 *kg/m*^2^, which was comparable to the areal density of our material, ${\rho }_{Proteus}^{areal}$ = 71 *kg*/*m*^2^. Whereas our hierarchical structure was effectively non-cuttable by an angle grinder and wore the cutting discs extensively (see Fig. 11), Mars 220 steel plates were penetrated relatively quickly, within 20 seconds typically (see Fig. 12 and Table 5).

**Table 5:** The drill penetrated all samples of the hardened MARS220 steel. Tests were done for benchmarking and comparison.

Material	Test sample	Duration	Testing Outcome	Penetration
MARS 220, armor steel for comparison. 10 mm thick panel ${\rho }_{Mars}^{areal}$ = 78.5 *kg*/*m*^2^	#7	18 sec	Complete penetration	10 mm (100%)
	#8	16 sec	Complete penetration	10 mm (100%)
	#9	15 sec	Complete penetration	10 mm (100%)

We also carried out cutting experiments on cylindrical samples of our material to enable computed tomography (CT) imaging of the partial incisions. We performed cutting tests with the disc aligned with the sphere center, a sphere side, and in-between two closely spaced spheres (see Supplementary Information A for CT images). The cylindrical samples were non-cuttable, and significant disc wear was observed, which rendered the cutting discs ineffective within one minute. We used premium Tyrolit and Marcrist 850 cutting discs, with 115 mm and 125 mm diameters respectively. Marcrist specify that their discs can be used for cutting and grinding metals, concrete, masonry and building materials.

### Section 3. Analytical considerations of vibrations of a rotating disc

Southwell^25^ and Timoshenko^26^ derived an analytical solution for the natural frequencies of a circular disc. The solution gives a lower bound on the natural frequencies of the rotating disc and consists of two components. Firstly, natural frequencies of a stationary, non-rotating disc are computed. Next, one accounts for the centrifugal forces, which make the disc stiffer during the disc rotation because centrifugal inertia is stretching/tensioning the disc.

The natural frequencies of a stationary disc, according to Southwell^25^ are:

9 $${\omega }_{ns}^{stat}=\sqrt{\frac{{\alpha }_{ns}}{{R}^{4}}\frac{D}{\rho \cdot t}}$$

where the bending plate rigidity is denoted as:

10 $$D=\frac{E\cdot {t}^{3}}{12(1-{\nu }^{2})}$$

and the remaining parameters describe the material properties and geometry of a disc. We used the values below to represent a typical cutting disc:

11 $$\begin{array}{rcl}R & = & 57.5\,mm\,({\rm{radius}})\\ t & = & 1\,mm\,({\rm{thickness}})\\ E & = & 33\,GPa\,({\rm{Young}}\,{\rm{modulus}})\\ \nu  & = & 0.3\,({\rm{Poisson}}\,{\rm{ratio}})\\ \rho  & = & 1.85\,g/c{m}^{2}\,({\rm{density}}).\end{array}$$

**Figure 13:**
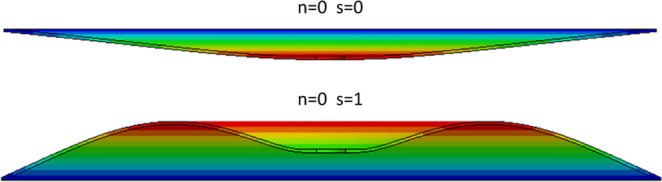
Section view of the radial modes of vibrations. Zero mode resembles deflection under static pressure. Note that usually, the lowest frequency corresponds to *n* = *1 and s* = 0 and not the static deflection mode with *n* = 0 and *s* = *0*.

Parameter *α*_*ns*_ gives the natural frequency for each mode, consisting of *n* angular and *s* radial waves (see Fig. 13) as shown in Table 6.

**Table 6:** *α*_*ns*_ for the range of modal shapes.

	n = 0	n = 1	n = 2	n = 3
s = 0	14.1	2.87	29	156
s = 1	438	422	1210	2840

The natural frequencies in cycles per second for a stationary, circular disc, using material properties and geometry from (11), are shown in Table 7:

**Table 7:** Natural frequencies of a typical cutting disc, ${f}_{ns}^{stat}$.

	n = 0	n = 1	n = 2	n = 3
s = 0	231 Hz	**104.2 Hz**	331.3 Hz	768.4 Hz
s = 1	1287.6 Hz	1263.9 Hz	2140.1 Hz	3278.7 Hz

One should note that the lowest natural frequency is associated with *n* = 1 and *s* = 0, and not *n* = 0 and *s* = 0. The rotation of the disc adds centrifugal forces, which stretch the disc and increase its natural frequencies. The effect is similar to tensioning a string in a musical instrument, which increases the frequency of its free vibrations and, consequently, its pitch. In order to incorporate that effect, frequencies of a rotating, flexible disc with no appreciable bending rigidity need to be computed first. Southwell^25^ and Timoshenko^26^ reported the formula for free vibrations of a flexible disc as:

12 $${\omega }^{rotat}({\lambda }_{ns},{\omega }_{disc})=\sqrt{{\lambda }_{ns}\cdot {\omega }_{disc}^{2}}$$

where *ω*_*disc*_ is the rotational velocity of disc in [rad/sec] and *λ*_*ns*_ corresponds to the modes of vibrations explained above and is given as shown in Table 8:

**Table 8:** *λ*_*ns*_ for the range of modal shapes.

	n = 0	n = 1	n = 2	n = 3
s = 0	0	1	2.35	4.05
s = 1	3.3	5.95	8.95	12.3

Thereby, the frequencies of a flexible disc rotating at a typical rotational velocity of a cutting disc, namely *ω*_*disc*_ = 11000 *rpm*, are shown in Table 9:

**Table 9:** Natural frequencies, *f*_*ns*_^*flex*^ of a flexible disc rotating at 11000 *rpm*.

	n = 0	n = 1	n = 2	n = 3
s = 0	0 Hz	183.3 Hz	281 Hz	369 Hz
s = 1	333 Hz	447.2 Hz	548.5 Hz	643 Hz

The lower bound on natural frequencies of a rotating disc, considering the actual disc material properties and the effect of the centrifugal forces can be approximated as a geometric average of the frequencies of a stationary disc and natural frequencies of a rotating flexible disc, namely:

13 $${\omega }_{ns}=\sqrt{{({\omega }_{ns}^{stat})}^{2}+{({\omega }_{ns}^{rotat})}^{2}}$$

Therefore, the natural frequencies of our rotating disc have lower bounds given by:

With the fundamental natural frequency of:

14 $${f}_{1,0}=210.9\,Hz$$

Considering the rotation of the disc at:

15 $${f}_{disc}=11000\,rpm/60\,s=183.3\,Hz$$

The excitation experienced by the sphere from the forward and backward travelling waves of the fundamental mode, using Table 9 for *n* = 1 and *s* = 0 and referring to Eq. (5) are:

16 $${f}_{1,0}^{sphere}=1\cdot \left(\frac{210.9\,Hz}{1},\pm ,183.3,\,,H,z\right)=210.9\pm 183.3\,Hz$$

Analogously for the next analytical mode (n = 2 and s = 0), the frequencies of the forward and backward travelling waves are:

17 $${f}_{2,0}^{sphere}=2\cdot \left(\frac{434.5\,Hz}{2},\pm ,183.3,\,,H,z\right)=434.5\pm 366.6\,Hz$$

One can observe that a higher order wave (with n = 2, 3, …), when coupled with the disc rotations, can produce a forward travelling wave with a significantly higher frequency, and consequently higher frequency vibrations applied to the embedded sphere than the baseline natural frequency of the disc, for example:

18 $${f}_{2,0}^{F}=434.5\,Hz+366.6\,Hz=801.1\,Hz\gg 434.5\,Hz$$

### Section 4. Finite element analysis of the cutting disc vibrations

**Figure 14:**
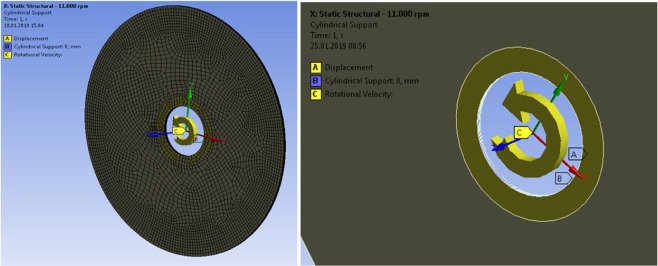
Orientation of the coordinate system and application of boundary conditions. ‘A’ indicates displacement in z-direction = 0, ‘B’ indicates cylindrical support in inner diameter and ‘C’ is Rotational Velocity of all bodies.

ANSYS software was used to calculate the natural frequencies of the used disc. A finite element model of the actual cutting disc was created (Fig. 14). The disc was modelled as 8 layers because the cutting blade is made of a layered glass fiber reinforced composite. We modelled the disc with 115 mm diameter and 25 mm cylindrical hole in the center. We applied the boundary conditions by restraining out of plane deflections and out of plane rotations of the region in the range of 25 *mm* < *d* < 40 *mm* to represent the clamping applied to the disc in the angle grinder. The model was meshed using thin solid elements (SOLID 186 from ANSYS library of elements). Orientation of the coordinate system and application of the boundary conditions are shown in Fig. 14. We used material properties of the disc as given below:

*E*_*x*_ = 52 *GPa* (Young modulus in x-direction)

*E*_*y*_ = 14 *GPa*

*E*_*z*_ = 14 *GPa*

*ν*_*xy*_ = 0.28 (Poisson ratio in x-y plane)

*ν*_*yz*_ = 0.30

*ν*_*xz*_ = 0.28

ρ = 1.85 *g cm*^3^.

The orientation of the composite layers employed in the model is shown in Table 11. Note that the material properties are given in the local coordinate system and are rotated in each layer to produce relatively axi-symmetric material properties.

**Table 10:** Natural frequency of a stiff, rotating disc, *f*_*ns*_ =.

	n = 0	n = 1	n = 2	n = 3
s = 0	231 Hz	210.9 Hz	434.5 Hz	852.4 Hz
s = 1	1330 Hz	1340.6 Hz	2209.3 Hz	3341.2 Hz

**Table 11:** Thickness and orientation of glass fibers in a composite cutting disc.

layer	Thickness [mm]	Angle, x-direction
1	0.125	0°
2	0.125	90°
3	0.125	45°
4	0.125	−45°
5	0.125	45°
6	0.125	−45°
7	0.125	90°
8	0.125	0°

**Figure 15:**
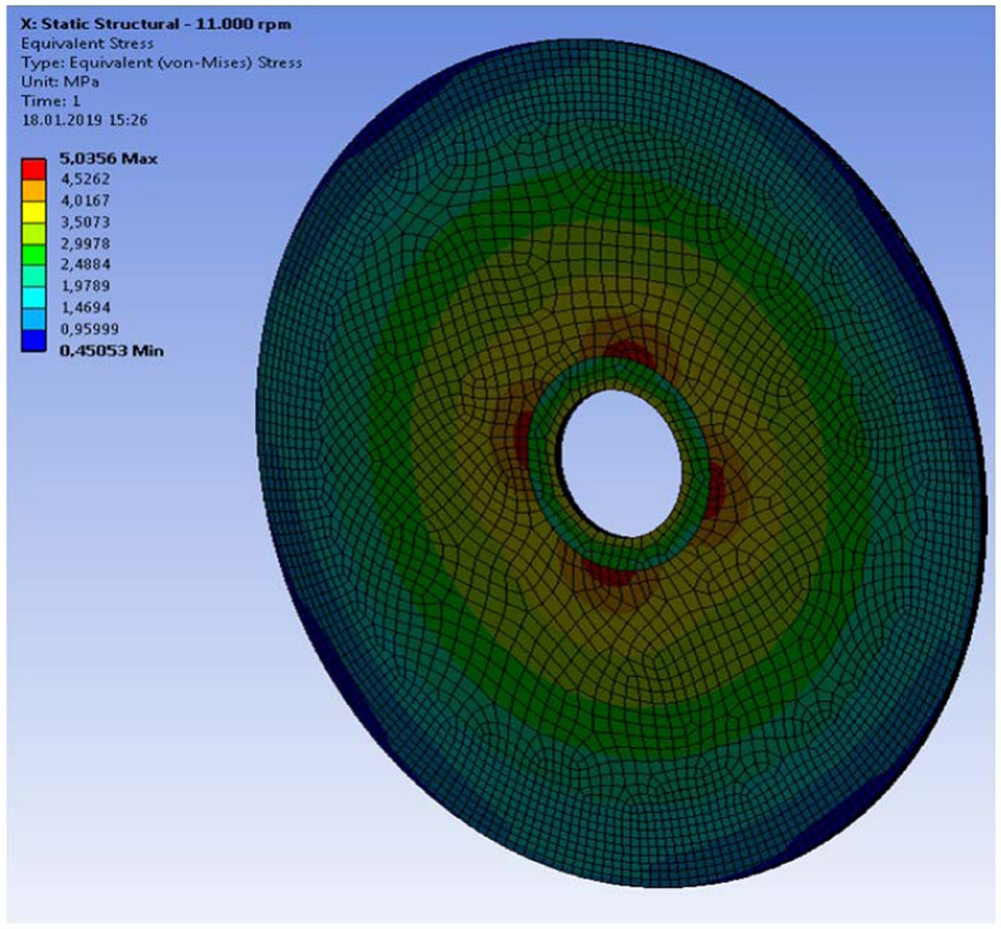
Von Mises (uniaxial test equivalent) stress at 11.000 rpm at the top layer with fibers aligned with x-axis. Although each layer had a preferred direction and slightly non-uniform stress distribution, the stiffness of all eight layers was isotropic from the practical point of view.

A rotational velocity of 11,000 *rpm* or 1152 *rad*/*s* was applied to the disc. In the first step a static-structural simulation allowed us to compute the stresses produced by the centrifugal force. The resulting maximum stresses are in the range of 5 MPa (Fig. 15).

**Figure 16:**
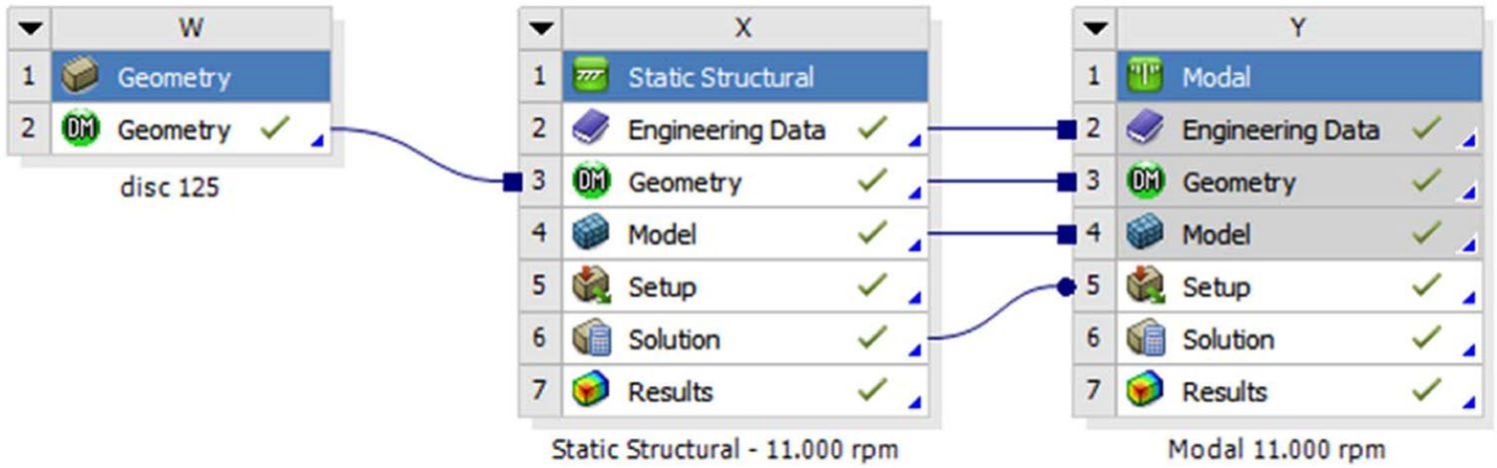
Transfer of centrifugal stress into modal analysis of the disc in order to compute frequencies of vibrations, which account for the stiffening from centrifugal forces.

In the second step the centrifugal stresses were used to setup eigenmode analysis to find the natural frequencies and vibration modes of the rotating disc as shown in Fig. 16.

**Figure 17:**
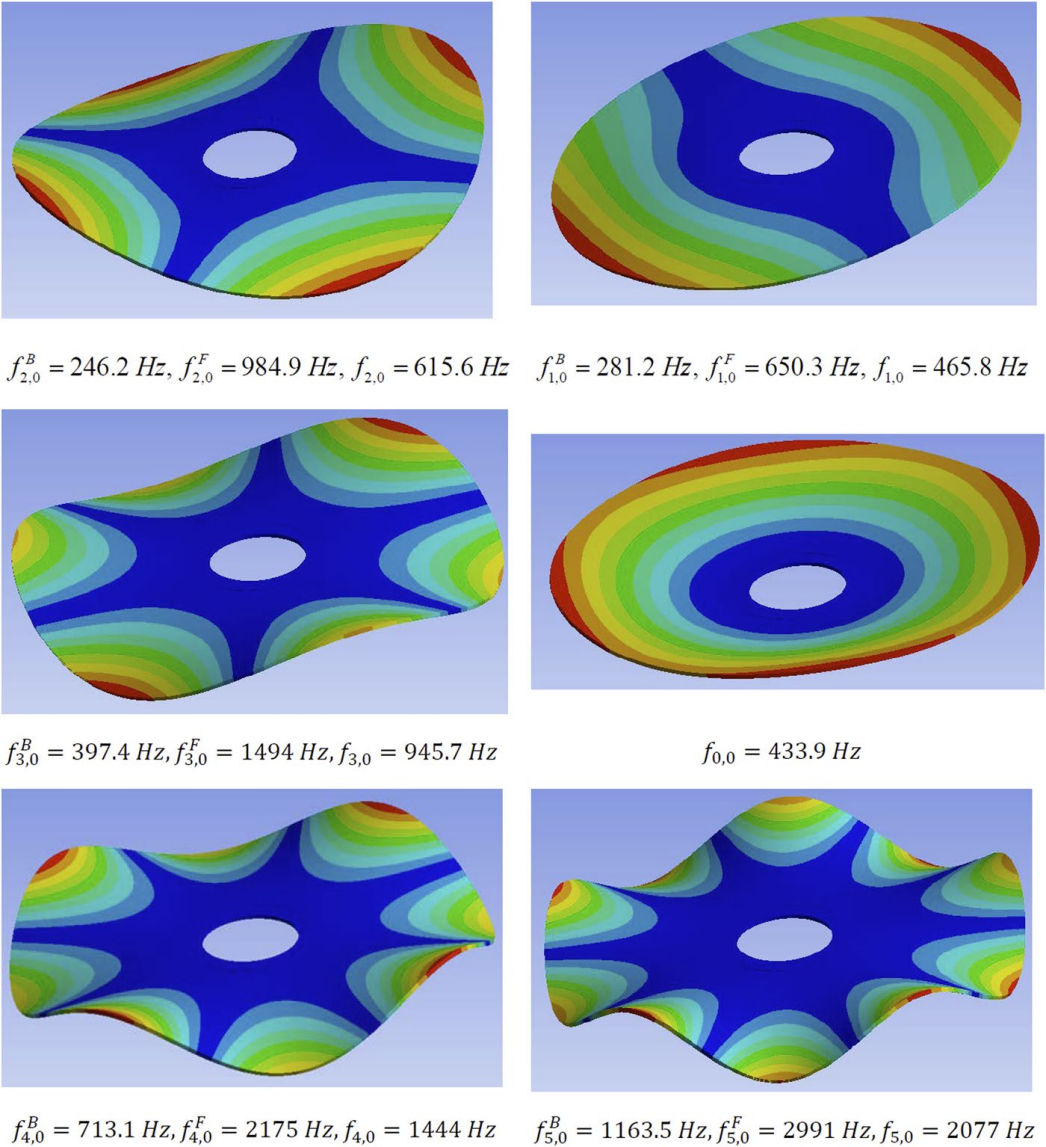
Free vibration modes: natural frequencies, and frequencies of the travelling waves. The modes are ordered from the lowest to the highest frequency of the backward travelling wave or the natural frequency for the modes with radial oscillations only.

The results of the modal analysis are listed in Fig. 17. Modes and shapes of vibrations differ from the theoretical solution because the numerical model accounts for a large circular opening in the center of the disc. It also used the orthotropic composite material properties. The backward and forward travelling waves are of primary importance for our analysis. These frequencies are summarized in Tables 12 and 13. For the sake of completeness, frequencies of the radial modes are given in Table 14.

**Table 12:** Frequencies of backward travelling waves.

	n = 0	n = 1	n = 2	n = 3
s=0	—	**281.2 Hz**	**246.2 Hz**	**397.4 Hz**
s=1	—	**2401.9 Hz**	**2585.9 Hz**	**3138.7 Hz**

**Table 13:** Frequencies of forward travelling waves.

	n = 0	n = 1	n = 2	n = 3
s = 0	—	**650.3 Hz**	**984.9 Hz**	**1494.5 Hz**
s = 1	—	**2751.4 Hz**	**3324 Hz**	**4135.7 Hz**

**Table 14:** Natural frequencies, which do *not* have forward and backward travelling waves but are axisymmetric waves resembling water ripples.

	n = 0	n = 1	n = 2	n = 3
s = 0	**433.9 Hz**	—	—	—
s = 1	**2475 Hz**	—	—	—

### Section 5. Natural frequencies of ceramic sphere(s) embedded in cellular aluminum

**Figure 18:**
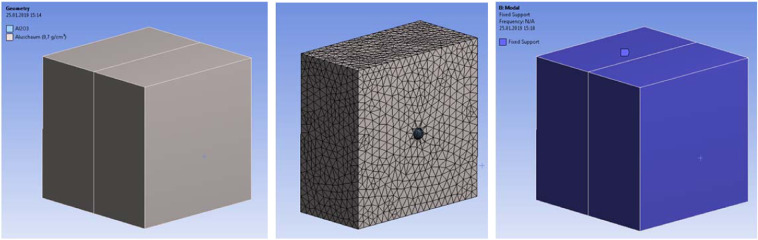
Finite element model of a ceramic sphere embedded in a cellular metallic matrix. We used a homogenized representation with Young’s modulus and density of our aluminum foam material. The ceramic sphere was modelled as an elastic sphere with the mechanical properties of alumina ceramics.

The model was created in ANSYS Workbench. Firstly, we considered the vibration of a single ceramic sphere with a diameter of 13 mm placed in the center of the cube (400 mm × 400 mm × 400 mm). The ceramic sphere and the surrounding media shared nodes (Fig. 18). Thereby, contact elements were not needed. The bodies (ceramic sphere and the surrounding solid material representing metallic foam) were meshed with 72,400 elements. The aluminum foam body was fixed on the outer boundaries.

**Figure 19:**
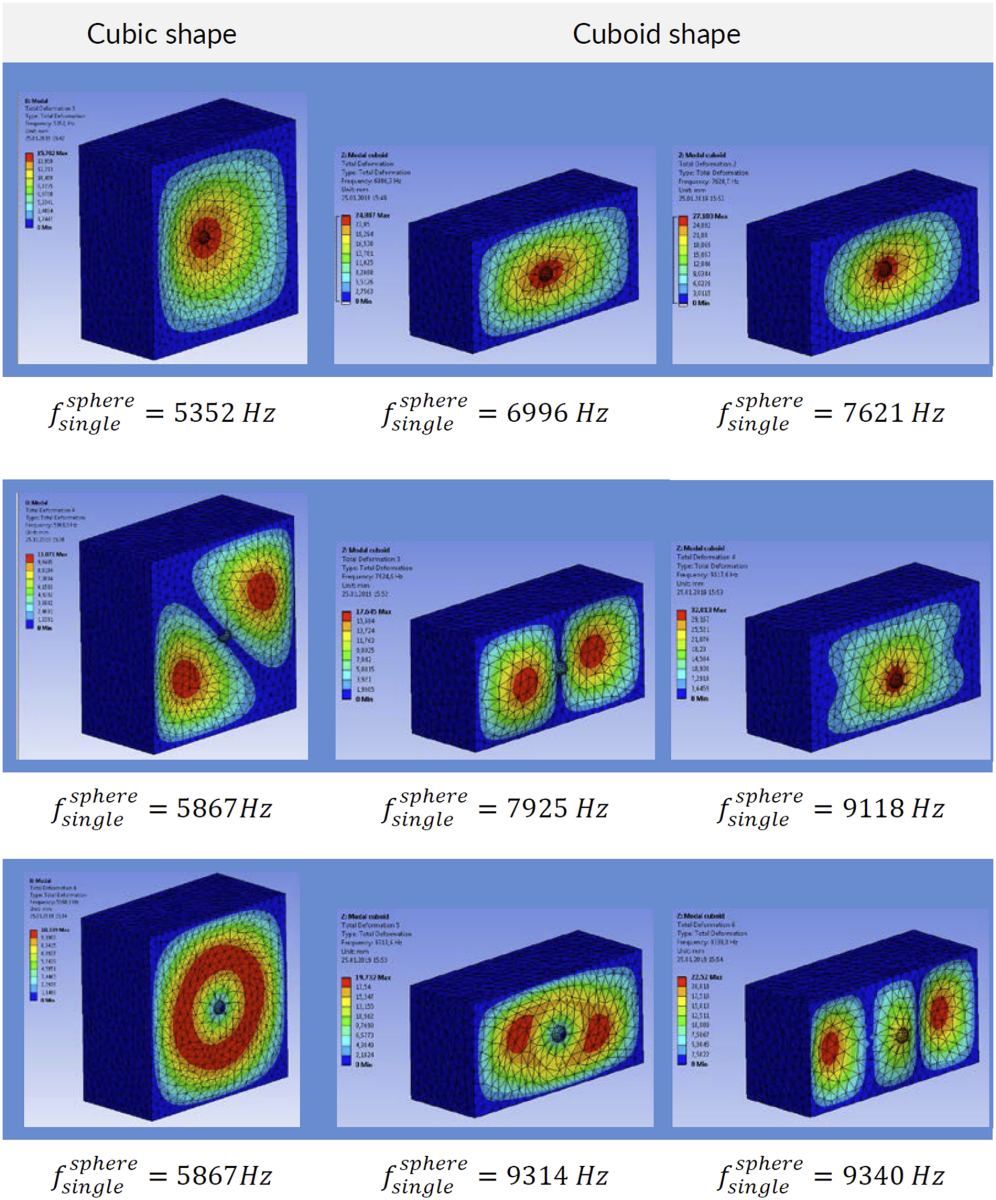
Modal solution for a cubic block (400 × 400 × 400 mm^3^) and cuboid (400 × 300 × 200 mm^3^) of aluminum foam with *one* embedded ceramic sphere. The differences highlight the effect of the panel thickness on the range of natural frequencies.

**Figure 20:**
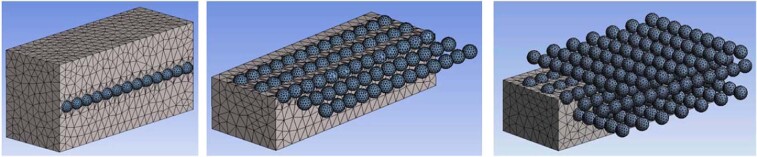
Expanded finite element model of ceramic spheres embedded in a cellular metallic matrix. A line of ceramic spheres is shown on the left, the plane of spheres in the center, and spheres ordered in 3 parallel planes on the right.

**Figure 21:**
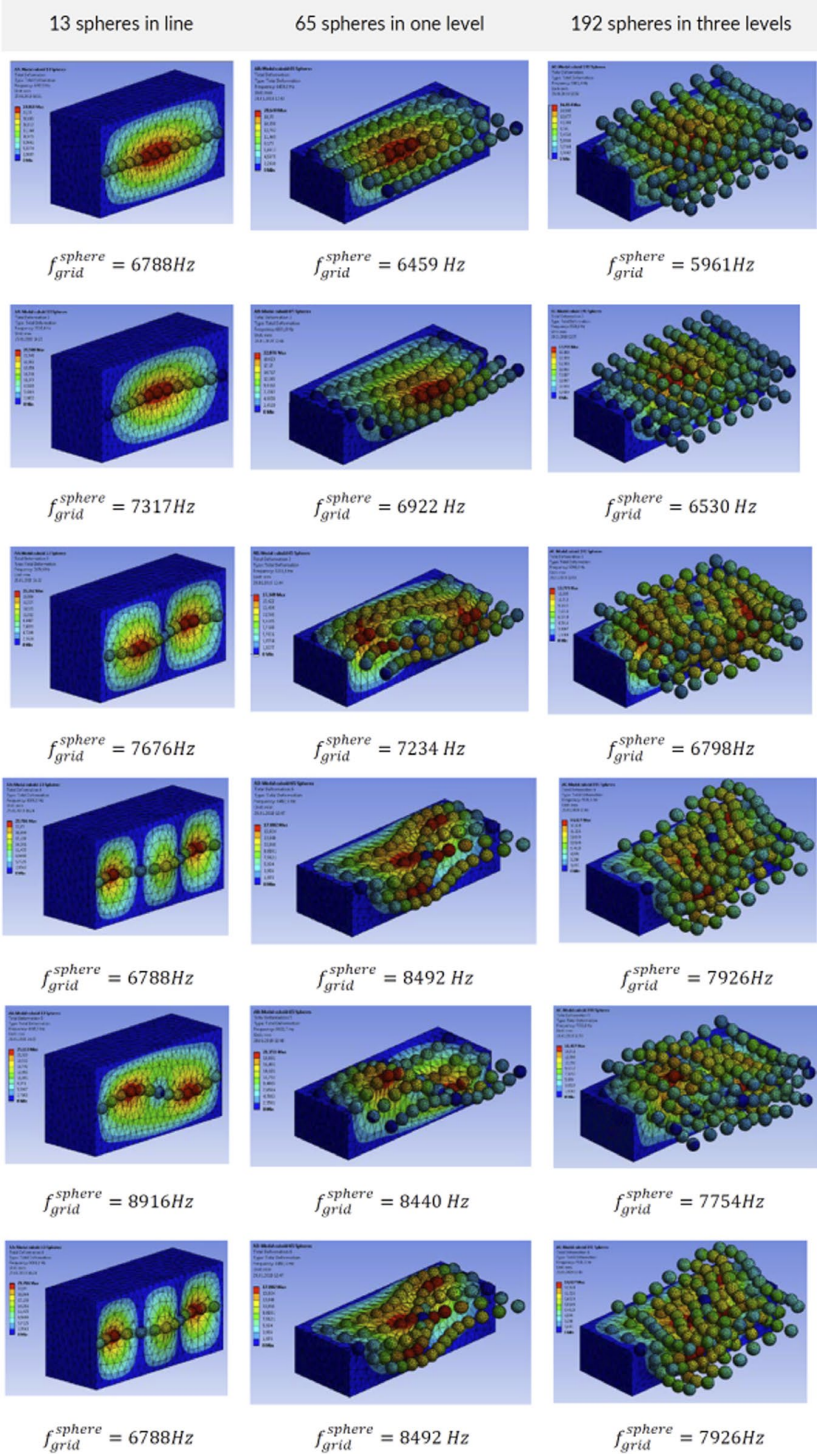
Modal solution for a cuboid-shaped foam body (400 × 300 × 200 mm^3^) with a line of 13 spheres, 65 spheres in the plane and with three layers of 192 embedded spheres. Increasing the number of spheres reduced the natural frequency due to increase of the average medium density.

The natural vibrations of a ceramic sphere embedded in metallic foam depend on the proximity of the boundaries such as our faceplates (see Fig. 19). Bringing the boundaries closer to the sphere increases its natural frequency. We have also analyzed the effect of multiple ceramic spheres by computing natural frequencies for cases with a row of ceramic spheres and an array of spheres (Fig. 20) and a grid of spheres (Fig. 21). The presence or absence of the ceramic spheres in the surrounding material influences the natural frequencies. The inclusion of the ceramic material increases the density of the surrounding media because alumina has a greater density of *ρ*_*Al*203_ = 3950 *kg*/*m*^3^, then aluminum foam at *ρ*_*f*_ = 730 *kg*/*m*^3^. At the same time, the spatial grid of the ceramic spheres has a minor effect on the homogenized material stiffness because it is controlled by the compressibility of the compliant foam in between ceramic spheres as shown in our mechanical tests (see Fig. 3). Increased mass density reduced the natural vibrational frequencies, which is consistent with the results shown in Figs. 20 and 21.

We can conclude that the computed frequencies of our models range from ${f}_{single}^{sphere}=5352\,Hz$ to ${f}_{single}^{sphere}=7621\,Hz$ depending on the proximity of the boundaries. Packing the cellular structure with three multiple layers of ceramic spheres would give ${f}_{grid}^{sphere}=5961\,Hz$, which is likely to be in line with the fundamental frequency of our metamorphic material, ‘Proteus’. Interestingly, a multitude of higher modes of vibrations is also possible, because they are closely spaced, with frequency gaps in the range of hundreds of Hz only see (Fig. 19).

### Section 6. Chemical composition of the cutting discs, metallic foam and ceramic spheres

**Figure 22:**
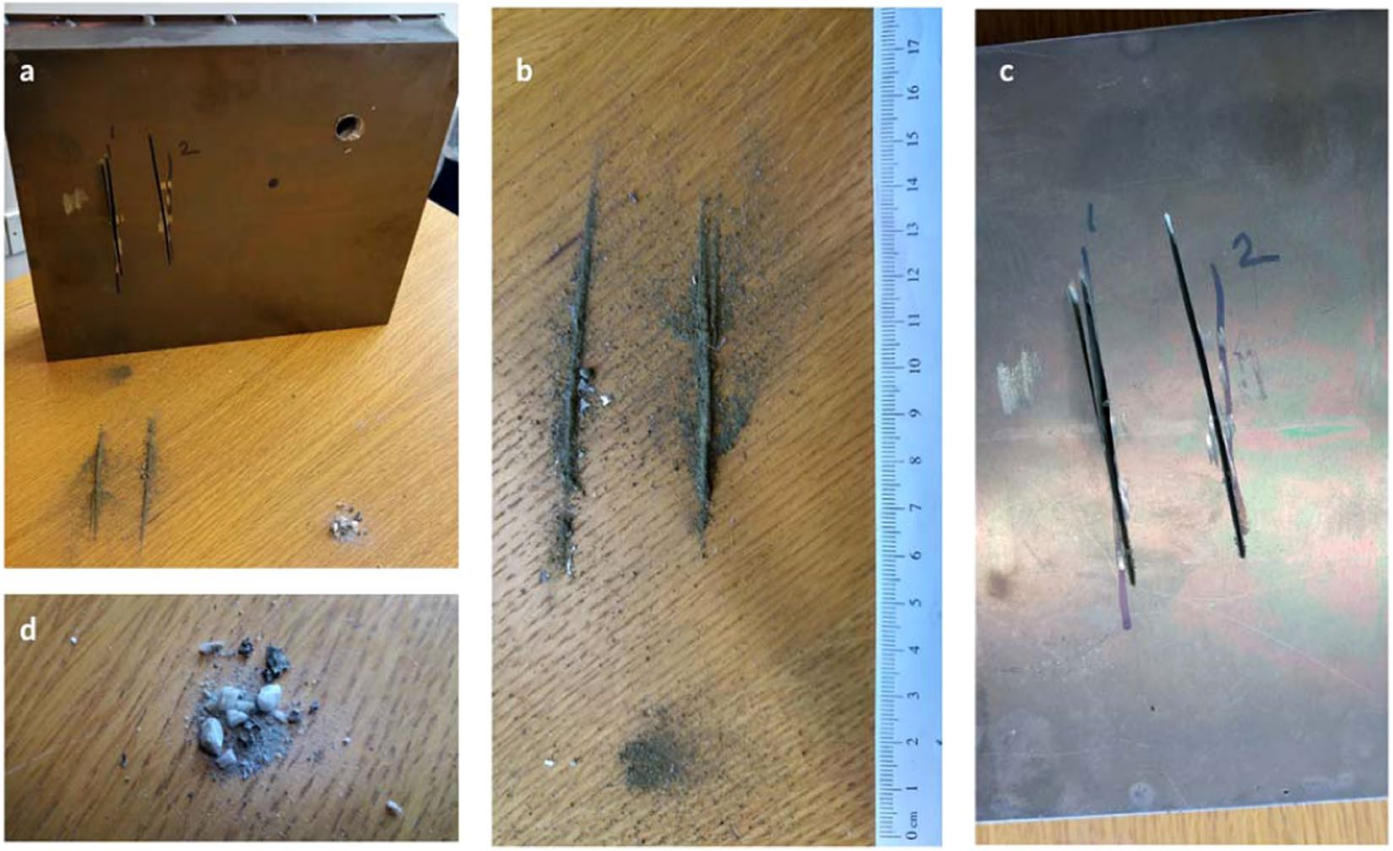
Powder resulting from the unsuccessful cutting attempts of the sandwich panel sample (245 mm ×172 mm ×40 mm) was collected for EDX analysis and SEM images. (**a**) specimen and the particulate matter, (**b**) powder from angle grinder cutting attempts, (**c**) corresponding partial incisions in the panels, (**d**) particles and particulate matter following drilling attempt.

Typical cutting discs are made of proprietary fiber glass reinforced composites. Marcrist supplied us with Material Safety Data Sheet (no M SDS-02-0001-2), which disclosed the chemical components in the cutting disc (Table 15). It contains up to 20% of fiberglass reinforcement and significant proportions of ceramics such as aluminum oxide, silicon carbide and zirconium oxide. We also extracted particulate matter from the incision in plate panes shown in Fig. 22. Microscopic analysis of the geometrical shapes and size of the particles is shown in Fig. 7(d–f).

**Table 15:** Composition of the cutting disc supplied by the manufacturer (MSDS).

Component	%
Brown/Blue/white Aluminum Oxide	0–70
and/or Silicon Carbide	0–70
and/or Zirconium Oxide	0–30
Cured Phenolic Resin	5–20
and/or Cubitron	0–20
and/or Woven Fiberglass	0–20
and/or Iron Pyrite	0–20
and/or Cryolite	1–10
and/or Titanium Dioxide	0–5
and/or Calcium Oxide	0–5
and/or Barium Sulfate	0–5
and/or Sulfur	0–5
and/or Zinc Sulfide	0–5
and/or Magnesium Oxide	0–5
and/or Iron Oxide	0–5
and/or Graphite	0–5
and/or Potassium Fluoroborate	0–5
and/or Calcium Carbonate	0–2

**Figure 23:**
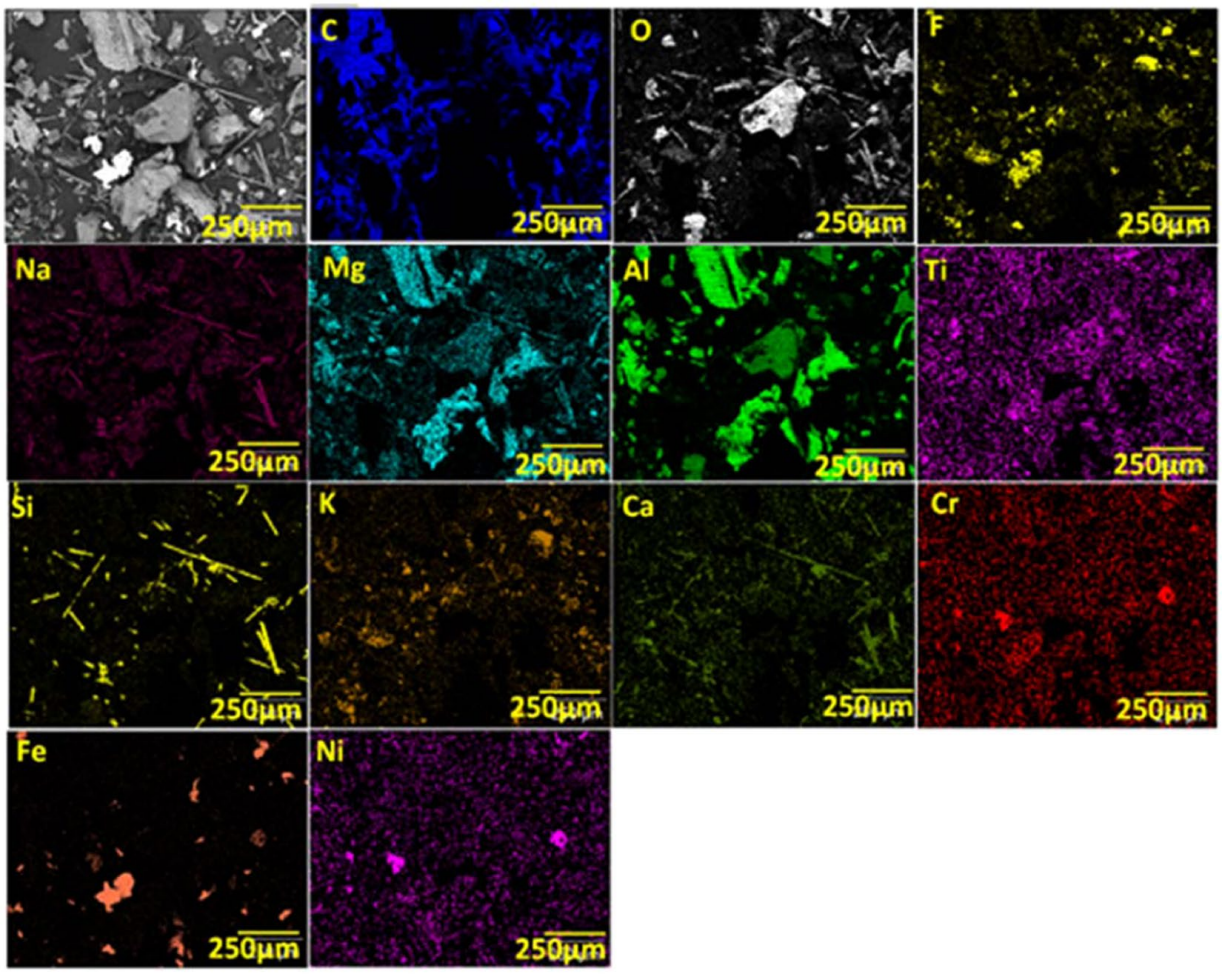
EDX mapping of the powder content extracted from the partial cut of our material sample. Carbon (C) as well as Silicon (Si) indicates glass fibre, SiO2 fragments. Oxygen (O) corresponding to the powder particles containing oxygen such as Aluminium (Al) Oxide, Magnesium (Mg) Oxide, Calcium (Ca) Oxide, Iron (Fe) Oxide and Titanium (Ti) Dioxide. Fluor (F) and Potas (K) indicates Potassium Fluoroborate. Iron (Fe), Nickel (Ni) and Chromium (Cr) identify stainless steel shards.

We also carried out energy-dispersive X-ray spectroscopy (EDX) to shed light on the chemical elements in the extracted particulate matter. Based on the images in Fig. 23, we estimated the content of chemical elements in the individual powder particles. EDX analysis of the powder (Table 16) was consistent with the datasheet supplied by the cutting disc manufacturer, Marcrist (Table 15). We also found particles originating from our cellular aluminum foam matrix and alumina oxide ceramic spheres.

**Table 16:** The extracted spectrum of EDX analysis presenting the element content of the partial cut of our material sample.

Element	Weight %	Atom %
C*	37.0	55.4
Al	34.6	22.2
O	12.7	14.3
Fe	6.2	2.0
F	2.4	2.3
Si	2.0	1.3
K	1.6	0.7
Ni	0.9	0.3
Ca	0.9	0.4
Mg	0.7	0.5
Ti	0.4	0.2
Na	0.3	0.2
Cr	0.3	0.1

*Note: The high carbon content is an artefact of the sample preparation process and is not indicative of the true carbon value in the extracted powder.

### Section 7. Computer tomography of material samples

Computed tomography (CT) is used increasingly in material science, as it enables a fast and non-destructive visualization of internal structures. CT is based on the collection of X-ray absorption images of a specimen from different rotational angles. In the reconstruction process, the 3D volume is calculated from the X-ray images. It consists of so-called voxels, whose edge length mainly defines the resolution of the measurement. The grey value of each voxel reflects the local X-ray absorption characteristic. A high grey value represents a high X-ray absorption. This representation enables one to distinguish different materials in the CT measurement.

**Figure 24:**
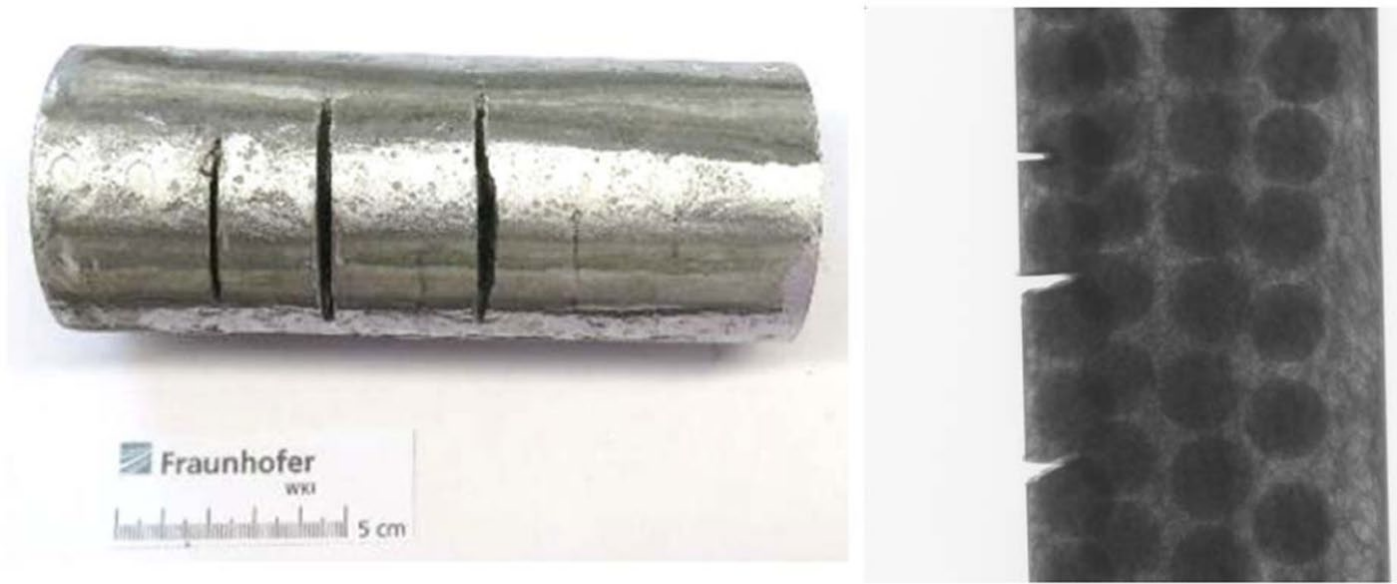
Cylindrical material sample after angle grinder cutting attack accompanied by an x-ray transmission image. A series of transmission images is the basis of CT measurements.

**Figure 25:**
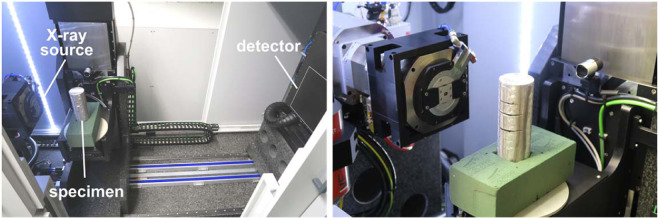
Micro-CT setup of measurements performed at Fraunhofer-Institut für Holzforschung, Wilhelm-Klauditz Institut WKI in Hannover, Germany. The specimen with cuts produced by an angle grinder was rotated and X-ray transmission images were recorded to enable reconstruction of the 3D material structure.

CT was especially suitable for the inspection of the metallic foam specimens because the exact interaction of our meta-material and an angle grinder was not externally observable. Due to the highly wear-resistant nature of our hierarchical structure, preparation of microsections would be very difficult, if not impossible to achieve. A cylindrical specimen was manufactured for the CT measurements (150 mm tall with 60 mm diameter as shown in Fig. 1c and in Fig. 24 below). The CT measurement (Fig. 25) allowed us to uncover the 3-dimensional structure of the material in the cutting region, including the course of the cuts, the distribution of powder in the foam structure, and the presence of defects such as cracks following the cutting attacks.

The spatial resolution is dependent on the measuring geometry. A small distance between the focal point and the rotation axis is often desirable for a high magnification. When the specimen itself is larger than the scanning volume – the region of interest (ROI) – the outer dimensions of the specimen can physically prevent the scanning volume from being located as close to the focal point as desired. Furthermore, material outside the ROI, which has to be penetrated by the X-rays, reduces scan quality due to the additional absorption of X-rays and reconstruction artefacts. The cylindrical shape of the specimen reduced the specimen volume to the ROI, enabling a positioning close to the focal point and avoiding specimen material outside the ROI as would be the case for a rectangular plate specimen. In addition, the CT measurement relies on the partial penetration of the X-rays through the specimen in every rotational stage. Complete absorbance can occur for example when a wall, consisting of a highly absorptive material like steel, is oriented parallel to the beam path. This can cause massive reconstruction artefacts and is prevented by the cylindrical geometry. The thin metallic face sheet on the cylindrical sample also acted as an X-ray filter reducing the X-ray beam hardening inside the specimen.

Fraunhofer Wilhelm-Klauditz-Institut WKI in Hannover, Germany performed the scans on a Procon X-Ray CT-AlphaDuo device. To maximize the resolution, while capturing the complete region of interest (a cylinder with a height of 70 mm and a diameter of 60 mm), the CT scan was conducted in helix mode combined with a measuring field extension. Focus-object-distance and focus-detector-distance were set to 188 mm and 1300 mm respectively. X-ray parameters were a voltage of 230 kV and a current of 100 µA. 4161 projections averaging 6 images with an integration time of 450 ms each were collected. The voxel resolution of the reconstructed volume amounts to 19.9 µm. For visualization of the volume data VGSTUDIO MAX 3.0 was used.

## Supplementary information

Movie_01.mp4.

Movie_02.mp4.

Movie_03.mp4.

Movie_04.mp4.

Movie_05.mp4.

Movie_06.mp4.

Movie_07.mp4.

Movie_08.mp4.

Movie 09.mp4.

Movie_10.mp4.

Movie_11.mp4.

Suppl_Information_A_CT.

Suppl_Information_B.

Suppl_Information_C_SEM.

Supplementary information 15.
