# Publisher Correction: Non-cuttable material created through local resonance and strain rate effects

**DOI:** 10.1038/s41598-020-75485-9

**Published:** 2020-10-30

**Authors:** Stefan Szyniszewski, Rene Vogel, Florian Bittner, Ewa Jakubczyk, Miranda Anderson, Manuel Pelacci, Ajoku Chinedu, Hans-Josef Endres, Thomas Hipke

**Affiliations:** 1 Durham University, Durham, United Kingdom; 2 Fraunhofer Institute for Machine Tools and Forming Technology IWU, Chemnitz, Germany; 3 Fraunhofer Institute for Wood Research, Wilhelm-Klauditz-Institut WKI, Hannover, Germany; 4 Leibniz University Hannover, Institute of Plastics and Circular Economy IKK, Garbsen, Germany; 5 University of Surrey, Guildford, United Kingdom; 6 University of Stirling, Stirling, United Kingdom

Correction to: *Scientific Reports* 10.1038/s41598-020-65976-0, published online 20 July 2020

The original HTML version of this Article contained errors.

In Equations 4, 16 and 17 additional commas were incorrectly displayed.

Additionally, the Supplementary information files of this Article contained an incorrect version of Supplementary movie 11. Furthermore, Supplementary information 15 was erroneously published.

These errors have now been corrected in the HTML version and its Supplementary information files; the PDF version was correct at the time of publication.

