# Supplementary material for: Non-cuttable material created through local resonance and strain rate effects
Source: Sci Rep. 2020 Jul 20;10:11539. doi: 10.1038/s41598-020-65976-0 (PMC7371712; doi:10.1038/s41598-020-65976-0)

Creation of non-cuttable material through the use of local resonance and strain rate effects

Stefan Szyniszewski [1], Rene Vogel [2], Florian Bittner [3,4], Ewa Jakubczyk[5], Miranda Anderson [6], Manuel Pelacci[1], Ajoku Chinedu [1], Hans-Josef Endres [3,4], Thomas Hipke [2]*

[1] Durham University, Durham, United Kingdom
[2] Fraunhofer Institute for Machine Tools and Forming Technology IWU, Chemnitz, Germany
[3] Fraunhofer Institute for Wood Research, Wilhelm-Klauditz-Institut WKI, Hannover, Germany
[4] Leibniz University Hannover, Institute of Plastics and Circular Economy IKK, Garbsen, Germany
[5] University of Surrey, United Kingdom
[6] University of Stirling, United Kingdom

Supplementary Information A:
Full set of images from CT-scans of the cylindrical sample

Fig. 1: Internal structure of our cylindrical sample

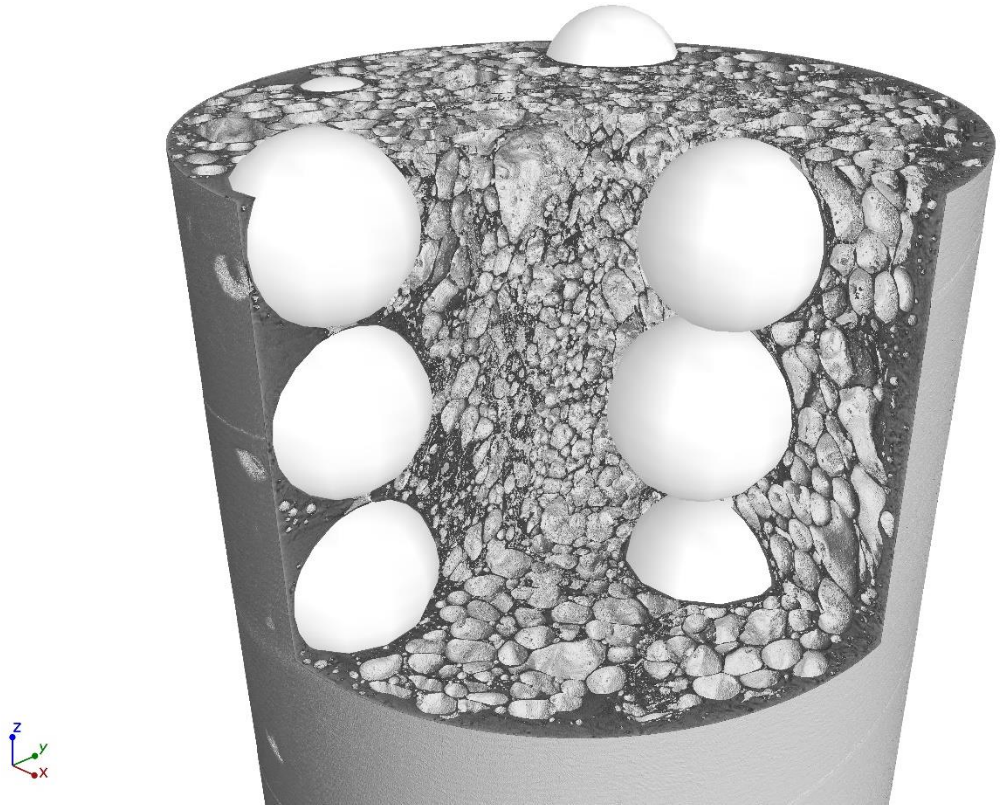

Fig. 2: Location of ceramic spheres in a cylindrical sample

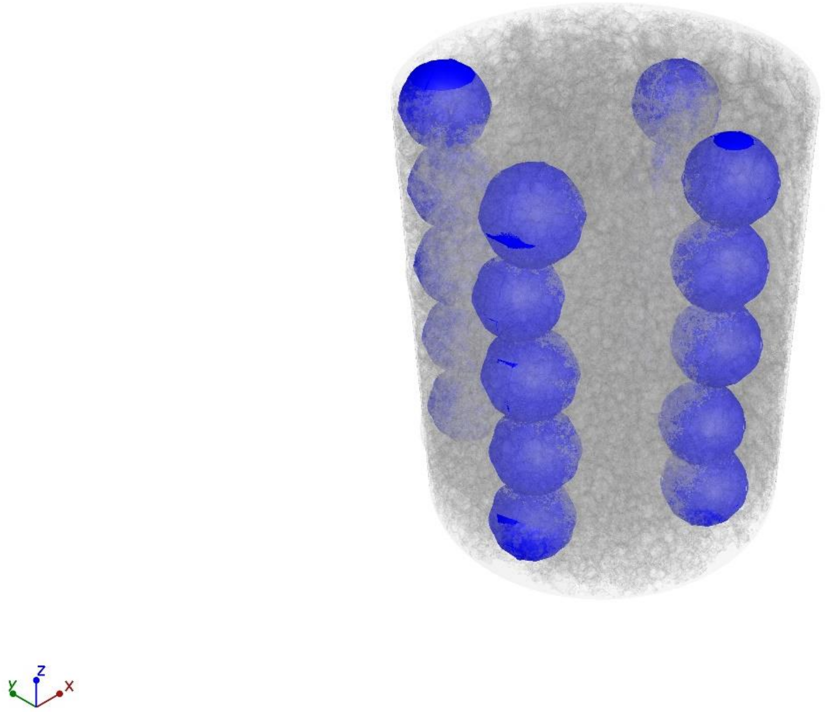

Fig. 3: Cross section of our cylindrical sample illustrating the wall thickness distribution of the aluminium foam

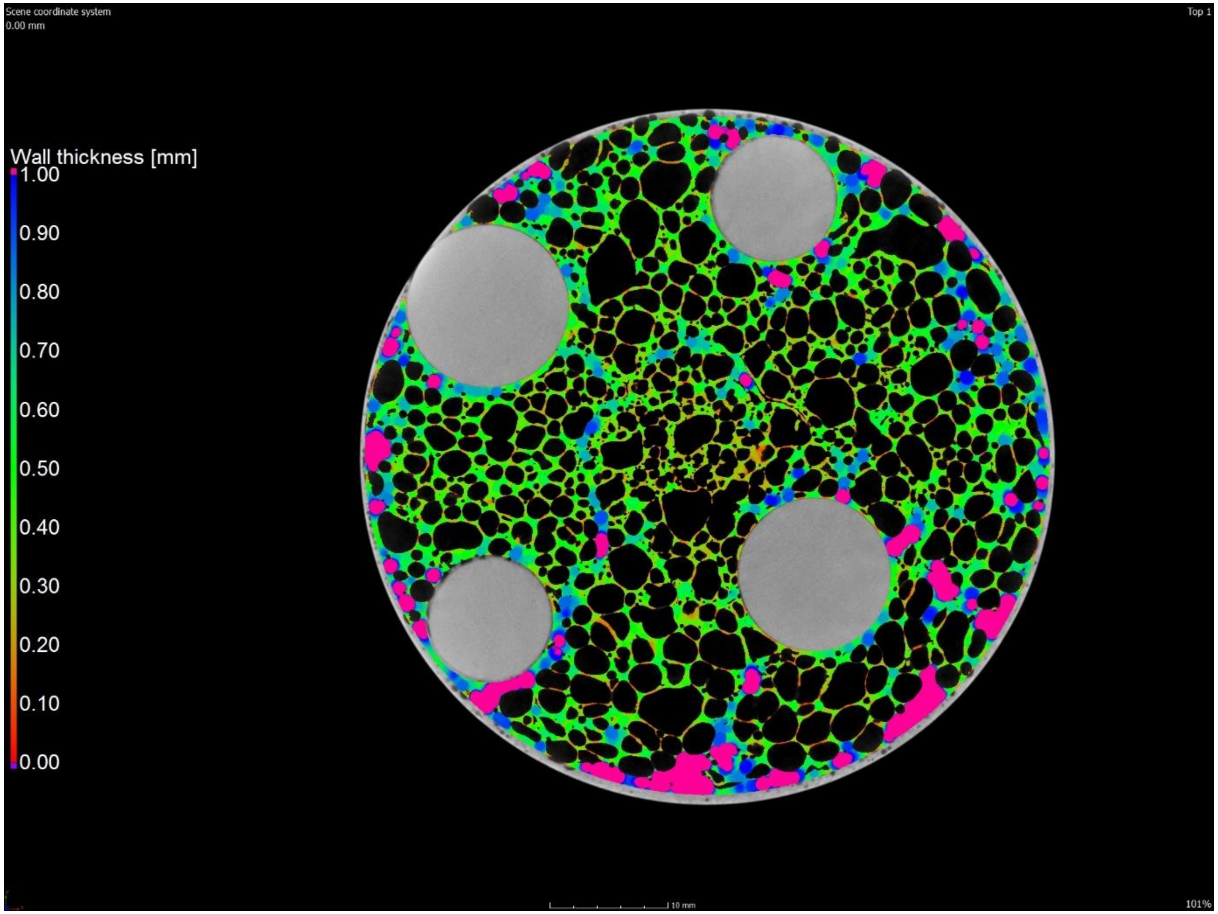

Fig. 4: Distribution of aluminium foam cell volume calculated for an exemplary area of the cylindrical sample

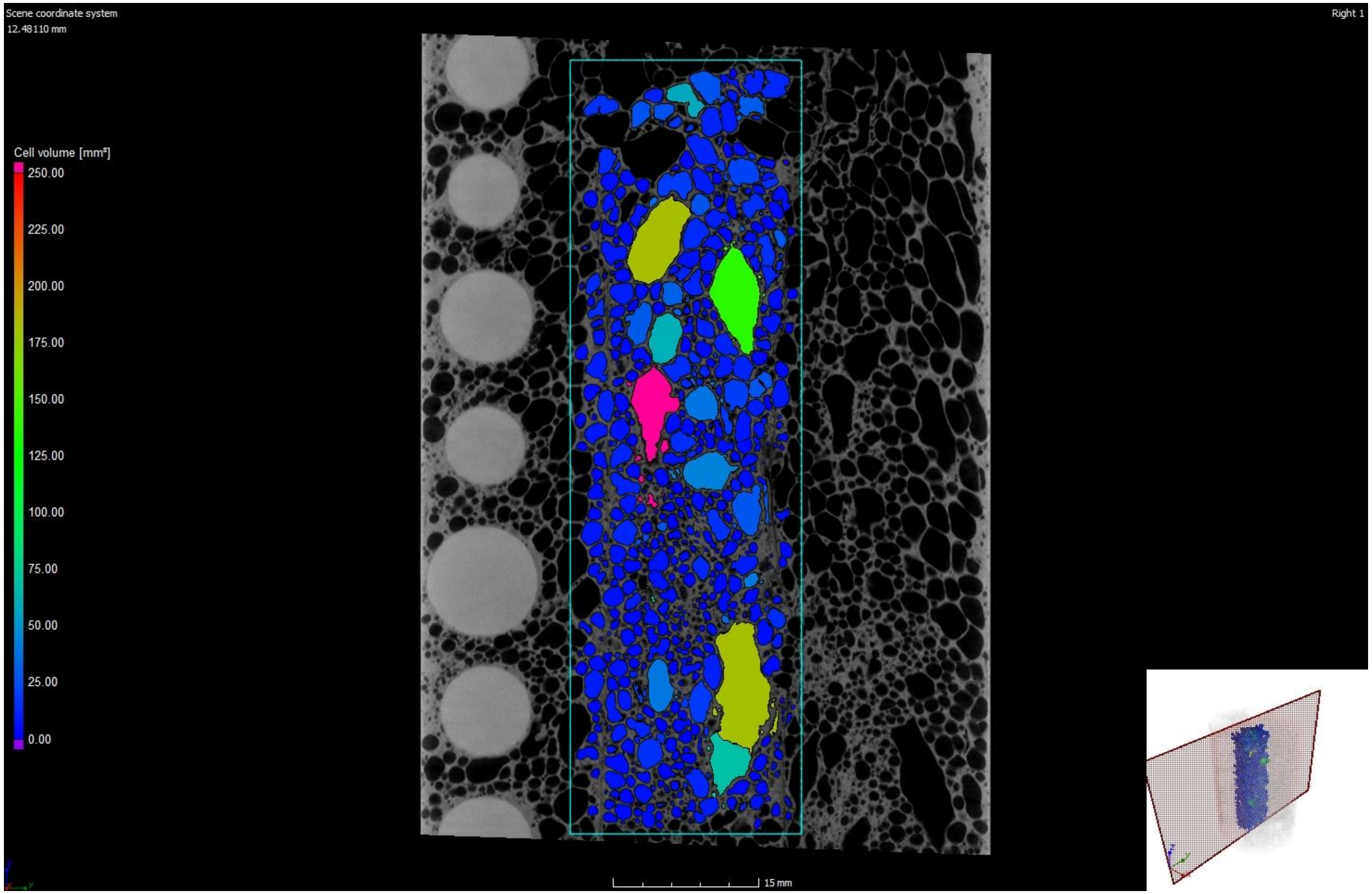

Fig. 5: Powder aggregation adjacent to the centrical cut of a sphere inside the cylinder obtained from CT scan

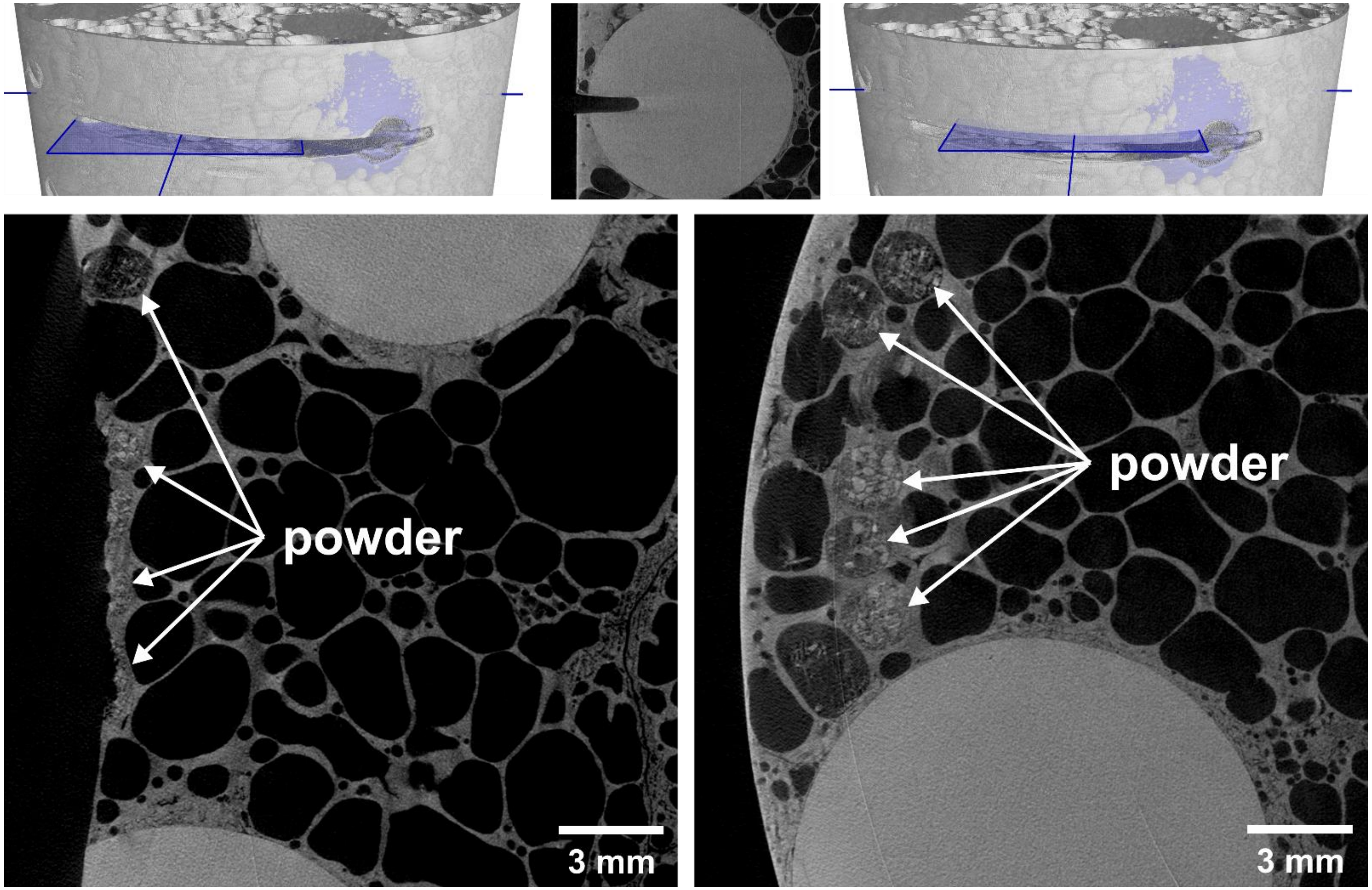

Fig. 6: Cracks inside the centrical cut sphere inside the cylinder obtained from CT scan

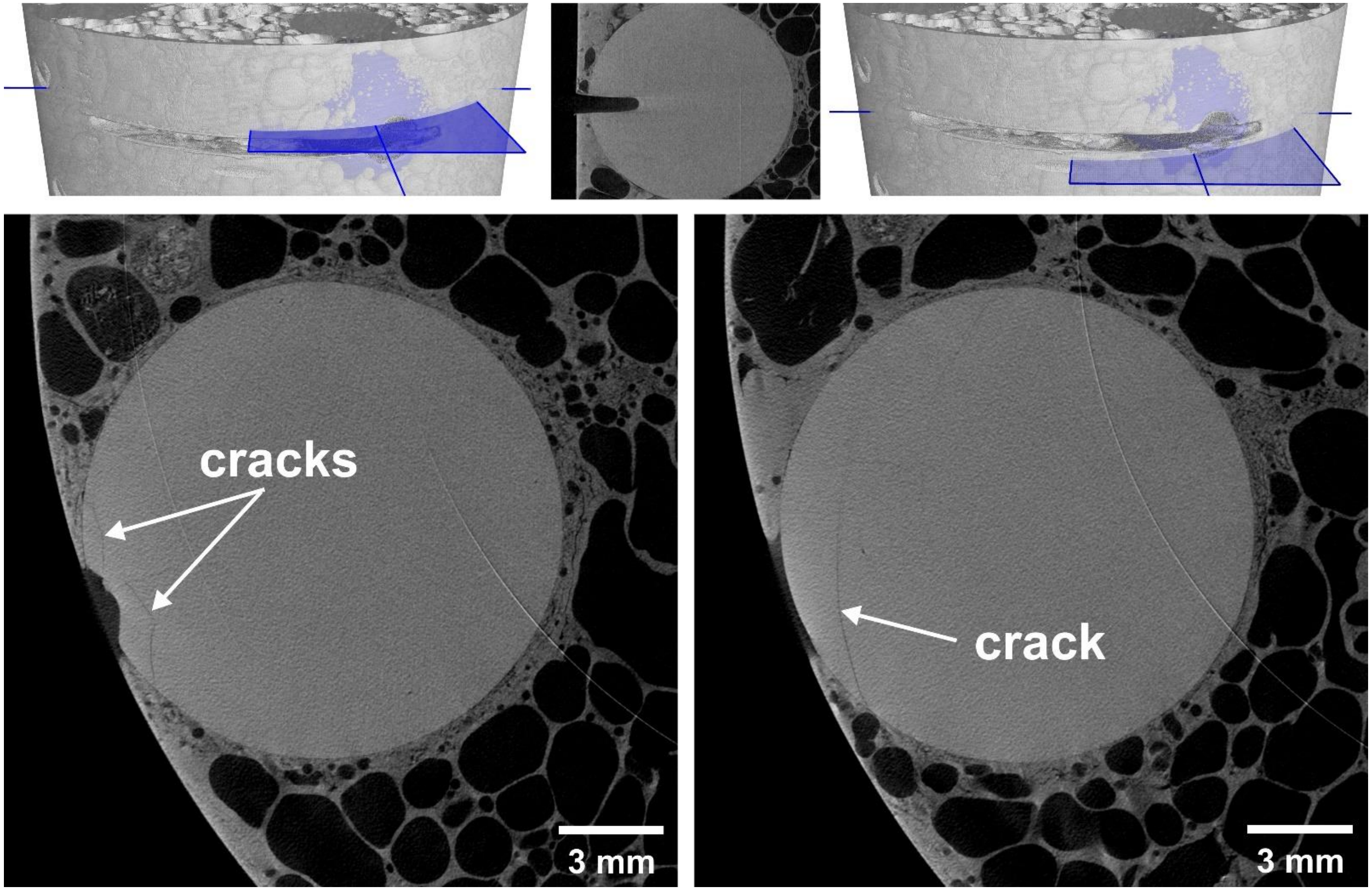

Fig. 7: 3D representation of sphere (blue), powder (red) and cracks (green) at the cut between two spheres inside the cylinder obtained from CT scan

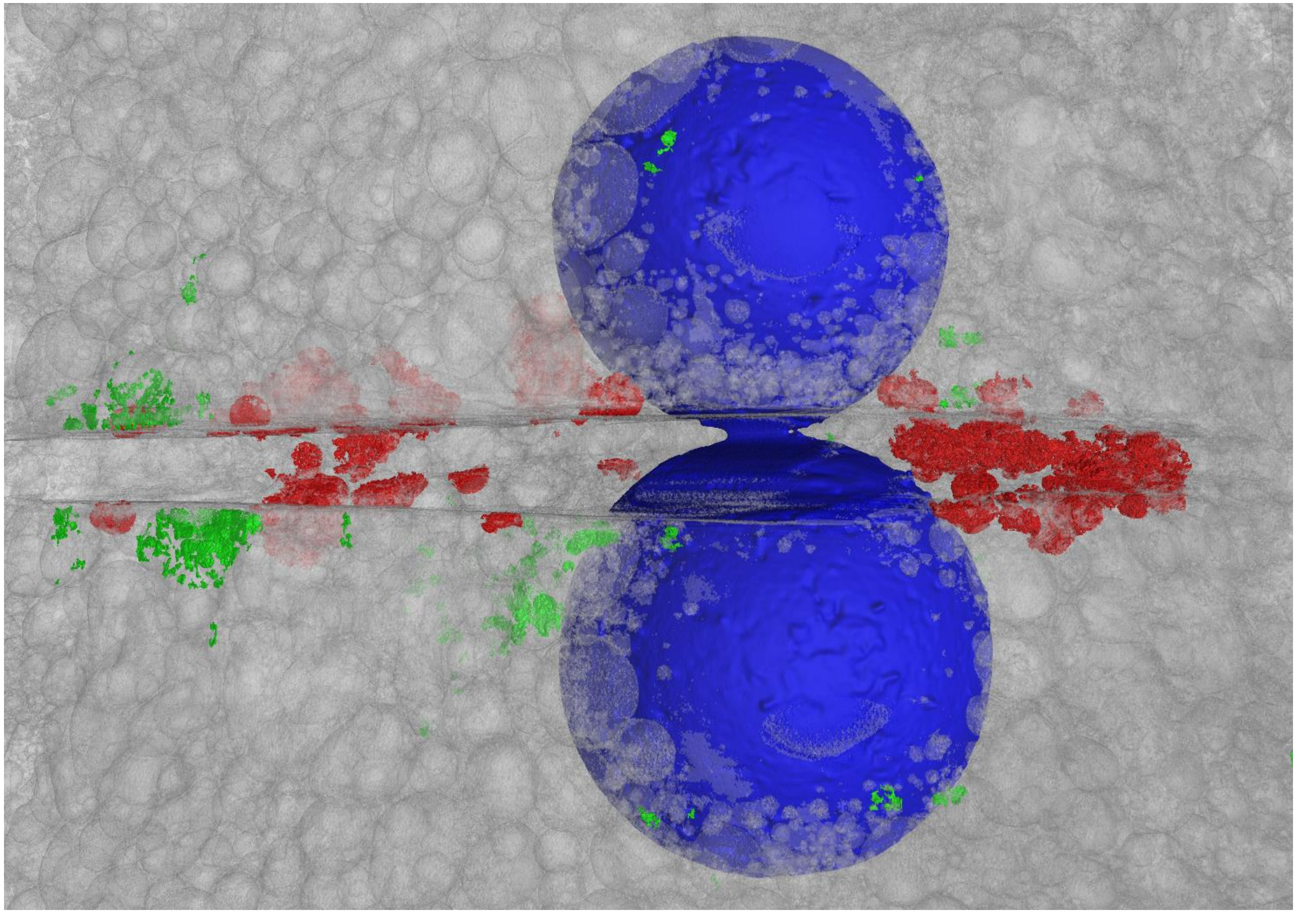

Fig. 8: Powder aggregation adjacent to the cut between two spheres inside the cylinder obtained from CT scan

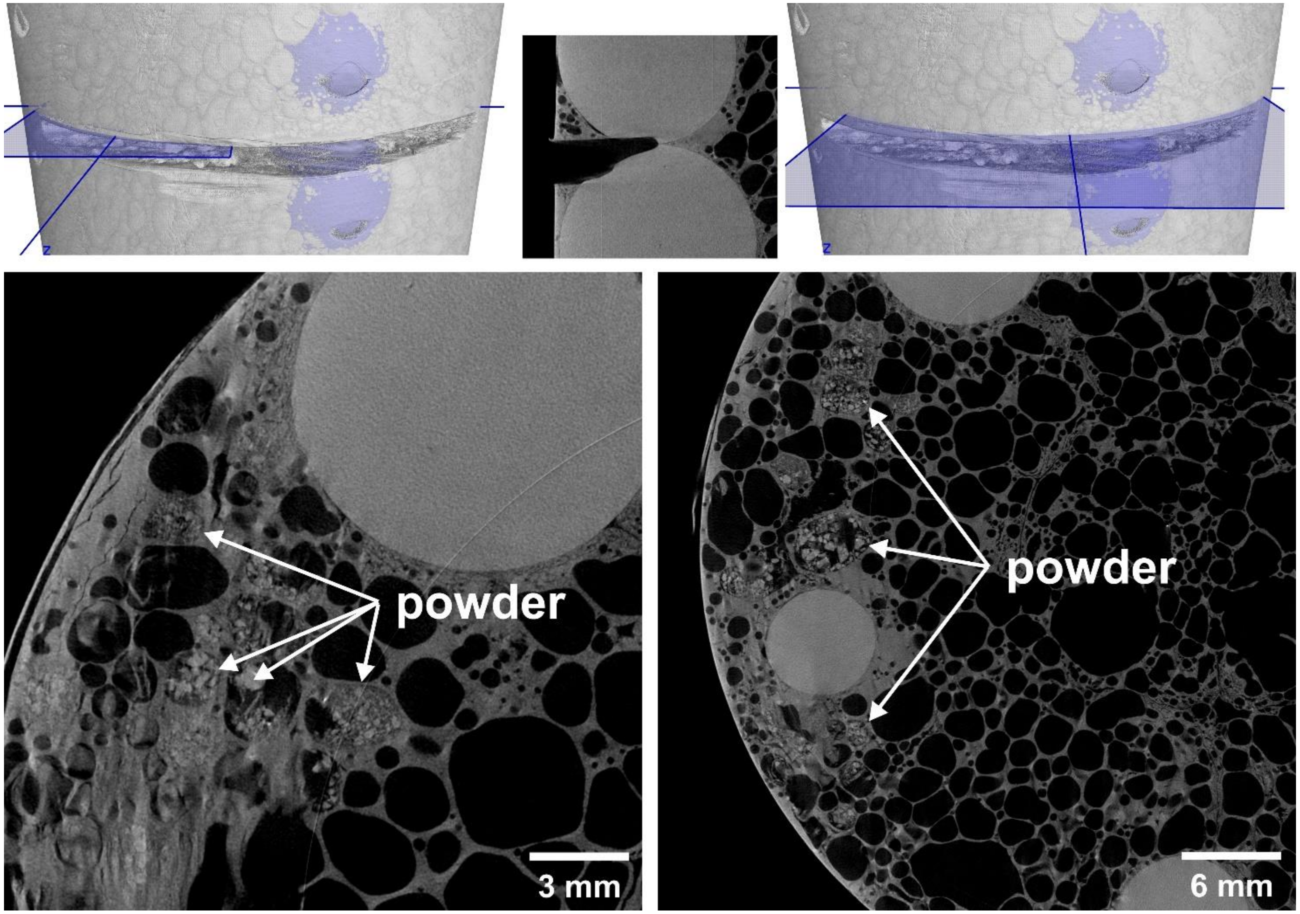

Fig. 9: Vibration-induced cracks inside the aluminium foam matrix adjacent to the cut between two spheres inside the cylinder obtained from CT scan

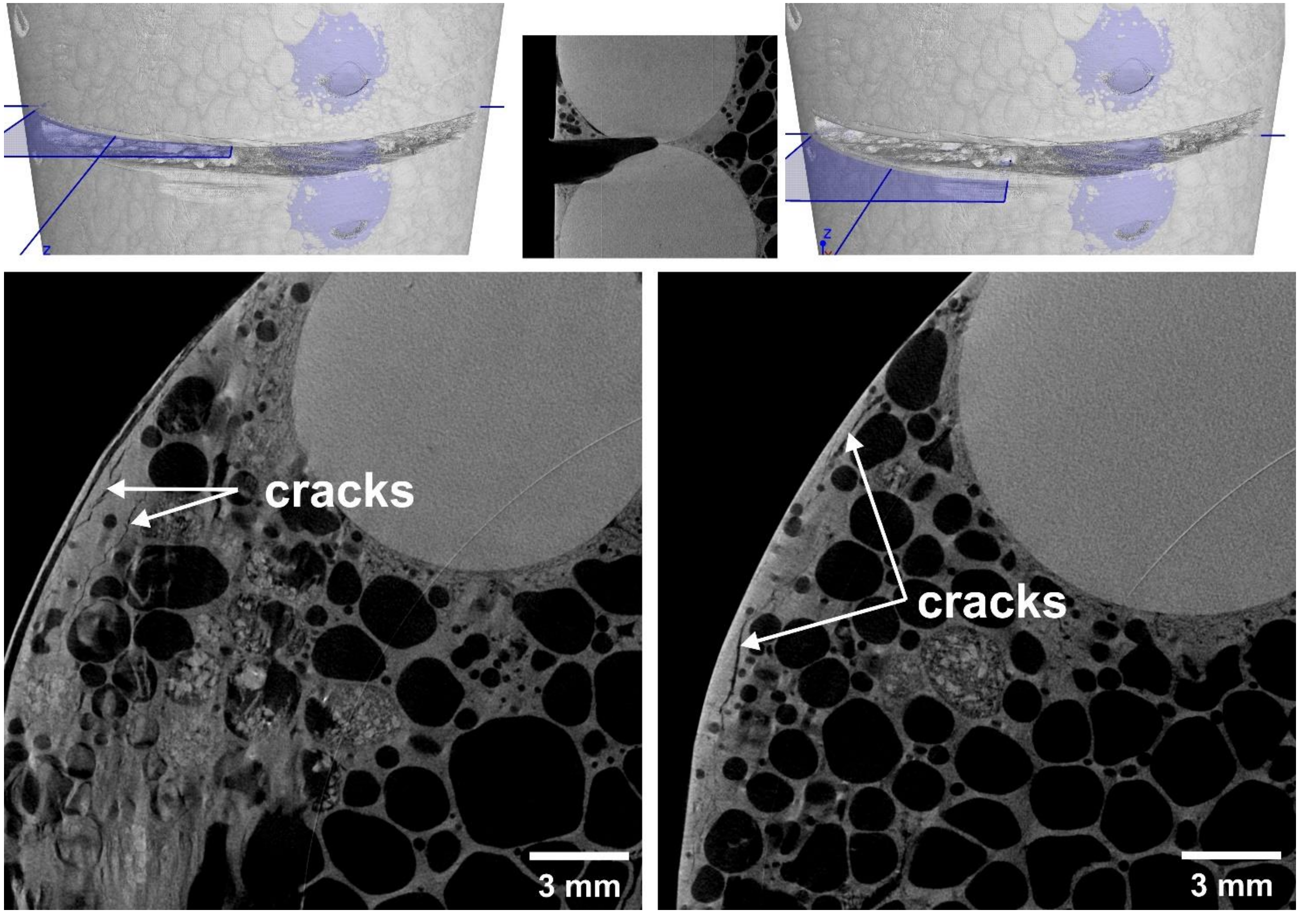

Fig. 10: Vibration-induced cracks inside the aluminium foam matrix adjacent to the cut between two spheres inside the cylinder obtained from CT scan

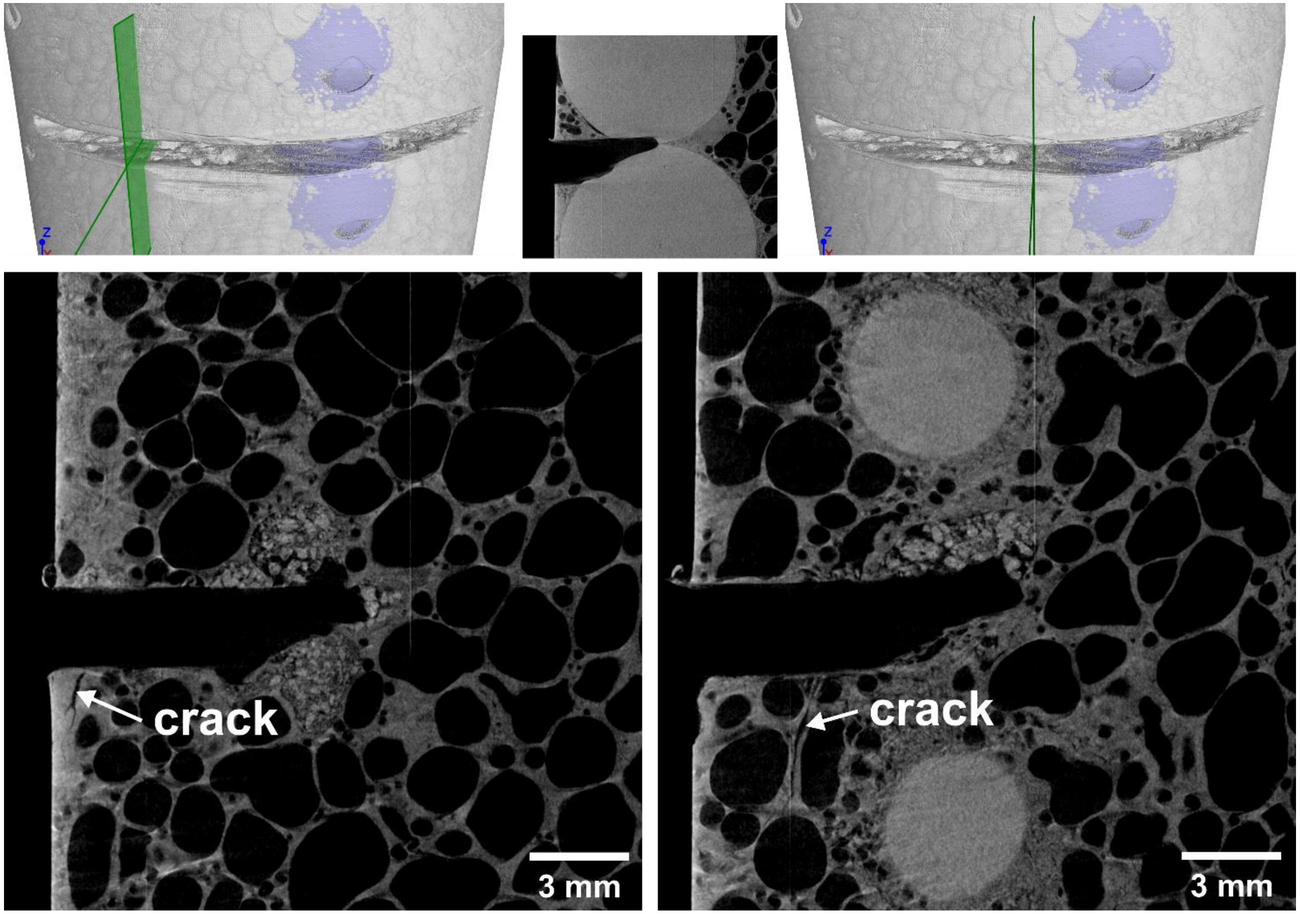

Fig. 11: 3D representation of sphere (blue), powder (red) and cracks (green) at the partial cut of a sphere inside the cylinder obtained from CT scan

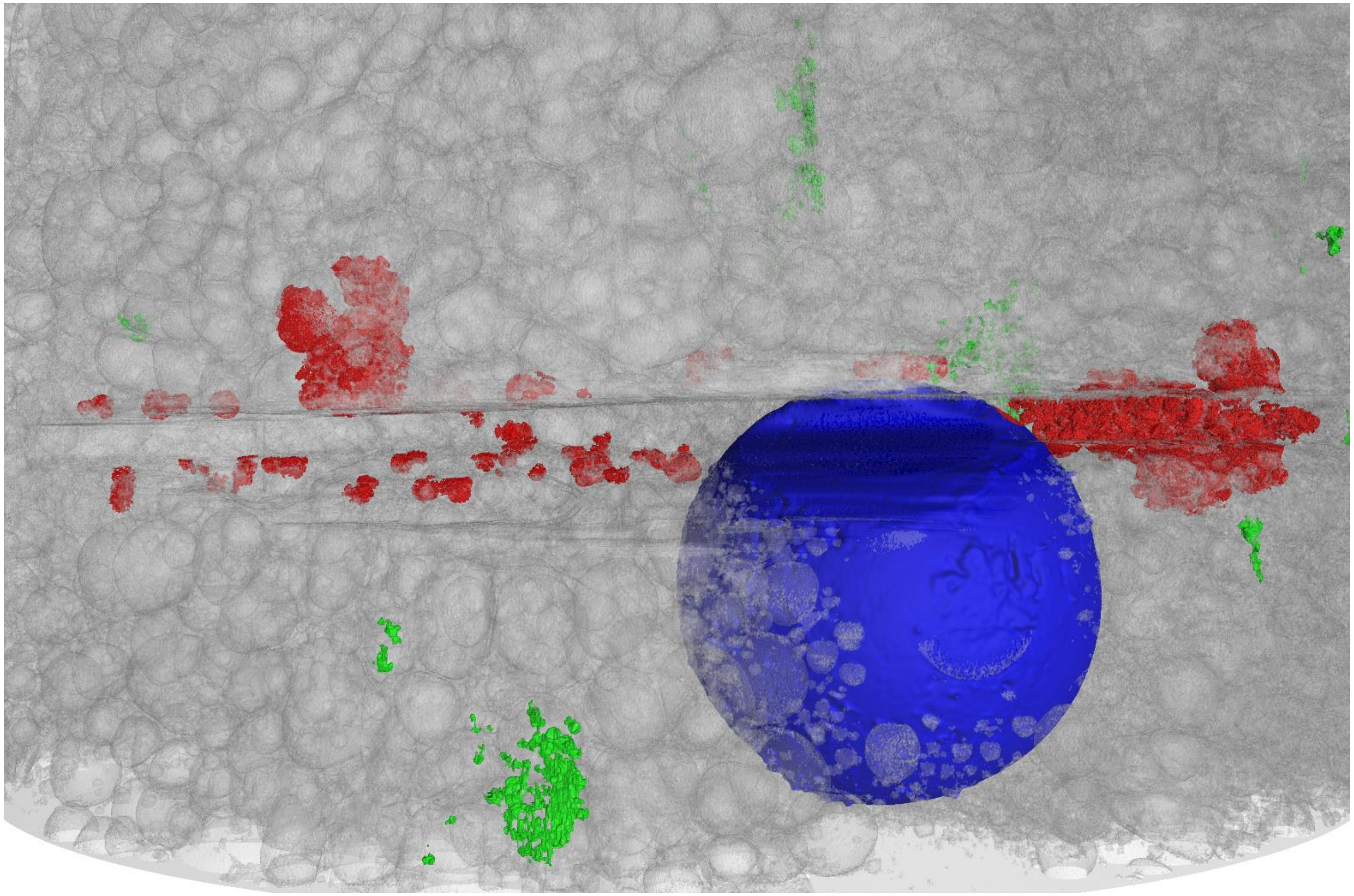

Fig. 12: Powder aggregation adjacent to the partial cut of a sphere inside the cylinder obtained from CT scan

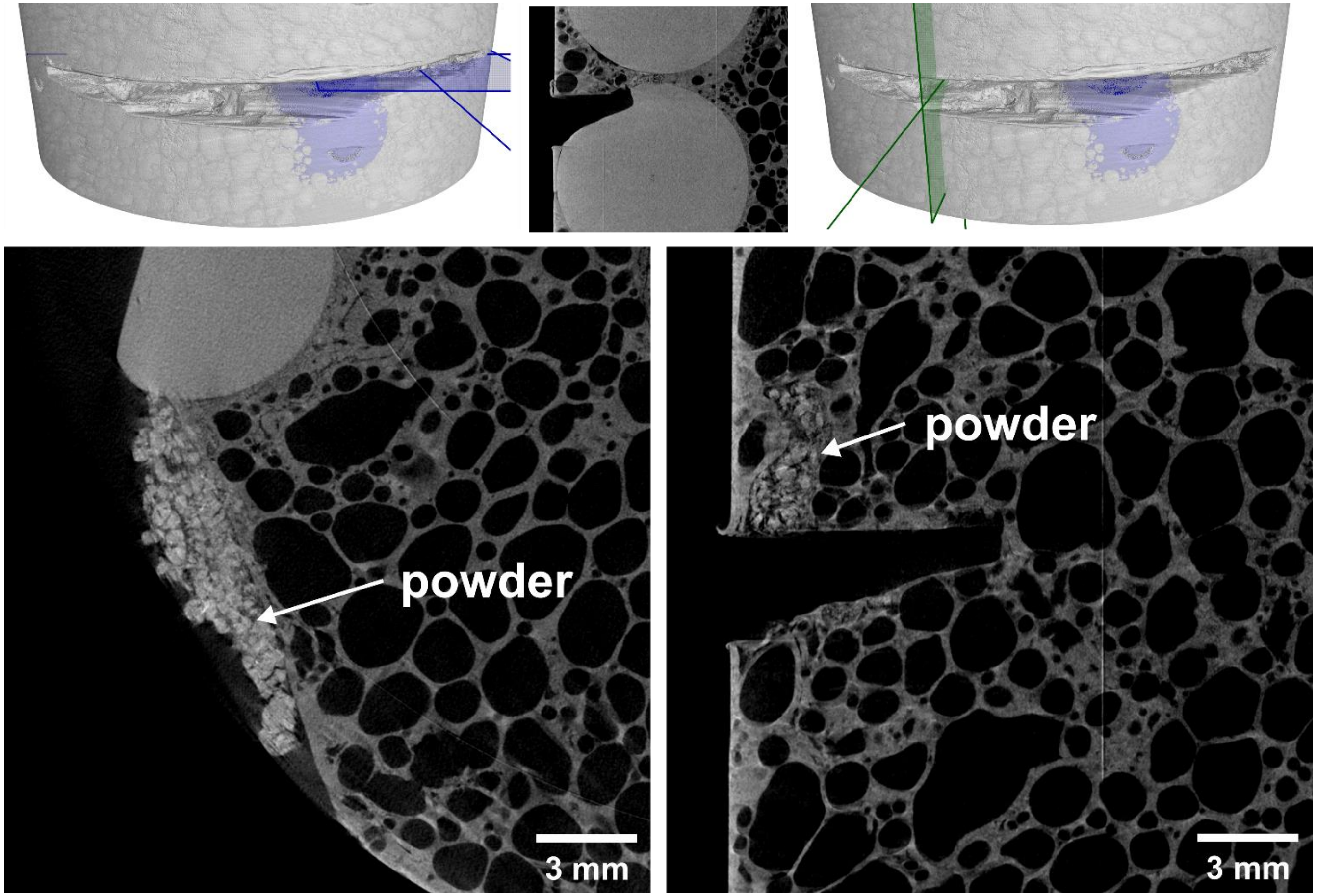

Supplement: Supplementary file 12 — Suppl_Information_A_CT. [file 41598_2020_65976_MOESM12_ESM.pdf]
