# Supplementary material for: Non-cuttable material created through local resonance and strain rate effects
Source: Sci Rep. 2020 Jul 20;10:11539. doi: 10.1038/s41598-020-65976-0 (PMC7371712; doi:10.1038/s41598-020-65976-0)
Supplement: Supplementary file 13 — Suppl_Information_B. [file 41598_2020_65976_MOESM13_ESM.pdf]

Durham University

# Non-cuttable material created through local resonance and strain rate effects

*Stefan Szyniszewski [1*], Rene Vogel [2], Florian Bittner [3,4], Ewa Jakubczyk[5], Miranda Anderson [6], Manuel Pelacci[5], Ajoku Chinedu [5], Hans-Josef Endres [3,4], Thomas Hipke [2]*

[1] Durham University, Durham, United Kingdom
[2] Fraunhofer Institute for Machine Tools and Forming Technology IWU, Chemnitz, Germany
[3] Fraunhofer Institute for Wood Research, Wilhelm-Klauditz-Institut WKI, Hannover, Germany
[4] Leibniz University Hannover, Institute of Plastics and Circular Economy IKK, Garbsen, Germany
[5] University of Surrey, United Kingdom
[6] University of Stirling, United Kingdom

## Supplementary Information B:
## Campbell, W. "Protection of Steam Turbine Disk Wheels from Axial Vibration."
## ASME Transactions 46, 31–160 (1924)

STEAM POWER

WATER TREATMENT FOR CONTINUOUS STEAM PRODUCTION, R. E. Hall.
THE INCREASE IN THERMAL EFFICIENCY DUE TO RESUPERHEATING IN STEAM TURBINES, W. E. Blowney and G. B. Warren.
A REVIEW OF RECENT APPLICATIONS OF POWDERED COAL TO STEAM BOILERS, Henry Kreisinger.
RECENT DEVELOPMENTS IN THE BURNING OF ANTHRACITE, W. A. Shoudy and R. C. Denny.

MANAGEMENT

SHOP MANAGEMENT, Frederick W. Taylor.
Presentation of Resolutions in Memory of Frank B. Gilbreth.
THE DEVELOPMENT OF A MODERN HOSIERY PLANT, Sanford E. Thompson and H. T. Rollins.

*Thursday Afternoon, December 4*

SIMULTANEOUS SESSIONS

MANAGEMENT AND MACHINE-SHOP PRACTICE

PRODUCTION CONTROL, George D. Babcock.
DESIGN, MANUFACTURE, AND PRODUCTION CONTROL OF A STANDARD MACHINE, Ralph E. Flanders.

OIL AND GAS POWER

SOLID-INJECTION OIL ENGINES, R. Hildebrand.
LARGE OIL ENGINES, WITH SPECIAL REFERENCE TO THE DOUBLE-ACTING TWO-CYCLE TYPE, Charles Edward Lucke.
GAS TURBINES, Lionel S. Marks and M. Danilov.

HYDRAULICS

A METHOD FOR THE ECONOMIC DESIGN OF PENSTOCKS, H. L. Doolittle.
INTAKES FOR POWER PLANTS, Robert W. Angus.

LECTURE

PROPERTIES OF MATTER UNDER HIGH PRESSURE, P. W. Bridgman.

*Thursday Evening, December 4*

PROGRESS CONFERENCE OF TECHNICAL COMMITTEES OF THE SOCIETY
CARNOT CENTENARY

No. 1920

# THE PROTECTION OF STEAM-TURBINE DISK WHEELS FROM AXIAL VIBRATION[1]

BY WILFRED CAMPBELL,[2] SCHENECTADY, N. Y.

Non-Member

*One of the most important features in the design and manufacture of a steam turbine is the elimination of the possibility of vibration occurring at the various natural frequencies of its disk wheels and buckets. This paper describes an investigation by the General Electric Company of various forms of vibrations and waves which may exist in steam-turbine disk wheels. The dangerous critical speeds that must be guarded against are discussed, together with other minor resonant conditions that it is advisable to avoid. The testing machines used for verification of predicted frequencies and critical speeds are described in detail as well as the different types of tests made. The conclusion gives the procedure, necessary in all cases, for the definite protection of steam-turbine bucket wheels from axial vibration, as justified by several years of successful manufacture.*

THE purpose of this paper is to present the main features of the work done by the General Electric Company (of America), which led to the solution of the problem of vibration of turbine disk wheels, and to describe the way in which wheels are designed and tested in order to insure freedom from vibration.

2 The investigation was undertaken in order to account for wheel failures of a peculiar and erratic nature which could not be explained on the basis of high stress alone. The number of failures was small, considering the total number of wheels in operation. These failures were not confined to any single type of machine, but they did show a preference in general for thin wheels of large diameter.

3 That this difficulty has actually been overcome with no major alteration in the turbine is emphatically brought out by results

[1] Prepared with the coöperation of A. L. Kimball, Jr., Assoc. A. S. M. E., and Ernest L. Robinson, Mem. A. S. M. E.

[2] General Electric Company. Deceased, July 7, 1924.

Presented at the Spring Meeting, Cleveland, Ohio, May 26 to 29, 1924, of THE AMERICAN SOCIETY OF MECHANICAL ENGINEERS.

obtained by the General Electric Company in the past three years from its use of disk wheels properly designed and tested.

TABLE 1 FOR TURBINES OF OVER 5000 KW. INSTALLED BEFORE MARCH 1, 1924

| | |
|---|---|
| Number of wheels installed | 4399 |
| Number of wheels tested (standing) | 3596 |
| Number of wheels rotated in wheel-testing machine | 320 |
| Number of tests in wheel-testing machine | 405 |
| Number of wheels tested in customer's plants (standing) | 1683 |
| Number of machines investigated in customer's plants | 291 |
| Number of machines tested under load in customer's plants | 24 |
| Number of wheels replaced to avoid possible trouble | 497 |
| Number of wheels tuned for vibration | 212 |

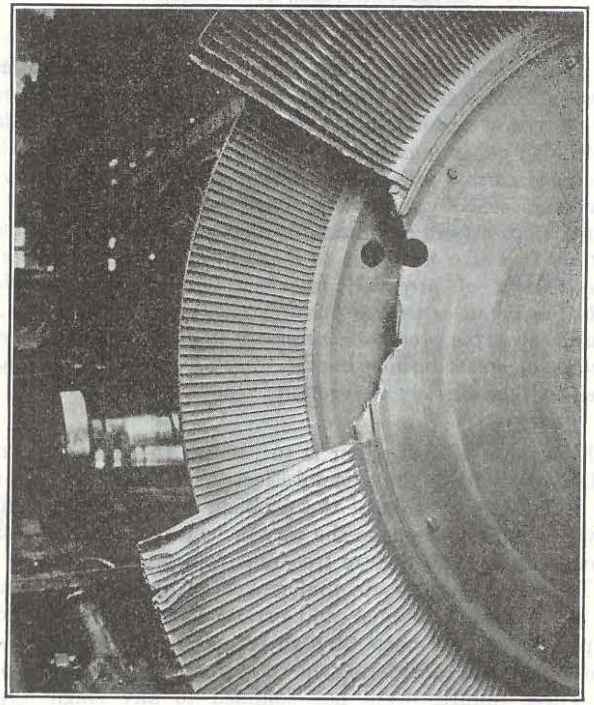

FIG. 1 BROKEN TURBINE BUCKET WHEEL; 9TH STAGE OF 15,000-KW. 1800-R.P.M. 9-STAGE TURBINE

4 Up to the end of 1923 this company had manufactured and installed over 9000 steam turbines aggregating in total generating capacity more than 15,000,000 kw. This investigation is chiefly concerned with large-size machines, that is, of over 5000 kw. capacity. Before the year 1919 the General Electric Company had manufactured and installed in operating plants, a total of 227 turbines of ratings exceeding 5000 kw. each. The total generating capacity represented by these machines was over 2,404,000 kw.



Since the year 1919, when this particular investigation was started, there have been 206 more turbines installed exceeding 5000 kw. each, increasing the total of generating capacity of the larger size turbines to 5,864,500 kw. at the end of 1923.

5 Table 1 shows the magnitude of the investigation, giving a few simple figures as to the number of machines and the number of turbine wheels investigated. The capacity of the testing machines now in operation is sufficient to provide for the testing of 600 wheels annually, under all conditions of speed.

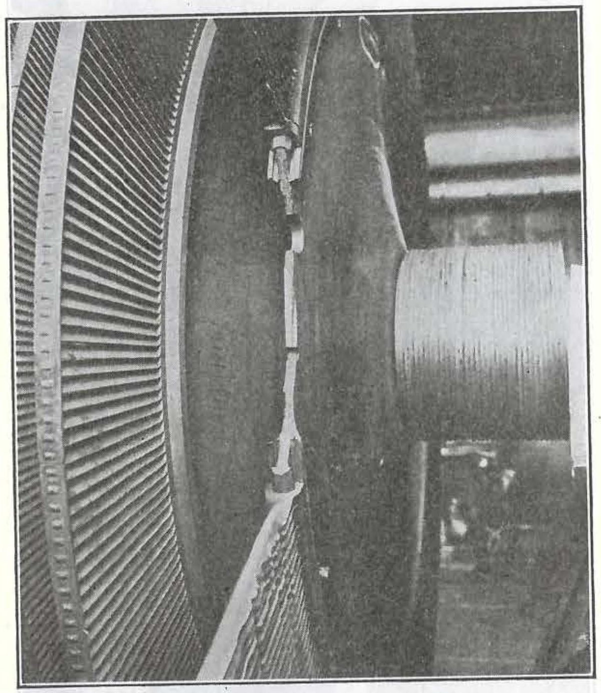

FIG. 2 DETAIL OF FRACTURE SHOWING CHARACTERISTIC FATIGUE FAILURE

6 That the methods developed are effective is attested by the successful elimination of all serious wheel and bucket troubles due to lateral vibration from the operation of recently built turbines.

## PART I — HISTORICAL OUTLINE

7 Before discussing in detail the nature of the vibrations to which disk wheels are subject, a brief narrative of the work of investigation will be presented. This seems necessary in order to give a proper perspective of the problem as a whole.

## Growth of Capacity

8 The design of turbines with one row of buckets per wheel took place long prior to the entry of America into the war. These designs used higher linear bucket velocities and were produced in unprecedented quantities during the war period. The rapid increases of turbine capacity which took place at the same time

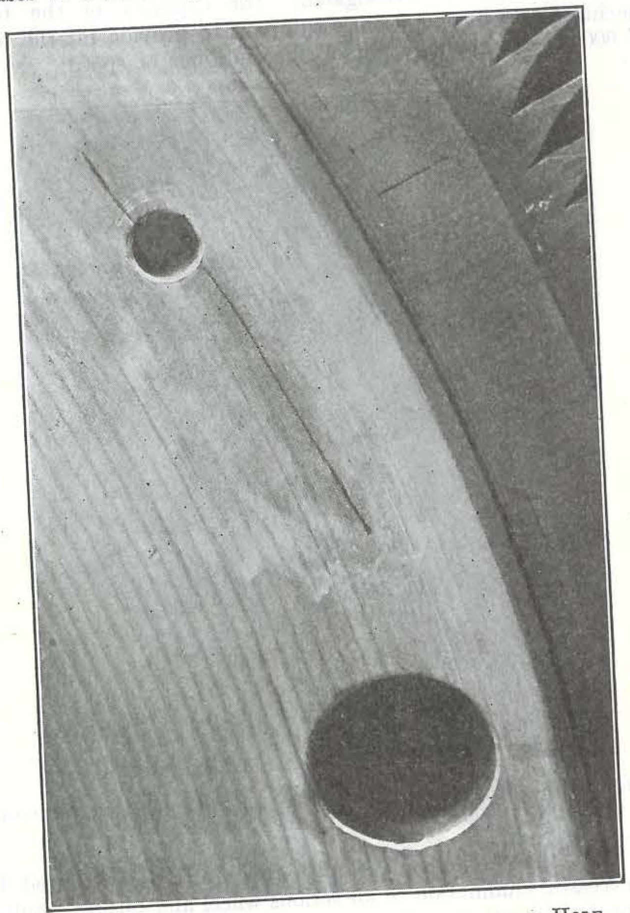

Fig. 3 Fatigue Crack Which Started at a Hole

were in a large degree accomplished by the use of the larger diameters introduced to give the greater bucket speeds, and by using longer buckets. The mechanical possibilities were pushed to the limit. While some improvements in steam conditions came at the same time, the important thing to note is that the real period of increased capacity due to improved thermal processes



occurred later and is still going on whereas, almost at one leap, the early designs were pushed to the limit from the point of view of structural strength.

9 Several principles of design pointed in the direction of light wheels. The maximum wheel stress is at the bore and this could be reduced by using lighter, thinner disks having less centrifugal bursting tendency. In fact, these were cut down in thickness as much as could be without creating a new maximum in the web due to the pull of the buckets. And not only did the desire

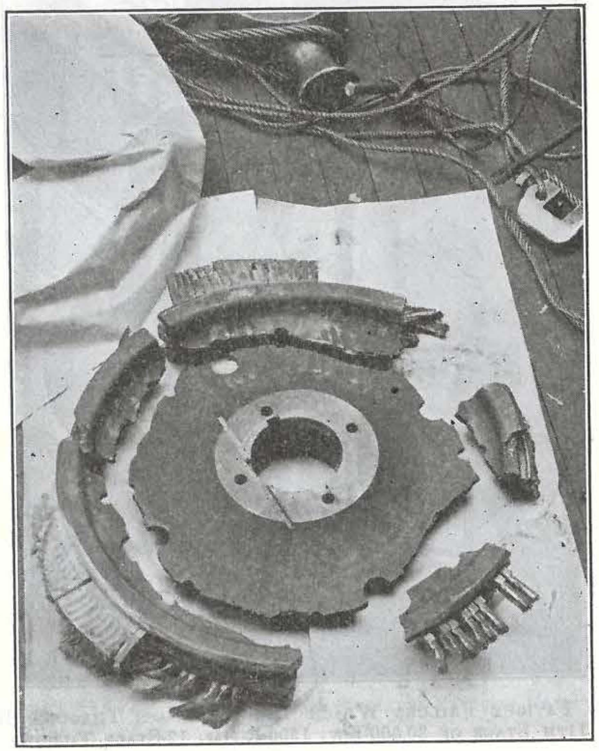

Fig. 4 Small Turbine Wheel Broken by Accidental Overspeeding. 2nd Stage of 500-Kw. 3600-R.p.m. 3-Stage Turbine

for conservative stresses point toward light wheels, but also the desire for a stiff rotor. Heavy wheels are accompanied by lower critical shaft speeds. The unquestioned advantage of a stiff shaft, when possible, also dictated light wheels. These various influences were perfectly natural at the time. The subject of vibration had not been brought to prominence. It was hazy and uncertain and no difficulties had been definitely connected with it. The plain path of reason seemed to be along the lines indicated.

### Types of Failures

10 In order to visualize the sort of difficulty which led to the present investigation, it will be well to examine a number of failures.

11 Figs. 1 and 2 show a break which originated in a small tapped hole and passed through a large steam balance hole. This was a vibration fatigue failure. An examination of the fractured surface shows the characteristic central line and progressive curves.

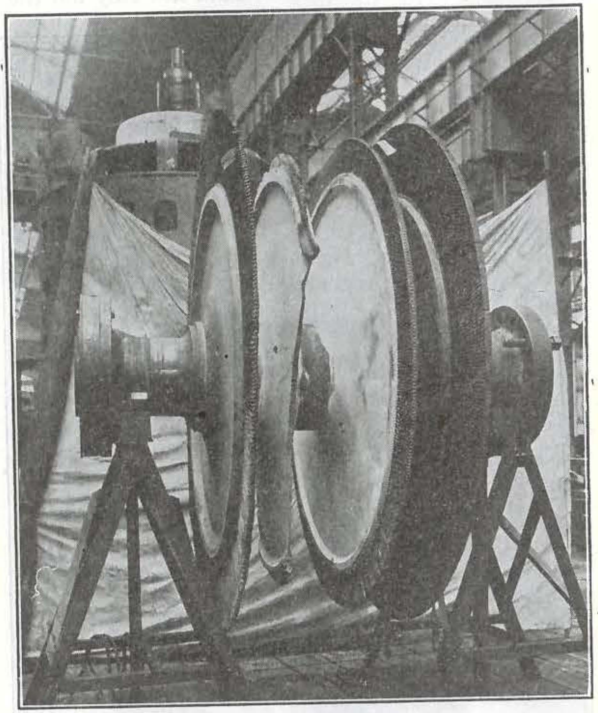

Fig. 5 Fatigue Failure Which Did Not Pass Through Holes. 11th Stage of 30,000-Kw. 1500-R.p.m. 12-Stage Turbine

Fig. 3 shows another crack discovered before complete rupture in the same wheel.

12 Fig. 4 shows a wheel which completely burst. Note that the line of fracture has passed through every one of the holes in the web of the wheel. This was due to accidental overspeed.

13 Figs. 5 and 6 show a typical fatigue fracture which did not originate at a hole.

14 Fig. 7 shows a break in which cracks originated at more than one hole. Figs. 8 and 9 show details of this fracture at each of the two holes. Although Fig. 7 bears a resemblance in its completeness of failure to Fig. 4, the type of fracture is entirely different and is characteristic of a fatigue failure.



15 In other cases the only damage was loss of buckets. Fig. 10 shows a wheel from which a number of buckets have been broken. In this case the breaks were due to axial vibration.

16 In Fig. 11 various failures occurring in the bucket dovetail are shown. In each case the marking indicates vibration in an axial direction.

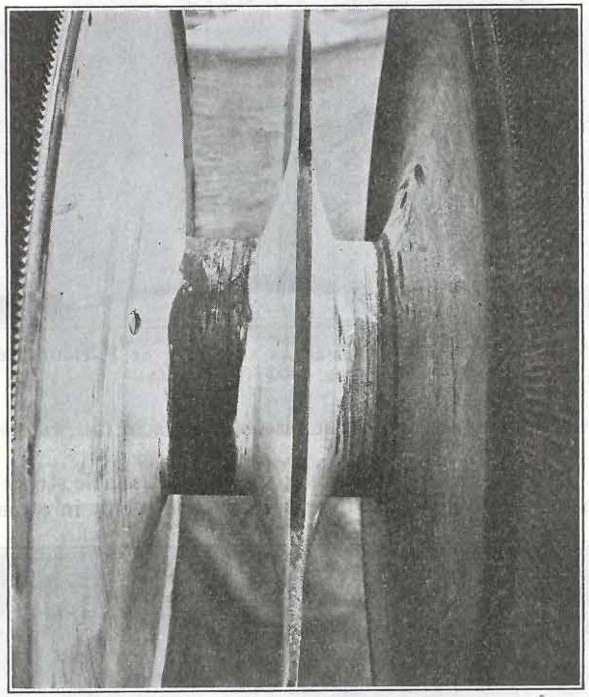

Fig. 6 Detail Showing Characteristic Fatigue Failure

17 Fig. 12 shows another class of turbine trouble in which a diaphragm has been scored in two diametrically opposite spots by a rubbing wheel. Fig. 13 is a section of the same diaphragm at the point of greatest rubbing together with a profile of the wheel showing the shape taken by all of the buckets.

18 These examples will serve to illustrate the various types of failure. Most of them were plainly due to vibration resulting in fatigue. A few were clearly the result of accidental overspeed such as Fig. 4. In certain cases the fractures avoided holes, but in general there was a distinct affinity for holes owing undoubtedly to the higher localized stresses around them.

### Hole Stresses

19 The first remedial measure used was to give immediate attention to the localized stresses in the neighborhoods of the steam balance holes and other discontinuities in the webs of the wheels.

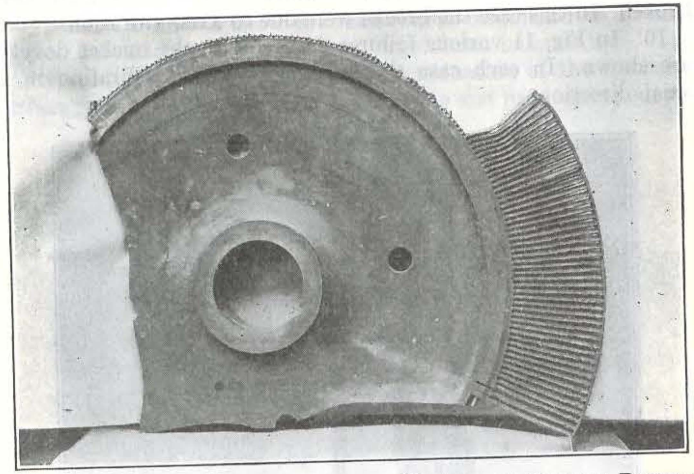

Fig. 7 Turbine Wheel Failure as a Result of Fatigue Bending. 3d Stage of 6000-Hp. Turbine

20 It is not the object of this paper to discuss hole stresses, but it is necessary to note that they become serious only in connection with vibration, by constituting a place for a fatigue crack to start. If a wheel is properly protected from vibration, there will be no repeated stresses at any point. Rather elaborate experiments have shown that the reinforcement of holes will serve to increase the resistance of a wheel to vibration in case it should be necessary to design with the expectation of fatigue stresses. But the greater safety lies in guarding against the stresses themselves by the precautionary measures developed for use in manufacture.

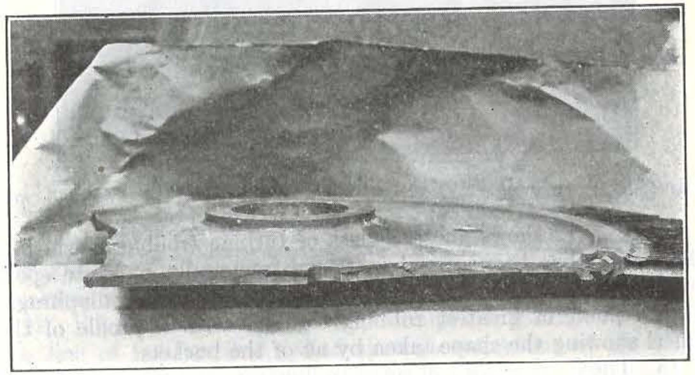

Fig. 8 Detail Showing How the Crack Started at One Hole



### Bucket Lacing

21 The early experiments on the vibration of buckets indicated that a lacing wire paralleling the shroud band and connecting different groups of buckets would remove various secondary vibrations. Although the fundamental period was not much influenced this expedient was actually used in a number of turbines. The

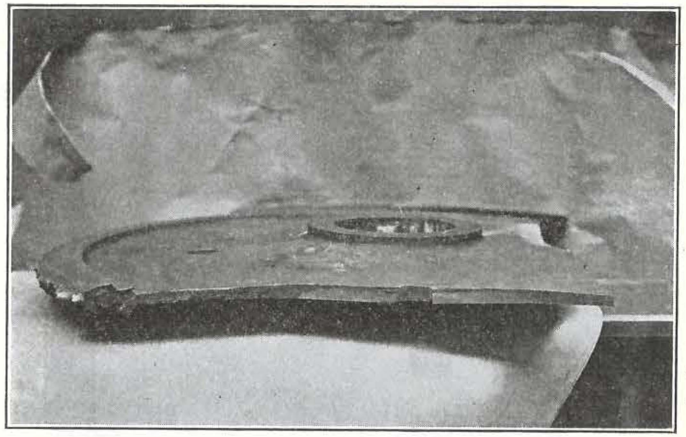

Fig. 9 Detail Showing How the Crack Started at Another Hole

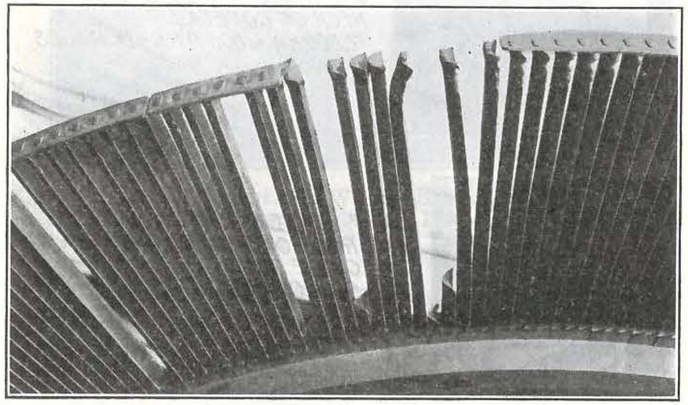

Fig. 10 Roots of Buckets Broken by Fatigue Bending Due to Vibration. 12th Stage of 10,000-Kw. 1500-R.p.m. 12-Stage Turbine

lacing wire, however, failed to remove the cause of the trouble and became itself another hazard. During these experiments a taut wire attached to the end of each bucket raised the frequency, thus illustrating the similar effect due to centrifugal force.

## The Idea of Wave Motion

22 About the same time certain types of vibration of standing wheels were investigated by means of sand pictures. The usual

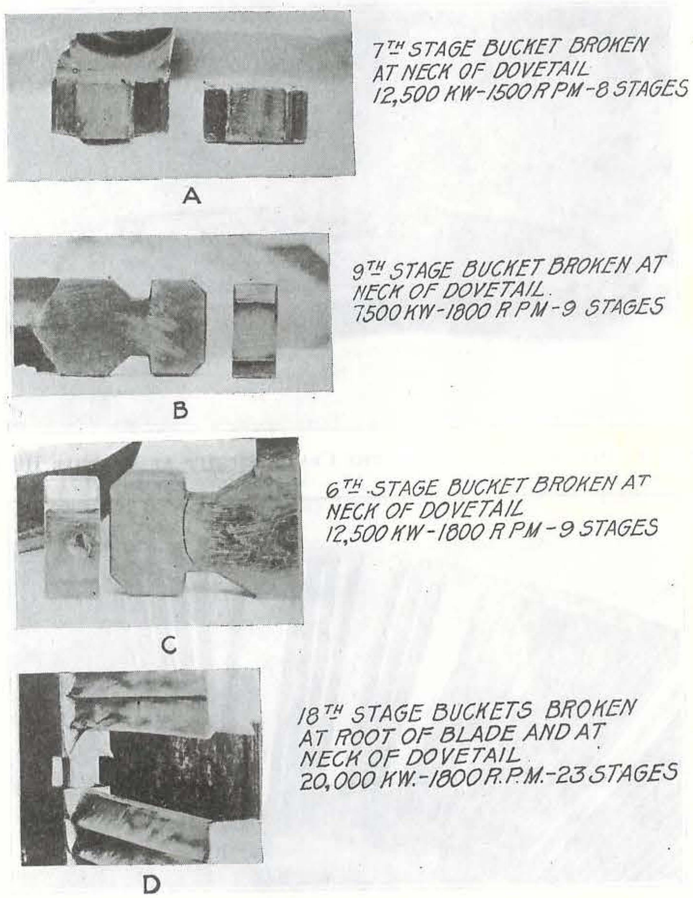


Fig. 11 Details of Bucket Failure Due to Vibration

form was a series of vibrating segments symmetrically arranged about the circumference and extending into the web but separated by radial lines of quiet called nodal radii or nodes. These will be discussed in detail presently.



## Windage Theory

23 In connection with the study of strains about holes in wheel webs, a series of india-rubber wheels as shown in Fig. 14 had been made and photographed by means of instantaneous electric sparks while rotating at high speed. In order to produce representative stresses throughout the rubber disks, metal weights were attached about the circumference, shaped to simulate the loading due to turbine bucket blades.

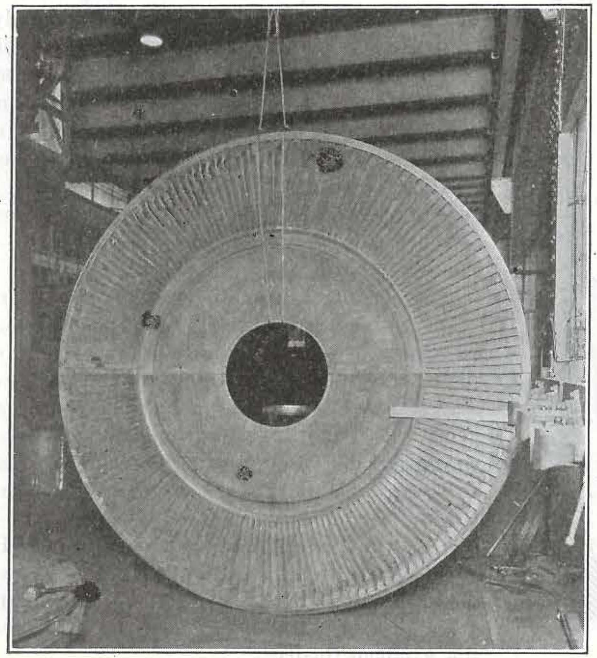

Fig. 12 Turbine Diaphragm Which Has Suffered Rubbing in Two Diametrically Opposite Regions. 17th Stage of 30,000-Kw. 1800-R.p.m. 17-Stage Turbine

24 In running these wheels it was discovered that above certain definite speeds their circumferences developed a form of wave motion as shown in Fig. 15. This was examined by means of an intermittent spark either synchronized with the speed of rotation or adjusted to occur at slightly greater or less frequencies. The shapes of the waves and their rates of progress were thus examined visually, and it was found that these waves progressed around the wheel in the direction of rotation, but at a less speed than the speed of the wheel; that is, relative to the wheel itself the wave was traveling backward, seemingly driven by the windage encountered, like the fluttering of a flag.

25 This gave rise to the so-called windage theory that the waves were developed and driven backward in the wheel by the atmosphere in which it was revolving. Investigation of this theory led to the rotation of paper disks in a vacuum. The wave motions, clearly observable in an atmosphere, disappeared as the atmosphere became rarified. These experiments were immediately extended to exceedingly thin steel disks which were found to behave in a similar manner. Since the waves could not exceed the speed of the actuating wind without encountering resistance, they could only be supported in wheels whose natural frequencies corresponded to waves traveling at less speed than the speed of rotation. It was therefore made a condition of design of turbine wheels that the natural wave speed should exceed the speed of rotation wherever possible. This resulted in a general thickening of all wheels being designed for turbines constructed at that time so as to give them a greater rigidity to withstand the supposedly detrimental effect of the wind action.

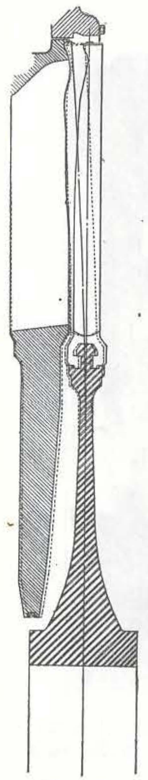

Fig. 13 Showing Depth of Rubbing at Worst Parts of Fig. 12

26 Subsequently, oscillograph coils were placed in two large turbines during operation and complete survey of these machines showed that several stages developed wave phenomena of the same general characteristics. Turbine wheels were thus shown capable of supporting traveling waves. In these turbines the waves appeared only when the turbine carried more than a certain definite load and died out when the load was removed. However, waves were found to be traveling in the wheel in a direction opposite to its rotation and at a higher speed, which could not be explained by the windage theory.

## Wave-Phenomena Records Made from a Thin Steel Disk

27 The first oscillograms taken in which a revolving coil was used were made with a thin sheet-metal disk. Fig. 16 is typical of the type of oscillograph record obtained. The upper curve $A$ is taken by a coil stationary in space and opposite the rim of the disk; it shows that there is a wave motion in the disk. The smoother portion of the curve corresponds to the part where the wheel disk is most remote from the coil and the more disturbed portion indicates that the wheel rim is in close proximity. These more disturbed portions, which may be called beats, occur at a much slower rate than the speed of rotation so that the corresponding wave crest producing them must have progressed around in the disk itself.



28 Curve $B$ is taken by a coil made to revolve with the wheel but mounted on a separate arm. When this coil passed near the supporting pillow block a voltage was induced which made the long narrow lines in the curve, there being one of these for each revolution. The V-shaped points in this record show that a transverse motion is taking place in the wheel disk, resulting in change of clearance between the disk and the revolving coil. It can be shown that this is not in any sense synchronous with revolutions but simply records a transverse vibration of the wheel itself.

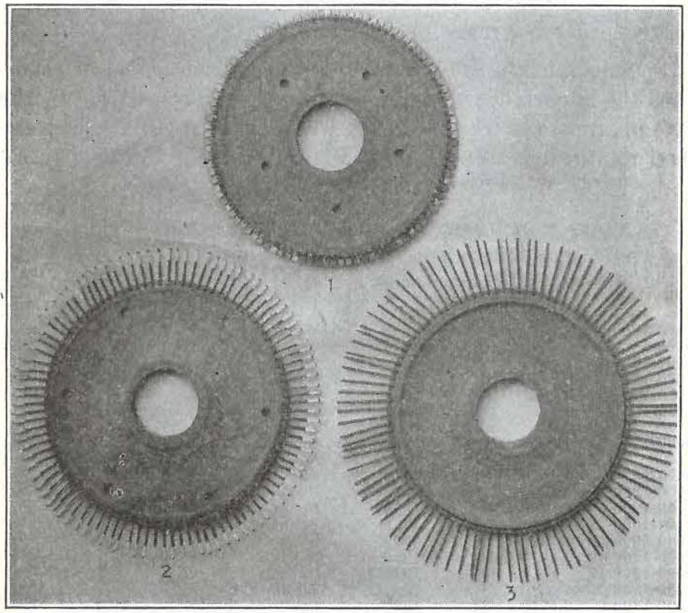


Fig. 14 India-Rubber Wheels Used to Examine the Strains about Holes in Web

29 Curve $C$ is taken from the 40-cycle a.c. line and is here used as a timing wave. The small interruptions in this wave are made by a contact on the shaft carrying the model steel disk and they correspond, therefore, to revolutions of the disk.

30 The record shown in Fig. 17 was made by a thin steel disk revolving slowly. This disk was vibrated by means of an alternating-current magnet carried on a rotating arm at the same

speed. The upper record *A* was obtained by a fixed coil while the lower record *B* is a 40-cycle timing wave. The irregularities in the record obtained by the stationary coil are caused by the passage of the rotating exciting magnet of which, it will be noted, the electrical frequency is one-half the mechanical frequency. This record, briefly, shows wheel vibration of the 6-node type including both forward and backward waves, which will be explained in the next part of this paper. This record is reproduced in the 5th edition of Stodola's Steam Turbines.

### Recognition of Wheel Critical Speeds

31 Subsequently Table 2 was compiled which definitely established the importance of the wave stationary in space, that is, the wave progressing backward in the wheel at the speed at which the wheel rotates forward. This speed is called a *wheel critical speed.*

TABLE 2 SUMMARY OF WHEEL AND BUCKET TROUBLES

| Rating | Stage | Year trouble occurred | Trouble | Nodes | Backward speed of wave r.p.s. | Operating speed r.p.s. |
|---|---|---|---|---|---|---|
| 35000-1500-20 | 19 | 1918 | Wheel | 4 | 25.6 | 25 |
| 15000-1800-9 | 9 | 1918 | Wheel | 4 | 27.9 | 30 |
| 15000-1800-9 | 9 | 1919 | Wheel | 4 | 29.5 | 30 |
| 15000-1800-7 | 3 | 1920 | Wheel | 6 | 28.5 | 30 |
| 3000-Variable-4 | 3 | 1921 | Wheel | 6 | 48.1 | 48 |
| 30000-1500-12 | 11 | 1921 | Wheel | 4 | 25.2 | 25 |
| 20000-1800-12 | 10 | {1917, 1919, 1920} | Buckets | 8 | 30.2 | 30 |
| 30000-1800-17 | 13 | 1918 | Buckets | 8 | 30.1 | 30 |
| 30000-1800-17 | 17 | 1918 | Buckets | 4 | 32.6 | 30 |
| 45000-1200-21 | 21 | 1918 | Buckets | 4 | 19.8 | 20 |
| 7500-1500-8 | 2 | 1919 | Buckets | 6 | 25.4 | 25 |
| 5000-3600-5 | 2 | 1919 | Buckets | 4 | 57.4 | 60 |
| 30000-1800-17 | 11 | 1920 | Buckets | 8 | 29.6 | 30 |
| 30000-1800-17 | 12 | 1920 | Buckets | 6 | 28.8 | 30 |
| 15000-1800-23 | 21 | 1920 | Dovetail | 4 | 29.5 | 30 |
| 10000-1800-9 | 8 | 1921 | Buckets | 8 | 30.6 | 30 |
| 35000-1500-22 | 17 | 1921 | Buckets | 6 | 24.2 | 25 |
| 30000-1500-20 | 14 | 1921 | Buckets | 8 | 25.9 | 25 |

In making a statement of the importance of this phenomenon, it should not be assumed that it is the only type of wheel vibration of a serious nature. This type of vibration has caused by far the largest number of failures, in fact, so large a fraction that breaks caused by other types of vibration may fairly be treated as exceptional.

32 At this time the importance of obtaining test data on full-sized wheels under actual operating conditions was first fully appreciated. This resulted in the design and construction of the first wheel-testing machine.



## PART II — EXPOSITION OF THE NATURE AND THEORY OF VIBRATION IN TURBINE WHEELS

33 To illustrate standing vibrations in turbine disk wheels, the following method was used. A turbine wheel was mounted in a horizontal position on a stub shaft. An electromagnet was clamped with its poles close to the edge of the wheel. On passing an alternating current through the coils of this magnet a series of pulls

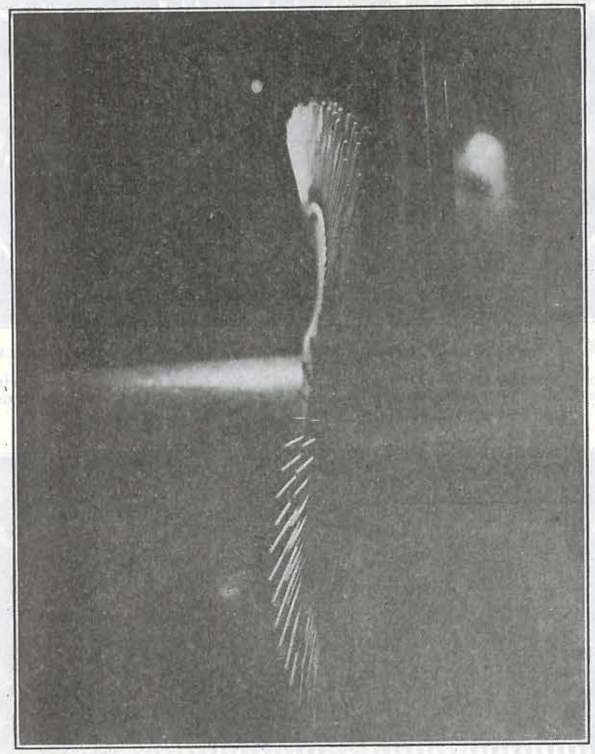

Fig. 15 Photograph by an Instantaneous Spark of an India-Rubber Wheel Exhibiting Wave Phenomena at a Speed of 650 R.p.m.

was exerted on the wheel tending to deflect it in a direction transverse to the plane of the disk. The frequency of these pulls is twice the frequency of the alternating current used because every complete electric cycle corresponds to two current pulsations in the magnet, and an electromagnet exerts a pull when current flows in either direction through the coil. The alternating-current generator was driven by a variable-speed direct-current motor by means of which the frequency of the magnet pull could be varied over a wide range.

## Sand Pictures

34 Sand was scattered over the wheel surface and the frequency of the magnetic pulls was varied until a particular frequency was reached at which the wheel responded. Fig. 18 shows a case where the wheel vibrated in four segments. In this

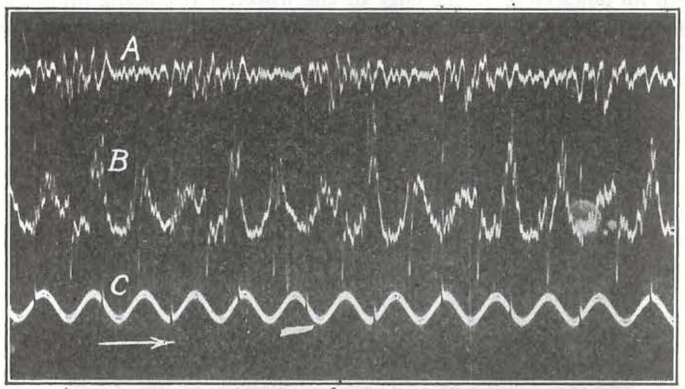


Fig. 16 One of the First Oscillograms Recording Disk Vibration

(Trace *A* was recorded by a stationary coil, Trace *B* by a coil revolving with the disk, and trace *C* is a 40-cycle timing wave. Model disk wheel of thin sheet metal.)

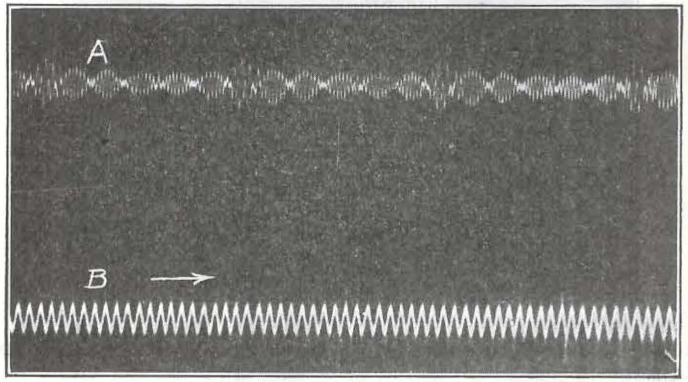


Fig. 17 Record of 6-Node Vibration in a Thin Steel Disk Excited by an A. C. Magnet Revolving with the Wheel

(Trace *A* was recorded by a stationary coil. Trace *B* is a 40-cycle timing wave.)

vibration each segment springs up and down, scattering the sand over to the quiet or nodal zones where there is no up-and-down motion. If, however, the frequency of the deflecting pulls of the magnet is altered even a very small amount the vibration immediately dies out, although the magnitude of the impulses of the



magnet remains the same as before. On raising the frequency of the magnetic pulls another point is found at which the wheel responds. It vibrates in segments, as before, but with 6 nodal radii or nodes equally spaced around the wheel circumference instead of four.

35 Figs. 19 and 20 illustrate a 6-node vibration and show that its location is not necessarily dependent on the position of a series of symmetrical discontinuities such as the steam balance

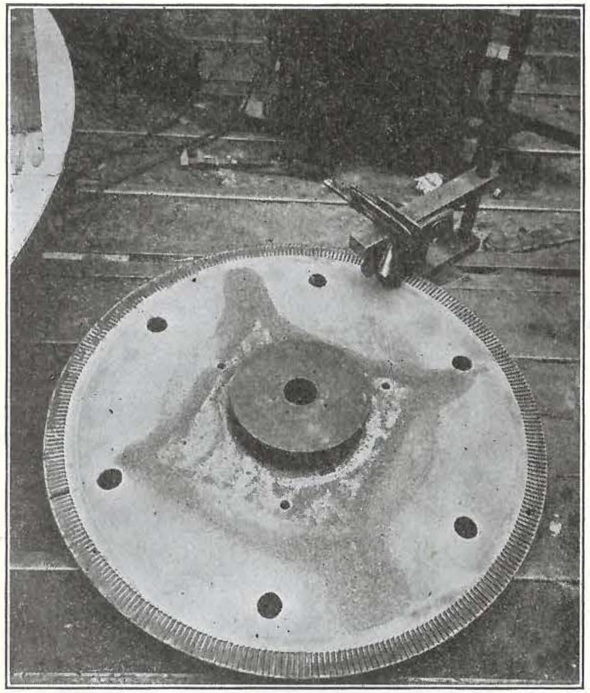

Fig. 18 4-Node Sand Picture Made by Vibration of a Wheel with Short Buckets

holes. Not only does the disk wheel respond when the pull frequency corresponds to four or to six radial nodes, but it may respond readily to frequencies corresponding to 8, 10, 12, or even a larger number of nodes, the number of nodes always being even because for every segment which springs upward during a vibration, the segment next to it on the other side of a nodal line must spring downward. These photographs illustrate the case of a small wheel with short buckets.

36 Figs. 21, 22, 23 and 24 show cases of 4, 6, 8 and 10 nodal vibrations for the case of a disk wheel carrying long buckets, the

total diameter of wheel and buckets being over 8 ft. This wheel was photographed with a layer of paper on the buckets to hold the sand. In the cases of four and six nodes it is seen that the regions of amplitude large enough to move the sand do not extend so deeply into the wheel as in the wheel with short buckets, while in the cases of eight and ten nodes the sand figure is confined to the bucket zone entirely.

37 The following general observations may be made on this type of vibration in which segments around the edge of the wheel

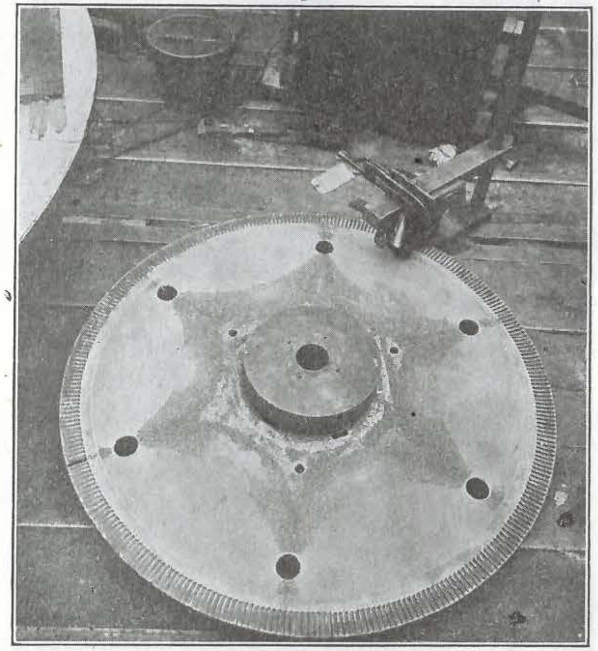

Fig. 19 6-Node Sand Picture Made by Vibration of a Wheel with Short Buckets

spring up and down, being separated from each other by radial nodal lines:

1 Every disk wheel responds readily to vibrations of four, six, eight, etc. radial nodes, each type of vibration having its own characteristic frequency.

2 The higher the number of nodes the higher the frequency of the vibration and the less easily is the vibration excited.

3 The higher the number of nodes the more difficult it is to force the sand figures towards the center of the disk.



4 Both the disk wheel and the buckets vibrate together as a continuous disk and must be treated as a unit in this type of vibration.

38 Vibrations may also take place with two nodes, as will subsequently be discussed. This type exerts a couple on the shaft transverse to its length, while the types described are balanced in their reactions on the shaft.

39 Many other types of vibration exist, including concentric ring nodes and combinations of ring and radial nodes. A hybrid

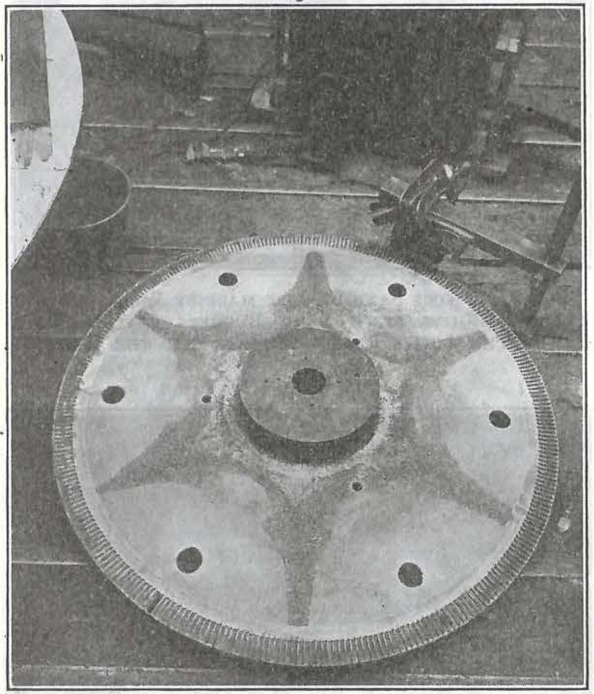

Fig. 20 6-Node Sand Picture Showing Independence of Pattern from Hole Location. Compare with Fig. 19

form resulting from a combination of six- and twelve-node radial types is shown in Fig. 25. These types of vibration are not readily excited and do not enter into this discussion, because they have not been found to be the cause of serious trouble.

## Effect of Centrifugal Force on Vibration Frequency

40 After the natural vibration frequencies of a turbine disk wheel when not rotating are determined as described, a question which arises is the effect upon these vibration frequencies of

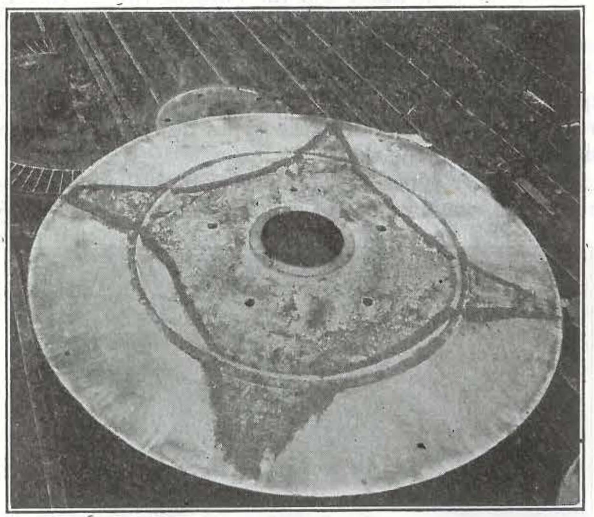

FIG. 21 4-NODE SAND PICTURE MADE BY VIBRATION OF A TURBINE WHEEL WITH LONG BUCKETS COVERED WITH PAPER. THE ACTIVE REGION EXTENDS INTO THE WHEEL

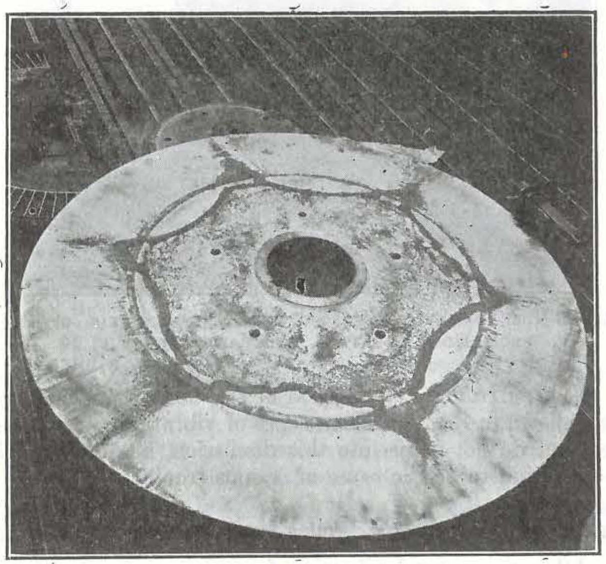

FIG. 22 6-NODE SAND PICTURE MADE BY VIBRATION OF A TURBINE WHEEL WITH LONG BUCKETS COVERED WITH PAPER



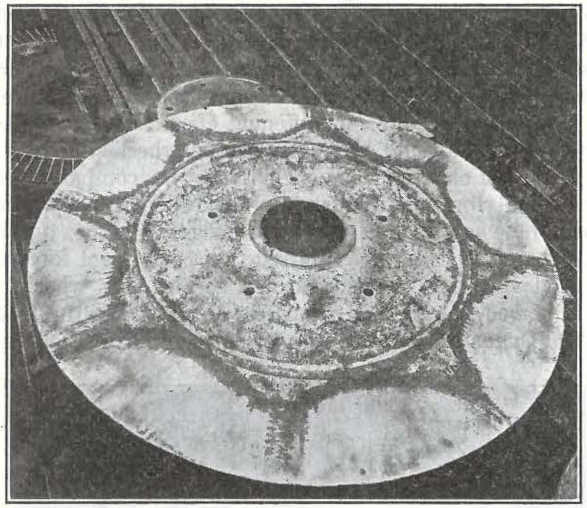

FIG. 23 8-NODE SAND PICTURE MADE BY VIBRATION OF A TURBINE WHEEL WITH LONG BUCKETS COVERED WITH PAPER

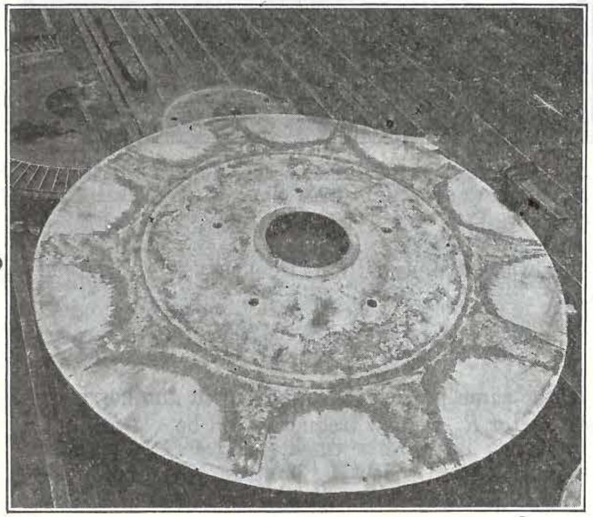

FIG. 24 10-NODE SAND PICTURE MADE BY VIBRATION OF A TURBINE WHEEL WITH LONG BUCKETS COVERED WITH PAPER. THE ACTIVE REGION IS CONFINED TO THE BUCKETS

rotation of the wheel at high speed. The frequency of a given type of vibration is determined by two factors, (*a*) the stiffness and (*b*) the mass of the vibrating body. The stiffer the body the faster it vibrates, and the more massive it is the slower will it vibrate. Now centrifugal force has no effect on the mass of the wheel, but it has a powerful stiffening effect. This force acting radially outward around the edge of the wheel stiffens it and raises its vibration frequency. This may be compared to the raising of the vibration frequency of a kettle drum by drawing the membrane outward around the edges by the tightening screws. Therefore it may be inferred that centrifugal force raises the natural vibration frequencies of a turbine disk wheel.

41 It is well known that a particle of mass $m$ with an elastic support of such stiffness that a force $R_s$, required to produce unit

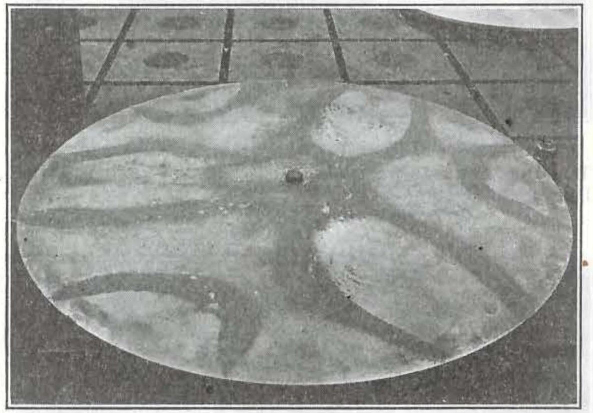

FIG. 25 COMPLEX SAND PICTURE WITH 12 NODES AT THE EDGE AND 6 NODES NEAR THE CENTER MADE BY VIBRATION OF A THIN STEEL PLATE. THIS IS A RARE TYPE OF MOTION.

deflection, will have a natural frequency of vibration, $f_s$, expressed by

$$f_s = \frac{1}{2\pi}\sqrt{\frac{R_s}{m}} \quad \ldots\ldots\ldots\ldots \quad [1]$$

42 If the same particle is supported in another manner with an elastic factor $R_c$, its new frequency will be

$$f_c = \frac{1}{2\pi}\sqrt{\frac{R_c}{m}} \quad \ldots\ldots\ldots\ldots \quad [2]$$

43 Now when both stiffnesses act at once the frequency will be

$$f_r = \frac{1}{2\pi}\sqrt{\frac{R_s+R_c}{m}} \quad \ldots\ldots\ldots \quad [3]$$



44 Suppose $R_s$ to represent the stiffness furnished by elastic supports and $R_c$ the stiffness contributed by centrifugal effects. Assuming the latter proportional to the square of the speed, $N_s$, in revolutions per second, this proportionality may be expressed by the use of an arbitrary coefficient $B$ defined by the relation

$$R_c = B(4\pi^2 m N_s^2) \quad \ldots\ldots\ldots \quad [4]$$

45 Making use of this relation and eliminating $R_s$ by the use of Equation [1] the frequency of the particle, $f_r$, due to the combined effects of stiffness and rotation may be written

$$f_r = \sqrt{f_s^2 + BN_s^2} \quad \ldots\ldots\ldots \quad [5]$$

46 This formula, here derived for the case of a particle, has been justified many hundreds of times for use with a complete turbine bucket wheel by actual measurement as described in later sections of this paper. Stodola[1] arrived at the same conclusion on theoretical grounds.

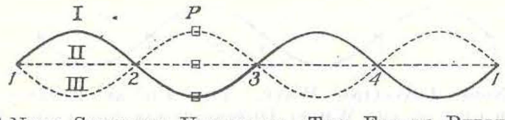


FIG. 26 4-NODE STANDING VIBRATION. THE FIGURE REPRESENTS THE DEVELOPED EDGE OF THE WHEEL DURING THREE SUCCESSIVE PHASES

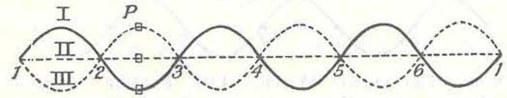


FIG. 27 6-NODE STANDING VIBRATION. THE FIGURE REPRESENTS THE DEVELOPED EDGE OF THE WHEEL DURING THREE SUCCESSIVE PHASES

47 $B$ is the speed coefficient which varies with the design of the wheel and the type of vibration. If the vibrating sectors extend a considerable distance into the wheel so that the deflection curve extends well toward the wheel center, $B$ has a lower value than when most of the bending of the wheel is near its edge, as in the case of a larger number of nodes. The value of the speed coefficient is generally from 2 to 3, and a coefficient as small as unity is rare.

### TRAVELING WAVES

48 Thus far disk-wheel vibrations with radial nodes and the effect of centrifugal force on these vibrations have been discussed in some detail. The type of vibration which has been found to be responsible for serious wheel failures will now be taken up. This type of vibration results when, instead of the wheels vibrating in

[1] *Schweitzerische Bauzeitung*, May, 1914.

segments with stationary radial nodes, a wave train travels around the wheel circumference.

49 Before considering traveling waves, a diagrammatic representation of radial nodal vibrations of a turbine wheel will be presented. Fig. 26 represents diagrammatically the edge of a turbine disk wheel, and shows the curves assumed by it when the wheel is vibrating with four nodes. The drawing shows the edge of the wheel developed as though all points along the entire circumference could be seen at once. Evidently the two ends of each curve correspond to the same point on the wheel and are, therefore, numbered identically.

50 Curves I, II, and III show three successive stages one-quarter of a complete period apart. The point on the wheel edge marked $P$ is chosen half-way between nodal points and vibrates through the maximum amplitude. The points 1, 2, 3, etc. remain stationary as they lie in the quiet nodal radii between the vibrat-

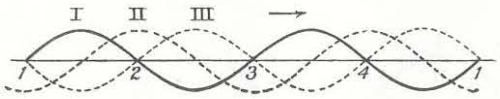


Fig. 28 4-Node Traveling Wave. The Figure Represents the Developed Edge of the Wheel During Three Successive Phases

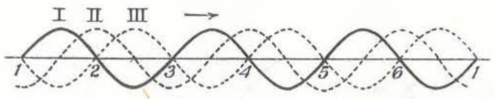


Fig. 29 6-Node Traveling Wave. The Figure Represents the Developed Edge of the Wheel During Three Successive Phases

ing segments. First the wheel edge is bent as shown by the full curve I; one-quarter of a period later the edge becomes straight as shown in curve II, but the portions between nodal points have a rapid motion which carries them over to the maximum deflection in the opposite direction in curve III, one-quarter of a vibration period later, or one-half a period from the initial position. At the end of a full period, the shape evidently is again the same as it was to start with as indicated by the full line curve I. Fig. 27 shows the same sequence for a 6-node vibration.

51 Fig. 28 shows the developed edge of a wheel in a similar manner and represents the case of 4-node traveling waves instead of standing vibrations. The difference between the case of traveling waves and standing vibrations is seen to be that the nodal points 1, 2, 3, etc. move along the edge of the wheel instead of remaining at fixed points.

52 Curve II shows the wheel shape after the nodal points 1, 2, etc. have moved one-quarter of a wave length to the right, and III shows the shape after another one-quarter wave-length



motion where the nodes have moved to the right one-half a wave length in all. At this instant the shape of the wheel is the same as for the case of the standing vibrations previously considered. The difference lies in the motion only. In the case of the standing vibration the nodes are stationary. In the case of the traveling waves the nodes are moving to the right. Fig. 29 shows the same sequence for a six-node vibration.

## Comparisons between Standing Vibrations and Traveling Waves

53 The following comparisons may be made between the standing vibrations and the corresponding traveling waves for a given disk wheel:

*a* In each case there must be an even number of nodes, that is, for every upward portion of the deflection curve there is a corresponding downward portion because of the continuity of the circumference.

*b* In standing vibrations the nodes are stationary in the wheel; in traveling waves they move around it. In the first case we have true nodes in the sense that they represent parts of the wheel which are always quiet so they may be observed by the eye. In the second case we have traveling nodes; every part of the wheel edge vibrates and no quiet zones can be seen. A rapidly moving traveling wave can only be seen by the eye by means of instantaneous illumination.

*c* The *frequency* of vibration of every particle along the edge of a given disk wheel is the same *either for a case of standing vibration or for traveling waves,* provided the number of nodes is the same. This important point will presently be explained. A knowledge of it is requisite to the determination of the velocity of a traveling wave from the standing vibration frequency. For instance, turning to Figs. 26 and 28, it is seen that if the vibration frequencies of each point on the rim are the same in each case, the traveling wave must move to the right one whole wave length, while the standing vibration goes through one complete cycle. Thus *the speed of a traveling wave per second equals the number of complete vibrations of the corresponding standing wave per second multiplied by the length of a complete wave.*

*d* For the standing vibration the *amplitude* of the particles varies along the edge of the disk from zero at the nodal points to the maximum vibration amplitude at points half-way between the nodes. For the traveling waves, all particles around the edge of the wheel vibrate in turn through the same amplitude.

*e* For a standing vibration all of the particles along the edge of the wheel vibrate in the same time phase, that is, all particles vibrate together so that each comes to rest at the same instant and each has its maximum velocity of motion at the same instant

during the vibratory motion. For traveling waves, however, the particles along the wheel edge do not vibrate in time phase but vibrate one after another in turn, successively coming to rest and successively acquiring their maximum velocity of motion during vibration. Since they all vibrate one after another through

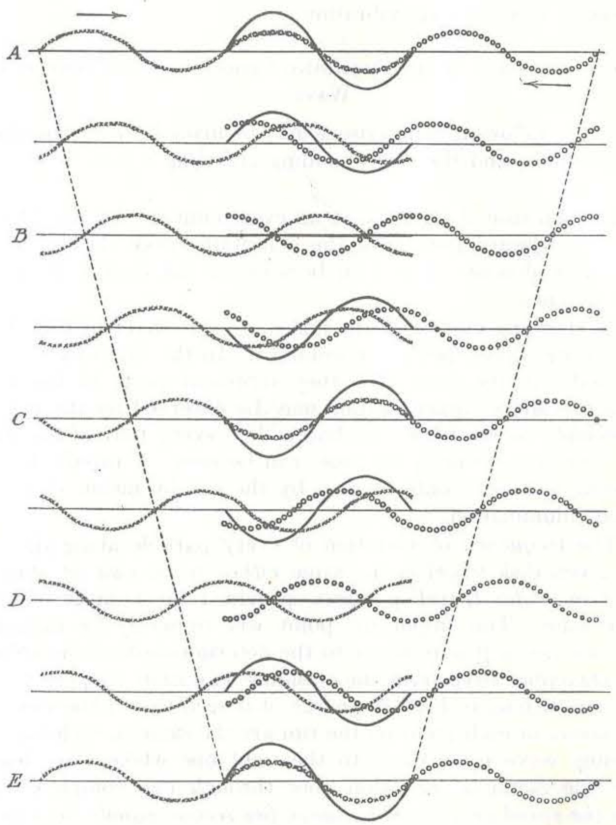


Fig. 30 Composition of Two Equal Waves Traveling in Opposite Directions to Form a Standing Vibration

the same amplitude a wave shape results of constant amplitude traveling around the wheel edge.

54 To sum up the last two paragraphs: for a standing vibration, the particles along the edge of a wheel all vibrate in the same time phase, but their amplitudes vary successively between nodes; for a traveling wave all particles vibrate through the same amplitude, but their time phases vary successively along the wheel edge.



## Relations between Standing Vibrations and Traveling Waves

55 A well-established principle of wave motion is that *standing vibrations with stationary nodes result from the superposition of two identical wave trains traveling in opposite directions, each of which has an amplitude equal to half that of the resulting standing vibration.* A familiar illustration of this principle is observed when two stones are thrown on the surface of a pond giving rise to two outspreading wave trains. Midway between the two stones the two identical wave trains approaching from opposite directions are superposed upon each other. There results in this region a series of standing vibrations of the surface of the pond with stationary nodal points between them. The particles of water vibrate up and down with the same frequency for the standing vibrations as for each of the wave trains of which these vibrations are composed.

56 This illustration taken from mechanics has other parallels. The sound vibrations in an organ pipe with fixed nodal points are similarly explained by a combination of oppositely moving sound waves. In long-distance electric transmission lines standing vibrations with fixed nodes between them may also be produced by the combination of two oppositely moving wave trains.

57 A consideration of Fig. 30 will be useful as an illustration. *A*, *B*, *C*, and *D* show successive stages of a standing vibration for each quarter of its period, resulting from the superposition of two identical wave trains moving in opposite directions. The crosses represent the wave progressing toward the right, while the circles show a leftward-moving wave of equal amplitude. When the stage *E* is reached the cycle is completed and the deflection curve is the same as at the first stage *A*. In the stage *A* the two oppositely moving waves are exactly superposed upon each other so that they add.

58 When each traveling wave has moved one-quarter of a wave length as shown in *B* they cancel, so there is zero up or down displacement at all points, resulting in the straight line. After the second quarter of a wave length of motion both waves are again superposed so that the displacements add, but the displacements are all opposite to those shown in *A*. In stage *D* the deflections cancel again and in stage *E* after each wave train has moved a complete wave length the deflections add again, giving the original deflection curve.

59 The important point to be understood is that the natural frequency of vibration of the particles is the same for a standing vibration as for a traveling wave. It has already been explained that the frequency of a particle depends only on its mass and a stiffness factor represented by the restoring force per unit of displacement which, for isochronous vibrations, is the same for

each unit of mass throughout the entire structure. The principles of elasticity show that these proportional restoring forces, acting upon the various particles of unit mass, depend on the shape of deformation only. Since the shape is the same for either type of motion, it is seen that the vibration period of each particle is the same in either case.

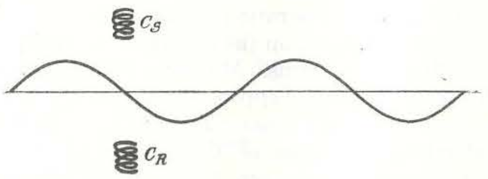


Fig. 31 Developed Edge of a Wheel Carrying a 4-Node Wave

($C_s$ is a stationary magnetic exploring coil and $C_r$ a similar coil revolving at the same speed as the wheel.)

60 This explanation shows how the speed of a wave train in a turbine wheel with a particular number of nodes may be calculated from the standing vibration frequency of the wheel when vibrating with the same number of nodes.

### Wave Speeds

61 The point to be emphasized is that a wave train of a particular number of wave lengths travels around the edge of a turbine wheel at *one particular characteristic speed only.* Just as a 4-node standing vibration responds at one frequency so also the 4-node wave trains of which the standing vibration is composed must travel around the wheel at one particular speed. So also for wave trains of 6, 8, or 10 nodes, etc. Each has one particular speed with which it must travel in the disk wheel.

### Detection of the Presence of Traveling Waves in a Revolving Disk

62 Fig. 31 represents the developed edge of a turbine wheel carrying a 4-node wave train, which moves to the right with a certain particular velocity. $C_s$ is a small magnetic coil fixed to the stationary diaphragm so it can register the to-and-fro motions of the wheel in an oscillograph by means of the inductive effect of the wheel as it approaches and recedes from the magnetic coil during vibration. A coil $C_r$ is attached to an arm which is carried around with the revolving disk wheel, so it also can register the vibration frequency of the revolving wheel in an oscillograph.

63 When the wheel is stationary, both coils register the same frequency. Assume that a wave travels around the wheel 25 times a second. The frequency registered by each coil would be $2\times 25=50$ cycles per second, because for the case of four nodes shown the wheel carries two complete waves.



64 Assume now that the wheel is revolving at 10 r.p.s. in the direction in which the wave moves. Since the wave always has a definite speed *in the wheel,* the coil carried around *with the wheel* should register almost the same frequency as before. The frequency would be exactly the same were it not for centrifugal force, the effect of which we have already discussed. The wheel is stiffened by it so that the vibration frequencies are raised and the wave speeds are increased. The effect would not be very great at 10 r.p.s. Assume, for example, that the wave train travels around the wheel 26 times a second instead of 25 times due to this cause. The revolving coil will then register $2\times 26=52$ cycles per second when the wheel is revolving 10 r.p.s.

65 On the other hand, the stationary coil now registers a higher frequency than it registered when the wheel was stationary. The wave train on the wheel is carried forwards by the wheel motion at a speed of 10 r.p.s. besides its natural speed in the wheel of 26 r.p.s., so the wave passes the fixed coil at a speed of $10+26=36$ r.p.s., and the frequency registered by this coil is $2\times 36=72$, because the wheel carries a train of two waves in the case assumed. Therefore, the forward-traveling wave registers a frequency of 52 cycles per second on the moving coil and 72 cycles per second on the stationary coil, whereas when the wheel was stationary both coils registered 50 cycles per second.

66 Now consider a case where the 4-node wave train is moving backward in the wheel while the wheel is revolving at the same speed of 10 r.p.s. The effect of the centrifugal force is the same as before so the wave must travel *in the wheel* with the same speed of 26 r.p.s. as before, but in the opposite direction. The frequency recorded by the revolving coil is $2\times 26=52$ cycles per second, the same as for the forward-traveling wave, since this coil records the same frequency for the same wave speed whether the wave moves past it forward or backward. The effect upon the frequency recorded by the stationary coil, however, is different. Since this is a backward-traveling wave, the forward motion of the wheel of 10 r.p.s. allows the wave to travel backward past the fixed coil with a speed of only $26-10=16$ r.p.s. In other words, the forward motion of the wheel subtracts from the backward motion of the wave as measured by the fixed coil. The frequency registered by the fixed coil is, therefore, $2\times 16=32$ cycles per second for the 4-node backward wave train.

67 Again, take the case of this disk wheel carrying both wave trains simultaneously and also revolving at 10 r.p.s. For the forward wave train it will be recalled that the revolving coil registers 52 cycles per second and the fixed coil registers 72 cycles per second, while for the backward moving wave train the revolving coil again registers 52 cycles per second, and the fixed coil registers 32 cycles per second. The revolving coil registers only

one frequency of 52 for either wave train separately or for the combination, but the fixed coil registers 72 for the forward-traveling wave and 32 for the backward-traveling wave, and both 32 and 72 simultaneously for the two waves superposed, that is, for the vibration in the wheel.

68 To sum up Pars. 66 and 67, it may be said that when a disk wheel carrying a standing vibration is revolved so that the radial nodal lines are carried around with the wheel, the frequency recorded by a coil carried around with the wheel slowly rises due

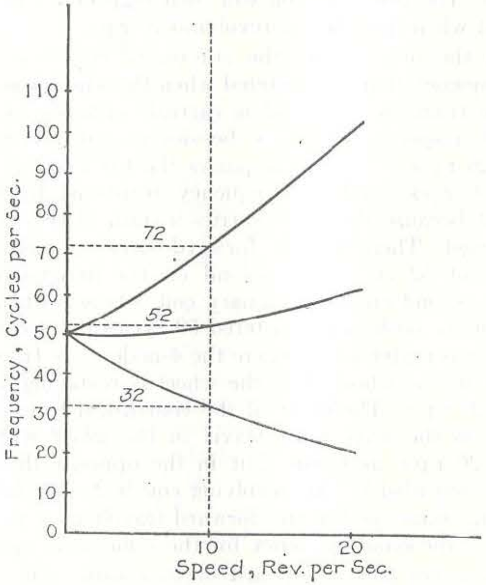


Fig. 32 Frequency-Speed Diagram for 4 Nodes

to centrifugal force as the speed of the wheel increases. The frequency recorded by a coil fixed on the diaphragm, so that it does not revolve with the wheel, registers two frequencies, the higher frequency due to the forward-moving component wave train and the lower frequency of the backward-moving component wave train. These two frequencies diverge more and more as the wheel speed is increased.

## Frequency-Speed Diagram

69 These facts are shown graphically in Fig. 32 which gives a diagrammatic representation that has been found to be very useful.



70 The vertical scale represents the frequency registered in an oscillograph by the magnetic coils, and the horizontal scale the rotational speed of the disk. The middle curve gives the variation of frequency with speed as recorded by the revolving coil. The upper and lower curves show the two frequencies registered by the fixed coil, the upper giving the frequency due to the forward component wave and the lower the frequency due to the backward component wave train of the 4-node vibration. When the wheel is at rest the figure shows that both coils register 50. As the speed of the wheel is raised to 10 r.p.s. the revolving-coil frequency rises to 52 and the two frequencies recorded by the fixed coil diverge, the upper rising to 72 and the lower falling to 32.

71 The gradual rise of frequency of the wheel as its speed is increased is expressed by equation [5] previously derived

$$f_r = \sqrt{f_s^2 + BN_s^2}$$

The upper curve shows how the frequency of a forward-moving wave train, as measured at a fixed point, rises relatively to the frequency detected by the revolving coil, because this wave train is carried forwards by the wheel, and is thus passing the fixed coil at a higher speed than it would were the wheel not rotating. This rise in frequency is measured by the number of wave lengths per second that the wave train is carried forward by the wheel rotation which equals the product of the number of waves on the wheel rim, $\frac{1}{2}n$, by the number of revolutions per second of the wheel, $N_s$. This product $\frac{1}{2}nN_s$ is the frequency in excess of that of the wheel as measured by the revolving coil, that is, in excess of $f_r$. If $H$ is the higher frequency recorded by the stationary coil and represented by the upper curve, then

$$H = f_r + \tfrac{1}{2}nN_s \quad . \ . \ . \ . \ . \ . \ . \ . \ . \ [6]$$

In the same way the lower curve shows how the frequency of the backward-traveling wave as measured at a fixed point is decreased because in this case the wave motion is opposite in direction to the motion of the wheel. Thus if $M$ is the lower frequency recorded by the stationary coil and represented by the lower curve,

$$M = f_r - \tfrac{1}{2}nN_s \quad . \ . \ . \ . \ . \ . \ . \ . \ . \ [7]$$

72 If the backward-moving component wave train is absent, only the upper frequency is registered by the fixed coil corresponding to a forward-moving traveling wave. If the forward-moving component wave train is absent, only the lower frequency is recorded due to the backward-traveling wave. The frequency recorded by the revolving coil, however, is always the same whether one or both of the component wave trains exist and in whatever relative amplitudes they exist.

73 It is therefore evident that by the use of two exploring coils as described, one revolving with the wheel, the other being

fixed in space, the presence of a forward- or a backward-traveling wave train or both can be detected.

74 The first observation of traveling waves in a turbine wheel was made by means of fixed oscillograph coils installed in the diaphragms of an operating turbine in 1919. Early in 1920 during

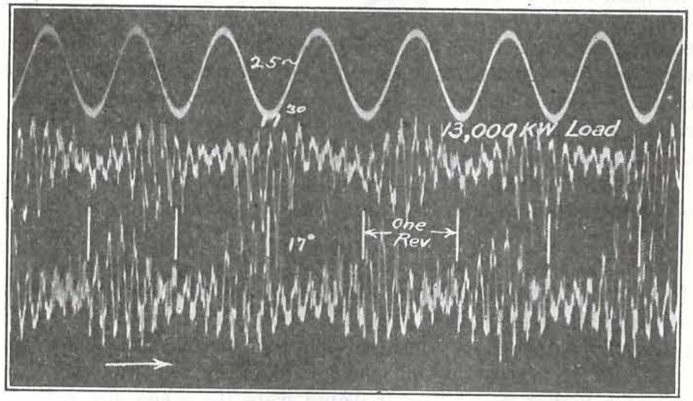


Fig. 33 Oscillograph Records made by 17th Stage of 20,000-Kw. 1500-R.P.M. 23-Stage Turbine

(The upper curve is a 25-cycle timing wave. The other two curves were made by stationary coils 30 deg. apart. This is a case of 6-node forward- and backward-traveling waves.)

the investigation of another turbine, several stages were equipped with two such coils 30 deg. apart. One of these stages yielded the records reproduced in Fig. 33.

### Observation of Traveling Waves by Means of Two Fixed Coils 30 Deg. Apart

75 The upper curve of Fig. 33 is produced by the 25-cycle a.c. generator being driven by this turbine. Since the generator has two poles, it revolves once for every cycle, and this a.c. frequency curve marks off the generator revolutions on the film. The two lower oscillograph curves are the records of the two fixed coils, 30 deg. apart. Time is measured to the right. The upper of these two curves is the record of the first of the two fixed coils. The lower curve is the record of the second coil, set in the diaphragm 30 deg. beyond the former so a given point on the disk wheel reaches this coil somewhat later than the first one.

76 The records of these two coils show a close correspondence, both having a high-frequency oscillation which goes through a low-frequency pulsation in amplitude giving the effect of beats. Furthermore the upper curve lies behind the lower one by about one-quarter of the distance between the low-frequency amplitude



pulsations or beats. Since the time recorded by the amplitude pulsations is four times as great as that by which the upper record lies behind the lower, due to the 30 deg. between the coils, the most likely explanation is that there are high spots on the wheel, 4×30 deg., or 120 deg., apart, which cause this amplitude pulsation. This can be made clear from a consideration of Fig. 34.

77 The wheel revolves in a counterclockwise direction as shown by the arrow. The two coils marked 1 and 2 are 30 deg. apart,

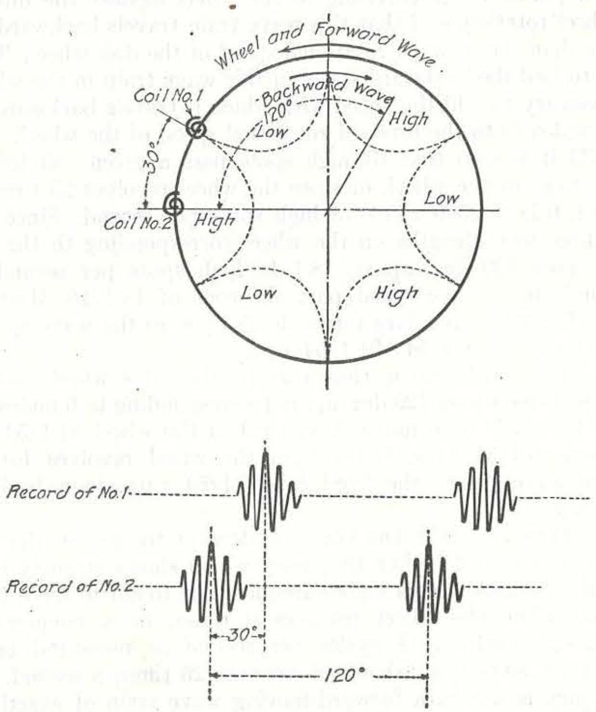


Fig. 34 Diagram Showing 6-Node Forward and Backward Waves Such as are Recorded in Fig. 33

and are fixed in space. The diagrammatic oscillograph record shows the beats recorded by the two coils, No. 1 lagging behind No. 2 by one-quarter of a period. Since it takes four times as much time for successive high spots to reach a given coil as for a given high spot to pass from one coil to the other, these high spots must be 4×30 deg., or 120 deg., apart on the revolving disk. The inference is that these high spots are wave crests corresponding to a train of three waves, 120 deg. apart and that the waves cause the disk wheel to approach and recede from the recording coils periodically. When the wheel is close to a coil

the oscillograph responds strongly, and when it recedes the oscillograph responds less strongly.

78 If there is such a wave train the question now is to determine its direction and speed. The high spots evidently move in a clockwise direction opposite to the wheel rotation, because they reach coil No. 2 before they reach coil No. 1, as shown in Fig. 33 where the record of coil No. 2 shows a time lead over that of coil No. 1. This means that the wave which causes these low period pulsations is traveling in the wheel against the direction of wheel rotation, and that this wave train travels backward even faster than the forward rotational speed of the disk wheel. Therefore, to find the backward speed of this wave train in the wheel it is necessary to add the speed with which it travels backward past the fixed coils to the forward rotational speed of the wheel. From Fig. 33 it is seen that $6\frac{1}{2}$ high spots pass a given coil for nine revolutions of the wheel, or since the wheel revolves 25 times per second, $6\,1/2 \times 25/9 = 18\,1/18$ high spots per second. Since there are three wave lengths on the wheel corresponding to the three high spots 120 deg. apart, 18 1/18 high spots per second corresponds to a wave speed past the coils of $18\,1/18 \div 3 = 6\,1/54$ r.p.s. The wheel revolves forwards 25 r.p.s. so the wave speed in the wheel $= 25 + 6\,1/54 = 31\,1/54$ r.p.s.

79 The conclusion is thus reached that this wheel carries a train of three waves 120 deg. apart (corresponding to 6 nodes) and that this wave train moves backward in the wheel 31 1/54 r.p.s. which is 6 1/54 r.p.s. faster than the wheel revolves forward, so the wave passes the fixed coils 6 1/54 r.p.s. in a backward direction.

80 Thus far there has been no attempt to explain the cause of the superposed higher frequency which shows strongly in the record of Fig. 33. This higher frequency is found to have 60 1/2 periods while the wheel revolves 9 times, or a frequency of $60\,1/2 \times 25/9 = 168\,1/18$ cycles per second as measured on the fixed coils, since the disk wheel revolves 25 times a second. This frequency is due to a forward-moving wave train of exactly the same type as the backward-moving wave train, that is, a train of three waves 120 deg. apart, or a 6-node wave train. If such a wave train registers a frequency of 168 1/18 cycles per second on the fixed coils, its speed past these coils in r.p.s. must be $168\,1/18 \div 3 = 56\,1/54$ r.p.s., because there are three wave lengths on the wheel rim. Since this wave train is assumed to move forward and the wheel is also moving forward, its speed *in the wheel* must be less than that registered by the fixed coil by an amount equal to the wheel speed, that is, 25 r.p.s., because the wave train is carried forwards by the wheel rotation. This gives a wave speed in the wheel of 31 1/54 r.p.s. But this is exactly the characteristic speed of a train of three waves as it checks with the speed of the backward-traveling wave train of this type already found. Par. 61 showed that a given type of wave has a definite speed in a disk wheel which revolves at a given speed, and that this wave speed is the same whether the wave train travels forward or backward in the wheel. This coincidence is therefore striking evidence of the truth of the statement that the high frequency registered was caused by a forward-moving wave train of six nodes, that is, of the same type as the backward-moving wave train.



81 There is further evidence that the wave train recording the higher frequency is moving forward and the wave crests are 120 deg. apart as in the case of the backward waves. From a close examination of the film record of Fig. 33 it will be seen that the higher frequency of coil No. 1 leads that of the lower record by a fraction of a period. (The polarity of the two coils happens to be opposite in this record.) This means that the disturbance producing this harmonic moves *forward* because it reaches coil No. 1 before it reaches coil No. 2. Furthermore this lead is as before about one-quarter of a complete period. This again corresponds to waves which are 4×30 deg., or 120 deg. apart.

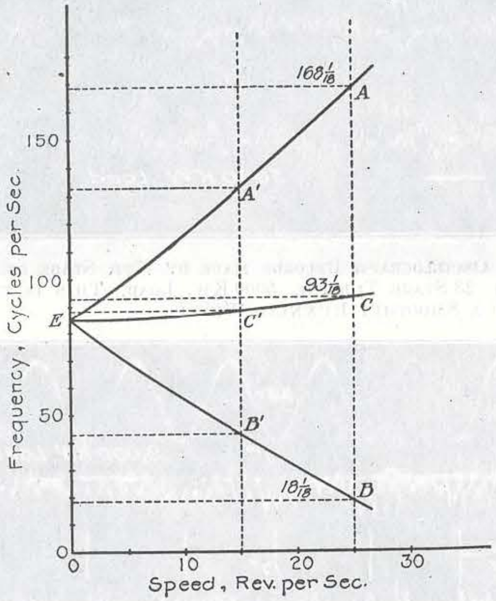


FIG. 35 FREQUENCY-SPEED DIAGRAM FOR 6 NODES. POINTS *A*, *B* AND *C* WERE DETERMINED FROM THE RECORD IN FIG. 33

82 As to the relative amplitudes of these two wave trains, a casual inspection of the film Fig. 33 might lead one to believe that the amplitude of the forward wave train producing the

higher frequency was as great as that of the backward wave train. It is necessary to keep in mind, however, that in an oscillograph record the amplitude is dependent on the induced voltage which in turn depends on both the amplitude and the frequency of the vibration so that higher frequencies have amplitudes recorded

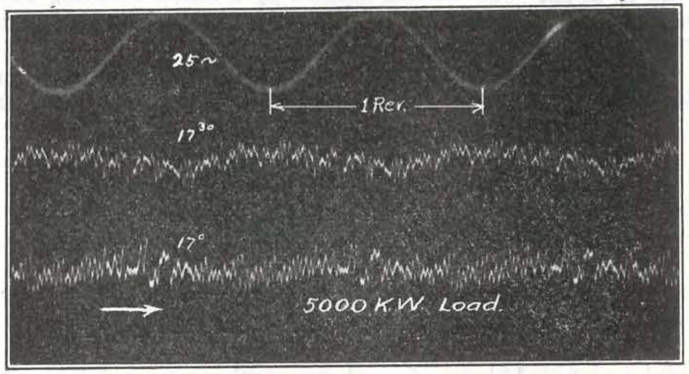


Fig. 36 Oscillograph Records made by 17th Stage of 20,000-Kw. 1500-R.p.m. 23-Stage Turbine, 5000-Kw. Load. This is the "Autograph" of a Smoothly Running Wheel

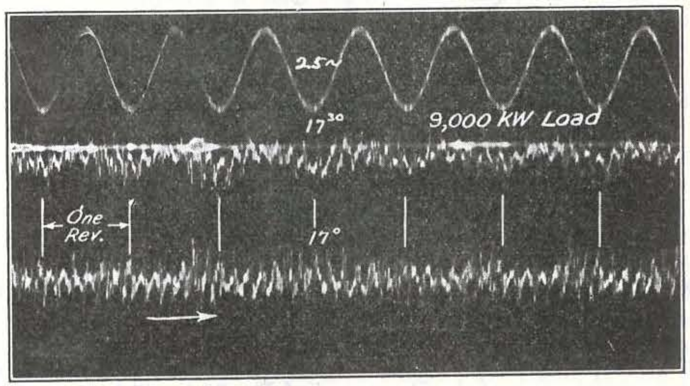


Fig. 37 Oscillograph Records made by 17th Stage of 20,000-Kw. 1500-R.p.m. 23-Stage Turbine, 9000-Kw. Load. Wave Motion Has Not Yet Developed

which are magnified in proportion to the increase of frequency. For instance, since the higher frequency is about nine times as great as the lower in Fig. 33, the higher frequency would be expected to be amplified about nine times as much as it should be compared with the lower frequency recorded. In all probability the amplitude of the backward-traveling wave is greater than that of the forward wave. There are other reasons for believing this, to be considered later.



83 The film record of Fig. 33 which has just been analyzed is the one which is reproduced on page 916 of the fifth edition of Stodola's book on Steam and Gas Turbines.

84 Fig. 35 shows the frequency speed diagram for this wheel for six nodes. *A* and *B* correspond to the high and low frequencies recorded by the film shown in Fig. 33, i.e., 168 1/18 and 18 1/18

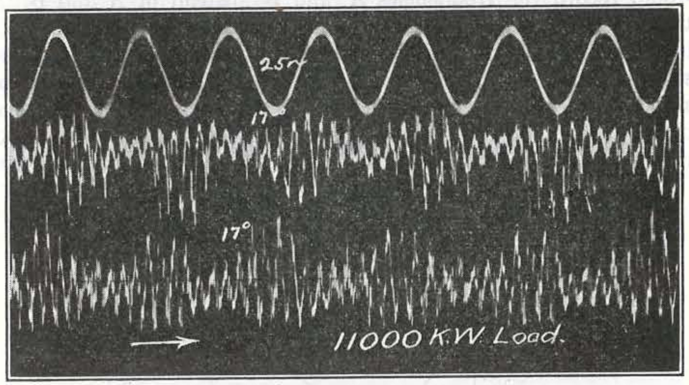


Fig. 38 Oscillograph Records made by 17th Stage of 20,000-Kw. 1500-R.p.m. 23-Stage Turbine, 11,000-Kw. Load. Wave Motion is Fully Developed. Compare with Fig. 33

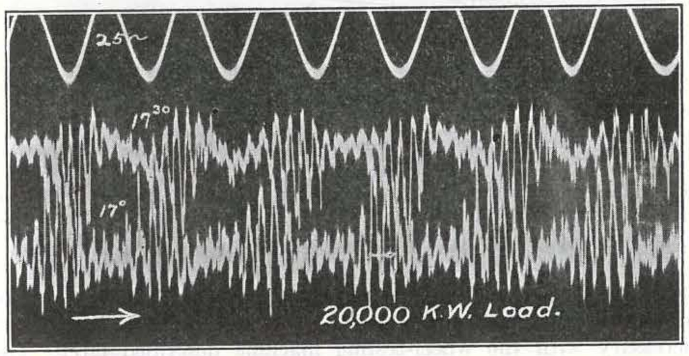


Fig. 39 Oscillograph Records made by 17th Stage of 20,000-Kw. 1500-R.p.m. 23-Stage Turbine, 20,000-Kw. Load. Wave Motion Still Maintained

cycles per second or to the wave speeds 56 1/54 and 6 1/54 r.p.s., because in the case of six nodes the wheel carries three complete waves. *C* equals the value of $f_r$, the frequency of the wheel itself rotating at the normal running speed of 25 r.p.s. This may be calculated by formulas derived from Equations [6] and [7].

$$f_r = H - \tfrac{1}{2}nN_s \quad . \; . \; . \; . \; . \; . \; . \; . \quad [8]$$

$$f_r = M + \tfrac{1}{2}nN_s \quad . \; . \; . \; . \; . \; . \; . \; . \quad [9]$$

Thus $f_r = 168\,1/18 - 75 = 93\,1/18$

$f_r = 18\,1/18 + 75 = 93\,1/18$

85 Suppose the 6-node wave train should still persist with the speed of the disk wheel whose 6-node characteristics are shown in Fig. 35 reduced from 25 r.p.s. to 15 r.p.s. Then the fixed coil would record the frequencies $A'$ and $B'$ instead of $A$ and $B$. If there were a revolving coil, it would record the frequency $C'$ instead of $C$. If the wheel were brought to rest with the wave still persisting, both coils would record the same frequency or the standing frequency $E$ for six nodes. It can therefore be seen that if the standing frequency $E$ is measured for a given number of nodes such a diagram can be constructed to a fair approximation,

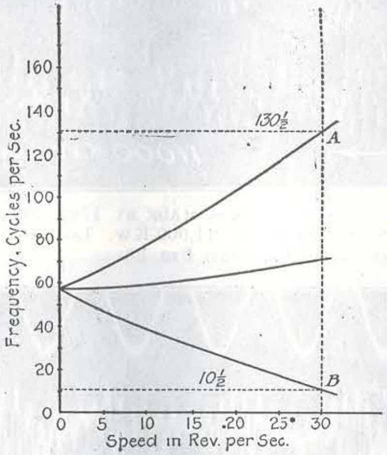


Fig. 40 Frequency-Speed Diagram for 4 Nodes. 17th Stage of 15,000-Kw. 1800-R.p.m. 23-Stage Turbine

(Points $A$ and $B$ were determined from the record in Fig. 41.)

because an approximate value of the speed coefficient can be assumed. For dependable results, however, rotational tests are necessary with the wheel-testing machine described later. The frequency-speed diagram may have on it curves for all of the usual types of radial nodal vibrations as, for instance, 4, 6, 8, and 10 nodes.

86 Fig. 33, the record just discussed, is the record of the 17th stage of a 23-stage, 20,000-kw. turbine. This record was taken while the machine was carrying 13,000 kw. load. Fig. 36 shows a record of the same wheel, but with only 5000 kw. load. No vibration phenomena developed at this load. The jagged and irregular record repeats exactly for each revolution. It may be regarded as the wheel autograph, and due to slight irregularities in the rim opposite which the coils are placed. These are magnified



because of the high speed. Fig. 37 shows where the load has been raised to 9000 kw. Not until the load reaches 11,000 kw. as shown in Fig. 38 do the vibration phenomena distinctly develop. Fig. 39 shows the record at a 20,000-kw. load or full load. The

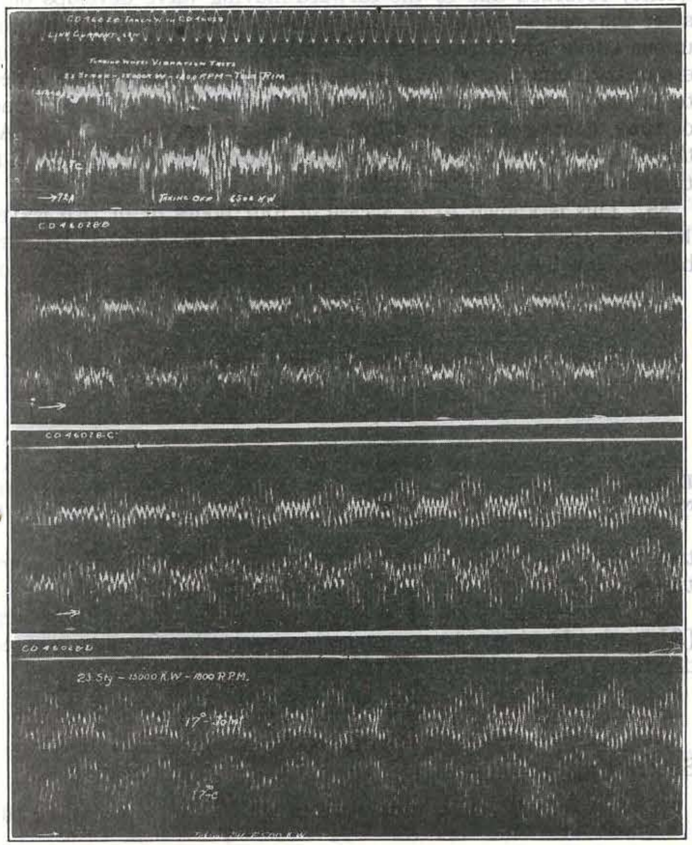

Fig. 41 Oscillograph Records made by 17th Stage of 15,000-Kw. 1800-R.p.m. 23-Stage Turbine

(The upper curve is the 60-cycle line current. The other two curves were made by stationary coils 90 deg. apart. 4-node forward- and backward-traveling waves are indicated.)

wave phenomena when once developed appear to remain about the same up to 20,000 kw. load.

## Observation of Traveling Waves by Means of Two Fixed Coils 90 Deg. Apart

87 Fig. 40 gives the frequency-speed diagram for the 17th stage of a 23-stage, 1800-r.p.m., 15,000-kw. turbine, where wave

motion in a wheel was detected by means of two fixed coils on the diaphragm. In this case the coils were 90 deg. apart. Fig. 41 shows the record from which the diagram was made. This case differs from the one previously described in that the higher frequency recorded due to the forward-moving wave develops only after the load is removed. The removal of load is shown by the upper curve which registers the electrical frequency of the generator becoming a straight line, remembering that time is measured to the right. The higher frequency develops in about one second of time, corresponding to 60 a.c. cycles of the upper curve. As this is a 4-pole machine two a.c. cycles correspond to one revolution, and 30 r.p.s. is the running speed of the machine.

88 This is known to be a case of four nodes or of two waves 180 deg. apart, because from the record it appears that it takes half as long for a high spot to go from one coil to another as for

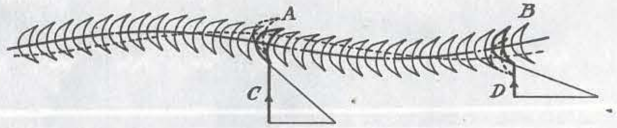


FIG. 42 THE CHANGE OF ANGLE OR "FEATHERING" OF A BUCKET DURING THE PASSAGE OF A TRAVELING WAVE PERMITS THE MAINTENANCE OF THE WAVE BY MEANS OF ENERGY ABSORBED FROM THE STEAM

two successive high spots to pass one coil. The high spots are twice as far apart as the coils, that is, 2×90 deg.=180 deg.

89 Another good check is obtained by the use of Equations [6] and [7] derived in connection with the frequency-speed diagram.

$$H = f_r + \tfrac{1}{2}nN_s$$
$$M = f_r - \tfrac{1}{2}nN_s$$

Subtracting,

$$H - M = nN_s, \text{ or } n = \frac{H-M}{N_s} \quad \ldots\ldots [10]$$

90 From the frequency-speed diagram shown in Fig. 35 corresponding to the film of Fig. 33 previously discussed, $H=168\ 1/18$ cycles per sec., $M=18\ 1/18$ cycles per sec., and $N_s=25$ r.p.s.

91 Thus from Equation [10]

$$n = \frac{H-M}{N_s} = \frac{168\ 1/18 - 18\ 1/18}{25} = 6 \text{ nodes}$$

92 For the case shown in Figs. 40 and 41 an exact analysis is difficult because where the higher frequency comes out clearly, the machine has doubtless increased slightly in speed, due to the sudden dropping of the 6500-kw. load. There can be no doubt,



however, that the following interpretation is very close to the truth.

$H = 130\frac{1}{2}$ cycles per sec., $M = 10\frac{1}{2}$ cycles per sec., $N_s = 30$ r.p.s.

$$\frac{H-M}{N_s} = \frac{130\frac{1}{2}-10\frac{1}{2}}{30} = 4 \text{ nodes}$$

## "FEATHERING" ACTION OF BUCKETS A POSSIBLE CAUSE OF TRAVELING WAVES

93 Thus far nothing has been said about the cause of vibration in the two cases just described. Further study brought out the fact that waves of this sort rarely occur in turbine disk wheels and the phenomenon is confined to unusually thin types of wheels

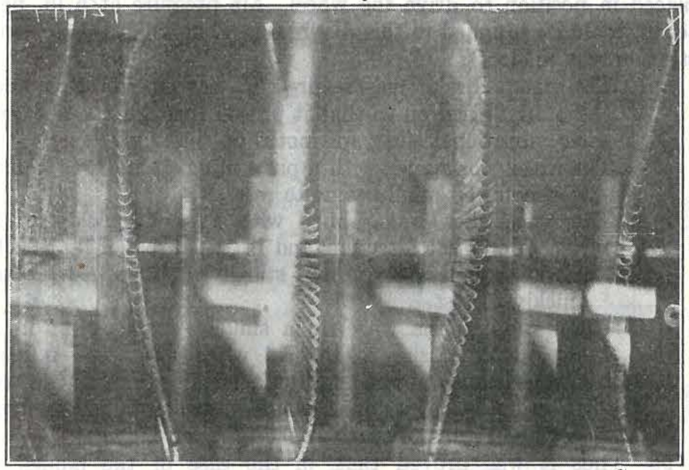

FIG. 43 A TRAVELING WAVE IN A MODEL WHEEL KEPT IN MOTION BY HIGH-VELOCITY AIR FROM MODEL DIAPHRAGM NOZZLES

in which waves are easily built up. After these films were analyzed, a satisfactory explanation of the cause was sought. Referring to Fig. 42 in which the circumference of the wheel disk is formed into a wave shape, the relative angular twisting of the two buckets *A* and *B* will be somewhat as shown. It will be seen that the axial component from the energy left in the steam at the point of leaving the buckets as shown at *C* and *D* will be greater at *C* than at *D*, both reactions being vertically upwards in the figure.

94 Assuming the wheel to be stationary in space and the wave form to move toward the right in the wheel to a new position as indicated by the dotted line, it will be seen that the buckets *A* and *B* are moved to the positions shown dotted. This means that the force *C*, which is larger than *D*, is operating on

the bucket $A$ in the direction that $A$ is moving, due to the wave transition. At the same instant the force $D$ is opposing the motion of bucket $B$ moving in a downward direction due to the wave transition. However, since the forces acting in the direction of bucket motion, as at $C$, are greater than the forces opposing the bucket motion, as at $D$, it is clear that energy is added to the maintenance of the wave form after it has been initiated.

95 Fig. 43 was taken from an apparatus specially designed to illustrate the action above described. A small thin sheet-metal disk about 18 in. in diameter was supplied with model turbine buckets around the circumference. This was carried on a shaft mounted in bearings with a small prony brake fitted to the same shaft. A model diaphragm with uniform nozzle openings around the entire circumference was secured to an air-tight box and placed in position to drive the wheel. Air pressure at 5 lb. per sq. in. could be supplied to this diaphragm. The following demonstration was made:

1 The prony brake was secured to prevent rotation and the air turned on so that it passed through the nozzles. No vibrational effect was noted on the wheel.

2 The wheel was struck with a piece of wood and was found to shiver and finally come to rest.

3 The wheel disk was rubbed with a stick quickly in a circumferential direction and in the direction in which it was designed to run, but still it was found to return quickly to rest.

4 Upon rubbing the wheel in a similar manner, but in a direction opposite to that in which it should rotate, a wave shape of large amplitude, traveling in a backward direction, was developed, which was maintained as long as the air pressure was applied.

96 Thus it was proved that, in the case of the model, a backward-traveling wave could be maintained after once being initiated, simply by the passage of the air current through the buckets in the usual manner. The reason just given for the maintenance of the backward wave due to this action can also be used to prove that the forward wave would be damped, as in this case the larger force $C$ would be working against the motion of the bucket. This explains why a forward wave was not maintained when the wheel was rubbed in the forward direction.

97 After the backward-traveling wave was set up in the manner already described, the prony brake was gradually released, allowing the wheel to rotate and gradually pick up speed. The wave shape was still maintained but the velocity of the wave relative to space became slower and slower as the wheel accelerated until finally the wave shape stood stationary in space. An amplitude large enough to rub the diaphragm in spots could be built up by slightly increasing the air pressure.



## Vibrations Due to Forces of Resonant Frequencies

98 A rotating turbine disk wheel may readily be made to vibrate with the application of an alternating force corresponding to any one of its resonant frequencies. Fig. 44 shows the frequency-speed diagram of a wheel covering the cases of 4-, 6-, 8-, and 10-node vibration frequencies. Higher vibration frequencies exist, but they are not so important since they are not so readily excited as the lower frequencies. Suppose the wheel to be revolving at a speed of 30 r.p.s. If an alternating transverse force be applied to the rim of this wheel by means of an a.c. magnet fixed in space, the wheel should vibrate in response to any one of 8 different

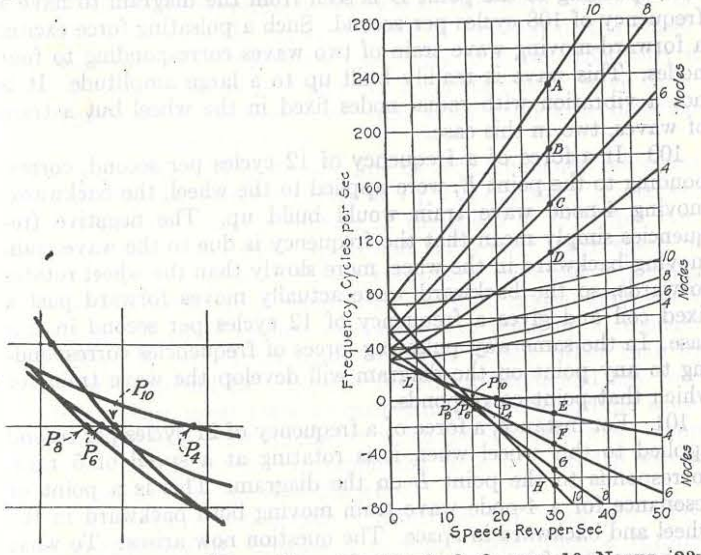


**Fig. 44 Frequency-Speed Diagram for 4, 6, 8, and 10 Nodes, 23d Stage of 15,000-Kw. 1800-R.p.m. 23-Stage Turbine**

(Points $A$, $B$, $C$, $D$, $E$, $F$, $G$, and $H$ show frequencies which, should they occur, would provoke resonant vibration. $P_8$, $P_6$, $P_{10}$, and $P_4$ (shown in detail in sketch at the left) are the wheel critical speeds in the order in which they occur for this wheel.)

frequencies $A$, $B$, $C$, $D$, $E$, $F$, $G$, and $H$, corresponding to the speed of revolution of the wheel which, in this case, was assumed to be 30 r.p.s. They are the very frequencies which can be observed for 4-, 6-, 8-, and 10-node vibrations by an oscillograph coil which is fixed opposite the rim of the wheel in the same way as the a.c. magnet is fixed. They are the *resonant* frequencies of the wheel for 4, 6, 8 and 10 nodes for a pulsating force which acts transversely from a point fixed in space opposite the wheel. It is on this account that only the two curves of the frequency-speed diagram are considered which correspond to a fixed coil in determining these frequencies, namely, the upper and lower curves

corresponding to frequencies arising from forward and backward wave trains. The middle curves represent frequencies which would be observed by a coil carried around with the wheel and have no connection with a fixed coil or a fixed pulsating force.

99 The points to fix clearly in mind are: (*a*) that a pulsating force of the right frequency applied at a fixed point to a disk wheel revolving at a definite speed may excite wheel vibrations corresponding to any one of a series of resonant frequencies such as *A, B, C, D, E, F, G,* and *H,* as shown in Fig. 44; and (*b*) that these frequencies in each case can correspond to only a single train of traveling waves. For instance, an applied alternating force corresponding to the point *D* is seen from the diagram to have a frequency of 108 cycles per second. Such a pulsating force excites a forward-moving wave train of two waves corresponding to four nodes. This wave is readily built up to a large amplitude. It is not a vibration with radial nodes fixed in the wheel but a train of waves, two in this case.

100 If a force of a frequency of 12 cycles per second, corresponding to the point *E,* were applied to the wheel, the backward-moving 4-node wave train would build up. The negative frequencies simply mean that the frequency is due to the wave train moving backward in the wheel more slowly than the wheel rotates forwards, so the backward wave actually moves forward past a fixed coil and gives a frequency of 12 cycles per second in this case. In the same way pulsating forces of frequencies corresponding to any point on the diagram will develop the wave train for which that point corresponds.

101 For instance, a force of a frequency of 21 cycles per second applied to this wheel when it is rotating at a speed of 5 r.p.s. corresponds to the point *L* on the diagram. This is a point of resonance for a 4-node wave train moving both backward in the wheel and backward in space. The question now arises: To what do the points of intersection (such as $P_4$ on the 4-node curve) of these curves with the zero frequency line correspond? Here is a wave moving backward in the wheel which registers zero frequency on a coil fixed in space. In other words, a wave train is standing stationary in space because it moves backward in the wheel at the same speed that the wheel moves forward. These waves, although moving in the wheel, are stationary to an observer. This is the most important case of bucket-wheel wave motion, because practically all serious wheel failures have been definitely proved to be the result of this particular type of vibration. This particular condition in turbine wheels is discussed in greater detail in the next section.

102 In this section of the paper, two general causes of wheel vibration have been considered: (*a*) a feathering action of the steam on the buckets which may maintain waves; and (*b*) pulsating forces which may cause various resonance responses of the wheel. The first type of vibration is eliminated by the use of wheels of sufficient thickness so that the energy dissipation during vibration is too great to permit a building up of such a vibration. The second type of vibration is eliminated by building the disk wheels so that resonant frequencies are removed so far from disturbing frequencies in the turbine that vibrations do not occur.



## Stationary Waves and Critical Speeds of Wheels

103 It has already been mentioned that the type of vibration responsible for practically all serious wheel failures consists of a train of backward-traveling waves whose backward speed in the wheel exactly equals the forward speed of rotation of the wheel. This results in waves which are stationary in space. Evidently a fixed coil would register no frequency for a stationary wave train, so the presence of such a wave train alone cannot be detected by means of a single fixed coil.

104 Referring to Fig. 44, the line of zero frequency intersects the 4-, 6-, 8-, and 10-node curves at $P_4$, $P_6$, $P_8$, and $P_{10}$, respectively. When the wheel is running at the speed of 19.3 r.p.s. corresponding to $P_4$, the wheel speed and the 4-node wave speed in the wheel are the same so that a stationary wave corresponding to a backward-traveling wave train of four nodes may easily be built up. A small amount of energy may build up a resonant wave train. A wave train made to travel at any other than its natural speed would be a type of forced vibration and has been found to require great force to maintain it. Every turbine wheel has a series of particular speeds for which the speeds of wave trains of 4, 6, 8, 10 nodes, etc. corresponding to 2, 3, 4, and 5 waves, etc. are equal to the speed of the wheel.

105 These particular speeds of a turbine disk wheel are called *wheel critical speeds,* because it is found that when a turbine wheel is running at any one of these speeds, it is possible for a wave train of large amplitude to develop which may cause the wheel to fail. In other words, turbine wheels are liable to develop stationary waves, but stationary waves can only occur at particular speeds of the wheel, called critical speeds, one for each type of wave train. $P_4$, $P_6$, etc., on Fig. 44 are some of the critical speeds for this particular wheel. Others exist for higher numbers of nodes, but these have not been found to be so serious.

## Probable Cause of Stationary Waves

106 Thus far little has been said about the cause of the development of stationary wave trains at critical wheel speeds. That turbine wheels may develop this particular type of vibration with comparative ease is beyond question, because these wave trains may develop during test *without the application of any special external force.* When once the nature of these stationary

waves is well understood, however, the cause of their development is not difficult to assign.

107 It has been stated that traveling waves could be excited by a pulsating force fixed in space, having a frequency corresponding to the speed of the waves past it. When the wave train is stationary in space, the pulsation of the force plainly is no longer required. In other words, a spot of extra pressure is sufficient to maintain a stationary wave. It is found that the application of a fixed force of only a few pounds, such as a small direct-current magnet or a small steam jet, to the side of a turbine wheel causes it to respond strongly at a whole series of critical speeds, even up to critical speeds corresponding to 16- or 18-node wave trains of eight or nine wave lengths on the wheel, and it responds only at critical speeds. It can be shown by calculation that such a force of only two or three pounds may give a continuous supply of energy to a standing wave train, and that this energy, despite the small force, may easily amount to 40 or 50 watts.

108 Tests on the energy necessary to maintain vibrations of various amplitudes in turbine disk wheels show that this amount of energy may readily maintain a wave train of four or six nodes, of amplitude sufficient to cause serious trouble. Furthermore, small amplitudes which can be detected by oscillograph coils during test may require no more than a few watts of energy to maintain them.

109 This statement seems amazing in connection with a steel turbine disk wheel, but it has been definitely proved by tests. When we consider a wheel operating in a turbine under the action of steam, it hardly needs to be said that the slightest irregularity in the nozzles might result in a transverse steam force on the wheel a few pounds greater from some nozzles than from others. Only a few pounds difference is sufficient to cause serious trouble in a wheel running at a critical speed. Therefore, wheels must be so designed that they do not operate at any of their critical speeds, as there is always a possibility of such small forces being present in turbines.

## Importance of Critical Speed

110 Clear evidence of stationary waves has been observed in certain cases of failure and also in some cases where failure has not resulted. Scorings were produced on the diaphragm in spots, 180 deg. apart or 120 deg. apart, by the neighboring wheel, the high spots of the stationary wave actually rubbing the diaphragm and producing marks. Figs. 12 and 13 show a case where the 17th-stage diaphragm of a 30,000-kw. turbine is badly scored in two spots 180 deg. apart. These were doubtless caused by a 4-node stationary wave. Fig. 45 shows the 11th-stage diaphragm of a 30,000-kw. turbine, on which three scorings appear due to a 6-node stationary wave train.



111 In several cases similar rubbing has occurred on the diaphragms on each side of the wheel, there being an equal number of spots cut on each side, the spots on the exit side alternating with those on the entrance side. In these cases of local diaphragm rubbing, the wheel has rubbed around its entire circumference.

112 While such occurrences point toward standing waves as being a possible cause of trouble, the most convincing evidence of all as to the importance of critical speeds is given in Table 2. Nearly every case of wheel and bucket troubles on record shows

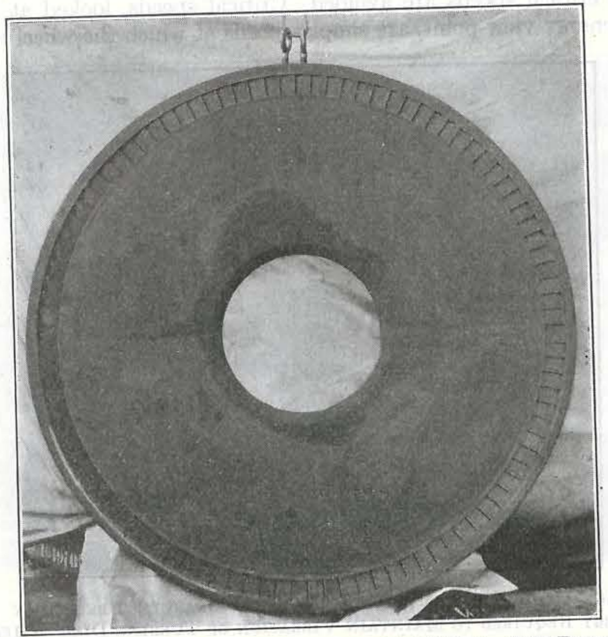

Fig. 45 11th-Stage Diaphragm of 30,000-Kw. 1800-R.P.M. 17-Stage Turbine Showing Scorings Produced by Rubbing of the Shroud Band of the Neighboring Wheel at Three Equidistant Spots 120 Deg. Apart Just Outside the Nozzles, Due to a 6-Node Stationary Wave at Critical Speed

a coincidence between some particular wave speed in the bucket wheel and running speed. Furthermore, many tests on actual turbine wheels in the vibration-testing machines have confirmed the truth of this conclusion.

## Vibrations from the View-Point of Energy

113 The determining factor in the building up and maintenance of vibrations and waves is the relation between energy supply and dissipation. Given a certain supply of energy tending

to build up waves and a small amount of dissipation of the wave energy per unit of amplitude, the amplitude of the wave will be large. Given a large amount of dissipation of energy, the wave amplitude will be correspondingly small.

114 Two ways of preventing vibrations of waves therefore appear: (*a*) Decrease the possibility of absorption of energy in wave production; (*b*) Increase the dissipation of the wave energy.

115 Both methods are used in the production of disk wheels. The first is used by so adjusting the wheel vibration frequencies that critical speeds are avoided. Critical speeds, looked at from the energy view-point, are simply speeds at which the wheel easily absorbs wave energy. The second method is used in the increased thickness of disk wheels, whereby the dissipation of vibration energy may be greatly increased.

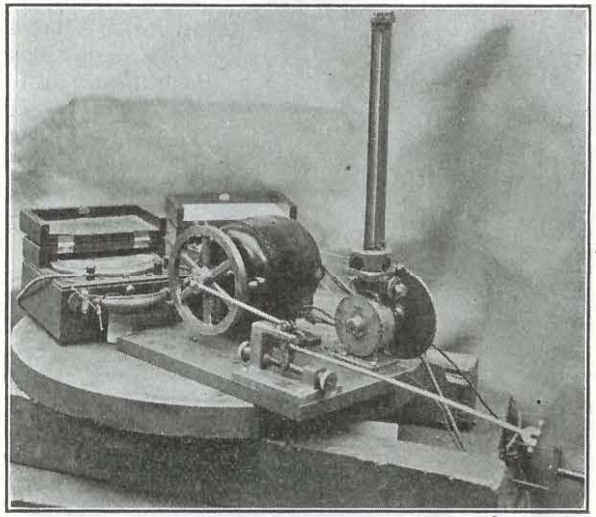

Fig. 46 Mechanical Vibrator Used to Measure Frequency and Energy Required to Maintain Vibration of Turbine Disk Wheels

116 When a wave train of constant amplitude is maintained in a disk wheel, the energy dissipated must equal the energy supplied, otherwise the amplitude would not remain constant. The appreciation of this fact makes it possible to find the actual supply of energy necessary to maintain a given wave train by a measurement of the dissipation. The energy supply in maintaining waves in a disk wheel cannot be directly measured as it comes from forces in the turbine during operation about which little quantitative information is available. The energy dissipation, however, may be directly measured by a test on the wheel outside of the turbine.



## Measurement of Rate of Energy Dissipation in a Vibrating Disk Wheel

117 The rate of energy dissipation was first measured by means of the apparatus shown in Fig. 46. A periodic force was applied transversely to the edge of the turbine wheel through the rod indirectly connected to the crankpin of the motor-driven flywheel. The speed of the motor was adjusted until the turbine wheel vibrated in resonance. The energy input to the motor is the sum of the energy required to maintain vibration plus all of the losses. This amount of energy was found to be about 80 watts for a 125-in. diameter turbine wheel vibrating in four nodes through a total amplitude of 3/8 in. Therefore the energy dissipated in the wheel cannot be over 80 watts, and if corrections are made for losses it appears to be of the order of magnitude of 50 watts.

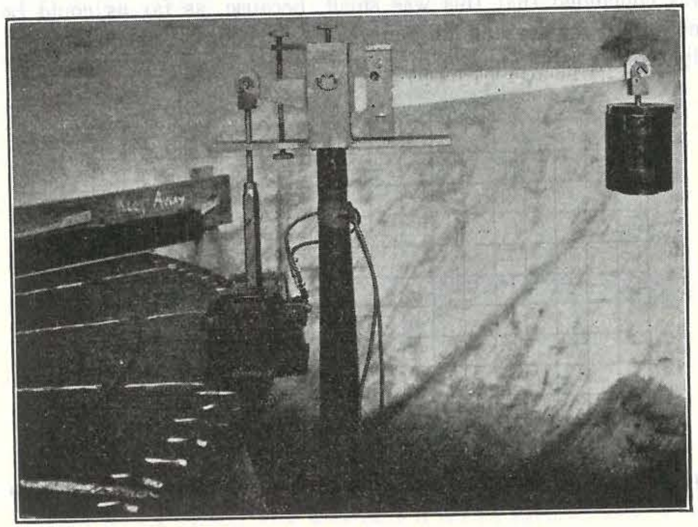

Fig. 47 Balance for Measuring the Energy Dissipated by the Vibration of a Turbine Wheel

118 In order to confirm these measurements another method was tried in which the vibration energy was supplied to the wheel by means of an electromagnet suspended from the end of a balance beam as shown in Fig. 47. The frequency of the alternating current supplied to the magnet was adjusted until the wheel vibrated in resonance. When the maximum possible amplitude of vibration, for a given amount of excitation of the magnet was obtained, the *average* pull on the magnet was weighed directly. Assuming that the pull varies according to a sine-squared law from zero to a maximum of twice the average value (that is, neglecting the

effect of variation of the air gap) the rate of energy supply to the wheel can be computed from the frequency and amplitude of motion.

119 The advantage of this method of test is that practically all losses in the apparatus used to make the measurement are eliminated. The energy supplied is calculated directly from the product of the applied force and the motion of the wheel edge at the point of application of the force, integrated over the required number of vibration cycles. The only source of external loss is through the pedestal on which the wheel is supported. It was concluded that this was small, because, as far as could be measured, for several different types of pedestal the wheel dissipated the same amount of energy.

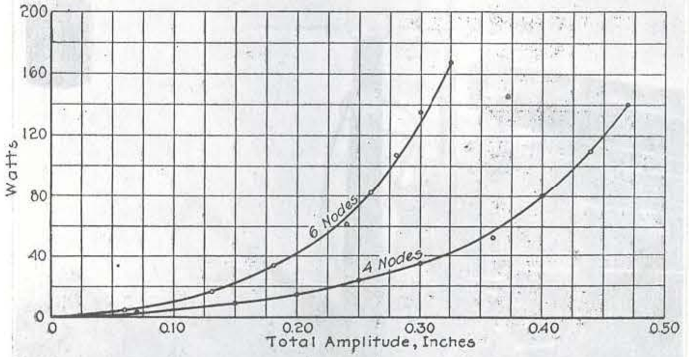


Fig. 48 Curves Showing Relation Between Energy Dissipated and Total Amplitude of Wave Train at Shroud Band

120 Fig. 48 shows curves of energy dissipation for 4- and for 6-node wave trains for a medium-thin last-stage disk wheel. The energy dissipation is seen to increase about as the square of the increase of amplitude of vibration for small amplitudes and at a more rapid rate for larger amplitudes. The energy dissipated by the air friction is comparatively small. The larger part of this energy is dissipated by internal friction within the steel itself due to the repeated bending.

121 The curves show how very small an amount of energy is required to maintain small amplitudes of vibration. For a total amplitude of 1/10 in. at the bucket tips, four watts will maintain a 4-node wave train, and ten watts will maintain a 6-node wave train at this amplitude. For a 1/4-in. amplitude, however, 24 watts is required for the 4-node wave train, and 73 watts for the 6-node wave train.



## Calculated Rate of Supply of Energy in Maintaining Waves in Turbine Wheels

122 The rate of supply of energy to turbine wheels in the maintaining of waves has been calculated for waves built up by the feathering action of steam on the wave forms in the buckets, described elsewhere, and for a stationary wave train built up by a spot of extra transverse pressure. These calculations show that for waves built up by bucket feathering the energy supply varies as the square of the amplitude, but for waves built up by a fixed pressure spot, which comes into action at critical speeds, the energy supply varies directly as the amplitude. The latter effects are consequently relatively large at small amplitudes as shown in Fig. 49 where curve II represents a larger energy supply than

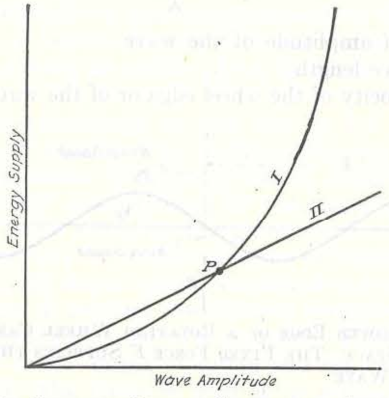


Fig. 49 Relation Between Energy Supply and Amplitude of Wave

(Curve I is for a traveling wave, while curve II is for a stationary wave. From the shapes of the curves it is seen that the latter may *originate* more easily.)

curve I for amplitudes below that corresponding to the point of intersection of the curves at *P*. According to this analysis a reason why stationary wave trains developed by pressure spots are more serious than traveling waves due to feathering is that the feathering cannot come into effective action until the wave train has reached a certain amplitude. To initiate a wave train the transverse pressure spot is the more effective.

123 Since stationary wave trains have been found to be the primary cause of turbine wheel failures, and since the building up of a stationary wave train is believed to be due to the second of the two causes of wave development presented, an indication of the manner in which a fixed force may supply energy continuously to a stationary wave train will be presented.

124 Fig. 50 represents the developed edge of a turbine wheel carrying a 4-node wave train stationary in space. The wheel edge is shown moving to the right with a velocity *V* and the wave is

traveling to the left in the wheel with the same velocity, so that it stands stationary in space. Assume a fixed force $F$ acting at a nodal point as shown. At this point a particle $P$ has a component of motion in the direction of this fixed force equal to $V_F$ in the figure. The product of this velocity and the force $F$ gives the rate at which work is done on the particle at this instant. Since the force is fixed in space it acts first on one particle and then on the next as the edge of the disk wheel moves along, but it does work continuously at this rate. This is therefore the rate at which work is supplied to the wheel in building and maintaining the wave train. It is easily shown for a sine wave that the value of this transverse velocity component is

$$V_F = \frac{2\pi V y_0}{\lambda} \quad . \; . \; . \; . \; . \; . \; . \; . \; . \; . \quad [11]$$

where $y_0$ = half amplitude of the wave
$\lambda$ = wave length
$V$ = velocity of the wheel edge or of the wave.

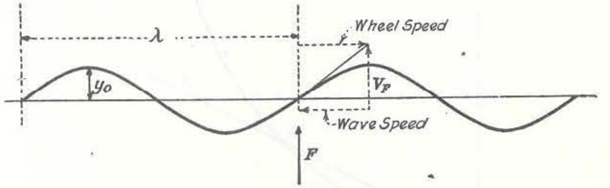


Fig. 50 Developed Edge of a Rotating Wheel Carrying a Wave, Stationary in Space. The Fixed Force $F$ Supplies the Energy that Maintains the Wave

Multiplying this velocity component by the force $F$, the work per second supplied to the wave is

$$W = \frac{2\pi V y_0 F}{\lambda} \quad . \; . \; . \; . \; . \; . \; . \; . \; . \quad [12]$$

In the tests described it was found that when $y_0 = \frac{1}{4}$ in., $W$ may be about 25 watts. Taking average values for $V$ and for $\lambda$, it is found from Equation [12] that a force $F$ of only a few pounds may supply the required 25 watts to the wheel.

125 This equation assumes the force to be concentrated at a point. If the force is distributed over half a wave length, the total force required will be somewhat larger.

### Utility and Limitations of Theoretical Design

126 As will appear subsequently, the actual process of design, aside from wheels of totally new characteristics, is a process of comparison with a carefully correlated catalog of all previous similar wheels. Such a process, checked by test, is thoroughly



logical, and proves to be satisfactory. In fact, for reasons which it is the intention of this section to make clear, comparative selection and test is the only satisfactory means of design known at this time. Such a method is comprehensive.

127 Even now shapes proposed by theoretical means come within the scope of the testing organization for verification. And new shapes in which theoretical analysis is most needed are, unfortunately, by that fact most subject to variation from theory and, therefore, especially need to be checked. Similarly the wheels of ordinary design which need least to be checked are most susceptible of calculation. It is entirely conceivable that at some future time material specifications can be made sufficiently rigid, machine work sufficiently exact, and methods of assembly so precise that every wheel can be constructed under conditions of uniformity sufficient for a general dependence on theory. At the present time it is unsafe to accept the successful calculation of nine out of ten wheels as evidence that the tenth wheel also will be correct.

128 It seems unnecessary to give here in full the elaborate theories developed during these investigations, but no adequate idea of the importance of actually testing wheels can be had without a thorough understanding of the scope of the various means of design tried. Space must be given, therefore, to a description of the theory and the tests made in its verification.

129 The simplest elastic system considered was a simple cantilever bar or beam of uniform cross-section, similar to a vibrating reed. This system is capable of a complete analytic treatment, from which the shape during vibration and the exact frequency can be obtained. The latter is what is of interest here and it may be written

$$F = \frac{1}{0.569\pi}\sqrt{g\frac{EI}{wl^4}} = \frac{3.18t}{l^2}\sqrt{\frac{E}{\delta}} \quad . \; . \; . \; . \quad [13]$$

in which

$F$ = frequency, cycles per sec.
$g$ = gravity, in. per sec. per sec.
$E$ = Young's modulus, lb. per sq. in.
$I$ = moment of inertia of cross-section, in.$^4$
$w$ = weight, lb. per in. length of bar
$l$ = length, in.
$t$ = thickness of rectangular bar, in.
$\delta$ = density of steel, lb. per cu. in.

130 In making a verification of this formula the dimensions and weight of the bar may be easily measured. For the same material the frequency should vary directly as the thickness and inversely as the square of the length. Fig. 51, curves $A$, $B$, and $C$ show a series of three tests made in an unsatisfactory attempt to prove this simple rule. All bars were of steel, but no particular

attention was paid to getting the same stock. The thickest bar $B$ actually shows a progressive deviation and suggests a variation with $l^{1.9}$ instead of $l^2$. The last test $C$ was made with two bars balanced like a fork, and while this test is better, it was not considered satisfactory for such a simple rule. Therefore, the test represented by curve $D$, Fig. 51, was made with a ½-in. square bar clamped directly to the heavy cast-iron floor of the shop. The modulus of elasticity was separately determined by a deflection test as a simple beam. The plotted points show agreement with the theoretical line over a 10 to 1 range of frequency varia-

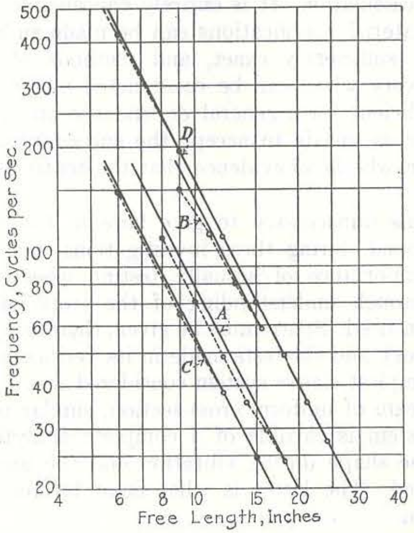


FIG. 51 VIBRATION OF STEEL BARS FIXED AT ONE END

(Full lines show theoretical variation of frequency. Broken lines show deviations in test.)

Bar A is 1.49 in. by 0.245 in.
Bar B is $\frac{7}{16}$ in. square.
Bar C is a 2-bar adjustable fork 0.86 in. by 0.180 in.
Bar D is ½ in. square.

tion, but even with this clamp the agreement starts to fall off at frequencies of about 300 cycles per second.

131 These simple tests have been described thus at length in order to show that laboratory care is necessary in the simplest cases in order to secure frequencies within one or two per cent of theoretical expectations.

132 Turning next to the question of disk wheels, the theory of vibration will be described. In previous sections it has been made clear that, so far as the frequency of various particles of the disk is concerned, it makes no difference whether the disk is subject to traveling waves or whether it vibrates with fixed nodes. In either case the natural frequency is the same, and all that is



essential is a solution capable of giving the frequency of vibration in each of the various natural types.

133 The problem of vibrating flat plates is thoroughly discussed by Lord Rayleigh,[1] but the complexity of a turbine wheel is somewhat beyond the formulas developed. Stodola[2] gives the formula for a flat circular plate based upon Kirchhoff's contribution to *Crelles Journal* in 1850, as

$$F=c\frac{t}{r^2}\sqrt{\frac{E}{\delta}} \quad . \quad . \quad . \quad . \quad . \quad [14]$$

in which $r$ = radius of plate, in.
$c$ = a constant depending on the nodal configuration and Poisson's ratio.

134 Stodola then goes on to examine at some length a method attributed to Ritz[3] in which the principle of least work is employed. Finally he selects a principle noted, in passing, by Rayleigh,[4] which proves to be the most practicable for use so far found.

135 Rayleigh's principle is that "The period calculated from any hypothetical type cannot exceed that belonging to the gravest normal type". (Section 89.)

136 The indicated method of calculation is to assume any hypothetical shape of deformation and calculate its true potential energy. Equate this to the maximum kinetic energy which the system would have if it could vibrate in the hypothetical shape, and the period so calculated cannot exceed the gravest natural period. Plainly the assumed shape which gives the longest period is nearest to the true condition.

137 In applying this method Stodola represents the wheel by a hyperbolic profile and makes ingenious allowances for the stiffness of the wheel rim and the weight of attached buckets. The hypothetical shape is sinusoidal about the circumference and along a radius it is an exponential curve. The potential and kinetic energies are formulated and the frequency of vibration expressed in terms of the above quantities and made a function of the exponent of the radial-deflection curve. Then by a differentiation the exponent leading to the minimum frequency (or gravest period) is selected. All of these steps are well justified on a theoretical basis. Rayleigh shows that there can be a considerable variation of the hypothetical from the true shape without greatly affecting the result. The exponential shape is, in itself, a reasonable approximation, but the Stodola method selects not only the

[1] The Theory of Sound, 1894.
[2] Ueber die Schwingungen von Dampfturbinen-Laufrädern. *Schweizerische Bauzeitung*, May 2, 1914.
[3] *Crelles Journal*, 1908.
[4] The Theory of Sound.

exponential shape but uses that exponent best suited to the case in hand.

138 In reviewing the various points of attack another plan may be mentioned which has not yet been worked out for application although its very simplicity commends it if it were as easy to construct the deflection curve for a disk wheel as for a rotor shaft. Since the idea of natural vibration supposes that the motion of all particles is both harmonic and isochronous, it follows that in the case of every particle, irrespective of displacement or time, the same ratio exists between weight and force per unit displacement, because all particles have the same period. In other words, if the material is all alike and all particles are of the same weight, the force per unit displacement is the same

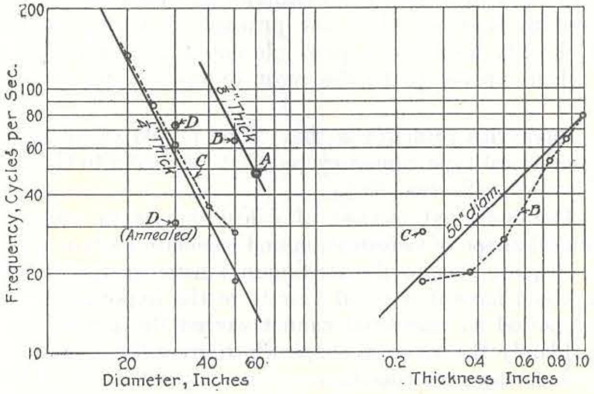


FIG. 52 4-NODE FREQUENCY OF FLAT CIRCULAR PLATES

(Full lines indicate theoretical relations. Broken lines show deviations in test. *A*, *B*, *C*, and *D* indicate the four different pieces of steel used.)

throughout the system. If the system is a shaft of uniform section or a plate of uniform thickness, the shape taken will always be such that at every point the unbalanced force (shear) on an element of length or surface will be strictly proportional to the deflection. In the case of a shaft or a reed where a deflection curve can be easily constructed by graphic processes, the indicated procedure is to assume a loading, construct the corresponding deflection curve and then alter the loading, and repeat until, by successive approximations, agreement of proportionality is secured between deflection and load.

139 Then the relation between change of shears in any part of the length, deflection and weight of the same section determines the frequency of the system. The method would be identical with that used in calculating the critical speed of rotation of a shaft. In the case of a vibrating disk wheel the shape within the rim



is such as would be taken under a loading per unit area proportional to the product of deflection and thickness.

140 The Rayleigh principle, mentioned above, has been developed along three different lines by the General Electric Com-

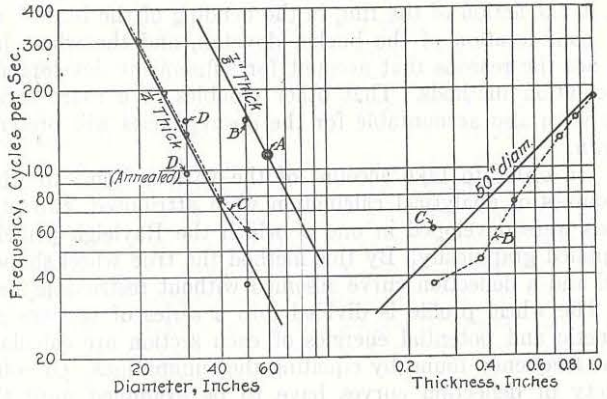


FIG. 53 6-NODE FREQUENCY OF FLAT CIRCULAR PLATES

(Full lines indicate theoretical relations. Broken lines show deviations in test. *A*, *B*, *C*, and *D* indicate the four different pieces of steel used.)

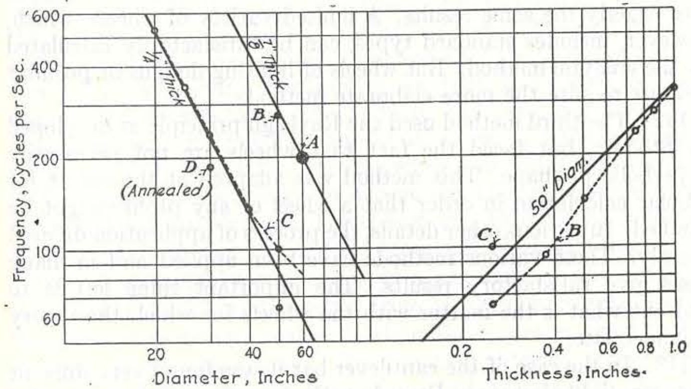


FIG. 54 8-NODE FREQUENCY OF FLAT CIRCULAR PLATES

(Full lines indicate theoretical relations. Broken lines show deviations in test. *A*, *B*, *C*, and *D* indicate the four different pieces of steel used.)

pany. The first frequency calculations were made by adapting the formulas of Stodola directly to the wheels in question. Charts of coefficients, tables, and printed forms were provided, and some hundreds of wheels were calculated in this way with good success. Usually wheels of nearly hyperbolic profile with standard rims and one row of short buckets or medium-length buckets were

easily and quickly computed by this method. There were, however, many important wheels with long buckets which failed to check the calculated frequencies. The protection of every wheel without exception was necessary. The difficulties were sought in variation of the actual profile from the assumed hyperbolic shape, in the action of the rim, in the bending of the bucket, and in the consideration of the bucket dovetail, and the wheel hub. These are the reasons that account for subsequent developments of calculation methods. That other troubles of a more serious nature were also accountable for the discrepancies will presently be shown.

141 In order to take account of the various items to which the troubles of analytical calculation were attributed, two other methods were developed in one of which the Rayleigh principle was applied graphically. By this method the true wheel shape is plotted and a deflection curve assumed without restrictions as to type. The wheel profile is divided into a series of sections and the kinetic and potential energies of each section are calculated and the frequency found by equating the summations. Of course a variety of deflection curves have to be examined until that giving the minimum frequency is found. Although standard calculation forms are used, the method is laborious as compared with analytic work, but it is comprehensive and sound in principle and with similar assumptions and limitations the two methods give exactly the same results. A limited variety of wheels, which, however, includes standard types, can be satisfactorily calculated by the analytic method. But wheels of limiting designs or peculiar contour require the more elaborate method.

142 The third method used the Rayleigh principle as developed by Stodola, but faced the fact that wheels are not necessarily hyperbolic in shape. This method was adapted at the outset for tabular calculation in order that a wheel of any profile might be handled. In various other details, the process of application differed slightly. These various methods have been applied and in many cases give satisfactory results. The important thing left is to find out what is the matter with the wheels for which the theory is inadequate.

143 In the case of the cantilever bar it was found very difficult to secure tight clamping. From bars the investigation was extended to flat circular plates. Kirchhoff's formula has already been cited, showing that for uniform material the frequency should vary in direct proportion to the thickness and in inverse proportion to the square of the radius. A series of tests was made on four plates to verify this rule.

144 The results are shown in Figs. 52, 53 and 54 for four, six, and eight nodes, respectively. The letters *A*, *B*, *C*, and *D*, are used to designate the four pieces of metal used. The left-hand curves show the variation of frequency due to change in diameter



for $\frac{7}{8}$ in. and $\frac{1}{4}$ in. thickness. The right-hand curves show the variation in frequency due to change of thickness for disks 50 in. in diameter. The *A* plate was tested but once and, as it agreed with the calculation, it is plotted as a master point from which the full lines representing theoretical relations are drawn. The *B* plate was 50 in. in diameter, and it was tested with thicknesses of 1 in., $\frac{7}{8}$, $\frac{3}{4}$, $\frac{1}{2}$, $\frac{3}{8}$ and $\frac{1}{4}$-in. Note that when 1 in. thick this wheel vibrated in substantial agreement with theory, but when $\frac{1}{2}$ in. thick the 4-node frequency was too low by 33 per cent. Note also that the *C* plate when 50 in. in diameter had a 4-node frequency more than 40 per cent too high, but as the diameter was subsequently reduced to 40, 30, 25, and 20 in. the agreement became better. It is also noteworthy that the agreement is better, the larger the number of nodes. Now note the plate *D*, 30 in. in diameter and $\frac{1}{4}$ in. thick. The expected 4-node frequency was 56. The measured value was 73, which is 30 per cent too high. This plate was subsequently "annealed" but the effect of this heat treatment was to reduce the 4-node frequency to 31, or less than half of its previous value, and 45 per cent too small as compared with expectations.

145 It had been noted that the same piece of metal — when altered in thickness or diameter — varied widely in the amount of its divergence from theory, far more widely then could be accounted for by variation in elastic modulus or density. It seemed probable that the condition of initial internal stress in the plate was changed by the removal of certain stressed portions of the material. It was difficult to account for the variations otherwise. Widely varying methods of support had failed to have appreciable effects. After the *D* plate had been put through a heat treatment intended to anneal it so as to remove internal stress, with the result that its frequency was reduced more than half, it appeared necessary to accept the idea of internal stress to explain the fluctuations. The easiest way to vary the condition of internal stress was to vary the temperature distribution.

146 A momentary application of a gas flame to the center of the disk *D* changed the frequency from 31 to 65, more than double, this frequency being measured while there was a temperature gradient along radial lines. The resulting internal stresses had the expected effect on the frequency which dropped as the temperature became more uniformly distributed throughout the disk. It is comforting to note that the fluctuations shown in the test plates of Figs. 52, 53 and 54 are extreme and appear much less severe in thicker plates. Most turbine wheels are of such proportions as to be less subject to variation of frequency on account of internal stress.

147 This series of simple experiments furnishes easily-understood evidence that one of the factors of prime importance

influencing the natural frequency of turbine wheel vibration is the exact condition of internal stress. It is also plain that on this account very refined methods of material treatment, machining, and assembly must be developed so that perfect uniformity can be assured before the practice of keeping a strict watch of every wheel for fluctuations from expected frequencies can be superseded. A very considerable amount of information has already

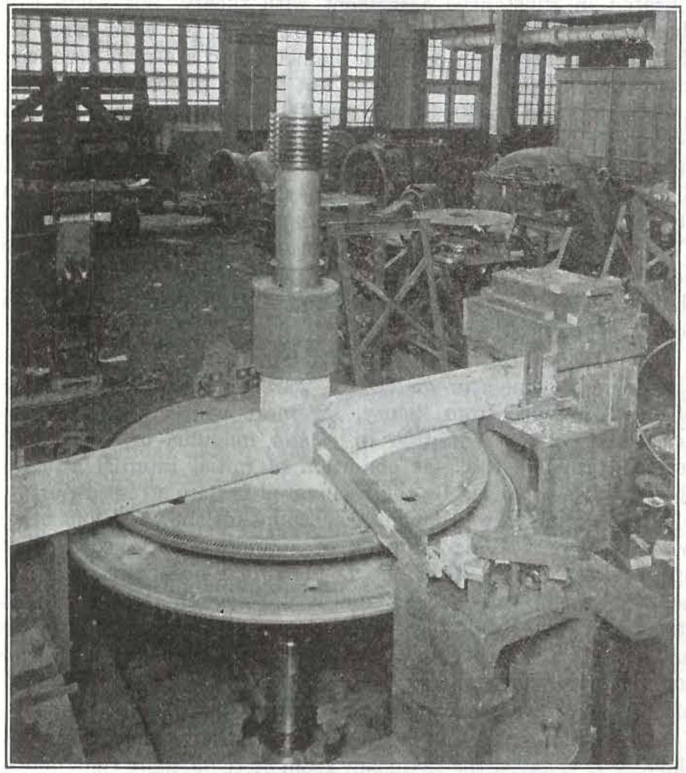

Fig. 55 Smoked-Glass Apparatus for Measuring Amplitude of Vibration of a Turbine Disk Wheel

been cataloged in explanation of frequency variations by the test methods in use.

## Measurement of Deflection Shape During Vibration

148 The determination of stresses due to vibration in a vibrating disk wheel is easily found from the deflection shape of the wheel. Once the shape is known, the stresses can be calculated by well-established formulas. It will be remembered that the



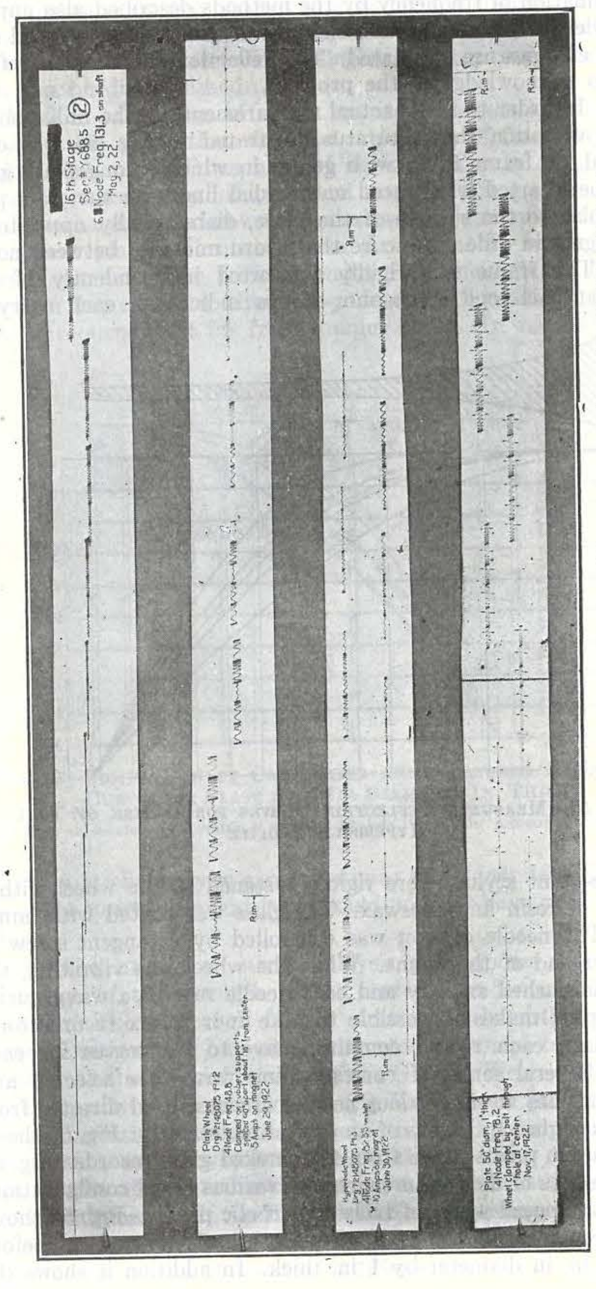

Fig. 56 Smoked-Glass Records of Amplitude of Vibration of Turbine Disk Wheels

(The full-sized record is measured with an optical micrometer.)

determination of frequency by the methods described also implies a knowledge of the deflection shape from which the potential and kinetic energies are calculated. The deflection shape is, therefore, vital to a knowledge of the problem.

149 In order to make actual measurements of the radial shape during vibration the apparatus illustrated in Fig. 55 was constructed. A frame fitted with guides in which a long plate glass could be inserted was placed on a radial line with the glass perpendicular to the surface of the plate, diametrically opposite to the magnet in order to secure the record mid-way between nodal radii. The frame was rigidly supported independently of the wheel at each end. Phosphor-bronze indicators, each carrying

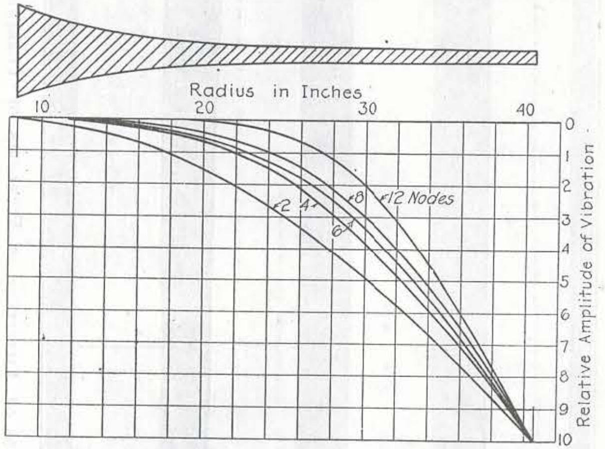


FIG. 57 MEASURED DEFLECTION CURVES FOR A DISK OF TRUE HYPERBOLIC PROFILE

a needle-point stylus, were rigidly fastened to the wheel with a mixture of resin and beeswax. The glass was coated with lampblack. The needle contact was controlled by a tangent screw at the outer end of the frame. While the wheel was vibrating, the plate was pushed radially and each needle records a wavy curve. A stop plug makes it possible to take four to six records on a single glass, each record containing five to ten waves for each needle. Several series of corresponding waves are selected and the amplitudes at the various needles are measured directly from the smoked glass by means of an optical micrometer. Fig. 56 shows photographic prints made from the smoked glass records. Fig. 57 shows a series of deflection curves for various nodal configurations made on a special wheel of truly hyperbolic profile. Fig. 58 shows the curves measured for the *B*-plate wheel referred to before when 50 in. in diameter by 1 in. thick. In addition it shows the



curves selected by the Stodola process for calculation. It will be noted that, while not exact in shape, the agreement is very good, in fact as far as the frequency calculation goes, very little difference can be distinguished.

150 The calculation of stress requires a better knowledge of the deflection shape than does the calculation of frequency. The smoked-glass method has been of especial value in furnishing a process for measuring deflection and thus indicating stress. When the deflection shape is known the stresses are given by the formulas for a bent plate deformed in two directions. It is not always possible to determine from resonant frequencies what type of vibration has caused a failure because more than one type may have a frequency not far from running speed. In case of certain

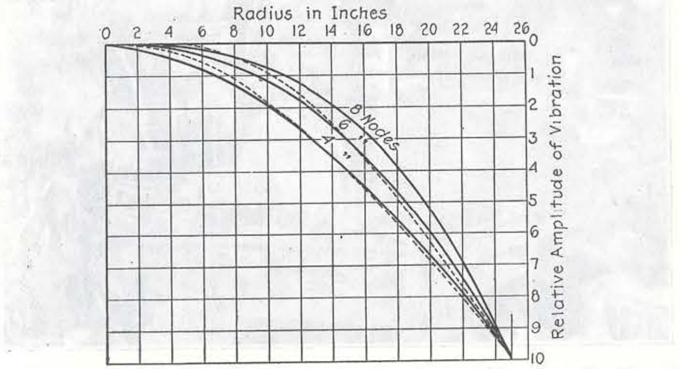


FIG. 58 COMPARISON OF CALCULATED AND MEASURED DEFLECTION CURVES FOR PLATE *B*, 50 IN. DIAM. x 1 IN. THICK

(Full lines calculated by formula $y = aR^s$. Broken lines show measured amplitudes.)

failures a stress analysis can sometimes show definitely that only one of the nearly resonant types could possibly have produced the failure.

151 The utility of stress analysis presupposes a complete knowledge of the strength of the material under conditions of vibration. The subject of fatigue stresses has been the object of many investigations, but usually devoted to completely reversed stresses. At the especial request of the General Electric Company, the University of Illinois Engineering Experiment Station undertook to investigate the case of repeated bending superposed on tension such as exists in a rotating turbine wheel. A variety of heat treatments of turbine steels have been investigated under these conditions and the first results of these experiments are described in Chapter IV of Bulletin 136 of the Engineering Experiment Station of the University of Illinois.

## PART III — METHODS OF DESIGN AND TESTING FOR THE PROTECTION OF TURBINE BUCKET WHEELS FROM AXIAL VIBRATION

152 The data obtained in the previous building and testing of wheels are utilized in the design of wheels having similar dimensions and forms. In the case of wheels already in service the speed coefficient may be used with considerable confidence in calculating critical speeds when the frequency of vibration of the wheel at rest is known. It is highly desirable, however, to check

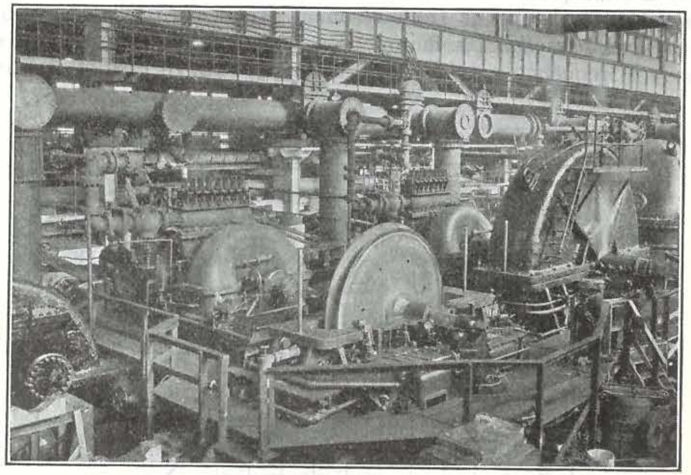

Fig. 59 Testing Machines for Determining the Vibration Characteristics of Turbine Wheels under Running Conditions

(The cover is removed from No. 1 machine to show test wheel within.)

results whenever possible by rotating the wheel in a wheel-testing machine.

### Turbine Wheel-Testing Machine

153 Fig. 59 is a photograph of the testing laboratory showing two complete wheel-testing machines. The smaller machine in the foreground has the cover removed. Fig. 60 is a photograph of the smaller machine with the cover on ready for test. This machine comprises a sort of bombproof chamber within which the wheel to be tested is operated. The upper half of the casing of this testing chamber is semicircular in shape, to constitute a hood over the wheel under test. This member is made of cast steel 8 in. thick for protection in case of accident to the test wheel.

154 The machine consists of a steam chamber in which the test wheel is mounted on the shaft alongside a heavy disk or



wheel as appears more clearly in Fig. 61. This shaft carrying the two wheels may be rotated at any required speed by means of a steam turbine. One pipe supplies steam to the casing when necessary while another pipe is utilized to convey the exhaust steam to the vacuum pump and condenser. An absolute pressure of about 4 lb. per sq. in. is maintained in the casing during standard tests.

155 The purpose of the steam in the wheel chamber circulating during the test is to keep the wheel relatively cool. If turbine wheels are rotated at high speed while surrounded by air at atmos-

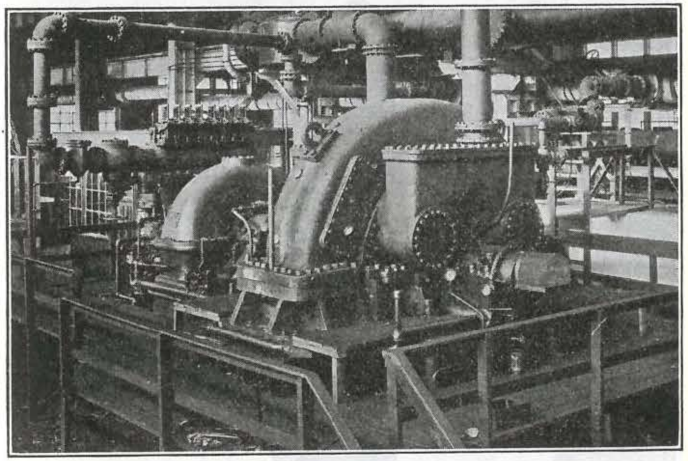

Fig. 60 No. 1 Wheel-Testing Machine Assembled with Cover on Ready for Test

pheric pressure so much heat is generated by the windage that the temperature of the wheel rises unduly. By circulating steam through the casing to the vacuum pump and condenser, the heating energy realized by rotation of the wheel is removed and the temperature of the wheel is maintained at the desired value.

### Use of the Oscillograph

156 In order to observe or record wave motions or vibrations which may occur in the wheel under test, a standard oscillograph is used, together with two sets of exploring coils suitably located within the wheel-testing machine. One set is stationary with respect to the wheel to be tested while the others rotate with the wheel. These exploring coils transmit to the oscillograph, which records them, electrical indications of the movement of the turbine wheel. The stationary coils indicate the movements of the wheel rim towards and away from the exploring coil as the wheel passes by the coil. The movable coil which rotates with the wheel,

on the other hand, records only the lateral motion of one given point in the wheel circumference. The records from these two coils disclose the nature of the wave phenomena developed in the wheel.

157 Exploring coils, located within the casing of the wheel-testing machine, are made steamproof by complete enclosure in a metal casing. Metal-cased wire is used for the electrical connections to the coils.

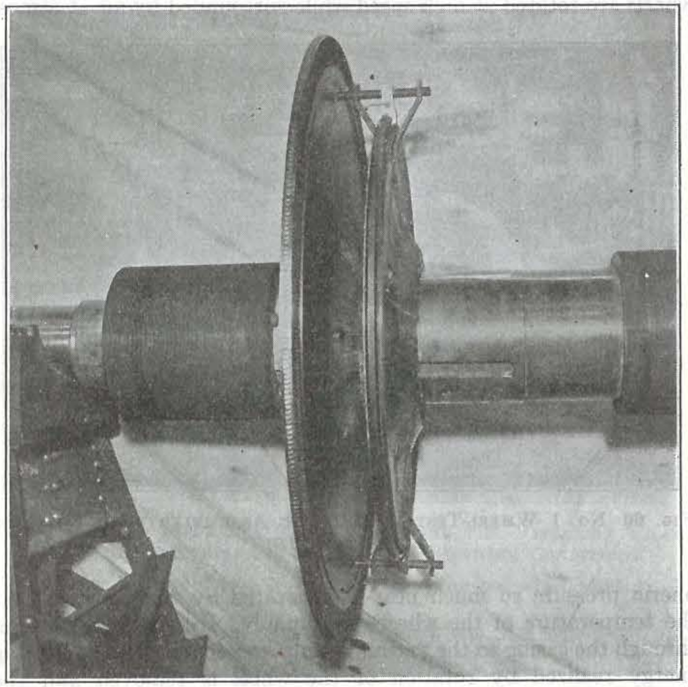

FIG. 61 TESTING-MACHINE SHAFT SHOWING EXPLORING COILS CARRIED ON AN ADJACENT AND RELATIVELY STIFF WHEEL

158 The rotating coils are carried in a tubular member fastened on the periphery of the relatively stiff coil-carrying wheel, always used in testing, whose vibration characteristics are so well known as not to be confused with those of the wheel under test. The tubular member is held parallel to the shaft and is adjusted in order to obtain the desired air gap between the coil and the test wheel. The metal-cased conductor from this coil is carried down the side of the coil-carrying wheel, brought out through the end of the hollow shaft, and connected to a collector ring. Another coil is placed 180 deg. away. This is used as a reserve and it serves also to maintain balance.



159 In some cases the wheel to be tested is of such dimensions that the vibration data are obtained from a point in the bucket region. In this case it is customary to silver-solder a small armature between two buckets in front of the rotating coil. Ordinarily, the air gap is between $\frac{1}{8}$ and $\frac{3}{8}$ in. on the large wheels and sometimes less on very small wheels.

160 The fixed exploring coil, already referred to, is suitably supported within the casing adjacent to the wheel rim, the metal-cased wire connection from this coil being brought outside the casing where electrical connection is made with the oscillograph.

## ELECTRICAL CONNECTIONS

161 The electrical connections of the various exploring coils, the electrical circuits of the amplifiers used, and of the oscillograph and of the exciting magnet used in test are shown diagrammatically in Fig. 62. The connections from the rotating coils lead out through the shaft and are connected with slip rings, the brushes from which lead to the primary of a transformer, a suitable source of direct current being connected in series. The development of brushes and rings which would satisfactorily collect the minute currents for the oscillograph was one of the many lesser achievements in the construction of this apparatus.

162 The stationary coil is connected through a switch to the primary of another transformer. The secondary windings from these transformers are connected respectively to suitable amplifier devices for magnifying the current fluctuation produced through the action of the test wheel on the exploring coils.

163 It will be understood that the current in one of these exploring coils, coming from the battery, develops a magnetic field, whose magnitude varies in accordance with the variation in the air gap between the adjacent parts of the wheel and the magnetic coil. In the case of the rotating coil the lateral vibration of the adjacent part of the wheel produces a change in magnetic reluctance. In the case of the stationary coil the change in magnetic reluctance is produced by variations in the distance of the wheel rim as it sweeps by the stationary coil. These variations in distance are due partly to irregularities in the structure of the wheel itself, and in case of wave phenomena, to lateral deflections of the wheel.

164 The current induced in the transformer secondary windings is amplified by vacuum tubes. This amplified fluctuating current is then led through another transformer, the secondaries of which lead to the oscillograph vibrators.

165 The oscillographs, indicated diagrammatically in the upper portion of Fig. 62 are standard instruments for producing and for recording images representing the fluctuations from instant to instant of electric currents. These images may be produced by

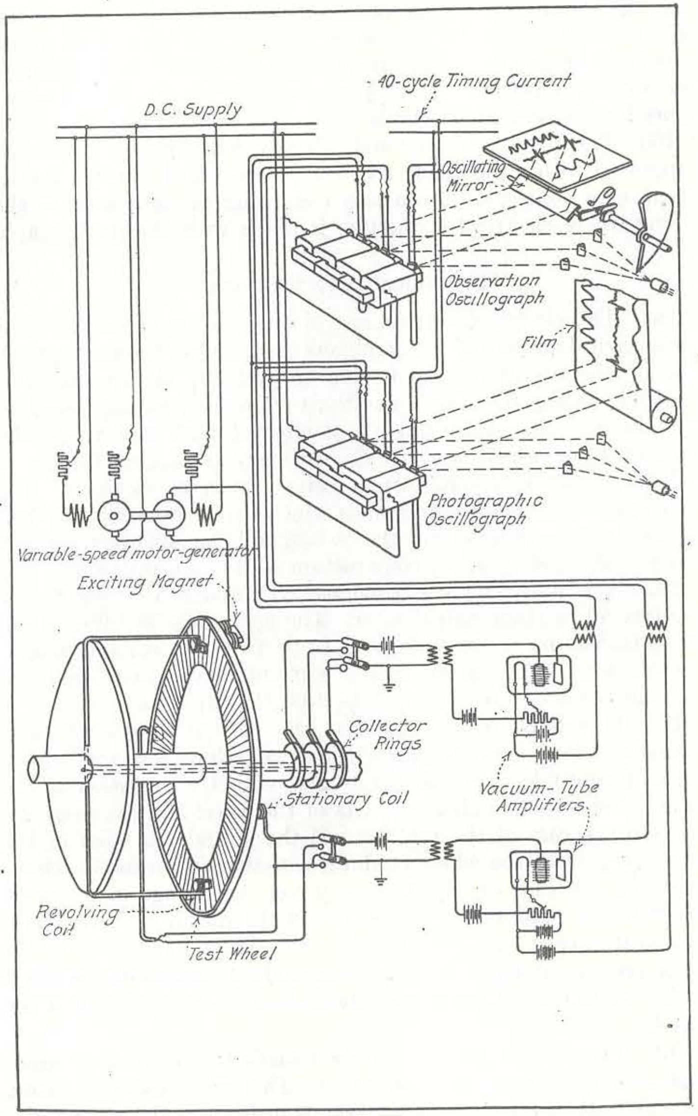


FIG. 62 ELECTRICAL CONNECTIONS FOR TESTING MACHINE

(Both revolving and stationary exploring coils are connected through vacuum-tube amplifiers to the observation and photographic oscillograph instruments. The exciting magnet receives current from a variable-speed motor-generator set.)



the trace of a point of light upon a ground glass or in a mirror, or images may be recorded permanently on a photographic film.

166 The fluctuating current is led to the bifilar suspension armature which carries a small mirror. This armature, which is located in a strong magnetic field, oscillates in proportion to the fluctuations in the current. Light from an arc lamp is transmitted through a lens and prisms to the mirror, and is reflected in turn to a suitable receiving surface. As used at present, this consists of an oscillating mirror, which is arranged to oscillate about a horizontal axis. The frequency of these oscillations is directly proportional to the speed of rotation of the test wheel. When this mirror is oscillated the wave forms are rendered visible, by reflection on a ground-glass receiving screen. The pivoted mirror is caused to oscillate by means of an arm which is held by a spring in contact with a cam. This cam is driven by a synchronous motor connected with the cam shaft. The synchronous motor receives the current from a small alternator driven directly by the shaft carrying the test wheel, the result being that the wave motions appear to be stationary instead of progressing across the field of vision.

167 The illumination of the screen is interrupted periodically by a shutter attached to the camshaft. During this dark period the cam returns the mirror to its initial position, and the light is then allowed to illuminate the screen again. Actually, only a spot of light is reflected on the screen, but owing to the rapid rotation of the camshaft and the phenomenon known as the "persistence of vision" the spot appears as a complete, more or less wavy line.

168 Three bifilar circuits carrying oscillating mirrors are used in the oscillograph, and are connected respectively to the coil circuits, whose wave forms it is desired either to observe or record. For reasons which will be explained, the oscillograph just referred to is used only for purposes of observation. One circuit receives its current indirectly from the rotating coil, another from the stationary coil and the third circuit receives its current from a source of 40-cycle alternating current, so that the indications produced from the operation of this latter member of the oscillograph serve as a time standard for the waves produced by the other two circuits.

169 A similar oscillograph, provided for the taking of photograph films, has all its circuits connected in multiple with those of the observation oscillograph just described, so that both receive currents of the same character. Inasmuch as the wave phenomena in the turbine wheel are transitory, the presence or absence of these wave phenomena is observed by inspection of the reflections from the oscillating mirror. When, however, the operator observes in the mirror of the observation oscillograph the occurrence of any particular wave phenomenon which it is desired to record, then at the desired instant, he signals an assistant. The latter

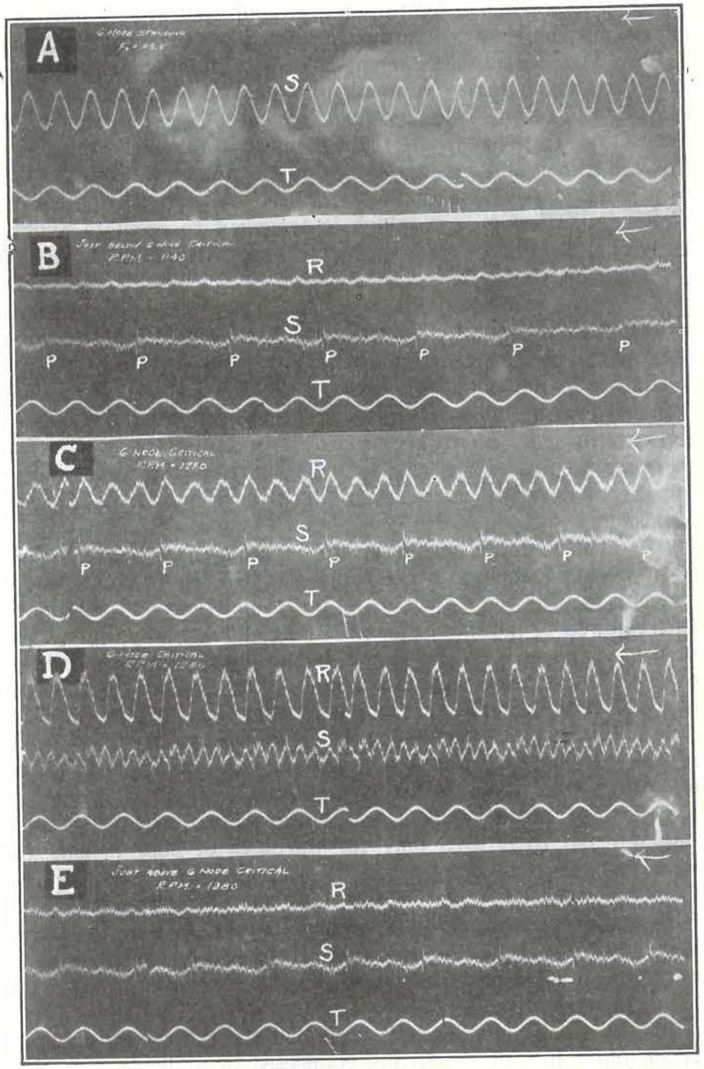


Fig. 63 Typical Oscillograph Records

$T$ is a 40-cycle timing wave
$S$ is the record made by the stationary coil
$R$ is the record made by the revolving coil
$A$ — Wheel stationary, 6-node vibration, 55.2 cycles per sec.
$B$ — 1140 r.p.m. The stationary coil $S$ gives the wheel "autograph." No vibration is indicated.
$C$ — 1250 r.p.m., 6-node critical speed. The revolving coil $R$ shows the backward wave in the wheel. The stationary coil $S$ still shows the wheel "autograph" since the wave is stationary in space.
$D$ — 1250 r.p.m., 6-node critical speed. A moment later than $C$. The stationary coil $S$ shows the development of the forward wave train at double the frequency shown by the backward wave train in the revolving coil record $R$.
$E$ — 1380 r.p.m. Wave phenomena have vanished. Compare with $B$.



thereupon operates the shutter of the recording oscillograph, causing an exposure to be made upon the film, which is immediately developed in the usual manner.

170 For the purpose of causing at will a vibration of the test wheel, a well-insulated electromagnet is located opposite the periphery of the wheel. This is usually located at the wheel rim in such a manner that the magnetic attraction will be in a direction parallel to the shaft. Electrical connections for this magnet are brought out from the shell and the alternating current is supplied from a variable-speed motor-generator set. Suitable rheostats enable frequencies varying through wide limits to be obtained. An electrical tachometer is used for measuring the speed of the motor-generator set. This is graduated in terms of frequency of the alternating current generated.

171 For the efficient use of the apparatus and the best organization of the work it has been found desirable to have two complete testing machines in use. While one is connected to the instrument chamber for the taking of records, the other machine is open or being assembled with the next wheel. All of the instruments for observation and control are grouped in the instrument room, including both the observation oscillograph and the photographic oscillograph. The necessary tachometers, control switches, rheostats, and the speaking tube to the turbine operator are within reach of the man in charge whose post is at the observation oscillograph. The developing room is immediately adjacent and equipped with all facilities for the quick development of records so that the operator can see, while the wheel remains at speed, whether his films successfully record the desired phenomena.

172 In testing a turbine wheel it is convenient first to determine the natural periods of vibration of the wheel when at rest, or standing still. This is often done with the wheel placed in the wheel-testing machine, previous to making the rotation test. The electromagnet already described, placed close to the rim of the wheel, receives the alternating current, thereby producing a pulsating magnetic pull on the wheel rim. Inasmuch as the magnet exerts a pull for each half wave of the alternating current the pulsating attraction exerted upon the wheel is numerically double that of the frequency of the alternating current. The nodal points on the wheel circumference are determined by observation and touch. Oscillograph records also corresponding to each particular number of nodes are taken and recorded.

## Oscillograph Films

173 Fig. 63 gives reproductions of oscillograph records of a wheel tested in the wheel-testing machine. At $A$ is the record taken to determine the standing frequency. The sine curve $T$ is the timing wave corresponding to the alternating current from supply mains. The curve $S$ is the record of the stationary exploring coil.

The vibration of the wheel as disclosed by this curve is caused by the action of the alternating-current magnet. When the frequency of the current in this magnet has been brought up to a value corresponding to one of the nodal frequencies of the wheel, a relatively large vibration of the wheel ensues, as is recorded on the film at *S*.

174 If now the test wheel be set in rotation, the current in the alternating-current magnet having been discontinued, the stationary exploring coil produces indications in the oscillograph even though no critical speed be obtained at which wave phenomena can devolop.

175 Indications of this character are shown in the film record reproduced at *B*. Here, as before, *T* is a timing wave. The irregular line *S* is the record of the stationary coil. This represents the wheel running rigidly and entirely free from wave phenomena. It will be noted that various peculiarities in the line repeat themselves at regular intervals, as, for example, at the points *P*. The distance between like points *P* represents the time of one revolution of the wheel. By comparison of the timing wave *T* which in this case was that of an alternating current of 40 cycles, the speed of rotation of the wheel can be accurately determined from the film, and may be checked up with the speed of the turbine driving the test wheel. Thus the line *S*, when its irregularities regularly repeat themselves, may well be referred to as the autograph of the wheel because it is different for every wheel tested.

176 The record at *R* on the oscillograph film *B* is that of the rotating coil. It will be noted that this record shows numerous fluctuations of very small amplitude. These are so small as to be negligible and show that there are no wave phenomena now present in the wheel.

177 Upon increasing the speed of the rotation of the wheel a point is finally reached where a wave train is markedly developed. This is clearly shown in film *C*. It will be observed that the record of the revolving coil is now in the form of a very marked wave, while the record of the stationary coil is substantially unchanged. This indicates the presence of a traveling wave of considerable magnitude in the turbine wheel. As the wave travels past the rotating coil the adjacent portion of the wheel oscillates backward and forward so as to produce the wave record *R*. It will now be noted that in the space of one revolution, as indicated by the distance between the points *P*, there occur three wave crests on each side of the zero line in the record of the rotating coil. Since there are three wave crests on each side of the wheel, making a total of six, this record indicates the presence of a 6-node wave train. Furthermore, the fact that the stationary coil gave the same record as in *B* indicates that the wave train in the wheel had no apparent effect on the stationary coil. It follows, therefore, that



the waves were stationary in space, and that the test wheel was running at a critical speed corresponding to a 6-node wave train.

178 The wheel was then permitted to run at this speed for a short time whereupon the oscillograph disclosed certain changes taking place in the wave phenomena, as shown in *D*. It will be noted here that the wave motion, as shown by the rotating coil record *R*, has become much more marked, and furthermore, that the stationary coil *S* has lost its original character and shows a series of waves, which upon examination will be found to be just twice as many in number as those now appearing in the rotating coil record *R*. This double frequency wave *S* represents a wave traveling forward in the wheel. The fact that the frequency shown on curve *S* is exactly twice the frequency of the curve R, confirms the fact that we are here dealing with a critical-speed phenomenon in which the backward-traveling wave is stationary in space and which has a forward wave superposed, upon it.

179 These speeds have been given the name " critical speeds " because nearly all vibration accidents have been associated with this condition, and also to distinguish them from other known resonant conditions.

180 At *E* is illustrated what happens upon slightly raising the speed of the turbine wheel over that corresponding to the record produced in *D*. It will be noted that the fixed coil record *S*, and the rotating coil *R* have returned practically to the condition exhibited in *B*, wherein the speed of the wheel was slightly below the critical speed.

181 One of the principal reasons for making rotation tests on the turbine wheel itself is that the various frequencies corresponding to vibration with radial nodes depend on the speed of rotation. It has been explained that the natural periods of vibration of the wheels increase as the wheel is set in rotation. This is readily shown by setting a wheel in resonant vibration with a definite number of nodes, by the use of the alternating current magnet with the wheel at rest, and then, while the wheel is in violent vibration, setting it in rotation. When the speed is sufficiently increased, vibration dies out. Upon decreasing the frequency of the magnet a sufficient amount, the wheel will again vibrate and a backward-traveling wave will develop having the same number of nodes as before. Furthermore, a similar but forward-traveling wave may be produced by sufficiently increasing the frequency of the magnet. When, therefore, for a given speed of rotation a certain frequency of impulse is applied to the wheel, less than that which would set the wheel into vibration if the wheel were at rest, a backward-traveling wave in the wheel may be induced, while if a certain higher frequency were applied, a wave traveling forward in the wheel at the same speed in the wheel as the backward-traveling wave, may be induced. These relationships have been explained in connection with the frequency-speed diagrams,

Figs. 32 and 35, and the unprovoked occurrence together with the recording of such waves has been noted.

## Demonstration of a Single Wave in the Testing Machine

182 The definite excitation of traveling waves under other than critical conditions is accomplished in the wheel-testing machine

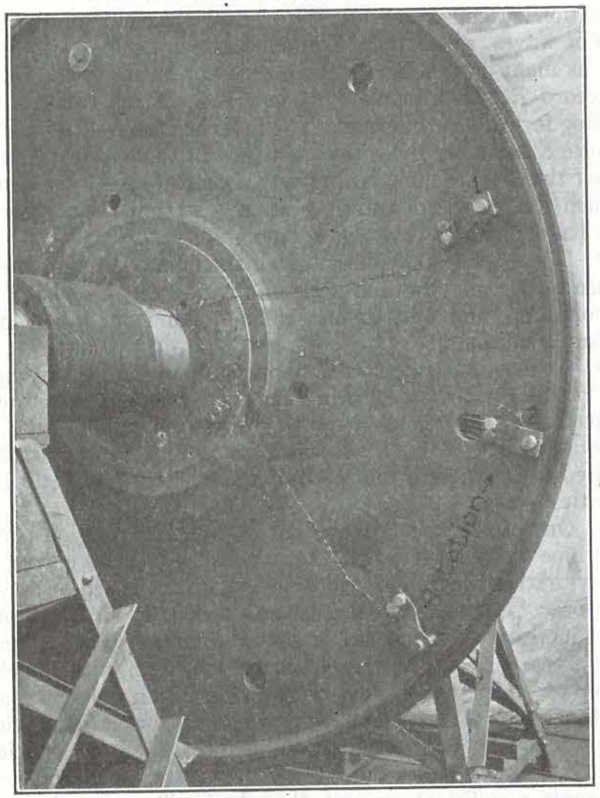

Fig. 64 Coil-Carrying Wheel Equipped with Three Coils Spaced 30 Deg. Apart for Detailed Analysis of Wave Motion

by means of the alternating-current magnet placed within the shell opposite the periphery of the wheel. The existence and direction of motion of such traveling waves in response to artificial excitation have been demonstrated further by a special series of tests using three oscillograph coils placed at intervals of 30 deg. on the carrier wheel as shown in Figs. 64 and 65.

183 In order to interpret the films properly it is necessary to note that the wave crest of the oscillograph record is normally



displaced one-quarter cycle from the crest of the wave in the wheel. Although each cycle on the film represents one vibration cycle, the actual motion of the wheel is recorded only indirectly by the film. The oscillograph records the induced voltage (with a negligible electrical lag) and the voltage reaches its maximum when the change of air gap is occurring most rapidly, that is, when the wheel is in its neutral position. Similarly, when the wheel pauses at its extreme position there is an instant of zero voltage

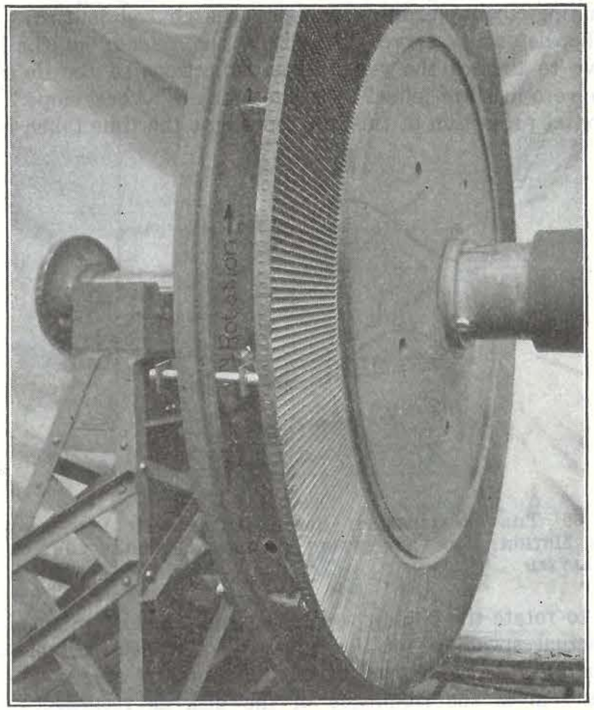


Fig. 65 Assembly of Test Wheel with Three Revolving Exploring Coils

during which the motion and induced voltage reverse their directions. Fig. 66 illustrates these relations. In all three coils the polarity was the same and also in each case the convention shown in Fig. 66 applies, namely, when the oscillograph record slopes downward to the right the wheel has a plus deformation and when it slopes upward to the right the deflection is minus.

184 The frequency-speed diagram for the wheel shown in Fig. 65 is given in Fig. 67. The curves for revolving coil frequencies and forward and backward wave frequencies as measured by a fixed coil are given for 4, 6, and 8 nodes.

185 At a speed of 5 r.p.s. a backward wave would register on a fixed coil a frequency of 50.5 cycles per second as shown at *A*, Fig. 67. The test shows the development of such a wave. The wheel was brought up to a speed of 5 r.p.s. and the alternating-current magnet frequency was made 50.5. A record represented by *B*, Fig. 68 was taken. The numbering of the coils is shown at *A*, No. 1 being the leading coil in every case. On the records of the coils, numbered 1, 2, and 3 to correspond with the diagram at *A*, is shown a deformity of the sine wave caused by the stationary alternating-current exciting magnet as each coil in turn passed it. This deformity serves to indicate the velocity of the wheel relative to that of the wave. The projections of the deformity on the zero axis are joined by a line marked " wheel slope ". The horizontal projection of this line represents the time taken by the

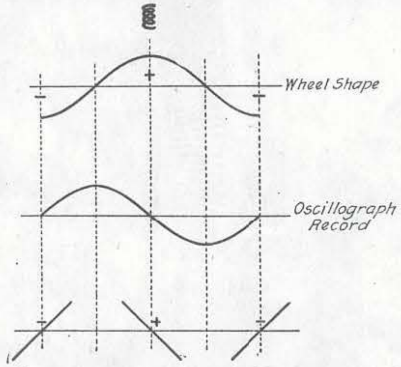


FIG. 66 THE OSCILLOGRAPH RECORD DIFFERS 90 DEG. FROM THE WHEEL MOTION. THE CONVENTION INDICATED ENABLES THE FILMS TO BE ANALYZED

wheel to rotate the 60 deg. separating coil No. 1 from coil No. 3. The actual speed of rotation given by the electric tachometer thus permits the calibration of the horizontal or time scale of the film and gives the frequency of the disk as recorded by the revolving coils as 60.5 as shown at *B*, Fig. 67.

186 The determinations of the direction and speed of the wave travel and the shape of the wave are made as follows: Since the coils are 30 deg. apart, the three traces on the record at *B*, Fig. 68, represent the motion of the edge of the wheel from its neutral position due to the wave motion at those three points on the circumference at any particular instant of time. Utilizing the convention explained in Fig. 66, the shape of the wheel at any instant is shown by the intersection of any vertical line with the three wave records. A series of vertical lines are drawn representing successive instants of time *a* to *j*. For each instant, a separate diagram is made at *C* and the corresponding deformation at each coil is noted.



187 An examination of Fig. 68, *a* to *j*, shows that a 4-node figure is definitely determined and at the same time the direction of rotation of the wave with respect to the three rotating coils is indicated. In this case it will be seen to be in a direction opposite to the rotation of the wheel itself. In order to determine the velocity of the wave, the points corresponding to the intersections of the zero line by the wave records of the three coils may be joined with a straight line as was done in the case of the wheel. This line is marked " wave slope " and the horizontal projection indicates the time taken for the wave to traverse 60 deg. or $\frac{1}{6}$ of a revolution. It is plain from Fig. 68 that the wave, which is

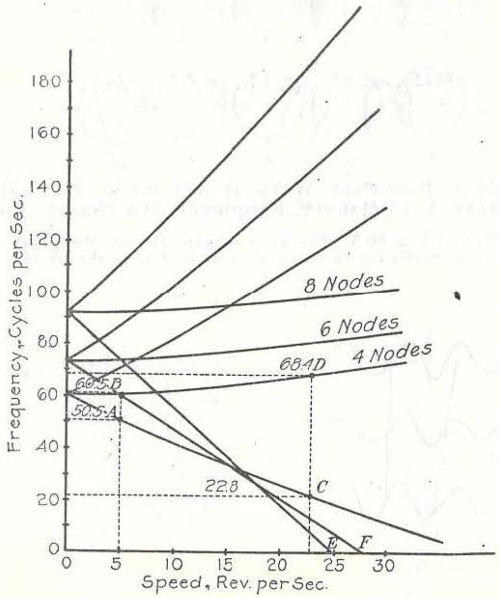


FIG. 67 FREQUENCY-SPEED DIAGRAM FOR 3-COIL TEST

traveling backward in the wheel, moves 60 deg. in a much shorter time than the wheel itself and hence the wave must actually be progressing backward in space.

188 A second test is illustrated in Fig. 69. The speed was brought up to 22.8 r.p.s. as shown in Fig. 67 at *C*. At this condition it will be noted that the frequency of the 4-node backward wave relative to a fixed point is exactly equal to the speed of rotation in r.p.s. This is called a minor resonant speed for 4 nodes, a condition which will be discussed presently. An examination of the records in the same way as described for Fig. 68 shows a 4-node backward wave with a speed of rotation greater than the wheel speed so that the wave is progressing backward in space. The

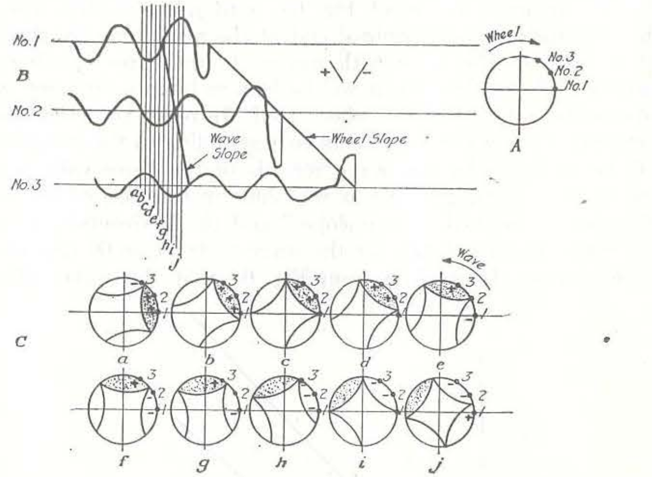


**Fig. 68 4-Node Backward Wave at 300 R.P.M. Maintained by a Stationary A. C. Magnet, Frequency 50.5 (See A, Fig. 67)**

(The disk frequency is 60.5. The wave velocity exceeds the wheel velocity and therefore the wave travels backward in space as well as in the wheel.)

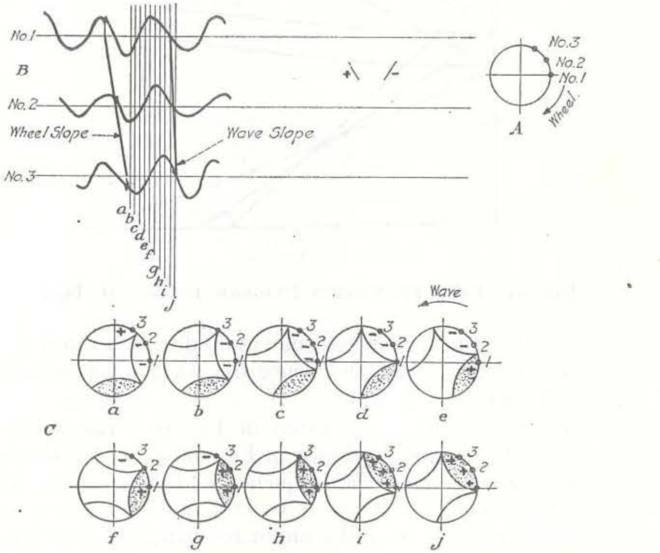


**Fig. 69 4-Node Backward Wave at 1370 R.P.M., The First Minor Resonance (See C, Fig. 67)**

(The wave velocity exceeds the wheel velocity and therefore the wave travels backward in space as well as in the wheel.)



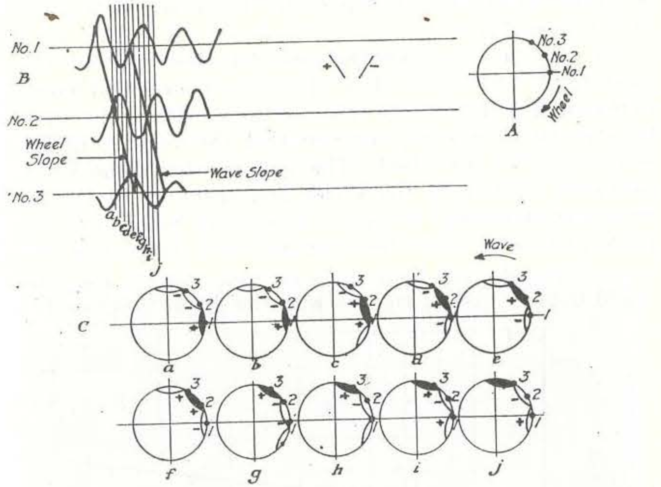


**Fig. 70 8-Node Critical Speed at 1480 R.P.M. (See *E*, Fig. 67)**

(The backward wave velocity in the wheel equals the forward wheel velocity, so that the wave is stationary in space.)

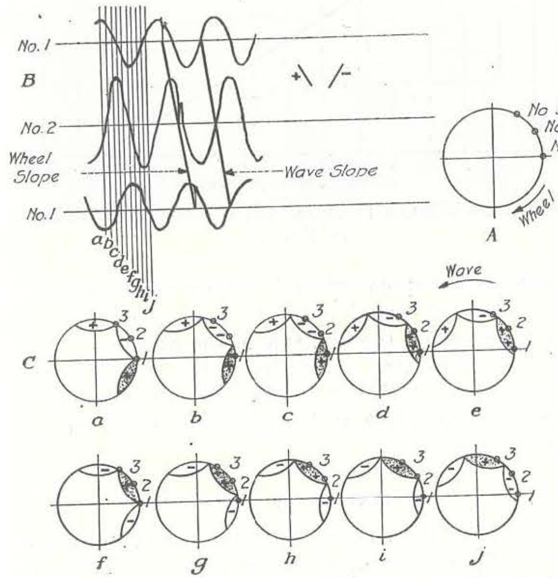


**Fig. 71 6-Node Critical Speed at 1660 R.P.M. (See *F*, Fig. 67)**

(The backward wave velocity in the wheel equals the forward wheel velocity, so that the wave is stationary in space. )

frequency of the wheel relative to the rotating coils is 68.4 as shown at *D*, Fig. 67.

189 Fig. 70 represents the condition observed with the wheel rotating at 24.7 r.p.s. The phenomenon recorded corresponds to the point *E*, Fig. 67. In this case the wheel and the wave slopes have exactly the same angle so that the velocity of the wave equals that of the wheel. The analysis shows the wave to be backward relative to the wheel, and therefore in this case the wave was stationary in space. This as an 8-node critical speed. Each particle of the wheel circumference traversing this wave passes through four high spots on each side of the wheel and would thus, in case of contact with the diaphragm, result in rub-

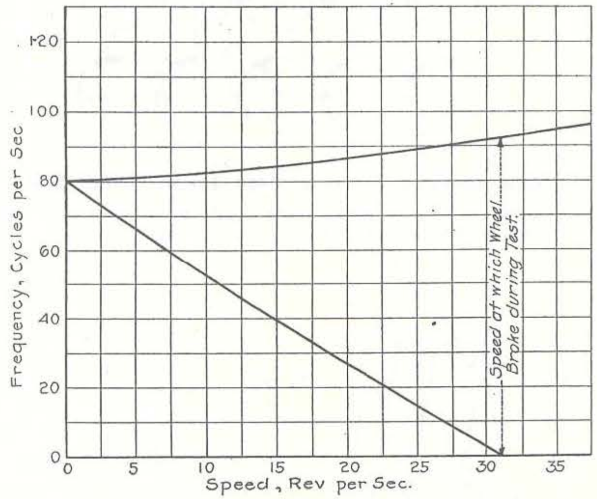


Fig. 72 Frequency-Speed Diagram for 6 Nodes for Wheel Shown in Fig. 73

bing at 4 equidistant spots. Should rubbing occur on the opposite side of the wheel, it would result in 4 equidistant spots intermediate to the spots on the first side of the wheel.

190 Fig. 71 illustrates a 6-node critical speed occurring at 27.7 r.p.s. as shown at *F*, Fig. 67. In this case if rubbing should occur it would take place at 3 equidistant spots on each side, with alternate spacing.

## Confirmation of Breakages

191 It has been the custom, since the first vibration troubles were successfully explained, to make tests in all the cases of serious wheel or bucket trouble. Either a duplicate wheel is made or, if in a suitable condition, the wheel that suffered the injury, is itself used. The first test made in this manner was of the 17th



stage wheel of a 20,000-kw., 1500-r.p.m., 23-stage turbine which developed a traveling wave, as determined by oscillograph record, while under load in the operating turbine.

192 By revolving this wheel in the wheel-testing machine and applying the alternating-current magnet at its rim, the fundamental relations between the applied force and the responding vibration were determined. Using this information together with that obtained from the oscillograms taken under load conditions it was possible to construct Fig. 72. From this diagram a prediction could be made that at a speed of about 31 r.p.s. a contin-

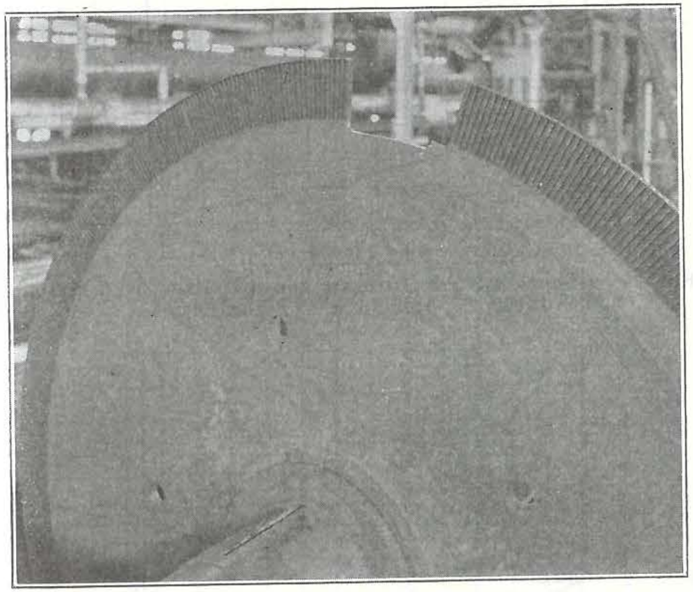

Fig. 73 Turbine Wheel Broken in Testing Machine by Rotation at 6-Node Critical Speed. 17th Stage of 20,000-Kw. 1500-R.P.M. 23-Stage Turbine

uous force applied at a fixed point in space should throw the wheel into vibration with a 6-node stationary wave.

193 This prediction was tested as follows: With no current on the magnet the wheel speed was brought up through the predicted danger point and nothing was observed. The wheel was then slowed down somewhat below that point and the electromagnet was energized by direct current furnishing a fixed force. The speed was then raised almost to the predicted point, when the wheel developed a violent lateral vibration, rubbing off the pole piece of the exciting magnet and very soon tearing out the buckets from the wheel rim as shown in Fig. 73, a striking corroboration

of the prediction and an indication of the seriousness of operating at a critical speed.

194 The 11th-stage wheel of a 12-stage double-flow turbine in a 30,000-kw., 1500-r.p.m. machine had failed in a manner illustrated in Fig. 5. A duplicate wheel in the same turbine was

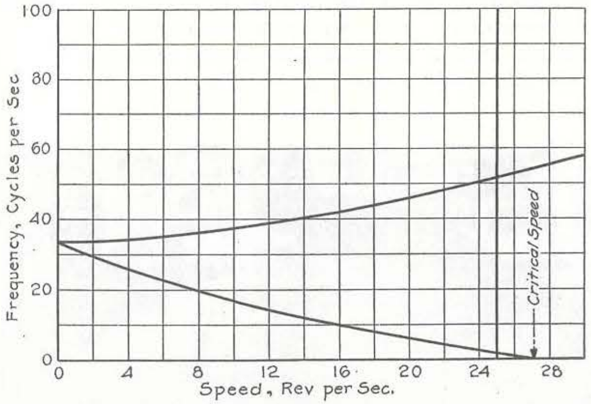


**Fig. 74 Frequency-Speed Diagram for 4 Nodes. 11th Stage of 30,000-Kw. 1500-R.p.m. 12-Stage Turbine**

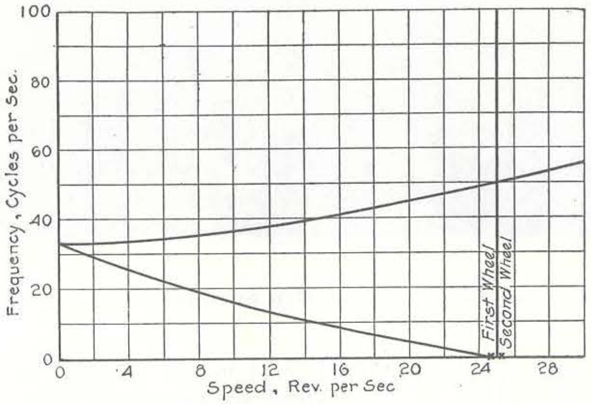


**Fig. 75 Frequency-Speed Diagram for 4 Nodes. 11th Stage of 30,000-Kw. 1500-R.p.m. 12-Stage Turbine**

used for the purpose of making rotation tests. Both wheels had been in service and operated several years. Fig. 74 was made from data obtained from a test of the duplicate wheel. A 4-node critical speed due to a wave stationary in space is indicated at 27 r.p.s. just above the running speed of 25 r.p.s. It is not unlikely that the wheel that failed differed slightly, due to minor variations in machining, from the duplicate which was tested. Such a variation



might very probably bring its actual wave speed equal to running speed.

195 The wheel in the testing machine, when operated at the speed of the 4-node wave stationary in space, developed heavy vibration and threw off a piece of shroud band which previously had been slightly damaged in the accident to the broken wheel. It was observed that the diaphragm opposite the wheel in service which had failed, had been deeply cut by the shroud band of the wheel, whereas the wheel used in test had no such contact with the diaphragm.

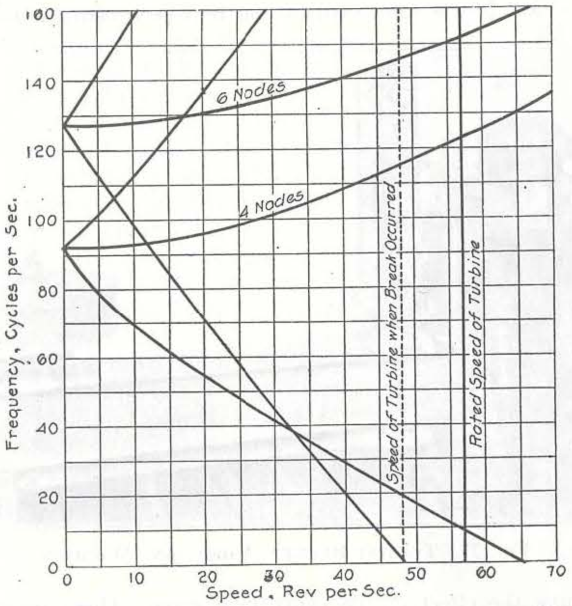


**Fig. 76 Frequency-Speed Diagram for 3d Stage of 6000-Hp. Marine Turbine**

196 The natural frequencies taken on another pair of duplicate wheels and also of the same dimensions as the former wheel yielded results from which Fig. 75 is plotted. Here, close coincidence with the operating speed is indicated. That trouble had not occurred with these wheels is attributed to the absence of sufficient exciting force.

197 Still another duplicate wheel in another station had been known repeatedly to rub the diaphragm. All wheels of this type were immediately replaced as soon as the cause of the accident to the broken wheel became known.

198 Another typical case was that of a third-stage wheel of a 6000-hp. turbine used for ship propulsion. Fig. 7 is a portion

of the broken wheel showing two breaks which occurred through steam pressure equalizing holes. Figs. 8 and 9 show the development of fatigue fractures which originated, in each case, at each side of a hole. Previous to the failure of the wheel under investigation, the ship had been continuously cruising with a propeller speed of 84 r.p.m. owing to foggy weather. At the time of the accident the tachometer showed 84 r.p.m. of the propeller, corresponding to a turbine speed of 48 r.p.s.

199 The vibration tests were carried out on a duplicate wheel made from the same drawing as the broken one. This wheel was placed upon its own shaft in order to determine the standing frequencies. It was afterward run in the wheel-testing machine to

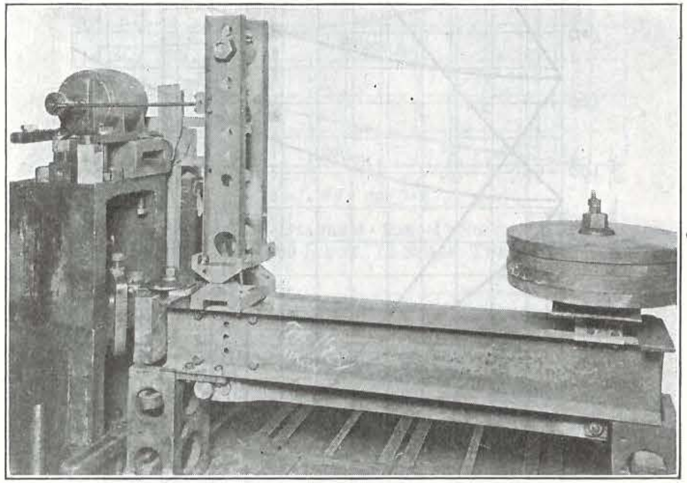

Fig. 77 Turbine-Bucket Vibration Machine

determine the effect of the centrifugal force. These results are plotted on Fig. 76 which shows the 4- and 6-node frequencies. The 6-node critical speed occurred at 48 r.p.s., which was just the speed at which failure actually took place, namely, 84 r.p.m. of the propeller and 48 r.p.s. of the turbine.

200 At this speed, corresponding to the 6-node critical speed of the wheel, the stationary wave could be maintained so that, if this speed were held constant for a considerable time, the wave could develop a large amplitude of vibration and the wheel undergo a series of repeated, or fatigue, stresses which would eventually cause failure. A substitute wheel, designed to replace the broken wheel, was increased in thickness 100 per cent, so that the 4-node critical speed was raised to a point 92 per cent and the 6-node speed to a point approximately 50 per cent above the normal running speed.



## Fatigue Cracks in Bucket Dovetails

201 The breaking of bucket dovetails on the last stage of a 30,000-kw., 1800-r.p.m., 17-stage turbine was attributed to wave phenomena, in which the speed of the backward wave was equal to that of the speed of rotation. The nature of the break indicated that axial motion of the bucket system had been occurring for a considerable time before rupture took place.

202 The break occurred at the dovetail and since the vibrational stresses were superposed upon the centrifugal stresses, it was realized that a bucket, to be broken in a similar manner, should be subjected to both kinds of stress. In order to accomplish this, a special machine shown in Fig. 77 was devised, in which it was possible to apply the tension load to the bucket dovetail which it would have in a wheel running at normal speed. The vertical member shown at the left hand end is allowed to rock around the metal ribbons at its base. The heavy weights together with the lever shown below the base create the necessary tension force on the specimen. The upper end of the bucket is secured to the top end of the vertical member and the section of the wheel at the lower end is secured to the block sliding in the base. This block is then pulled down by the lever already referred to. Fig. 78 illustrates the bucket and wheel section assembled in the vertical rocking member. This arrangement allows lateral motion of the bucket and wheel with a minimum variation of tension during the swing. The vertical rocking member is vibrated by means of a motor-driven eccentric to which a revolution counter is attached.

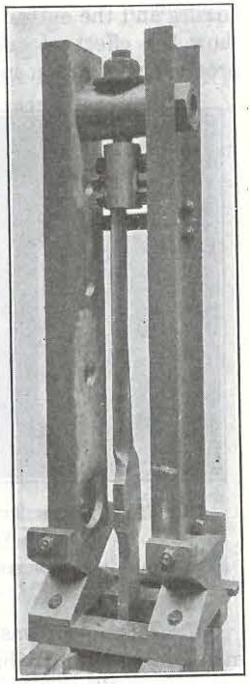

Fig. 78 Detailed View of Bucket in Vibrating Machine

203 Fig. 79 shows at *B* and *C* breakages at the lower tangs of two buckets that occurred in service after two months' operation. The specimen shown at *A* was a duplicate bucket fitted together with a cross-section of the wheel machined to the same width as the bucket. A smooth crack will be noticed in the lower left-hand tang of the bucket. This was produced by means of the apparatus explained, after six million repetitions of the stress.

204 In the case of the wheel breaking in service no rubbing had been encountered. The amplitude of motion used in the test

machine was equal to one-half the clearance between the shroud band and diaphragm.

205 Upon separating the pieces shown at *A* the surface conditions of the fracture were found to be of the same nature as in the case of the service wheel.

## Effect of Forced Rubbing on Vibration at Speeds Other than Wheel Critical Speed

206 Since the efficiency of a steam turbine depends to a certain extent upon the clearance between the discharge edges of the nozzles and the entrance edges of the buckets, a test was made to show the effect of rubbing of the wheels on the diaphragm in producing vibration in the wheels.

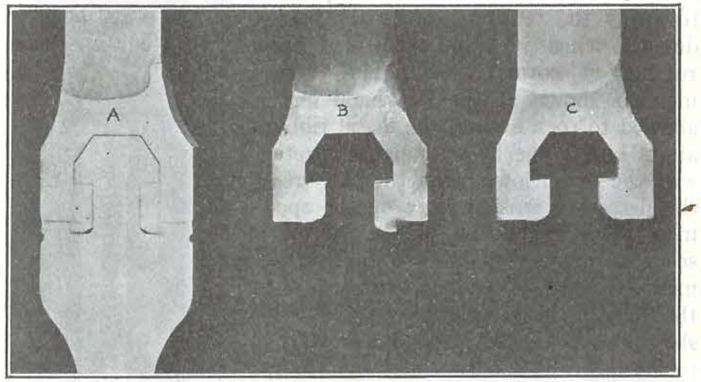


**Fig. 79 Broken Turbine-Bucket Dovetails. 17th Stage of 30,000-Kw. 1800-R.p.m. 17-Stage Turbine**

(The break at *A* was produced in the vibrating machine, Fig. 77. Breaks *B* and *C* occurred in service.)

207 The object was to determine the possibility of causing and maintaining lateral vibration in a steam-turbine wheel by exerting pressure at the rim by means of a rubbing block and also with a steam nozzle. The tests were made on a large-diameter wheel, shown in Fig. 80, of unusual flexibility. The wheel had been in service (3d stage) in a 20,000-kw., 1200-r.p.m., 9-stage turbine.

208 The wheel-testing machine was used and a brake shoe was arranged for the purpose of causing contact. This method of applying pressure would be somewhat similar to the worst condition that could occur in a turbine, namely, rubbing at one place. The brake shoe and method of applying pressure is illustrated in Fig. 81. A rope was attached to the external lever and extended to the instrument room. Rubbing pressure could be brought about at will during observation of wheel vibration. Visual observation



was made on the ground-glass screen of the oscillograph. When the revolving recording coil showed either vibration or erratic scratching the shoe was quickly released and the wheel permitted to run free. This was repeated for various speeds of the wheel. Fig. 82 is a diagram of the complete information obtained from this test. Standing frequencies were obtained in the usual manner, films recording critical and minor resonant speeds (which will be discussed presently) are indicated by stars. As an alternative to the rubbing shoe a steam nozzle projecting high-pressure steam in an axial direction could be substituted.

209 It was noticed that when running at a minor resonant speed the wheel forms a definite wave due to the shoe contact but when not at such a speed the record is very erratic, showing that no definite form of vibration took place. Fig. 83 shows the record made during the rubbing period at other than a critical speed. It was impossible to push the wheel into a definite state of vibration even though a large pressure was exerted.

210 When the shoe was released the wheel usually assumed a 4-node vibration even though it previously had been vibrating in a more complex type. The 4-node type is the one most frequently assumed when a standing wheel is struck a single blow. After the 4-node vibration died out the wheel always ran true again except when running at some critical or minor resonant speed. Figs. 83, 84, and 85, show the wheel in the three states previously referred to at other than a critical or resonant speed, Fig. 83 being made with the shoe in contact, Fig. 84 just after release, and Fig. 85 about a minute later.

211 No tests were made with the rubbing bar at critical speeds, the steam jet showing that amplitudes could be built up to a large amount at these speeds. The wheel when examined after test had the rim blued by heat developed from friction.

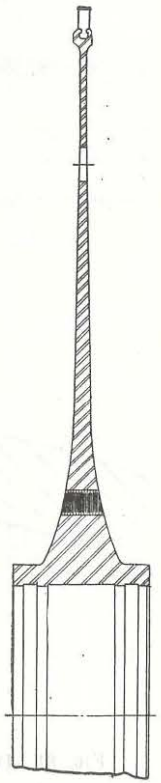

**Fig. 80 Turbine Wheel Used in Rubbing Test**

## Effect of Temperature on Frequency of Turbine Wheels

212 Fig. 86 shows the lowering of frequency due to differential temperature. These tests were made in the wheel-testing machine, a gas flame being projected on the rim of the wheel during slow rotation. Thermocouples placed at the rim and the hub, and connected to the collector rings at the end of the shaft, were used

for the purpose of measuring the temperature at these two points. It is seen that there are only slight changes in the 2- and 4-node frequencies, due to a temperature difference of 200 deg. fahr., but greater differences are noticeable with six and eight nodes. These temperature differences are only possible momentarily in service

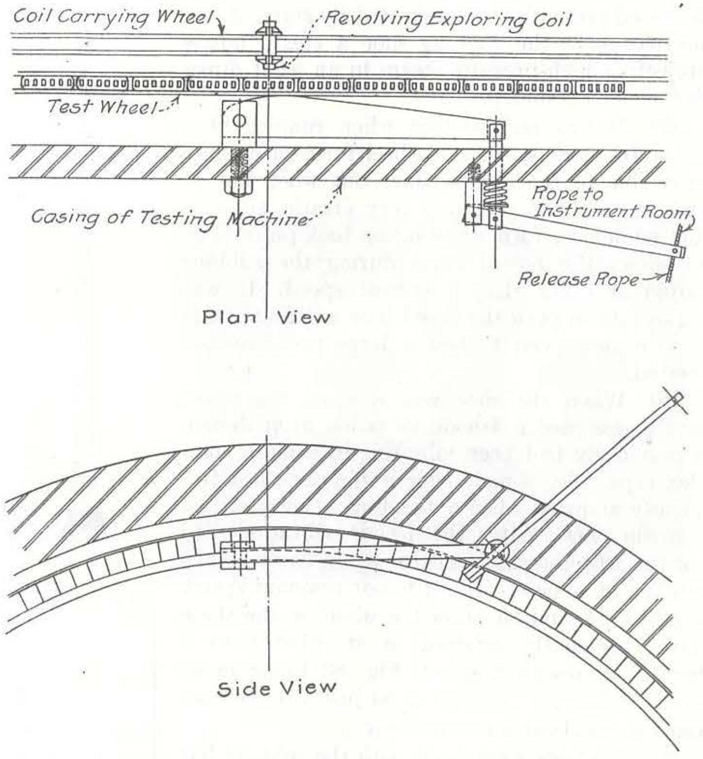


Fig. 81 Detail of Rubbing Bar Used in Rubbing Test

and wheels at the hottest end of the turbine are usually the only ones affected in this way.

213 Actual measurements of temperature differences in operating turbines between the inner and outer diameters of the diaphragm have not shown anything like these temperature differences. However, it is believed that sufficient margins for variations of this character have been allowed for in the safety limits adopted which prescribe appropriate margins from normal operating speeds for each of the critical speeds.



## Effect of Bucket Tightness on Frequency and Critical Speed

214 The method of fastening buckets to turbine wheels has a very important bearing on frequencies obtained under running

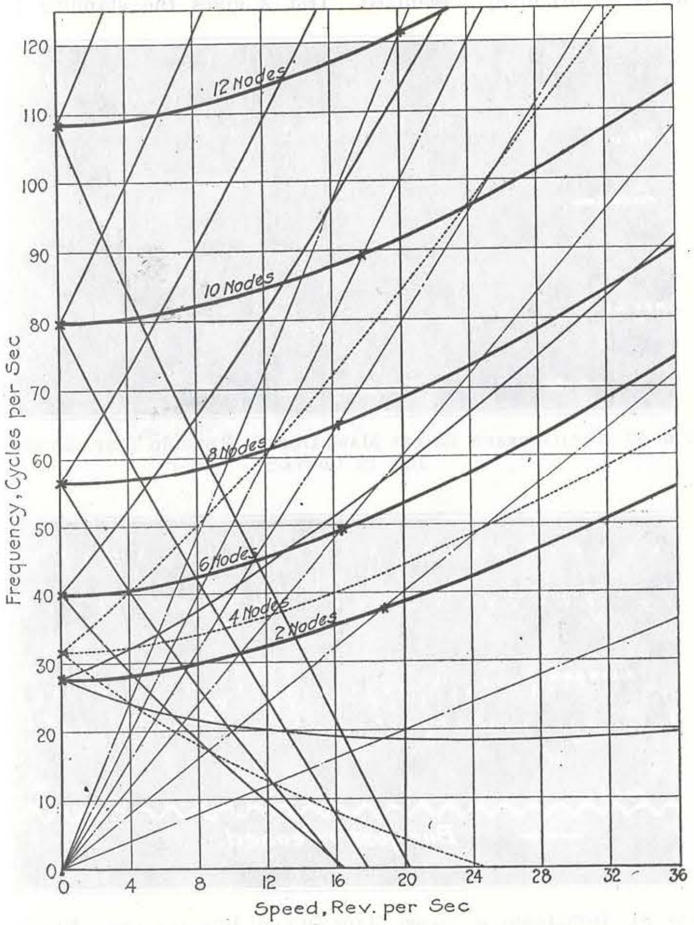


Fig. 82 Frequency-Speed Diagram for Wheel Used in Rubbing Test

conditions. This effect has been very definitely shown from many tests of which a few typical examples are given.

215 Several wheels were built with bucket dovetails of the type to be inserted in a grooved rim. An attempt was made to obtain a tight fit by initial tension in the neck of the bucket instead of forcing the dovetail head into the groove, which is

the common practice. Extra precautions were taken in an attempt to have an initial stress on the narrow neck section of the bucket sufficient to keep this fit tight under running conditions. Fig. 87 shows the various standing frequencies. Test 1 was made on the wheel as originally assembled. Test 2 gives the standing fre-

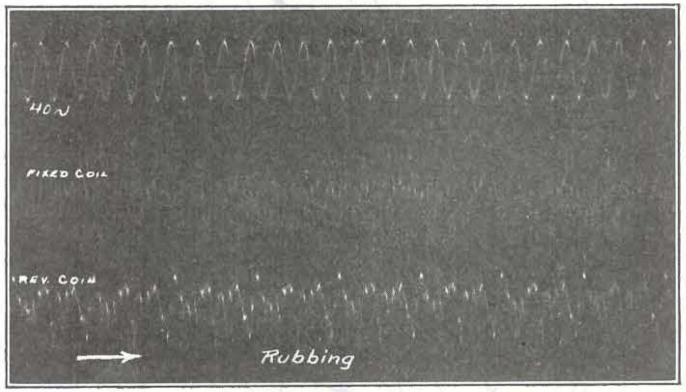


FIG. 83 OSCILLOGRAPH RECORD MADE DURING RUBBING TEST. RUBBING BAR IN CONTACT

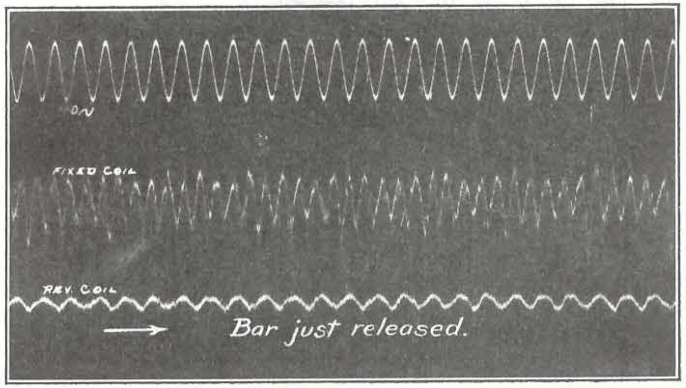


FIG. 84 OSCILLOGRAPH RECORD MADE DURING RUBBING TEST. RUBBING BAR JUST RELEASED

quencies after light calking of the bucket and Test 3 after heavy calking at the same point. It will be noted that between Test 1 and Test 2 a considerable change in the 2-node frequency occurred, much smaller differences occurring with the other types of vibration. Fig. 88 gives the critical speeds for 8, 10, and 12 nodes measured by running Tests 1, 2, and 3 of the wheel under the same conditions, respectively, as shown in Fig. 87. It will be



noted that the calking caused very large changes in these critical speeds.

216 Apparently, in spite of the attempts to insure tightness of the dovetail under running conditions, this was not obtained without the calking operation. The usual type of dovetail, in which the fit is obtained by forcing the dovetail in the groove, is much more consistent in this respect, the first type being unreliable in use because there is no assurance that a tight fit can be maintained.

217 Tests on the standard-type dovetail, which is forced into the groove, revealed the fact that, while a very considerable change in the standing frequency was brought about by removing the buckets and replacing them loosely, the running tests showed very little change in the critical speeds in the two cases. In the

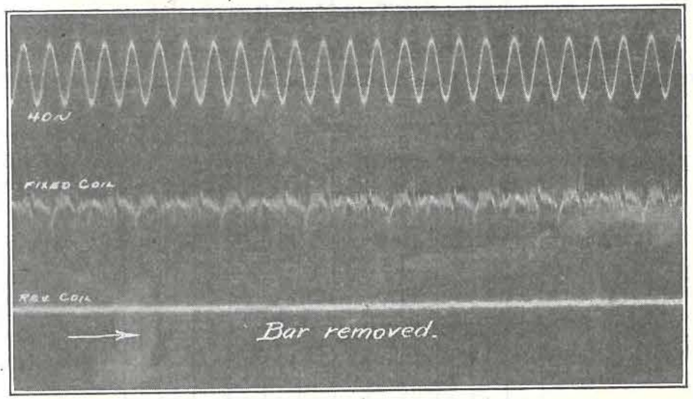


FIG. 85 OSCILLOGRAPH RECORD MADE DURING RUBBING TEST. A MOMENT AFTER RELEASE OF THE RUBBING BAR

standing condition the stiffness due to the dovetail fit had been changed but in both cases during running conditions the centrifugal forces insured a tight fit regardless of whether or not the buckets had been assembled loosely in the dovetail.

218 A somewhat similar case occurred with the type of dovetail in which the bucket straddles the rim. In this type of fastening calking is resorted to along the wheel rim for the purpose of insuring tightness. Table 3 gives the effect on the frequency and critical speed of changes in the tightness of the dovetail. Columns 1, 2 and 3 give the standing frequencies, while columns *A*, *B* and *C* give the critical speeds recorded during running tests. Columns 1 and *A* represent the original assembly after the buckets had been assembled with relatively heavy calking. Columns 2 and *B* give the results after the buckets had been removed and replaced without recalking. Columns 3 and *C* give the same kind of information after the wheel had been recalked.

219 It will be noted that both the frequencies and the critical speeds were lowered by removing the buckets from the wheel and reassembling, but both the standing frequencies and the critical speeds were raised to practically the normal conditions upon recalking.

TABLE 3 EFFECT OF BUCKET TIGHTNESS ON FREQUENCY AND CRITICAL SPEED

| | Standing Frequencies | | | Running Critical Speeds | | |
|---|---|---|---|---|---|---|
| | Original assembly | Loose buckets | Buckets recalked | Original assembly | Loose buckets | Buckets recalked |
| Nodes | 1 | 2 | 3 | *A* | *B* | *C* |
| 4 | 48 | 45.2 | 47.4 | 34.5 | 33.7 | 34.4 |
| 6 | 56 | 52.4 | 55.2 | 21.6 | 20.8 | 21.6 |
| 8 | 68 | 63.2 | 67.0 | 18.4 | 17.9 | 18.3 |
| 10 | 78 | 72.8 | 77 | 16.6 | 16.0 | 16.6 |
| 12 | 87 | 80.8 | 86.8 | 15 | 14.3 | 15.2 |

## Condition for Two-Node Resonance

220 Little has been said so far with regard to the 2-node resonant speed. One reason is that it has rarely been found to have

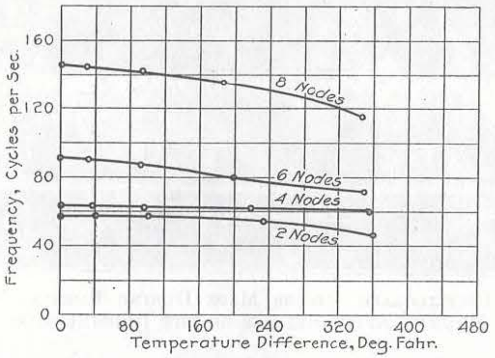


FIG. 86 EFFECT OF DIFFERENTIAL TEMPERATURE ON FREQUENCY (HOT RIM AND COLD HUB)

caused trouble. A second reason is that a 2-node wave in a rotating turbine wheel has never been known to be stationary in space. Therefore the same identical causes that produce standing waves with a larger number of nodes cannot be expected to act in the case of the 2-node wave trains.

221 Two-node vibrations differ from all other radial nodal vibrations in that the forces involved form a couple that is opposed by a corresponding couple in the shaft, whereas in the case of 4, 6, 8, etc. nodes all forces are balanced within the disk. Thus an oscillating motion of one end of the shaft would be expected to cause a 2-node vibration if the frequency of the applied force were equal to the resonant frequency of the disk. This is found to be actually the case when a small unbalanced motor is bolted to the



end of the shaft with the armature at right angles to the wheel shaft.

222 The 2-node frequency is affected by the centrifugal force in a manner similar to that of other types of vibration. Thus the frequency when running, according to Equation [5] is

$$f_r = \sqrt{f_s^2 + BN_s^2}$$

223 If it were possible for the 2-node wave to be stationary in space, this would occur when

$$f_r = \tfrac{1}{2}nN_s$$

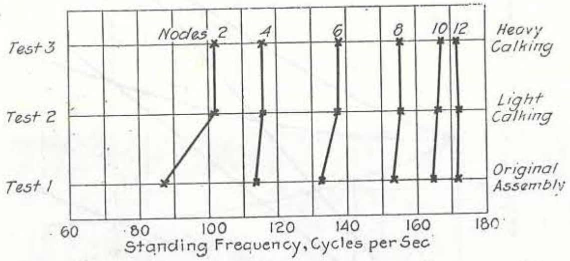


FIG. 87 EFFECT OF DOVETAIL TIGHTNESS UPON STANDING FREQUENCY

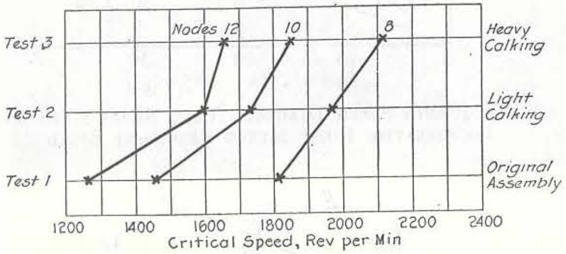


FIG. 88 EFFECT OF DOVETAIL TIGHTNESS UPON CRITICAL SPEED

224 Substituting this in the above formula the critical speed would be

$$N_s = \sqrt{\frac{f_s^2}{(\tfrac{1}{2}n)^2 - B}} \quad \dots\dots\dots [15]$$

225 When $B$ is greater than unity, and $n$ equals 2, it can be seen that $N_s$, the critical speed, would be imaginary. Since $B$ is always found to be above unity except under very unusual conditions, it is therefore believed that 2-node wave trains stationary in space do not occur.

226 However, in the wheel-testing machine 2-node records have been made at what is called a 2-node minor resonant speed. Referring to Fig. 89, which is a frequency-speed diagram for two nodes, it will be seen that there is a diagonal line drawn from the zero corner and passing through all points where the frequency

is equal to the speed of rotation. This intersects at point $x$ the backward-wave frequency line which, it will be recalled, is also the resonant frequency line for an applied impulse, fixed in space, such as the exciting magnet would give. It is at this speed that

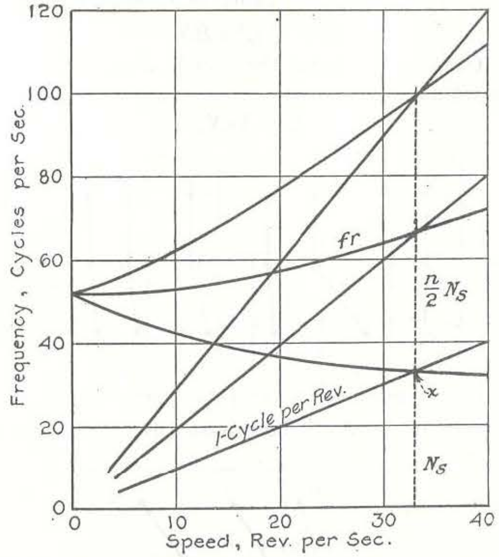


FIG. 89 FREQUENCY-SPEED DIAGRAM FOR 2 NODES. THE POINT $x$ LOCATES THE FIRST MINOR RESONANT SPEED

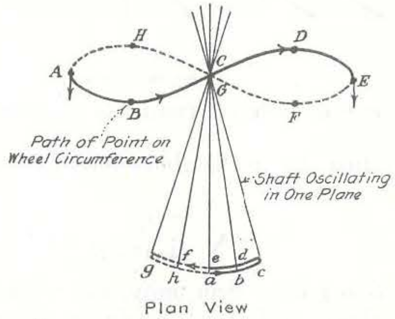


FIG. 90 DIAGRAM SHOWING RELATION BETWEEN DYNAMIC UNBALANCE AND 2-NODE VIBRATION

the periodic effects of shaft unbalance may excite a 2-node wheel vibration.

227 It will also be seen that a turbine wheel is in a resonant condition when the natural 2-node frequency for a particular speed, as recorded by a revolving coil, is just twice the revolutions per second of the wheel itself, so that it results in a wave being



set up with a wave velocity equal to twice that of the wheel itself. It is possible that a simple vibration shape, (i.e., fixed nodes in the disk) could be maintained if the wheel were run in a perfect vacuum, but under other conditions the high velocity component wave would receive a great amount of damping, probably greatly reducing the amplitude, while not so affecting the backward-traveling wave. The high velocity wave alone would travel with three times the wheel velocity while the backward wave would have a velocity in space equal to that of the wheel but in an opposite direction.

228. The path of a bucket during shaft vibration maintained by dynamic unbalance, which might excite a 2-node resonant vibration, is shown in Fig. 90. Dynamic unbalance tends to make the shaft oscillate mostly in one plane, the horizontal stiffness of

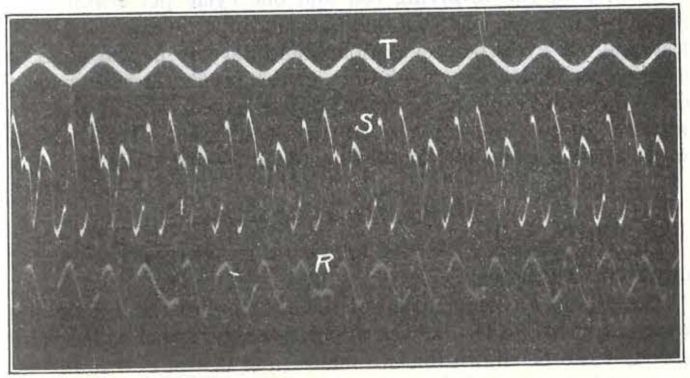


FIG. 91 OSCILLOGRAPH RECORD SHOWING 2-NODE FORWARD WAVE AT FIRST MINOR RESONANT SPEED

the bearing supports usually being less than the vertical stiffness. In the neutral position it exerts no force on the bucket. When it swings from $a$ to $c$ as indicated, it pulls the bucket on the path $ABC$. Swinging back along $c$, $d$, $e$, $f$, $g$, the shaft causes the bucket to travel along $C$, $D$, $E$, $F$, $G$, and on the return swing $g$, $h$, $a$, the bucket travels along $G$, $H$, $A$. It is evident therefore that during one oscillation, i.e. one revolution, of the shaft the bucket goes through two complete cycles. Thus the transverse frequency of the bucket due to unbalance is twice that of the revolutions per second of the wheel itself, which checks with the revolving coil frequency for a 2-node resonant condition discussed in the previous paragraph.

## RECORD OF TWO-NODE FIRST MINOR RESONANT SPEED

229 Fig. 91 is a standard oscillogram taken on a wheel in which excessive dynamic unbalance was intentionally applied to obtain

a 2-node resonant speed. The curve $T$ is a 40-cycle timing wave. The curve $S$ was registered by the coil stationary in space. The curve $R$ is that registered by a revolving coil.

230 During each revolution the revolving coil passed through the influence of the stationary coil causing the disturbances noticed on the revolving coil record. Comparison of the spacing between these disturbances with the timing wave indicates the speed of rotation. It will be noted that while the revolving coil registers exactly two cycles per revolution the stationary coil registers three cycles per revolution. This indicates a 2-node forward wave which was developed in this special case.

231 From the ratio of these frequencies per revolution information is obtained of what is going on at the time the oscillogram is taken. A backward wave would have registered two cycles per revolution of the revolving coil and one cycle per revolution on the stationary coil. In this case caution must be exercised to note that the one cycle per revolution would develop at the same time as the two cycles per revolution on the revolving coil, since the ordinary autograph of a non-vibrating wheel might appear to register one per revolution at any speed, whereas a true backward wave would appear only at the instant of the vibration developing.

## Minor Resonant Speeds

232 It is possible to excite and record minor resonant speeds for other nodal systems.

233 From a consideration of the equation of the 2-node minor resonant speed as well as the major resonant speed called critical speed, it can be shown for two nodes (and it also is true for other nodes) that some of the resonant conditions do not occur. The critical speed, if one exists, occurs at a speed given by Equation [15]

$$N_s = \frac{f_s}{\sqrt{(\frac{1}{2}n)^2 - B}}$$

For 2 nodes $(\frac{1}{2}n)^2 = 1$ and the radical becomes $\sqrt{1-B}$. Now in the case of turbine wheels if $B > 1$, $N_s$ is imaginary, namely, the 2-node backward-wave frequency line never intersects the zero line. There is, then, no critical speed for 2-node vibration. The danger from two nodes, if present, would be expected to occur at the minor resonant speed. The equations for first minor resonant speeds for any system are found as follows:

234 Referring to Fig. 92 it will be seen that the running frequency for a minor resonant speed is

$$f_r = N_s + \tfrac{1}{2}nN_s$$

Substituting in Equation [5]

$$N_s + \tfrac{1}{2}nN_s = \sqrt{f_s^2 + BN_s^2}$$



Solving for $N_s$

$$N_s = \frac{f_s}{\sqrt{(1+\frac{1}{2}n)^2 - B}} \quad \ldots\ldots\ldots \quad [16]$$

The equations for determining the first *sub-minor* resonant speed, indicated as the speed where the backward wave frequency line intersects the 1-cycle per revolution line below the zero frequency line is derived as follows:

235 Referring to Fig. 92,

$$\tfrac{1}{2}nN_s - N_s = \sqrt{f_s^2 + BN_s^2}$$

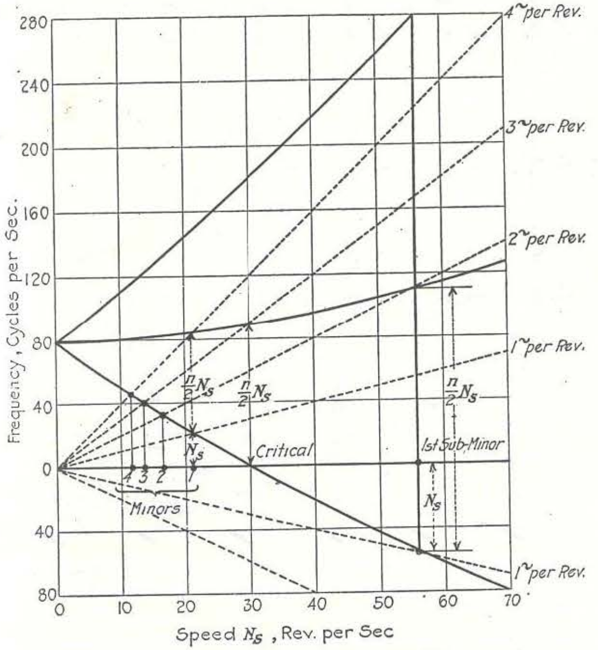


Fig. 92 Frequency-Speed Diagram Showing Minor, Critical, and Sub-Minor Resonant Speeds

The first sub-minor resonant speed then equals

$$N_s = \frac{f_s}{\sqrt{(\frac{1}{2}n - 1)^2 - B}} \quad \ldots\ldots\ldots \quad [17]$$

The second and third minor and sub-minor resonant speeds can be determined in a similar manner, the point of intersection in these cases being the 2-cycle and 3-cycle per revolution lines, respectively.

236 From the formulas in Table 4 the various resonant speeds can be calculated when the standing frequency $f_s$ and the speed coefficient $B$ are known for the particular number of nodes $n$.

TABLE 4 FORMULAS FOR RESONANT SPEEDS

| | | Nodes 2 | 4 | 6 | 8 | 10 |
|---|---|---|---|---|---|---|
| 3d Minor Resonance............ | $N_{S_3} = \dfrac{f_s}{\sqrt{\left(3+\dfrac{n}{2}\right)^2 - B}}$ | $\dfrac{f_s}{\sqrt{16-B}}$ | $\dfrac{f_s}{\sqrt{25-B}}$ | $\dfrac{f_s}{\sqrt{36-B}}$ | $\dfrac{f_s}{\sqrt{49-B}}$ | $\dfrac{f_s}{\sqrt{64-B}}$ |
| 2nd Minor Resonance............ | $N_{S_2} = \dfrac{f_s}{\sqrt{\left(2+\dfrac{n}{2}\right)^2 - B}}$ | $\dfrac{f_s}{\sqrt{9-B}}$ | $\dfrac{f_s}{\sqrt{16-B}}$ | $\dfrac{f_s}{\sqrt{25-B}}$ | $\dfrac{f_s}{\sqrt{36-B}}$ | $\dfrac{f_s}{\sqrt{49-B}}$ |
| 1st Minor Resonance............ | $N_{S_1} = \dfrac{f_s}{\sqrt{\left(1+\dfrac{n}{2}\right)^2 - B}}$ | $\dfrac{f_s}{\sqrt{4-B}}$ | $\dfrac{f_s}{\sqrt{9-B}}$ | $\dfrac{f_s}{\sqrt{16-B}}$ | $\dfrac{f_s}{\sqrt{25-B}}$ | $\dfrac{f_s}{\sqrt{36-B}}$ |
| Critical Speed ............ | $N_S = \dfrac{f_s}{\sqrt{\left(\dfrac{n}{2}\right)^2 - B}}$ | | $\dfrac{f_s}{\sqrt{4-B}}$ | $\dfrac{f_s}{\sqrt{9-B}}$ | $\dfrac{f_s}{\sqrt{16-B}}$ | $\dfrac{f_s}{\sqrt{25-B}}$ |
| 1st Sub-Minor Resonance........ | $N_{SA} = \dfrac{f_s}{\sqrt{\left(\dfrac{n}{2}-1\right)^2 - B}}$ | | | $\dfrac{f_s}{\sqrt{4-B}}$ | $\dfrac{f_s}{\sqrt{9-B}}$ | $\dfrac{f_s}{\sqrt{16-B}}$ |
| 2nd Sub-Minor Resonance........ | $N_{SB} = \dfrac{f_s}{\sqrt{\left(\dfrac{n}{2}-2\right)^2 - B}}$ | | | | $\dfrac{f_s}{\sqrt{4-B}}$ | $\dfrac{f_s}{\sqrt{9-B}}$ |
| 3d Sub-Minor Resonance........ | $N_{SC} = \dfrac{f_s}{\sqrt{\left(\dfrac{n}{2}-3\right)^2 - B}}$ | | | | | $\dfrac{f_s}{\sqrt{4-B}}$ |



237 From the similarity of these formulas it will be noted that assuming the standing frequencies and speed coefficients to be constant, a speed which is critical for one nodal type is resonant as a first minor for the next smaller number of nodes and as a first sub-minor for the next larger number of nodes. Thus in order to appreciate this similarity, suppose that the standing frequencies and speed coefficients are the same. It will be seen that the 6-node critical speed is also resonant for a 4-node first minor and a 2-node second minor on the one hand, and also for an 8-node first sub-minor and a 10-node second sub-minor on the other hand.

238 From the fact that the speed coefficient $B$ is always greater than unity it is easily shown that there are no 2-node critical or

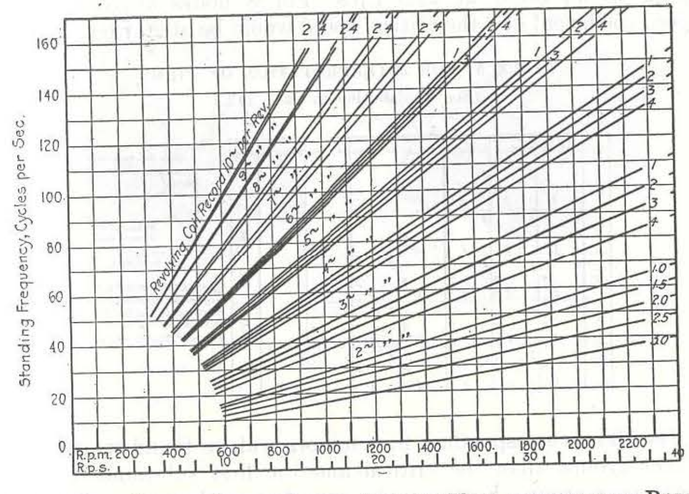


Fig. 93 The Critical-Speed Chart. This is Used also for the Rapid Determination of Minor Resonant Speeds

sub-minor resonances, nor any 4-node sub-minor resonances, etc., as indicated by the blank spaces in the table.

239 Fig. 93 is used for the rapid solution of all these formulas. The vertical scale represents the standing frequency $f_s$ in cycles per second. The horizontal scale marked critical speed both in r.p.m. and r.p.s. is also used to determine the other resonant speeds. The diagonal lines, which are grouped for the different nodal systems, are also marked for various values of the speed coefficient $B$. The lowest group, marked two cycles per revolution, is made first for the 4-node critical speed, the two cycles per revolution meaning that two complete cycles would be recorded on the revolving coil in the wheel-testing machine. The three cycles per revolution group is for the 6-node critical speed, the four cycles per revolution for eight nodes, and so on. However, as

already referred to, since the 2-node first minor resonance is represented by the same formula as the 4-node critical speed, the lowest group is also used for determining the 2-node minor resonant speed. Similarly, the three cycles per revolution group is used for the 4-node first minor and the four cycles per revolution group for the 6-node first minor, and so on. The group below any particular group of diagonal lines is used to determine the sub-minor critical speed for the same particular number of nodes.

240 For example, assuming a wheel with a 4-node standing frequency equal to 50 cycles per second, and with the speed coefficient 2, the major resonant or critical speed will then be at 35.5 r.p.s. For 6 nodes with standing frequency at 57.0 the critical speed would occur at 21.5 r.p.s. For 8 nodes at 75 and the speed coefficient of 2 the critical speed would be at 20 r.p.s.

TABLE 5 FOR INTERPRETATION OF FILMS

(See Explanation in Par. 242.)

| Nodes | 3rd Minor | 2nd Minor | 1st Minor | Critical | 1st Sub-Min | 2nd Sub-Min | 3rd Sub-Min | Nodes |
|---|---|---|---|---|---|---|---|---|
| 2 | 4, 5, 3 | 3, 4, 2 | 2, 3, 1 | | | | | 2 |
| 4 | 5, 7, 3 | 4, 6, 2 | 3, 5, 1 | 2, 4, 0 | | | | 4 |
| 6 | 6, 9, 3 | 5, 8, 2 | 4, 7, 1 | 3, 6, 0 | 2, 5, 1 | | | 6 |
| 8 | 7, 11, 3 | 6, 10, 2 | 5, 9, 1 | 4, 8, 0 | 3, 7, 1 | 2, 6, 2 | | 8 |
| 10 | 8, 13, 3 | 7, 12, 2 | 6, 11, 1 | 5, 10, 0 | 4, 9, 1 | 3, 8, 2 | 2, 7, 3 | 10 |

KEY- Numbers are cycles per revolution.
Revolving Coil Record (upper left); Stationary Coil (Forward Wave) (right); Stationary Coil (Backward Wave) (lower)

Example: A combination of 3 cycles per revolution on the Revolving coil and 5 on the Stationary coil represents a 4 Node 1st Minor, forward wave.

241 The corresponding first minors will be found in the next higher groups than the critical and the first sub-minors in the next lower group. For example, consider another case where the 6-node standing frequency is 50 cycles per second with a speed coefficient of 2. The 6-node first minor resonant speed will then be 13.4 r.p.s., the 6-node critical speed would be 18.9 r.p.s., and the 6-node first sub-minor resonant speed would be 35.5 r.p.s.

## Interpretation of Films

242 Table 5 contains the information necessary to determine quickly the particular type of possible resonant speed occurring at the time of making the oscillograms during test in the wheel-testing machine. The number in the upper left-hand corner represents the number of cycles per revolution recorded on the revolving coil. In the lower right-hand corner are two numbers; the upper is that recorded on the stationary coil if a forward wave in the wheel is occurring, while the lower number is that on the same coil registered by a backward-traveling wave in the wheel. The backward wave in all minors always travels in a direction in space



opposite to that of the wheel itself while in all sub-minors it travels in space at a slower speed but in the same direction as the wheel. As an additional precaution, the standing frequency is computed from the running frequency and an estimated speed coefficient. Agreement with the measured standing frequency then forms a check on the particular type of resonant speed recorded and removes any ambiguity of the table.

243 Although little trouble is attributed to minor resonant speeds in general, the subject has been gone into here for the purpose of showing the reasoning used in locating a critical speed far above running speed when it is impossible safely to run the wheel so far above its operating speed. Minor resonant speeds are thus used to get the information necessary for the accurate

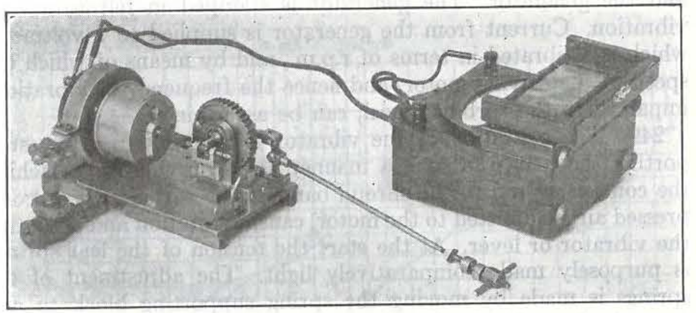

Fig. 94 Portable Compressed-Air-Driven Vibrator for Determining Standing Frequencies of Turbine Wheels

(Note the worm-gear speed reduction for electric tachometer.)

determination of critical speeds when such speeds cannot safely be attained.

## Tests of Wheels Already Installed and Operating

244 At an early stage of the investigation and as soon as sufficient evidence had been established as to the principal cause for turbine disk troubles, a program was worked out in order immediately to determine the condition of turbines already in operation. This work was taken up energetically, the machines were opened, and the natural periods of the wheels obtained.

245 For convenience in vibrating the wheels of turbines already installed, portable vibrating machines were used. The first device was operated electrically and was similar to that shown in Fig. 46.

246 A much more convenient device was later devised to be operated by compressed air. Fig. 94 shows this complete. The long connecting rod is provided with a clamping screw for temporary connection to the wheel shroud band. The opposite end of the rod is provided with a clevis, the lugs of which straddle a vertical lever. The lower end of the lever extends into a small

block which is restrained by adjustable laminated springs. The upper end of this lever is connected by a crank to a ball-bearing eccentric shaft, which carries a ratchet-type wheel. Under some conditions the block is vibrated by the lever and at other times the block remains stationary, or practically so and forms a pivotal support for the lever as will be seen later.

247 The ratchet wheel serves as an air turbine and is driven by compressed air from a nozzle. Changes of speed are brought about by regulation of the air valve.

248 The driving shaft is carried in ball bearings. One end of the shaft has a worm, which meshes with a worm wheel, driving an electric generator forming part of an electric tachometer. A flexible rubber coupling is provided between the worm-wheel shaft and the generator. The generator is clamped in felt to absorb vibration. Current from the generator is supplied to a voltmeter which is calibrated in terms of r.p.m., and by means of which the speed of the driving motor, and hence the frequency of vibrations imparted to the turbine wheel, can be ascertained.

249 The operation of the vibrator is as follows: After supporting the device in such a manner as to provide for attaching the connecting rod to the shroud band of the turbine wheel, compressed air is admitted to the motor, causing rotation and actuating the vibrator or lever. At the start the tension of the leaf springs is purposely made comparatively light. The adjustment of the springs is made by moving the spring supporting block to and from the lever block. The lever vibrates about the pivotal connection to the connecting rod as an axis. Until a speed corresponding to a natural resonance of the turbine wheel is reached the connecting-rod pivot of the lever continues to act as a fulcrum. However, when a resonant speed is reached, an amplitude is built up in the bucket wheel allowing this pivotal point to vibrate back and forth. At the same time, the lower end of the lever quickly ceases to vibrate, and practically all the energy is transferred through the connecting rod.

250 The operator either by sense of touch or observation determines the number of nodal points practically at rest in the circumference of the wheel and this, together with the frequency of the applied force, is recorded.

251 It is often necessary, in order to build up a sufficiently large amplitude to obtain trustworthy observations, to increase the applied air pressure after having somewhat increased the spring tension. As the mass of the turbine wheel to be vibrated is great, and the energy of the vibrator is small, it is difficult to change the speed from one natural frequency of the wheel to another by merely changing the air supply to the motor, and therefore a method commonly employed is to release the spring tension on the block until such time as the turbine wheel comes to rest or practically so. The motor is then operated at a gradually increasing



speed, and at the same time the tension is increased somewhat on the spring; the increase of speed is arrested automatically when the next higher natural frequency is reached. The former procedure is then gone through again and this process is repeated until all of the required frequencies have been determined.

252 From the information thus obtained curves are made for each machine showing at what speed it might be possible for each individual wheel to give trouble. Over 1600 wheels were vibrated in this manner and reference to Table 1 will give an idea of the scope of this work.

## Safe Design

253 To insure safe operation of turbine wheels, it is essential that all the wheels in a turbine rotor without exception shall be

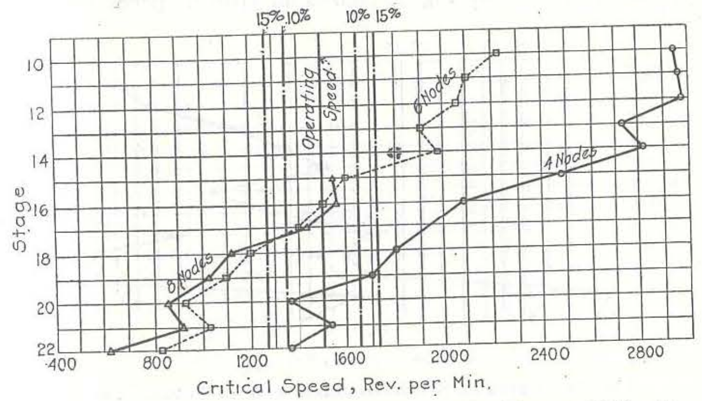


Fig. 95 Critical-Speed Diagram for an Old-Design 22-Stage Turbine

(Stages 15, 16, 17, 19, 20, 21, 22 all have critical speeds separated from running speed by insufficient margins.)

free from critical speeds within dangerous proximity to the operating speed of the rotor.

254 The chart of Fig. 93 is used for the rapid determination of the critical speed when the standing frequency for each particular nodal system is known together with a satisfactory speed coefficient $B$. After the various critical speeds have been determined in this manner, they are plotted on curves as shown in Fig. 95.

255 This diagram is for a 22-stage steam turbine. The horizontal scale represents revolutions per minute of the turbine rotor. The vertical scale represents wheels in the different stages of the rotor. In the diagram, the 13 largest wheels are the only ones indicated, the critical speeds corresponding to 4, 6 and 8 nodes being shown for each. These critical speeds are indicated in the

diagram by circles, squares and triangles respectively. It will be observed that some critical speeds occur close to the operating speed. Thus the wheels of stages 15, 16 and 17 all have critical speeds corresponding both to six and eight nodes occurring close to the operating speed, whereas stages 19, 20, 21, and 22 all have critical speeds corresponding to 4 nodes close to the operating speed. This diagram serves to indicate those stages whose critical speeds occur so close to running speed that, on account of the known variations of running speed, breadth of resonance, etc., the danger of stationary wave development cannot definitely be said not to exist. Figs. 96 and 97 are for machines especially designed to avoid wave development. It will be observed that there are no critical speeds in either case within 15 per cent of the operating speed. In Fig. 96 stages 16 and 17 have critical speeds below the operating speed, but Fig. 97 shows all critical speeds above the

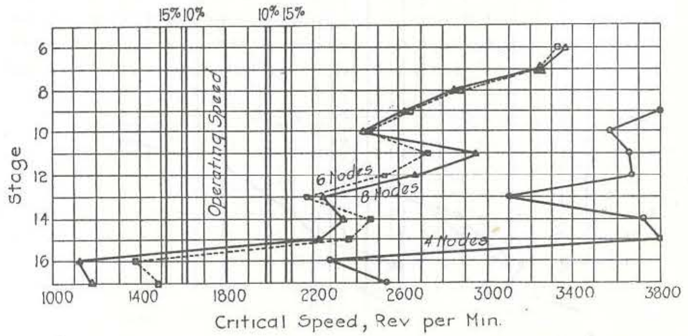


FIG. 96 CRITICAL-SPEED DIAGRAM FOR 17-STAGE TURBINE

(This machine is designed to avoid development of vibration. All critical speeds fall outside the prescribed margins.)

operating speed. The latter case is of course preferable where possible of attainment. In the case of stages 16 and 17 of Fig. 96 it was not possible to design wheels having 6 or 8 nodes above the operating speed, therefore care is necessary in the design to provide as large an interval as possible between the 4 and 6 node critical speeds.

## SAFE LIMITS

256 In determining the safe limits which should exist between the normal operating speed of the rotor and the speeds at which dangerous wave phenomena develop a number of considerations are involved. In the first place a certain broadness of resonance exists, throughout which vibration may develop. That is to say, wave phenomena do not occur with precise mathematical accuracy at exactly a particular speed, but may occur at speeds slightly above or slightly below the speed corresponding to the condition



of maximum resonance. Special tests have shown that this broadness of resonance amounts to about two per cent above and below the critical speed.

257 Another factor, to be considered in determining safe limits between the operating speed and the calculated critical speed, is the uncertainty in the numerical speed coefficient $B$. This coefficient serves to determine the running frequency of the turbine wheel when the actual standing frequency is known. During the process of design, the errors which may occur in applying this coefficient to wheels similar to those that have been tested must also be allowed for. Another consideration is the possible variation of the line frequency on which the turbine is to be operated. Temperature variations must also be covered.

258 For these effects 10 per cent above and below the operating speed is recommended for six and eight nodes, and 15 per cent

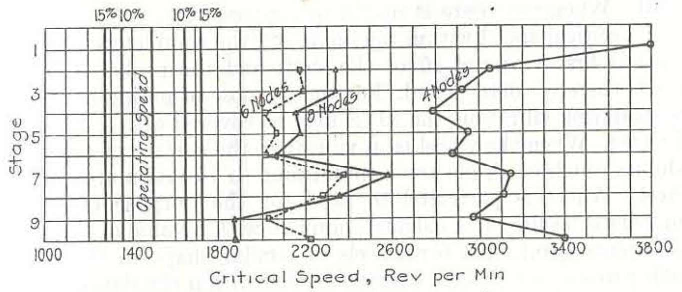


FIG. 97 CRITICAL-SPEED DIAGRAM FOR 10-STAGE TURBINE

(This machine is designed to avoid development of vibration. All critical speeds are above the prescribed margins.)

for the 4-node condition. These limits have been rigidly adhered to for some time, with apparently sufficient justification. Furthermore wheels are not passed for use that have 10-node, 12-node, etc., critical speeds near the operating speed. Such wheels are corrected by tuning. The same precautions are also taken for possible minor resonant speeds.

## TUNING

259 Tuning is resorted to where necessary for the purpose of altering the natural frequencies and is carried out as follows:

260 In proportioning or in *tuning* a wheel to obtain the desired margins between the critical speeds of the wheel and the normal operating speed, the relations between the stiffness and the mass of the parts of the wheel must be made such as to give the desired natural frequencies of vibration in the wheel. In general it may be stated that in a vibrating system, such as a turbine disk, the frequencies of vibration increase with the stiffness of the wheel

but decrease with the increase in mass of the wheel. This may be inferred from Equation [1] for a particle

$$f_s = \frac{1}{2\pi}\sqrt{\frac{R_s}{m}}$$

where $R_s$ equals stiffness and $m$ equals the mass of the vibrating particle. In designing the wheel this relation is borne in mind, since nodal frequencies of different numbers of nodes correspond to bending actions or flexures extending into the body of the wheel toward its center to greater or less degrees. Depending on the number of nodes, changes in the wheel between stiffness and mass may be expected to affect to different degrees, frequencies corresponding to different numbers of nodes. In order, therefore, for the wheel to be free from dangerous critical speeds, a proper coördination between the stiffness and mass of different portions of the wheel should exist.

261 Whenever there is reason to suppose that any wheel may have a critical speed within the limits set, the completely bucketed wheel is first vibrated off of the shaft and the probable critical speeds thereby determined. For the purpose of noting nodal segments during vibration, the wheel may be covered with a thin sheet of water. When the wheel is in vibration, the surface of the water exhibits rough or ripple areas conforming to vibration areas of the wheel. Water is preferred to sand for the purpose of getting immediate results. If a calculation made with a value of the speed coefficient determined for wheels of similar shape in the wheel-testing machine indicates a critical speed within the danger limits, the wheel is then placed upon its own shaft and vibrated again to determine more exactly the critical speeds as altered by the wheel fit on the shaft. Should the previous estimates be verified, then tuning is resorted to.

262 The tuning or reproportioning of the wheel may be done in a variety of ways depending upon the number of nodes and the frequency which it is desired to change. The previous experience and recorded data are found exceedingly helpful in facilitating this work.

263 Suppose, for example, the case of a 22nd-stage wheel with buckets 28 in. long and a disk diameter of 7½ ft., which upon being vibrated is found to have the 4-node critical speed approximately 8 per cent above the operating speed, and the critical speeds corresponding to six and eight nodes well outside the lower 10 per cent limit for these critical speeds. In this case it is necessary to raise the 4-node frequency to 15 per cent or more above the operating speed. This is done by removing the necessary amount of weight from the buckets. Temporarily attaching known weights to the shroud band of the wheel approximately indicates the effect of bucket weight on the frequencies of the wheel. From these data the necessary amount of weight to be removed from the



buckets is determined. Of course it is necessary to remove the buckets from the wheel in order to machine off metal which is taken from the backs of the buckets. Removal of weight from the outer or convex portion of the buckets is illustrated in Fig. 98 as indicated by the dotted lines. This remedy however is very rarely resorted to, as attempts are always made to insure a 4-node critical speed occurring well above the 15 per cent limit.

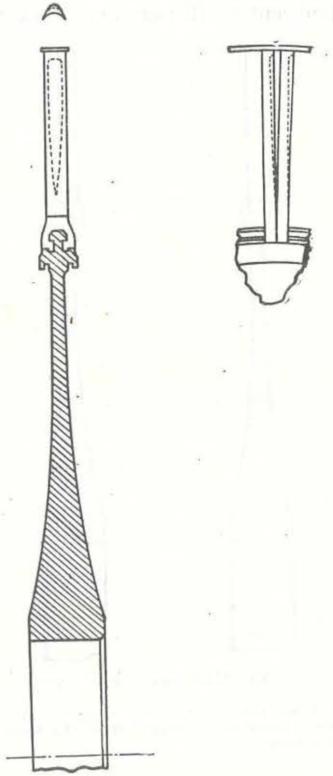

Fig. 98 Methods of Tuning

(Frequency may be raised by removing weight from the buckets as shown.)

264 Removing weight from the buckets in this manner results in a reduction of the mass without any appreciable effect on the stiffness, resulting in raising the frequency of the complete wheel.

265 As another illustration, consider a wheel with buckets 11½ in. long and with a disk diameter of 6 ft., but let it be supposed that the vibration tests show the existence of the 6-node critical speed about 3 per cent above the operating speed and the 8-node critical speed about 4 per cent below the operating speed, the

4-node critical speed occurring about 25 per cent above the operating speed. In this case, the 6-node and 8-node critical speeds may be lowered by removal of material from the wheel at a suitable point, but this must be done in such a way that the 4-node critical speed will not at the same time be lowered so far as to come within the danger limit of 15 per cent. This means that the 6-node critical speed must be brought to a point at least 10 per cent below the operating speed, the 8-node critical speed lowered from 4 per cent to 10 per cent below the running speed,

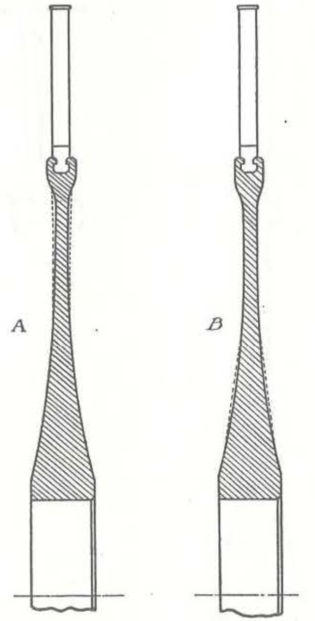


Fig. 99 Methods of Tuning

(Removal of weight from the outer web, as shown at *A*, affects mainly the 6- and 8-node frequencies. Removal of weight from the inner web, as shown at *B*, affects mainly the 4-node frequency.)

and all of these without lowering the 4-node critical speed by more than 10 per cent. In a case like this, great care must be taken to cut the wheel in the location which will most affect the critical speed which it is desired to change. Thus in the one under consideration, alterations in the web of the wheel next to the rim circumference have the greatest effect on the 8-node and 6-node frequency, while in the zone nearest the center of the wheel the removal of the material from the web has its greatest effect on the 4-node frequency.

266 For adjusting the 6- and 8-node frequency the wheel is then placed on the boring mill and slightly machined in the zone



extending from 6 to 8 in. inwardly from the rim. The wheel is then vibrated and the change in frequency noted. The operations are repeated until the desired frequencies are obtained. It is not passed however until it has received a running test in the wheel-testing machine or has shown by tests on its own shaft that the desired results have been obtained.

267 Fig. 99 at *A* is a profile of the turbine wheel. Dotted lines indicate where metal has been removed from the outside portion of the web of the wheel. The removal of metal from this region affects mostly the frequencies corresponding to six or eight nodes but has little effect upon the frequency corresponding to four nodes. Fig. 99 at *B* shows by dotted lines the location having most effect on the 4-node frequency.

## Summary of Procedure for the Production of a Turbine Wheel

268 The principles and theory of bucket-wheel vibration and the methods of testing have been explained and described. In conclusion it remains to summarize the protective measures utilized in the ordinary process of production in order to assure the safety of turbine wheels.

269 The first step is to design the bucket in accordance with the thermodynamic requirements, select the dovetail suited to the bucket and thus fix upon the design of wheel rim.

270 The second step is to select the general dimensions and contour of the wheel with a view to vibration characteristics. For this, reference is made to records of wheels of like bucket design and diameter. There are two complete catalogs showing the complete vibration characteristics of all wheels so far tested. In one catalog the wheels are listed according to dimension and this is used for design purposes. In the other the wheels are catalogued by machines and this is useful in tracing the record of any particular turbine or wheel.

271 The third step is the calculation of the stresses due to centrifugal force and the completion of the details in a satisfactory manner.

272 The fourth step is a review of the resulting design on two bases. The stresses must be satisfactory and the wheel design must suit the space and clearance requirements of the turbine assembly. Either one of these check inspections may serve to condemn the design and require a new start from the beginning.

273 The fifth step is the selection and assignment of a forging. It should be understood that every forging is examined for its strength properties by means of coupons and for its uniformity by a magnetic survey of its entire structure. A complete record of every forging is kept by serial number, and the assignment of a forging is a separate step in the process. Any deviation in the

shop, from drawing, however slight, that does not result in the destruction of the forging, is placed on record by a new drawing.

274 The sixth step is the standing vibration survey of the finished wheel to make sure that it does not deviate more than an allowable amount from the original expectations. If all the resonant speeds, including both the critical and the various minor resonant speeds do not have the required margins from running speed, tuning is resorted to. Generally one or two trials are sufficient to obtain by tuning the required vibration characteristics but occasionally a wheel has to be altered five or six times. This process of course, necessitates a revision of the drawing and a corresponding alteration of all the records involved.

275 In some cases the standing vibration test confirms the predictions from the catalogs of similar wheels for which satisfactory speed coefficients have been determined in the wheel-testing machine. Such wheels may be accepted as having satisfactory margins on the basis of the standing vibration test alone. But in the majority of all cases each wheel receives a complete running test in a wheel-testing machine.

276 The seventh step is the complete running vibration test in the wheel-testing machine. From 30 to 60 oscillograph films are taken showing exactly all phases of the wheel's behavior under running conditions. These films are all examined and those of value are indexed and filed. About 18,000 such records have been taken, and, of this great number, about one-third are preserved and available for quick reference at the present time.

277 The final result is a wheel which is either satisfactory and thoroughly protected from the possibility of resonant vibration, or else the wheel is rejected and a new design started through the necessary seven steps toward a final acceptance.

278 It will be readily appreciated that this thoroughness of examination and verification of predicted properties is at the basis of the remarkable freedom from vibrational troubles exhibited in the behavior of recently built turbines.

## DISCUSSION

S. Timoshenko.[1] The problem of the turbine-disk vibration is of great practical importance. The first analytical investigation on this subject was by Stodola, followed by the work of Lamb and Southwell.[2] These authors had been previously attracted to this subject by the experiments of Stoney[3] on a rotating india-rubber

[1] Westinghouse Research Laboratory, East Pittsburgh, Pa.
[2] Proc. Royal Society, vol. 99, p. 272, and vol. 101, p. 133.
[3] Institute of Technology, Manchester, England.



disk. In the same year Bauman discussed different types of vibration such as

- *a* Umbrella-shaped vibration
- *b* Segmental vibration with nodal diameters stationary in space
- *c* Segmental vibrations with nodal diameters rotating with shaft.

Lamb, in the work previously mentioned, discussed analytically all these types of vibration for the simple case of a disk having constant thickness.

The principal results described in the present paper can be best summarized as follows: The importance of obtaining vibration data on full-sized wheels under actual operating conditions has been fully appreciated by the author.

This method of procedure was essential. The exploring electric coil used in this machine in conjunction with the oscillograph proved to be a helpful device. By its use different types of vibration have been studied and satisfactory explanation given of their causes. The author shows that the type of vibration responsible for practically all serious wheel failures consists of a train of backward-traveling waves whose backward speed in the wheel exactly equals the forward speed of wheel rotation. This again emphasizes the importance of knowing critical speed.

In this respect it is important to observe that in calculating the critical speed, Rayleigh's approximate method[1] and its further development given by W. Ritz[2] is used in the paper. In applying this method the potential and kinetic energies must be calculated on the basis of an assumed form of deflection. In such manner the system under consideration is transformed into a system with only one degree of freedom. The frequency obtained in this manner will be the upper limit to the true value. While this method of calculation is usually accurate enough in the case of vibrating bars, it is not sufficiently accurate for plates such as disks where more complicated conditions at the edge occur. A calculation giving a better approximation then becomes necessary. The expression for the deflection must be taken in such a manner as to satisfy the conditions at the edge so far as the deflection and the slope are concerned. It must contain also a number of parameters whose magnitude must be determined in such a manner as to make the frequency a minimum. By increasing the number of these parameters, and by taking into consideration edge conditions while choosing the deflection, the accuracy in determining the frequency can be increased.

The method of calculating frequencies outlined in Pars. 138 and 139 is analogous to that of calculating critical loads for compressed

[1] Theory of Sound, pars. 88, 89.
[2] *Annalen der Physik,* vol. 28, p. 797.

columns, proposed by Vianello. It is accurate enough for bars and shafts, but satisfactory results cannot be expected for plates. A simple graphical method does not exist for calculating deflections of plates. Some simplification of the solution can be obtained by using the method of Marcus,[1] which is analogous to the Mohr method of graphical determination of the deflection curve for a bar. Instead of a funicular curve in the Mohr method, the deflection of a flexible membrane must be studied.

The effect of inertia forces on the frequency of vibration of a disk wheel mentioned in Pars. 41 to 43 and explained in an elementary way was studied by R. Southwell[2] and an interesting conclusion obtained. If $f_s$ denotes the frequency of a disk due to stiffness furnished by the elastic property of the disk and $f_c$ the frequency due to the stiffness contributed by centrifugal effects, then a lower limit for frequency $f_r$ of the disk at rotation will be obtained from the equation

$$f_r^2 = f_s^2 + f_c^2$$

This result in conjunction with that obtained by Rayleigh's method makes it possible to establish the accuracy of approximate calculations.

In considering the traveling waves in Par. 48 et seq., an analytical expression for these waves, in addition to their diagrammatic representation, would be useful for explaining this phenomenon. Let $R \cos pt \cos n\theta$ represent standing waves of the segmental type with $n$ nodal diameters. Taking the same waves, but of different phase as in the form $R \sin pt \sin n\theta$ and combining these two types, the traveling waves represented by $R \cos (pt \pm n\theta)$ will be obtained. The corresponding angular wave velocities are $\pm p/n$. Adding to this $\omega$, the angular velocity of the wheel, the angular velocities in space for the forward- and backward-traveling waves will be $\omega + p/n$ and $\omega - p/n$.

It thus can be shown that segmental waves, stationary in space, are produced by combining segmental waves with nodal diameters rotating with the shaft.

The experiments on the dissipation of energy by internal friction are interesting. Little is known on this subject[3] and further study can give important results. In experimenting with simple types of vibration, a study can be made of the manner in which internal friction depends on the frequency and on the magnitude of the stresses.

In regard to the method of determining stresses, it is difficult to agree that the calculation of stresses due to vibration in a

[1] *Armierter Beton*, 1919.

[2] Ibid.

[3] See paper by Honda and Konno, *Phil. Mag.*, vol. 42 (1921), p. 115; also *Zeitschr. f. Angew. Math. und Mech.*, vol. 4 (1924), p. 124.



vibrating disk wheel from the deflection shape of the wheel can be made with sufficient accuracy. It is known that approximate methods for calculating the deflection of plates such as the Rayleigh-Ritz method are sufficiently accurate, so far as the magnitude of the deflection is concerned. For determining the stresses the second and third derivatives of the deflection curve are necessary, and the accuracy with which these derivatives can be obtained from an approximate deflection shape is usually insufficient for practical applications. In order to obtain satisfactory results, direct measurements of variation of distances between the points on the surface of the vibrating wheel are necessary.

In studying vibration of a circular plate a discrepancy between theory and experiment was found. It should be observed that in this study the amplitude of vibrations and the manner of clamping the edge are not given. If the amplitude of the vibrations is not small in comparison with the thickness of the plate, then higher frequencies than those calculated by the Kirchhoff formula must be expected. This increase of frequency will be more likely to appear in the cases of thinner plates and of lower types of vibration.

H. F. Moore.[1] The paper is not only a successful attempt to meet a very practical problem in machine design, but an expedition into the almost totally unexplored field of the dynamics of stress and strain. Practically all the common formulas and methods used in studying the mechanics of materials are based on the laws of statics. In considering stresses and strains in high-speed machinery we should consider the effect of waves of stress, with crests, troughs, and maximum and minimum values. The present paper is an interesting and probably an important contribution to that field of knowledge.

The author states that the practical problem presented was to devise such means that axial vibration would not occur. It may be pointed out that the prevention of localized regions of high stress in a wheel is a secondary method of increasing safety by increasing the resistance to fatigue breakdown should vibration occur. Reference has been made to the investigation of the fatigue of metals carried on at the University of Illinois under the auspices of the National Research Council, Engineering Foundation, and various manufacturing companies. Among the conclusions reached in that investigation are two which bear on this point:

1 A rough-machined surface tends to create regions of high stress at the bottom of tool marks. The resistance to fatigue failure of a machine part with a rough-machined surface may be as much as 15 per cent less than that

[1] Research Professor of Engineering Materials, University of Illinois, Urbana, Ill. Mem. A.S.M.E.

of a machine part of the same material and dimensions with a smooth-finished surface.

2 Holes in the surface may double the localized stress at their edges. It is to be noted that heavy localized stress is not of great importance when the action of a steady stress, such as that caused by centrifugal force, is considered, but that heavy localized *repeated* stress may cause a fatigue crack to start and spread in the metal.

Paul Heymans.[1] The object of this discussion is (1) to call attention to existing stress analyses related to certain of the problems of the present paper; and (2) to emphasize and more closely define some points of theoretical importance bearing upon the interpretation of the experimental data obtained by the author.

*Ruptures of the Disks.* Examining the types of failures of the turbine disks presented in the first part of the paper, one is impressed by the fact that, although in certain cases the fractures avoided the holes existing in the disks, the majority of ruptures started and passed through the holes *and through very definite points of the holes.* This brings attention to the more general question of the disturbances caused in different types of stress distributions by the presence of circular or other types of internal discontinuities.

A. Leon[2] has given an analytical solution for the stress distribution around a circular hole in an infinitely extending plate. Calling $\widehat{rr}$ and $\widehat{\theta\theta}$ the radial and tangential stresses, the elastic state is given at any point $r$, $\phi$:

1 If the plate is put under a uniform pull in a direction parallel to the $x$-axis (Fig. 100), $p$ being the mean longitudinal stress, by:

$$\widehat{rr} = \frac{p}{2}\left(1 - \frac{r_0^2}{r^2}\right)\left[2 - 3\frac{r_0^2}{r^2} - 2\left(1 - 3\frac{r_0^2}{r^2}\right)\sin^2\phi\right]$$

$$\widehat{\theta\theta} = \frac{p}{2}\left[\left(1 - 3\frac{r_0^2}{r^2}\right)\frac{r_0^2}{r^2} + 2\left(1 + 3\frac{r_0^4}{r^4}\right)\sin^2\phi\right]$$

2 If the plate is put under uniform pull in all directions, $p$ being the mean stress, by:

$$\widehat{rr} = p\left(1 - \frac{r_0^2}{r^2}\right)$$

$$\widehat{\theta\theta} = p\left(1 + \frac{r_0^2}{r^2}\right)$$

It readily results from the above solution that:

[1] Assistant Professor of Theoretical Physics and Photoelasticity, Massachusetts Institute of Technology, Cambridge, Mass. Assoc-Mem. A. S. M. E.

[2] Alfons Leon, Uber die Störungen, die in elastischen Körpern durch Bohrungen und Bläschen entstehen.—*Oesterreichische Wochenschrift für Oeffentlichen Baudienst,* 1909.



1 In infinitely extending plates, uniformly pulled in one direction, the stress is a maximum for $\phi = 90$ deg. and $r = r_0$, i.e., at the boundary of the hole, *at the extremity of a diameter normal to the direction of pull,* and is equal to three times the mean stress. For $\phi = 0$ deg. and $r = r_0$, the stress is a compression and is equal to the mean stress.

2 In infinitely extending plates, uniformly pulled in all directions, the stress is a maximum for $r = r_0$, i. e., at the boundary of the hole, and is equal to twice the mean stress.

In finite members, such as a bar of rectangular cross-section which is submitted to a uniform longitudinal pull, the maximum stress at the boundary of the hole should be equal to three times the mean stress if the ratio of the diameter of the hole to the width of the plate is small enough. Fig. 101, which represents the photoelastic analysis,[1] shows that the actual stresses approach the theoretical values as the ratio specified above decreases. The solid lines represent the stresses measured, whereas the dotted lines are the analytical results for an infinitely extending plate.

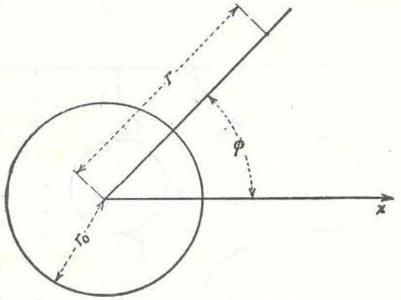

Fig. 100

No investigations, analytical or by the photoelastic method, of the stresses around discontinuities in rotating disks have been made, although they might be highly interesting in the light of the data obtained by the author. However, if the diameter of the hole be small as compared to the radius of the disk, and the hole be far enough from the center and from the rim of the wheel, the case of a plate under uniform unidirectional pull may illustrate qualitatively the disturbance, under centrifugal action, on the stress distribution, which without the presence of the hole would be nearly uniform in the region considered.

It will be noticed that the ruptures run or start at the holes at the two ends of diameters very approximately coinciding with the normal to the direction of the centrifugal force. This accords with the location of the points of maximum stress in the case of the pulled plate to which reference has been made. It might be estimated, although a verification by photoelastic analysis would be more satisfactory, that the hole introduces a maximum stress

[1] E. G. Coker, Trans., Instn. Engrs. & Shipbuilders in Scotland, Dec., 1919. Paul Heymans, Bull. Soc. Belge Ing. & Ind., Aug., 1921.

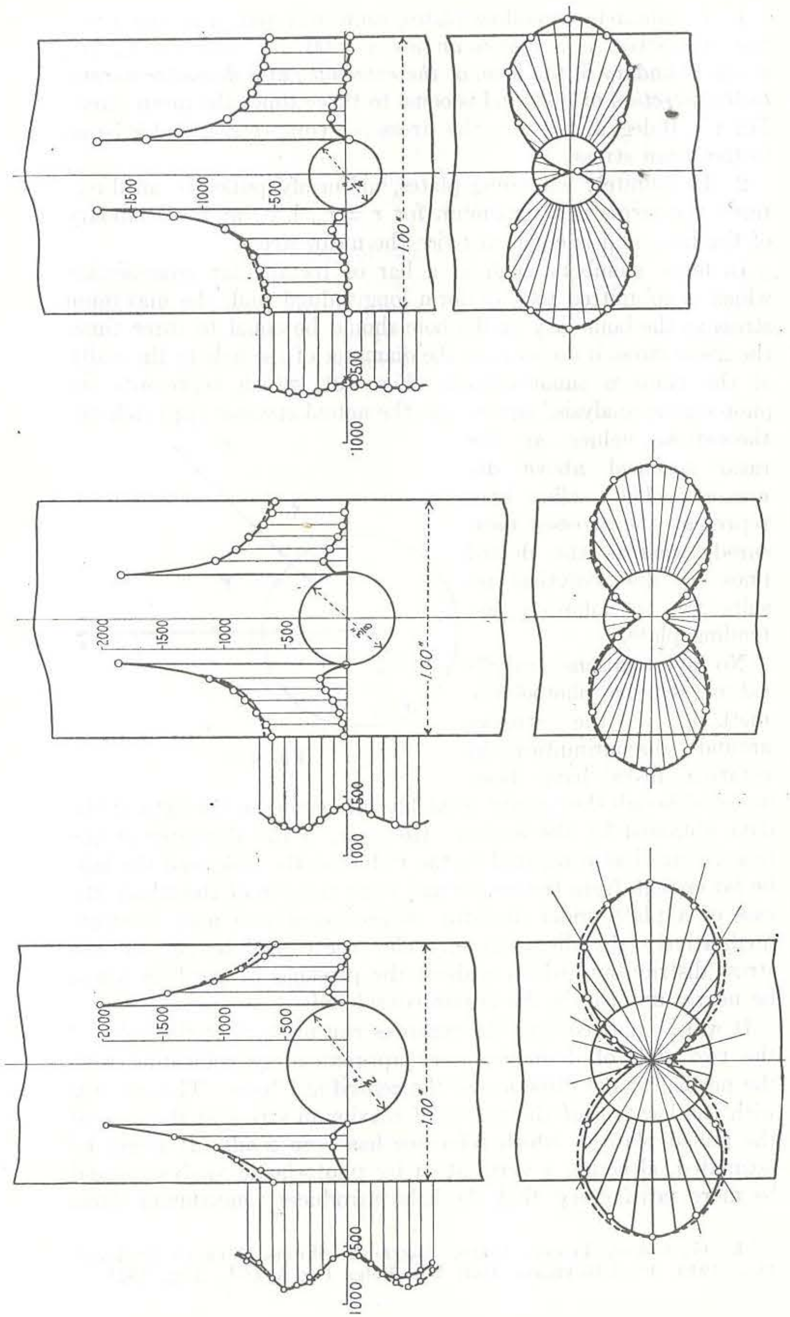

FIG. 101 PHOTOELASTIC ANALYSES OF STRESSES AT BOUNDARIES OF HOLES



equal to three times the stress which would exist without the presence of the hole.

The ruptures of the wheels emphasize the existence of highly localized stresses around the holes, and it may therefore be worth while to say that full information concerning the effect of these discontinuities can be obtained by photoelastic analysis. If a celluloid disk wheel be rotated and a stationary photoelastic image of the stressed wheel at any desired speed between 300 and 3000 r.p.m. be obtained by means of a timed electric spark, such as has been developed for other photoelastic investigations,[1] the stress distributions can be completely determined.

*Ruptures of the Buckets.* The ruptures of the buckets, as described in the paper, call attention to the concentration of stresses

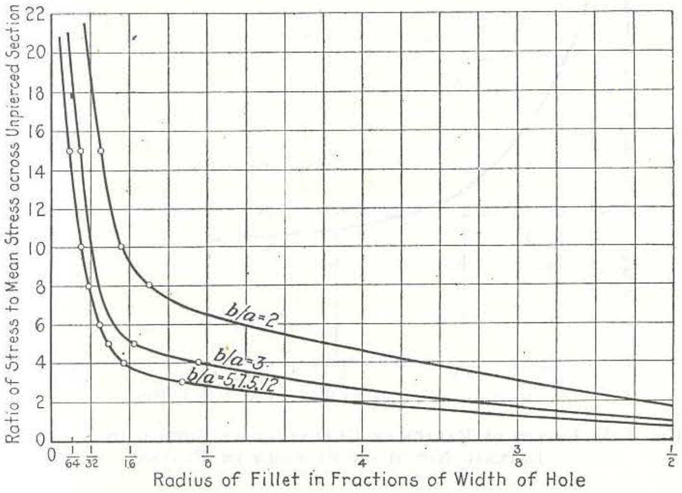


FIG. 102 EFFECT OF RADIUS OF FILLET ON STRESS IN PLATES WITH SQUARE OPENINGS

around external variations of profiles, such as exist in these buckets. It is known that when in the stressed member the radius of curvature at a variation of profile decreases, the intensity of the disturbance on the stress distribution increases; that is, the maximum stress increases. This increase of the maximum stress is well illustrated by the following photoelastic investigation.[2] The stresses around the corners of a square opening in a bar under uniform

[1] P. Heymans and A. L. Kimball, Jr., Stress Distribution in Rotating Gear Pinions as Determined by the Photoelastic Method. *Mechanical Engineering*, March, 1924.

[2] P. E. Pihl and O. D. Colvin, Jr., Investigation by the Photoelastic Method of the Distribution of Stresses around Various Square Openings in a Flat Plate. Thesis for the degree of Master of Science, presented at Massachusetts Institute of Technology, June, 1924.

longitudinal pull were measured with decreasing radii at the fillets in the corners. The increase in maximum stress with curvature at the corners is given by Fig. 102. Fig. 103 shows a similar investigation with a V-shaped lateral notch, with decreasing radii of curvature at the bottom of the V-shaped groove.[1]

The problem in turbine design should be to meet the kinetic conditions of profile of the buckets with the optimum curvatures avoiding stress concentrations.

## Points Bearing on Interpretation of Experimental Data

The object of the second part of this discussion is to emphasize and more closely define some points of theoretical importance

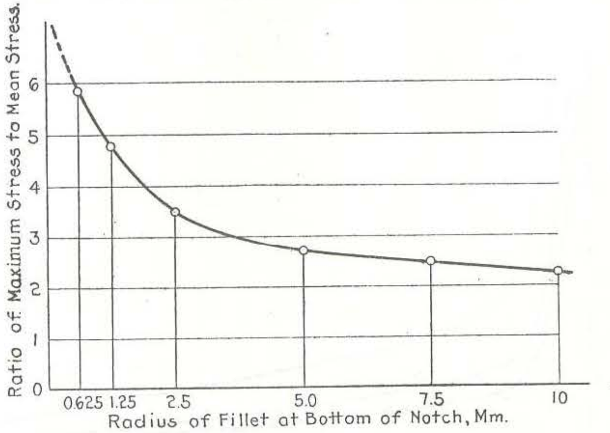


Fig. 103 Effect of Radius of Curvature at Bottom of V-Shaped Lateral Notch on Stresses in Plates

bearing upon the interpretation of certain experimental data presented in this paper.

*Tests by Means of Models Used on Account of Their Great Deflections.* The author of the paper reports a series of tests made on india-rubber wheels and very thin steel disks which were used on account of their great deflections. Attention must be called to the erroneousness of such tests when intended to yield information regarding materials or designs where those great deflections do not occur.

Let us write Lamé's general stress-strain relations:[2]

[1] Paul Heymans, La Détermination par la Photo-Elasticimetrie des Surtensions dues à Certaines Discontinuités. Mémoires, Académie Royale de Belgique, 2me. Série, t. VI, 1921.

[2] G. Lamé, Leçons sur la Théorie Mathématique de l'Elasticité des Corps Solides, 1852. A. E. H. Love, A Treatise on the Mathematical Theory of Elasticity, 3d ed., p. 97.



$$\begin{aligned}
X_x &= a_1e_{xx}+b_1e_{yy}+c_1e_{zz}+d_1e_{yz}+e_1e_{zx}+f_1e_{xy}\\
Y_y &= a_2e_{xx}+b_2e_{yy}+c_2e_{zz}+d_2e_{yz}+e_2e_{zx}+f_2e_{zy}\\
Z_z &= a_3e_{xx}+b_3e_{yy}+c_3e_{zz}+d_3e_{yz}+e_3e_{zx}+f_3e_{xy}\\
Y_z &= a_4e_{xx}+b_4e_{yy}+c_4e_{zz}+d_4e_{yz}+e_4e_{zx}+f_4e_{xy}\\
Z_x &= a_5e_{xx}+b_5e_{yy}+c_5e_{zz}+d_5e_{yz}+e_5e_{zx}+f_5e_{xy}\\
X_y &= a_6e_{xx}+b_6e_{yy}+c_6e_{zz}+d_6e_{yz}+e_6e_{zx}+f_6e_{xy}
\end{aligned}$$

These equations assume the generalized Hooke's Law that each of the six components of stress at any point of the stressed body is a linear function of the six components of strain at that point. It is seen that in this three-directional isotropic body the stress distribution is determined by thirty-six elastic constants. By introducing the conditions of isotropy in *all* directions the above equations reduce to the following:

$$\begin{aligned}
X_x &= (c_1+2d_4)e_{xx}+c_1e_{yy}+c_1e_{zz}\\
Y_y &= c_1e_{xx}+(c_1+2d_4)e_{yy}+c_1e_{zz}\\
Z_z &= c_1e_{xx}+c_1e_{yy}+(c_1+2d_4)e_{zz}\\
Y_z &= d_4e_{yz}\\
Z_x &= d_4e_{zx}\\
X_y &= d_4e_{xy}
\end{aligned}$$

Or, introducing Lamé's elastic constants $\lambda$ and $\mu$, and assuming that the cubical dilatation $\Delta$ is equal to the sum of the three linear dilatations $e_{xx}$, $e_{yy}$, $e_{zz}$, these equations take the well-known form:[1]

$$\begin{aligned}
X_x &= \lambda\Delta+2\mu e_{xx}\\
Y_y &= \lambda\Delta+2\mu e_{yy}\\
Z_z &= \lambda\Delta+2\mu e_{zz}\\
Y_z &= \mu e_{yz}\\
Z_x &= \mu e_{zx}\\
X_y &= \mu e_{xy}
\end{aligned}$$

or:

$$\begin{aligned}
e_{xx} &= E^{-1}[X_x-\sigma(Y_y+Z_z)]\\
e_{yy} &= E^{-1}[Y_y-\sigma(X_x+Z_z)]\\
e_{zz} &= E^{-1}[Z_z-\sigma(X_x+Y_y)]
\end{aligned}$$

where Young's modulus $E$ is substituted for $\dfrac{\mu(3\lambda+2\mu)}{\lambda+\mu}$ and Poisson's ratio $\sigma$ for $\dfrac{\lambda}{2(\lambda+\mu)}$.

These equations are the fundamental equations of the theory of elasticity and all theories derived therefrom. They postulate:

1 Isotropy
2 Hooke's law of linear proportionality between stress and strain

[1] A. E. H. Love, *loc. cit.*, p. 100.

3 Deformations, sufficiently small so that the square of any of the three principal dilatations or the double product of any of two of them is negligible compared with any one of them.

These relations express the stress and strain distributions for those and *only* those bodies which satisfy the three postulates. It results directly from these equations that, other things being equal, the stress and strain distributions will be the same in all such bodies; they will be different in those for which the three postulates are not satisfied. *Elastic similarity rests upon these three postulates.* When the model departs from any of these three fundamental conditions it is doubtful, in the majority of cases, if the data obtained can be *interpreted* so as to throw light upon the behavior of the structure itself, and it is certainly erroneous to transfer directly the results from the model to the structure.

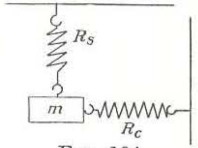

FIG. 104

*Effect of Centrifugal Force on the Transverse Vibration Frequency* (Pars. 41, 42, 43). Let us consider, as suggested by the author (Pars. 41 and 42), a particle $m$ supported by two different elastic connections $R_s$ and $R_c$ at an angle to each other as shown in Fig. 104. The frequencies due to each of these elastic connections, will be, as stated in the paper:

$$f_s = \frac{1}{2\pi}\sqrt{\frac{R_s}{m}} \qquad [1]$$

$$f_c = \frac{1}{2\pi}\sqrt{\frac{R_c}{m}} \qquad [2]$$

These two frequencies are only susceptible of direct addition, leading to a resulting frequency

$$f_r = \frac{1}{2\pi}\sqrt{\frac{R_s+R_c}{m}} \qquad [3]$$

if they affect the same degree of freedom of the system, i. e., if the elastic connections bear upon the same independent coördinate. In Fig. 104 this is not the case. It must be noted that in the case described in the paper one elastic connection corresponds to the transverse motion of the disk, whereas the centrifugal action primarily caused radial stress and strain. The two stiffnesses are *not* susceptible of direct addition. It must therefore be borne in mind, as the author of the paper seems to be well aware, that Equation [3] is only justified, as far as the centrifugal action is concerned, when $R_c$ represents the change in the transverse stiffness caused by the centrifugal force.



It is proposed, moreover, that the variation of "transverse stiffness" of the disk at rest and the rotating disk be analyzed as follows:

Let $e_{zz}$ be the transverse deformation per unit thickness at any point of the disk. The deformation $e_{zz}$ is related to the radial, tangential and transverse stress $\widehat{rr}$, $\widehat{\theta\theta}$ and $\widehat{ss}$ by the following classical stress-strain relations:

$$e_{zz} = E^{-1}[\widehat{ss} - \sigma(\widehat{rr} + \widehat{\theta\theta})]$$

where $E$ and $\sigma$ are respectively Young's modulus and Poisson's ratio.

For $\widehat{rr}$ and $\widehat{\theta\theta}$ equal to zero (disk at rest) the transverse stress will at any point produce a deformation equal to $[E^{-1}\widehat{ss}]$. The radial stress $\widehat{rr}$ and $\widehat{\theta\theta}$ (rotating disk) due to centrifugal action, and torque (and inertia in the transient state) will decrease this deformation by $\left[\frac{\sigma}{E}\widehat{rr}\right]$ and $\left[\frac{\sigma}{E}\widehat{\theta\theta}\right]$. Let $(\widehat{ss})_1$ be the stress producing unit deflection $e_{zz}$ when $\widehat{rr} = \widehat{\theta\theta} = 0$. The stress $(\widehat{ss})_2$ necessary to produce this same deflection when $\widehat{rr}$ and $\widehat{\theta\theta}$ are different from zero, becomes:

$$(\widehat{ss})_2 = [(\widehat{ss})_1 + \sigma(\widehat{rr} + \widehat{\theta\theta})]$$

This relation expresses the increase in "transverse stiffness."

It is consequently seen that the "transverse stiffness" increases *not only with the centrifugal force, but also with the torque.* The frequency $f_r$ (Equations [3] and [5] of the paper) should therefore include centrifugal action and torque. It would be interesting to know if such variations of the frequency with the torque were observed and if not, what explanation would be suggested.

*Relations between Standing Vibrations and Traveling Waves.* The statement made in Par. 59 is not free from objection. Whereas it is true that the frequency of a particle, part of a *discontinuous* medium, depends only on its mass and a stiffness factor, this property does not hold for continuous or pseudo-continuous media; that is, the frequency is *not* the same in such a medium for each unit of mass throughout the entire structure. This fact is demonstrated in the writer's paper entitled Mathematical Theory of Dynamic Stresses in Rotating Gear Pinions.[1]

*Test on a Simple Vibrating Cantilever Bar* (Pars. 129-131). The author of the paper refers to the theory of a simple vibrating cantilever bar and shows in Fig. 51 the calculated and observed frequencies of vibration of different tests. He finds that the observed frequencies check only within one or two per cent of the frequencies which he is led to expect theoretically. The writer is

[1] *Mechanical Engineering*, vol. 46, 1924, p. 583.

of the opinion that this lack of check may be due to the fact that the author's theoretical expectations are not entirely accurate. Indeed, following the classical theory, the differential equation of transverse vibratory motion of a beam of uniform rectangular cross-section is:

$$\varepsilon \frac{\partial^2 z}{\partial t^2} + \frac{Eh^2}{12} \frac{\partial^4 z}{\partial x^4} = 0 \quad . \; . \; . \; . \; . \; . \; . \quad [18]$$

where:

$z$ = transverse displacement
$\varepsilon$ = density
$E$ = Young's modulus
$h$ = height of the beam
$x$ = longitudinal coördinate.

If the beam is " encastré " at one end and free at the other, then:

$$(z)_{x=0} = 0, \quad \left(\frac{\partial z}{\partial x}\right)_{x=0} = 0 \quad . \; . \; . \; . \; . \; . \; . \quad [19]$$

$$\left(\frac{\partial^2 z}{\partial x^2}\right)_{x=l} = 0, \quad \left(\frac{\partial^3 z}{\partial x^3}\right)_{x=l} = 0 \quad . \; . \; . \; . \; . \; . \; . \quad [20]$$

These two last conditions [20] express that at the free end the beam has neither curvature nor variation of curvature.

For simplicity assume that $l = 1$. The Equations [20] become:

$$\left(\frac{\partial^2 z}{\partial x^2}\right)_{x=1} = 0, \quad \left(\frac{\partial^3 z}{\partial x^3}\right)_{x=1} = 0 \quad . \; . \; . \; . \; . \quad [21]$$

For integrating Equation [18], we can write, in the classical manner, as solutions:

$$z = \phi(x) \cos nt \text{ or } z = \phi(x) \sin nt \quad . \; . \; . \; . \; . \quad [22]$$

where $n$ is to be determined.

Equations [22] and their derivatives introduced in [18], [19] and [20] give the following relations which determine the function $\phi$:

$$\left.\begin{array}{ll} (a) \quad \dfrac{d^4\phi}{dx^4} - \lambda\phi\,(x) = 0 & \text{where } \lambda = \dfrac{12\varepsilon n^2}{Eh^2} \\ (b) \quad \phi\,(0) = \phi'\,(0) = 0 & \\ (c) \quad \phi''(1) = \phi'''(1) = 0 & \end{array}\right\} \; . \; . \quad [23]$$

Equation [23(*a*)] is a linear and homogeneous differential equation with constant coefficients, whose solution is of the form

$$\phi(x) = e^{\tau x} \quad . \; . \; . \; . \; . \; . \; . \; . \; . \quad [24]$$

where $\tau$ is determined by introducing solution [24] into the primitive Equation [23(*a*)]

$$\tau^4 - \lambda = 0$$



whence $\tau = \pm\sqrt{\pm\sqrt{\lambda}}$

or
$$\tau_1 = +\sqrt[4]{\lambda}$$
$$\tau_2 = -\sqrt[4]{\lambda}$$
$$\left.\begin{array}{l} \tau_3 = +i\sqrt[4]{\lambda} \\ \tau_4 = -i\sqrt[4]{\lambda} \end{array}\right\} \text{where } i = \sqrt{-1}$$

Putting $s = +\sqrt[4]{\lambda}$, the solutions of Equation [24] are:

$$\left.\begin{array}{l} \phi_1 = e^{+sx} \\ \phi_2 = e^{-sx} \\ \phi_3 = e^{+isx} \\ \phi_4 = e^{-isx} \end{array}\right\} \; . \; . \; . \; . \; . \; . \; . \; . \; . \; . \quad [25]$$

Equation [23(*b*)] being linear, any linear combination of the solutions [25] will also be solutions of the primitive equation. The following combinations represent the real solutions:

$$\frac{\phi_1+\phi_2}{2}, \quad \frac{\phi_1-\phi_2}{2}, \quad \frac{\phi_3+\phi_4}{2}, \quad \frac{\phi_3-\phi_4}{2}$$

Indeed

$$\frac{\phi_1+\phi_2}{2} = \frac{e^{sx}+e^{-sx}}{2} = \cosh(sx)$$

$$\frac{\phi_1-\phi_2}{2} = \frac{e^{sx}-e^{-sx}}{2} = \sinh(sx)$$

$$\frac{\phi_3+\phi_4}{2} = \frac{e^{isx}+e^{-isx}}{2} = \cos(sx)$$

$$\frac{\phi_3-\phi_4}{2} = \frac{e^{isx}-e^{-isx}}{2} = \sin(sx)$$

Hence we can write:

$$\phi_1(x) = \cosh(sx)$$
$$\phi_2(x) = \sinh(sx)$$
$$\phi_3(x) = \cos(sx)$$
$$\phi_4(x) = \sin(sx)$$

and the general solution for $\phi$ is

$$\phi(x) = A \cosh(\sqrt[4]{\lambda}x) + B \sinh(\sqrt[4]{\lambda}x) + C \cos(\sqrt[4]{\lambda}x) + D \sin(\sqrt[4]{\lambda}x) \quad . \; . \; . \; . \; . \; . \; . \; . \; . \; . \; . \quad [26]$$

where the values of $A$, $B$, $C$, $D$ and $\lambda$ satisfy the boundary conditions [23(*b*)] and [23(*c*)].

These boundary conditions introduced in Equation [26] yield the following condition equation:

$$\cosh s \cos s = -1$$

The roots of the transcendental equation $\cosh s \cos s = \pm 1$ are given in Jahnke-Emde: [1]

$$x_k = \tfrac{1}{2}(2k \pm 1)\pi - (-1)^k \alpha_k$$

[1] E. Jahnke-F. Emde, Funktionentafeln mit Formeln und Kurven, 1923, p. 3.

where $\alpha_k \doteq \frac{2}{a} \pm (-1)^k \frac{4}{a^2} + \frac{34}{3a^3} \pm (-1)^k \frac{112}{3a^4} + \ldots$, and

$$a = e^{(2k+1)}\frac{\pi}{2}$$

The roots of the equation cosh $s$ cos $s = -1$ consequently are: $s_1 = 1.8751$, $s_2 = 4.6941$, $s_3 = 7.8548$, $s_4 = 10.9955$, and where $k > 4$, the values of $s$ can be obtained with satisfactory approximation from the expression:

$$s_k = \tfrac{1}{2}(2k-1)\pi$$

These values of $s$ represent, according to the relation $s = \sqrt[4]{\lambda}$, the fourth power of $\lambda$. From the relation

$$\lambda = \frac{12\varepsilon n^2}{Eh^2}$$

we thereby obtain the values of $n$:

$$n_k = \sqrt{\frac{Eh^2}{12\varepsilon}\lambda_k} = s_k^2\sqrt{\frac{Eh^2}{12\varepsilon}}$$

This gives as a general solution of Equation [18] with the condition Equations [19] and [21]:

$$z = \sum_{k}^{k=1,2,\ldots,\infty} \phi_k(x)\,(\alpha_k \cos n_k t + \beta_k \sin n_k t) \quad . \; . \; . \quad [27]$$

where $\alpha_k$, and $\beta_k$ are constants which can be derived from the conditions of the system at the origin or at any other time.

*It is thereby seen that the kinetic equation of motion assumed by the author of the paper is only a first approximation,* the complete solution being given by Equation [27] where the values of the frequencies of the component oscillations are:

$$n_1 = \sqrt{\frac{Eh^2}{12\varepsilon}}\ 1.8751^2$$

$$n_2 = \sqrt{\frac{Eh^2}{12\varepsilon}}\ 4.6941^2$$

$$n_3 = \sqrt{\frac{Eh^2}{12\varepsilon}}\ 7.8548^2$$

$$n_4 = \sqrt{\frac{Eh^2}{12\varepsilon}}\ 10.9955^2$$

$$\vdots$$

$$n_k = \sqrt{\frac{Eh^2}{12\varepsilon}}\ \tfrac{1}{2}(2k-1)^2\pi^2$$



E. D. DICKINSON.[1] When the potential possibilities of the steam turbine came to be realized, there was a demand for sizes heretofore not considered practical or commercial. To supply this demand and to obtain simultaneously better efficiencies called for new standards of design. Mechanical design will be sound only when the fundamentals are understood. The paper under discussion shows, in a most logical and thoroughly analytical manner, how the explanation has been found for heretofore inexplainable failures in turbine wheels. With proper application of this additional knowledge, the likelihood of failure has been reduced to the minimum.

Referring to Table 1, it is the writer's understanding that the number of wheels in every case refers to single-row wheels and not to wheels with relatively wide rims for two or more rows of buckets. This table refers to turbines of over 5000 kw. capacity. The majority of turbines of 5000 kw. and smaller operate at higher speeds and are fitted with stiffer wheels of smaller diameter. There had been no indication of vibration of these smaller wheels. However, an exhaustive investigation was undertaken of all single-row turbine wheels as shown in Table 6.

TABLE 6 FOR TURBINES UNDER 5000 KW. INSTALLED BEFORE NOVEMBER, 1923

| | | |
|---|---|---|
| No. of wheels installed | Single Row | 3463 |
| | Double Row | 1307 |
| | Total | 4770 |
| No. of wheels tested (standing) | | 170 |
| No. of wheels rotated in wheel-testing machine | | 10 |
| No. of tests in wheel-testing machine | | 15 |
| No. of wheels tested in customers' plants (standing) | | 39 |
| No. of machines investigated in customers' plants | | 11 |
| No. of machines tested under load | | 1 |
| No. of wheels replaced to avoid possible trouble | | 8 |
| No. of wheels tuned for vibration | | 8 |

Referring to Item 5, Table 2, and to Pars. 198 and 199: While it is true that this wheel let go at approximately 2800 r.p.m. which is 48 r.p.s. and at this speed the wheel showed six nodes, it is also true that the turbine in all probability operated for a while at 3900 r.p.m. or 65 r.p.s. At this speed the wheel would be subject to 4-node vibration. It is therefore probable that the fracture was started while operating at the higher speed and was the direct result of 4-node vibration. A crack once started, it is to be expected that the wheel would ultimately fail. Vibration at 2800 r.p.m. would hasten the progress of the fracture. This is the only case on record of the failure of one of the smaller wheels which might be attributed to lateral vibration. The amplitude of the 6-node vibration of the smaller wheels is so minute that it

[1] Designing Engineer, Turbine Engineering Department, River Works, General Electric Company, West Lynn, Mass. Mem. A. S. M. E.

does not constitute an element of danger. In Par. 124 reference is made to a value of $y_0$ of $\frac{1}{4}$ in., which means an amplitude of vibration of approximately $\frac{1}{2}$ in. This refers to a wheel of relatively large diameter. In the smaller-diameter wheels when vibration tests were conducted with a magnet similar to that used on the large wheels, it was in many instances difficult to get an amplitude of vibration sufficient to give positive indications with the oscillograph. In referring to this it is not the intention to give the impression that small wheels are safer than large, but merely to bring out the fact that when designed for strength, they are inherently stiffer. Exhaustive investigations showed clearly that 6-node vibration of the smaller wheels contained no element of danger.

The smaller diameter wheels designed for 3600 r.p.m. have a 4-node major well above the running speed. Tests on a large number of wheels are very consistent. It is present practice to test two wheels of every new design as brought out.

The relative advantages and disadvantages of the so-called stiff shaft referred to in Par. 9 might well be the subject of another paper. Stiff shafts have the first critical above the running speed. There are in commercial service several thousand turbines with shafts having the first critical below the operating speed. These turbines have given most excellent account of themselves on the score of mechanical operation.

It is impossible to overestimate the value of the author's contribution. With thorough knowledge of the precautions that must be taken in a design, it is possible to minimize the liabilities of failure. So long as some of the failures referred to by the author were still unexplained, it is easily understood why many people considered the operation of turbines, and especially the larger ones, as hazardous. At the present time there is no more hazard in the operation of large steam turbines designed in accordance with the best practice than there is in the operation of steam boilers, oil engines, or any other piece of apparatus in which many of the component parts are subject to high stresses.

Roger D. De Wolf.[1] It seems to be a question as to whether it is necessary to have an exact equality of speed between the traveling wave and the speed of rotation. In Table 2, Item 2, there is a difference of 7 per cent between the backward speed of the wave and the operating speed of the wheel. Item 4 of the same table shows a difference of 5 per cent. In both cases the wheel broke. In Item 8, the second case of bucket failure, the difference in speed of the wave and the wheel was 8.7 per cent. These figures would indicate either that it is not necessary for the backward speed of the rotating wave to be exactly the same as the speed of rotation, or that the company is operating the machine for a considerable length of time at speeds different from that at which it was supposed to be operated.

[1] Chief Operating Engineer, Rochester Gas & Electric Corporation, Rochester, N. Y. Mem. A. S. M. E.



In addition to the vibrations that may be set up at the critical speed, the author treats of other vibrations that may be set up at minor resonant speeds. The writer understands that these vibrations would not be stationary in space, and would inquire if they continue after being set up, and if they are of such amplitude as to engender serious fatigue in the metal of the wheel.

Closure.[1] Dr. Timoshenko apparently fails to appreciate that a high degree of accuracy in the theoretical calculation of turbine-disk vibrations is futile as regards actual wheels. The author tried to make it clear that there is no lack of satisfactory theory and that, in the laboratory, both reeds and disks can be made to agree with theory. The paper has not been concerned with the shapes of wheel that can be accurately calculated, but has rather devoted itself to an exposition of the difficulties encountered in actual manufacture and a discussion of the divergences from theory which have never been appreciated before.

For instance, there are many variable factors in the problem of frequency calculation which are not susceptible of theoretical expression, such as the state of internal stress and the tightness of bucket fit, which have important effects upon the vibration frequency and which, if neglected, vitiate the results of the most exact theoretical calculations.

Southwell's conclusion that $f_r^2 = f_s^2 + f_c^2$, mentioned by Dr. Timoshenko, is interesting in view of the fact that, as noted in the paper, it is approximately confirmed in the case of many wheels tested.

As regards the uncertainty of stress determination, Dr. Timoshenko fails to realize the difficulty of determining in a turbine the actual maximum deflection itself. In comparison with this difficulty the determination of the shape of the deflection curve and its necessary derivatives is a mere detail. However, in a turbine the amplitude cannot exceed the clearance, and this *limit* alone permits useful calculations to be made even if a higher degree of accuracy might be desired.

Dr. Timoshenko's closing remarks are obviously due to haste incident to the short time available for consideration of the paper. The discrepancies in the case of circular plates became large for

[1] Owing to Mr. Campbell's untimely death very shortly after the presentation of this paper, the closure is prepared entirely by Messrs. Kimball and Robinson, but includes the remarks made by Mr. Campbell at the meeting in answer to Mr. DeWolf. The short time allowed at the meeting prevented the author from replying in person to any other discussion at that time.

thin plates or plates of large diameter. Those of moderate proportions, as shown in the plottings, agreed with theory. In view of the many photographs reproduced in the paper, the author hardly thought it necessary to state explicitly that in all cases the disks were mounted at the center as in a turbine and were entirely free at the edge. The actual amplitudes in sample cases may be scaled from the smoked-glass curves presented in Fig. 56 and by reference to Figs. 57 and 58. As a matter of fact, no significant variations of frequency have been observed with amplitudes so great as to be accompanied by bending stresses equal to the elastic limit.

Professor Moore has noted that the actual problem was to devise such means that axial vibration would not occur. He notes two important conclusions from his investigation of the fatique of metals. Although the present paper shows how dangerous vibrations are guarded against, still it is a fact that the stresses at steam balance holes in wheel webs are carefully limited and in addition the region about such holes is well polished to remove tool marks.

Dr. Heymans, in his contribution, first discusses the nature of the stress about a circular hole in an infinitely extending plate, giving the formulas for the stress distribution. It is interesting to note that these are the standard formulas used by the General Electric Company for calculations of stresses about holes in turbine wheels. It is pointed out that " ruptures run or start at the holes at the two ends of diameters very approximately coinciding with the normal to the direction of the centrifugal force." This fails to recognize that the centrifugal force, although radial often produces tangential stresses which are *greater* than the radial. As a matter of fact, the directions of the ruptures are determined by the superposition of the vibrational stresses upon the centrifugal stresses and by using the Goodman diagram for repeated stress other than reversed stress as determined by Professor Moore for the Joint Investigation on the Fatigue of Metals.

As regards the use of the photoelastic method of analysis for local stresses in internal angles and fillets, it can be said that the entire line of standard dovetails used by the General Electric Company has been analyzed by this method in comparison with a large variety of special dovetail designs, and shown to be capable of very little improvement.

The stress-strain formulas presented by Dr. Heymans are the standard ones which, as he notes, may be found in any work on the theory of elasticity. They do not apply to rubber models unless the strains are comparatively small. In the stress experiments on rubber models cited, the results give an approximate idea of the stress distribution, as was verified from comparisons with values calculated by the elastic theory using the same formulas mentioned by Dr. Heymans for stresses about a circular hole.



As regards the vibrations of thin disks even though the deflections are seemingly large, the classical theory of elasticity gives good results. A few simple experiments with vibrating disks and vibrating reeds show no perceptible changes of frequency with amplitude, as should be the case if the vibrational strains were large enough to make the standard elastic theory invalid.

Regarding the reference to Pars. 41 and 42, the two elastic connections $R_s$ and $R_c$ are not to be regarded as so applied as to give the vibrating mass two degrees of freedom as shown in Fig. 104. There is perhaps an ambiguity in the first line of Par. 42 which led to a misunderstanding of this point, but the way the two elastic constants $R_s$ and $R_c$ are combined in calculating the new frequency $f_r$ shows that only one degree of freedom is concerned, as Dr. Heymans himself concedes.

Regarding the remarks on " transverse stiffness," the use of this expression is evidently misunderstood. " Transverse stiffness " as used in the paper means resistance to transverse bending and not resistance to transverse elastic strains as set forth in Dr. Heymans' discussion in the two paragraphs beginning with " Let $e_{zz}$ be the . . . etc." Furthermore, the transverse stress *ss* mentioned in these paragraphs is purely hypothetical, as no such stress exists in the ordinary theory of disk-wheel stresses which treats the problem purely as a case of plane stress. In view of the misunderstanding as to transverse stiffness, the remarks on the effect of torque upon transverse stiffness do not apply.

In his objection to Par. 59 Dr. Heymans states that the frequency of a vibrating body is *not* the same for each unit of mass throughout the entire structure. While this statement is, mathematically speaking, true in the case of actual turbine wheels, one mode of vibration predominates to the practical exclusion of all others, as simple tests show, such as the sand pictures of Figs. 18 to 24 in the paper.

Referring to Dr. Heymans' remarks on a simple cantilever bar, he is incorrect in his statement that " the author's theoretical expectations are not entirely accurate." The classical theory which he presents is exactly that used by the author, and his and the author's formulas (Par. 129) are identical for the fundamental mode of vibration, except that Dr. Heymans assumes the bar to be of unit length, and has expressed his frequencies in radians per second instead of in cycles per second. The slight variations of the experiments on bars from theory are erratic and are the result of the difficulty of exactly realizing in the experiments the clamping conditions required by theory, and are not due to faulty theory. It has not been the intention of this paper to discuss at length the vibration of reeds and bars, but in view of the discussion it may be noted that experiments show it equally possible to have a pure vibration at the higher frequency of what Dr. Heymans calls the " second component oscillation," and to have

this independently of the first or fundamental type. Dr. Heymans' formulas merely show the possibility of the various independent types, but his contention that they necessarily all exist at once, while mathematically true, does not hold in practical cases.

The information presented by Mr. Dickinson in regard to small sizes of turbines is a valuable contribution.

Mr. DeWolf's question as to the difference between the speed of the backward-traveling wave and the speed of rotation of the wheel, is covered by what is called the "broadness of resonance." While vibration can be excited somewhat off the actual coincidence, tests show that vibrations do not build up unless the running speed is within a margin of two per cent of the wheel critical speed. The wave speeds given in Table 2, to which Mr. DeWolf refers, were made up from various sources and, especially in these early cases, had to be estimated by calculation or reference to similar designs because no actual records of the wheel characteristics were on file. The greater differences of this table are thus due to error of calculation or estimate, and not to excessive broadness of resonance.

In regard to minor resonant speeds, it may be said that waves which are not stationary in space have been produced in the wheel-testing machine, and that whereas a fixed-pressure spot will hold a stationary wave, an unbalance will give a minor resonance. If the unbalance moves the shaft in a horizontal plane with one exciting impression per revolution, and if the wave was started and happened to be of the correct velocity so that this one pressure per revolution was in resonance with it as it passed the particular plane of unbalance, the vibration could be excited. This is done in the wheel-testing machine by attaching a large piece of iron to the shaft, which causes one end of the shaft to move horizontally since the horizontal stiffness of the bearing is much less than the vertical stiffness. Although the possibility of the maintenance of such waves by unbalance has been thus demonstrated, it has never been possible to cause minor vibrations of serious amplitude. Minor resonant vibrations are associated with rough running, and the possibility of such resonance is not considered serious in machines in good operating condition.

No. **1921**

# TEMPERATURE AND STRESS DISTRIBUTION IN HOLLOW CYLINDERS

## APPLICATIONS TO BOILER TUBES, CYLINDER LINERS, AND ORDNANCE

By O. G. C. Dahl,[1] Cambridge, Mass.
Non-Member

*This paper contains an analysis of temperature and stress distribution in hollow cylinders. The results should be applicable to tubes, cylinder liners and ordnance.*

*Whenever a difference in temperature exists between the two walls of a hollow cylinder, stresses are set up. The magnitude of the temperature stresses depends upon the magnitude of the temperature difference and upon its variation with time. The resultant stresses to which a cylinder is subjected are the sum of the temperature stresses and the stresses due to forces and pressures.*

*The analysis has been limited to the case where the distribution of temperature is symmetrical with respect to the axis of the cylinder and also independent of distance parallel to the axis. In other words, the temperature at any point is assumed to be a function of radius and time only.*

*Complete solutions are presented for the case where the temperature difference between the two walls is constant. Steady-state solutions are presented for cases where the temperature difference varies sinusoidally with time, where it consists of a sinusoidally varying temperature superimposed upon a constant temperature, and where it undergoes any cyclic variation expressible as a Fourier series.*

*Numerical examples of computing the stresses in boiler tubes and in the cylinder liner of a Diesel engine are given and the results discussed.*

IT IS a well-known fact that stresses may be set up when differences in temperature exist between points in a piece of metal, depending upon whether or not it is free to expand. If the piece of metal under consideration constitutes a part of an engine, it is very likely that its capability of expanding is limited. It may perhaps expand freely in one direction while it is prevented from doing so in other directions. In such cases stresses due to temperature will be produced in addition to the stresses due to forces or pressures.

[1] Massachusetts Institute of Technology.

Presented at the Spring Meeting, Cleveland, Ohio, May 26 to 29, 1924, of The American Society of Mechanical Engineers.

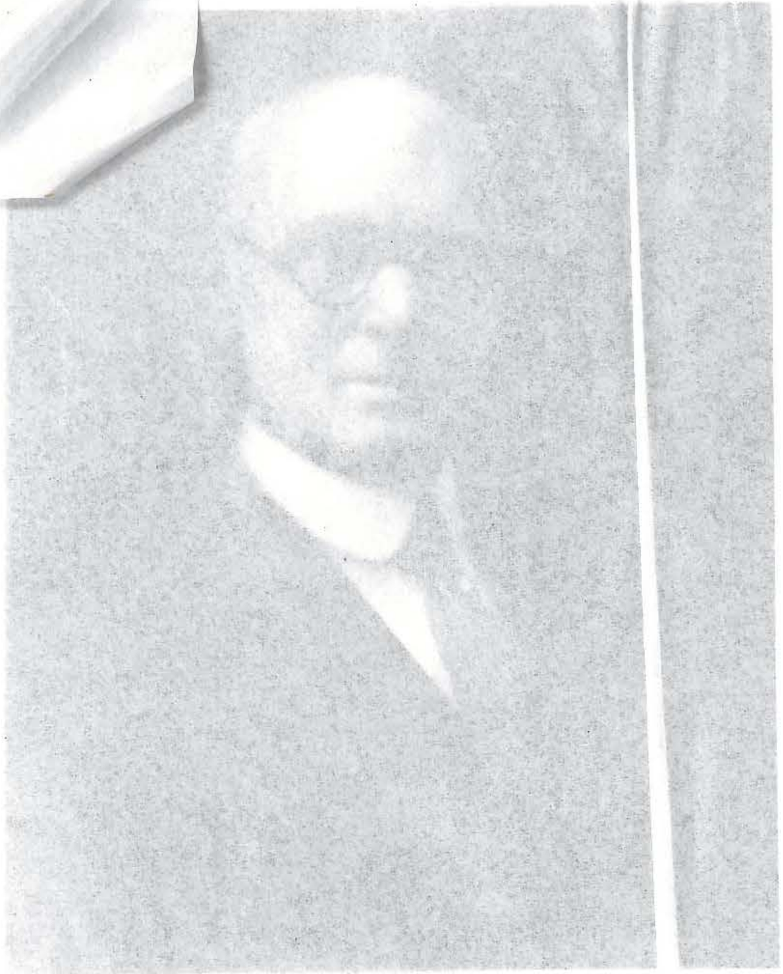

P429 P428.G.151.46
PPC.4205.46

# TRANSACTIONS

VOLUME 46

CLEVELAND MEETING
NEW YORK MEETING
1924

[illegible] LIBRARY
SEP 8
1925
CAMBRIDGE

PUBLISHED BY
THE AMERICAN SOCIETY OF MECHANICAL ENGINEERS
29 WEST 39TH STREET, NEW YORK
1925

P429 P428.G151.46
PPC.1205.46

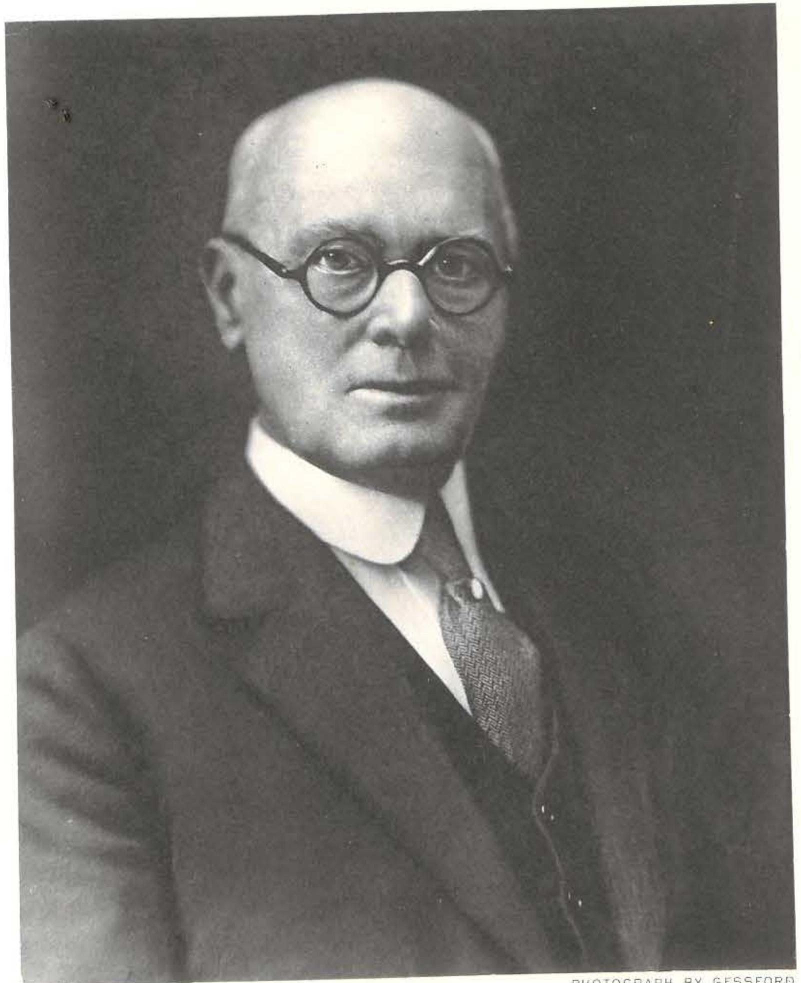

ANDERSEN-LAMB, CO. N. Y. PHOTOGRAPH BY GESSFORD

F. R. Low

PRESIDENT 1924
OF
THE AMERICAN SOCIETY OF MECHANICAL ENGINEERS

UNIVERSITY LIBRARY
SP 8 1925
CAMBRIDGE

# TRANSACTIONS

## VOLUME 46

CLEVELAND MEETING
NEW YORK MEETING
1924

PUBLISHED BY
THE AMERICAN SOCIETY OF MECHANICAL ENGINEERS
29 WEST 39TH STREET, NEW YORK
1925

Copyright, 1925, by
THE AMERICAN SOCIETY OF MECHANICAL ENGINEERS

# PUBLICATIONS COMMITTEE'S FOREWORD

IN SCOPE and size this volume of Transactions marks a return to normal. It reports the Spring and Annual Meetings more adequately than has been possible in preceding years and it includes a larger percentage of the papers presented at those meetings. In selecting the papers the Committee, as always, has been guided by the principle of choosing material of permanent technical value, and the discussions have been carefully edited.

The 1924 Transactions departs somewhat from the arrangement which has been followed for some years, in that the list of officers and committees, and the statement of the membership of the Society which has formerly appeared in the front of the book on pages with Roman numeral folios, is now a part of the Society Affairs.

In the index to the volume another change has been made. Spring and Annual Meeting papers printed in *Mechanical Engineering* but not in this volume, Section papers, technical reports, and other important contributions to *Mechanical Engineering* during 1924, are listed on pages immediately following the main index to the volume instead of being included within that index.

This page replaces the introductory note which has always preceded the biographical sketch of the president, and a statement by the Committee which has sometimes been inserted on a loose sheet in the volume.

A. G. Christie, *Chairman*
J. T. Wilkin
O. G. Dale
R. E. Flanders
Kenneth H. Condit
*Publications Committee.*

# CONTENTS OF VOLUME 46 [1]

CLEVELAND AND NEW YORK MEETINGS


| No. | | PAGE |
|---|---|---|
| 1918 | Biography of Fred R. Low | |
| 1919 | Society Affairs | 5 |
| 1920 | WILFRED CAMPBELL, The Protection of Steam-Turbine Disk Wheels from Axial Vibration | 7 |
| 1921 | O. G. C. DAHL, Temperature and Stress Distribution in Hollow Cylinders | 31 |
| 1922 | A. C. DANKS, The Gas Engine in the Steel Industry | 161 |
| 1923 | A. L. DE LEEUW, Analysis of a Machine-Shop Problem on a Quantity and Final-Economy Basis | 209 |
| 1924 | W. L. R. EMMET, The Emmet Mercury-Vapor Process | 227 |
| 1925 | CARL J. FECHHEIMER, Performance of Centrifugal Fans for Electrical Machinery | 253 |
| 1926*a* | L. W. SPRING, Industrial Applications of Metals at Various Temperatures | 287 |
| 1926*b* | V. T. MALCOLM, Methods of Testing at Various Temperatures and Their Limitations | 351 |
| 1926*c* | H. J. FRENCH and W. A. TUCKER, Available Data on the Properties of Irons and Steels at Various Temperatures | 356 |
| 1926*d* | CLAIR UPTHEGROVE and A. E. WHITE, Available Data on the Properties of Non-Ferrous Metals and Alloys at Various Temperatures | 399 |
| 1927 | FRED R. LOW, Power Resources, Present and Prospective | 433 |
| 1928 | Council Report | 535 |
| 1929 | W. E. BLOWNEY and G. B. WARREN, The Increase in Thermal Efficiency Due to Resuperheating in Steam Turbines | 547 |
| 1930 | HENRY KREISINGER, A Review of Recent Applications of Powdered Coal to Steam Boilers | 563 |
| 1931 | W. A. SHOUDY and R. C. DENNY, Recent Developments in the Burning of Anthracite | 595 |
| 1932 | GEORGE D. BABCOCK, Production Control | 639 |
| 1933 | RALPH E. FLANDERS, Design, Manufacture, and Production Control of a Standard Machine | 667 |
| | | 691 |


[1] The Society shall not be responsible for statements or opinions advanced in papers or in discussion at meetings of the Society or of its Divisions or Sections, or printed in its publications.

| No. | | Page |
|---|---|---|
| 1934 | W. H. Carrier and Daniel C. Lindsay, The Temperatures of Evaporation of Water into Air | 739 |
| 1935 | Sanford E. Thompson and H. T. Rollins, The Development of a Modern Hosiery Plant | 781 |
| 1936 | H. A. S. Howarth, A Graphical Study of Journal Lubrication (Part II) | 809 |
| 1937 | Louis Illmer, High-Pressure-Bearing Research | 833 |
| 1938 | Leonard N. Linsley, An Investigation of the Critical Bearing Pressures Causing Rupture in Lubricating-Oil Films | 855 |
| 1939 | Lloyd J. Franklin and Charles H. Smith, The Effect of Inaccuracy of Spacing on the Strength of Gear Teeth | 885 |
| 1940 | Joseph K. Wood, Mechanical Springs | 915 |
| 1941 | R. Eksergian, The Strength and Proportions of Wheels, Wheel Centers and Hubs | 929 |
| 1942 | H. Loring Wirt, An Experimental Investigation of Nozzle Efficiency | 981 |
| 1943 | Charles E. Lucke, Large Oil Engines, with Special Reference to the Double-Acting Two-Cycle Type | 1005 |
| 1944 | Lionel S. Marks and M. Danilov, Gas Turbines | 1095 |
| 1945 | Robert W. Angus, Intakes for Power Plants | 1131 |
| 1946 | H. L. Doolittle, A Method for the Economic Design of Penstocks | 1165 |
| 1947 | H. Zoelly, The Zoelly Turbine-Driven Locomotive | 1205 |
| 1948 | Charles E. Lucke, The Value of Efficiency in Transforming and Distributing Energy | 1245 |
| | Technical Committee Reports | 1285 |
| 1949 | Necrology | 1287 |
| 1950 | Index | 1331 |




# FRED R. LOW

## PRESIDENT OF THE SOCIETY FOR 1924

FRED R. LOW was born in Chelsea, Mass., in April, 1860. His education was received in the public schools of that city.

As a boy Mr. Low found a position with the Western Union Telegraph Company in Boston. He learned both telegraphy and stenography and for several years was a court and commercial stenographer. In 1880 he became stenographer to the editor of the Boston *Journal of Commerce*. He remained with this journal until 1888, part of the time as editor of the steam-engineering department. During his years with this paper, a time when attention was beginning to be directed strongly to steam-engine economy, he operated with Frank M. Clark, the Clark & Low Machine Company, and was co-inventor with Mr. Clark of a flue cleaner for vertical boilers, an integrating steam-engine indicator, an elevator control, a rotary engine, etc.

Since 1888 Mr. Low has been editor of *Power*. In this capacity he has attained distinction not only as an editor but as an authority on power-plant subjects. His lectures during the early days of *Power*, delivered before meetings of engineers and published monthly, were of inestimable educational value. More recently his editorials have achieved a permanent place in engineering literature. He is the author of The Power Catechism, The Compound Engine, Condensers, and The Steam Engine Indicator.

Mr. Low has been a member of the Society since 1886 and is one of its most active workers. In 1909 he was chairman of the Gas Power Section which was organized the preceding year and was the first professional division of the Society. He was a vice-president from 1918 to 1920, and president for the year 1923-24. Since 1917 he has been a member of the Boiler Code Committee. He was a member of the organizing committee of the Fuels Division and chairman of its Executive Committee during 1922-1923. From 1912 to 1917 he was a member of the Publications Committee of the Society. Probably his most outstanding contribution to the work of the Society has been in connection with the formulation of its Power Test Codes. He has served as chairman of this committee ever since its organization in 1918. He is also the Society's representative on the American Engineering Council.

Mr. Low is an honorary member of the Institution of Mechanical Engineers of Great Britain, the Marine Engineers Beneficial



UNIVERSITY LIBRARY B SP 8

Association, and the National Association of Practical Refrigerating Engineers, and a member of the American Association for the Advancement of Science, the National Association of Stationary Engineers, and the Verein Deutscher Ingenieure. At the centennial celebration of Rensselaer Polytechnic Institute in October, 1924, the degree of Doctor of Engineering was conferred upon him.

No. 1919

# SOCIETY AFFAIRS

## ORGANIZATION AND MEMBERSHIP

ON THE following pages are given the names of those who made up the executive and administrative personnel of the Society, its representatives on joint activities, and a summary of its membership for the year 1924. The personnel of professional committees and divisions, Local Sections officers, and detailed information concerning the organization of the Society was printed in the Year Book for 1924.

## OFFICERS AND COUNCIL

### PRESIDENT

Fred R. Low........New York, N. Y.

### VICE-PRESIDENTS

*Terms Expire December, 1924*

W. S. Finlay, Jr........New York, N. Y.
Wm. H. Kenerson........Providence, R. I.
Earl F. Scott........Atlanta, Ga.

*Terms Expire December, 1925*

George I. Rockwood........Worcester, Mass.
W. J. Sando........Milwaukee, Wis.
H. Birchard Taylor........Philadelphia, Pa.

### MANAGERS

*Terms Expire December, 1924*

Sherwood F. Jeter........Hartford, Conn.
H. P. Liversidge........Philadelphia, Pa.
Hollis P. Porter........Tulsa, Okla.

*Terms Expire December, 1925*

A. G. Christie........Baltimore, Md.
James H. Herron........Cleveland, Ohio
Roy V. Wright........New York, N. Y.

*Terms Expire December, 1926*

E. O. Eastwood........Seattle, Wash.
E. R. Fish........St. Louis, Mo.
Frank A. Scott........Cleveland, Ohio
