# Supplementary material for: Non-cuttable material created through local resonance and strain rate effects
Source: Sci Rep. 2020 Jul 20;10:11539. doi: 10.1038/s41598-020-65976-0 (PMC7371712; doi:10.1038/s41598-020-65976-0)

Non-cuttable material created through local resonance and strain rate effects

Stefan Szyniszewski [1], Rene Vogel [2], Florian Bittner [3,4], Ewa Jakubczyk[5], Miranda Anderson [6], Manuel Pelacci[5], Ajoku Chinedu [5], Hans-Josef Endres [3,4], Thomas Hipke [2]*

[1] Durham University, Durham, United Kingdom
[2] Fraunhofer Institute for Machine Tools and Forming Technology IWU, Chemnitz, Germany
[3] Fraunhofer Institute for Wood Research, Wilhelm-Klauditz-Institut WKI, Hannover, Germany
[4] Leibniz University Hannover, Institute of Plastics and Circular Economy IKK, Garbsen, Germany
[5] University of Surrey, United Kingdom
[6] University of Stirling, United Kingdom

Supplementary Information C:
Set of Scanning electron microscopy (SEM) micrographs

Fig.1. SEM image of the surface morphology of the ceramic spheres, showing the low densification of a low purity ceramic structure.

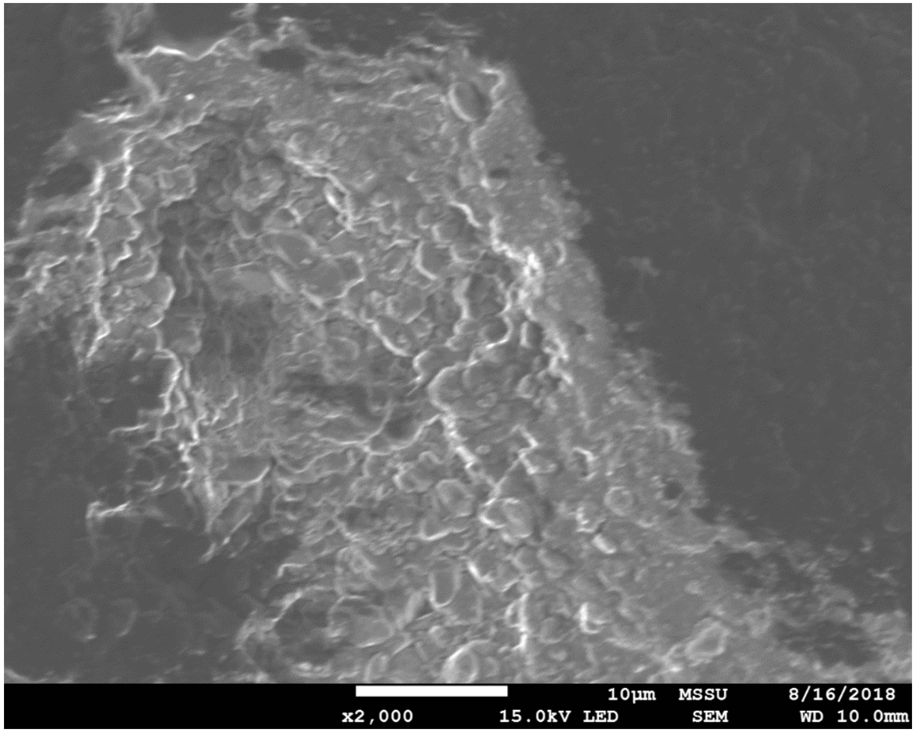

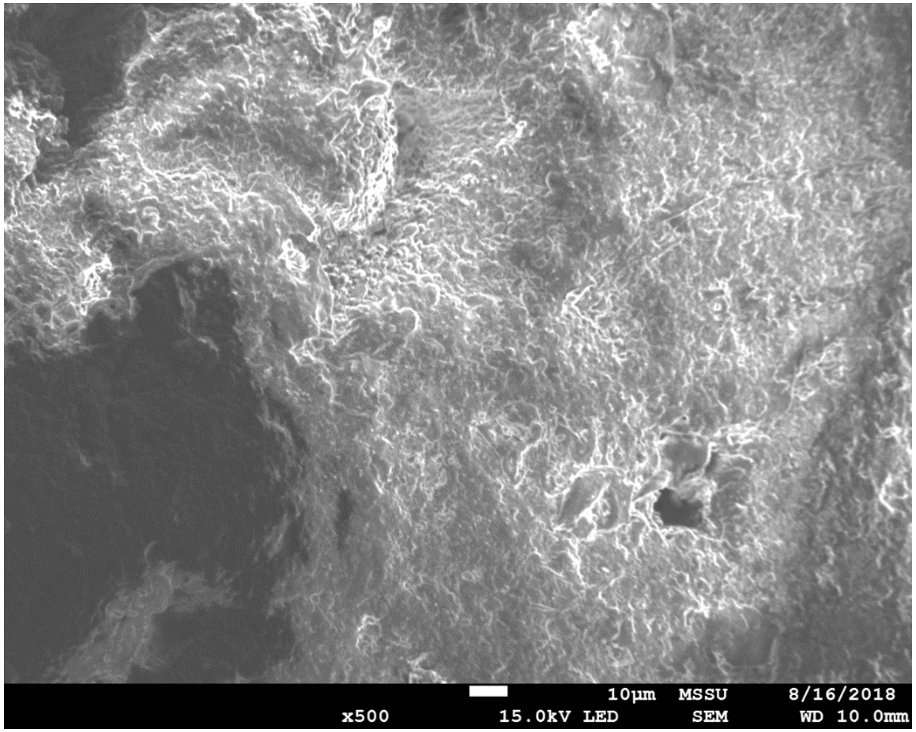

Fig.2. SEM image of the cross sectional morphology of the ceramic spheres.

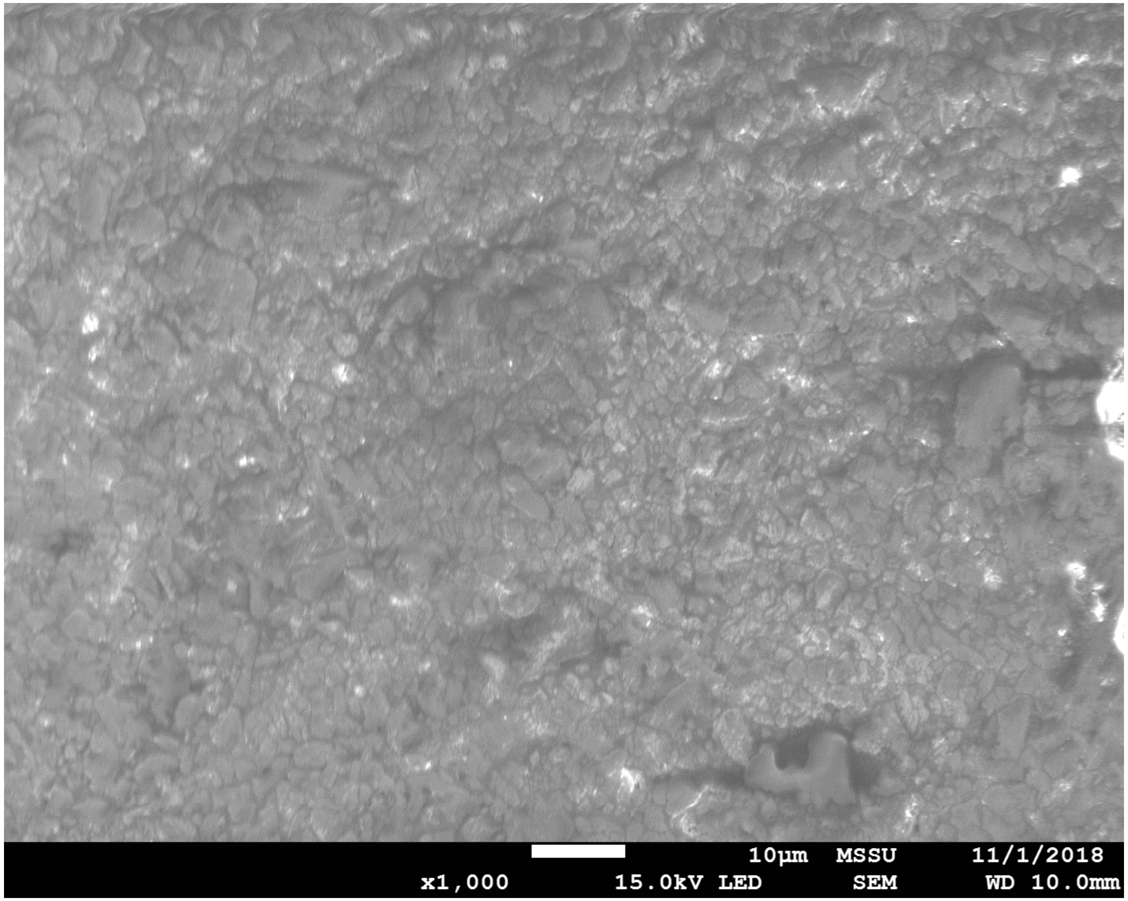

Fig. 3. Scanning electron micrograph of the extracted powder from a partial incision of our cylinder.

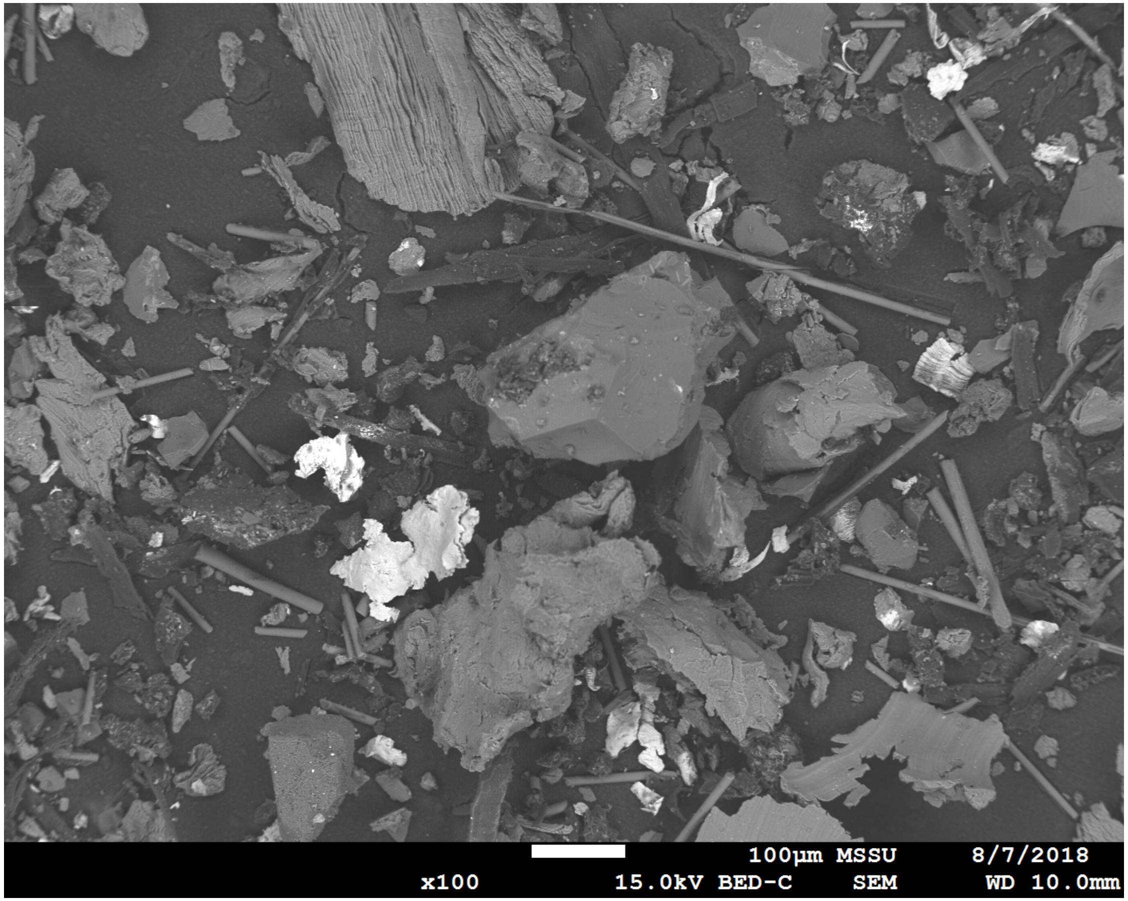

Fig. 4. Scanning electron micrograph of the extracted powder showing the different types and irregular size of the particulate matter. The bright areas indicate the presence of high atomic number elements and the dark areas indicate the presence of low atomic number elements.

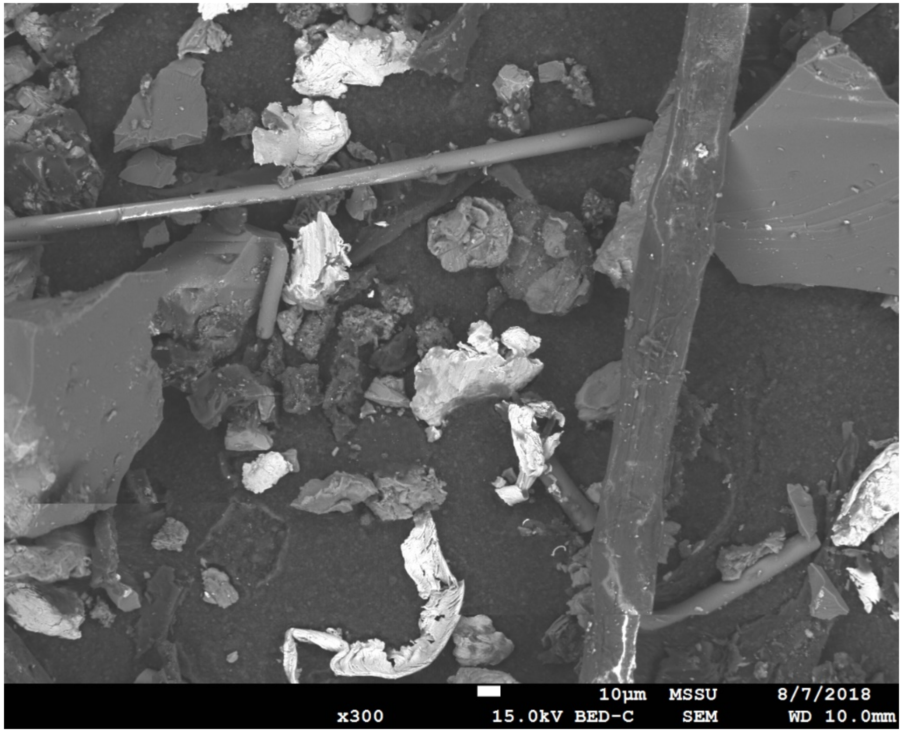

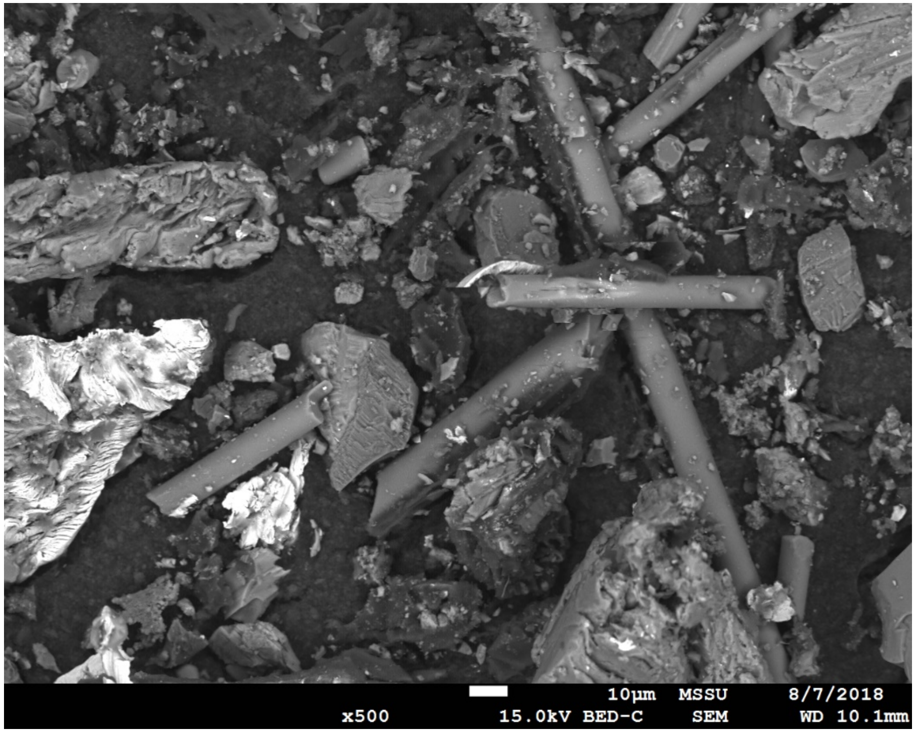

Fig. 5. Scanning electron micrographs of powder extracted from the partial incision of our metallic foam ceramic structure. The selected area corresponding to powder composition is consistent with the phase composition of a cutting disk provided by the manufacturer.

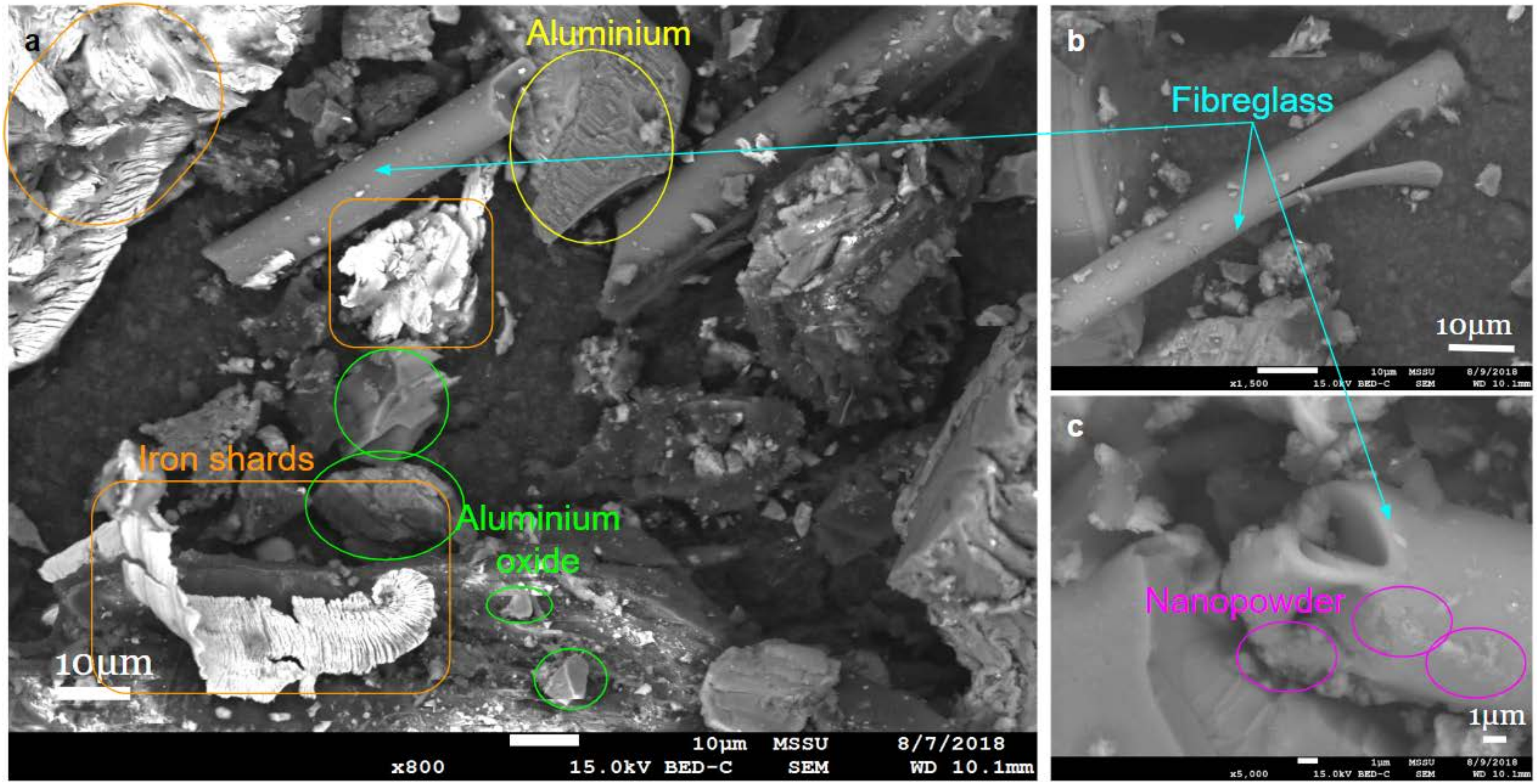

Fig.6. High magnification of SEM image highlights the existence of particulate matter across length scales ranging from nano through micro to mm.

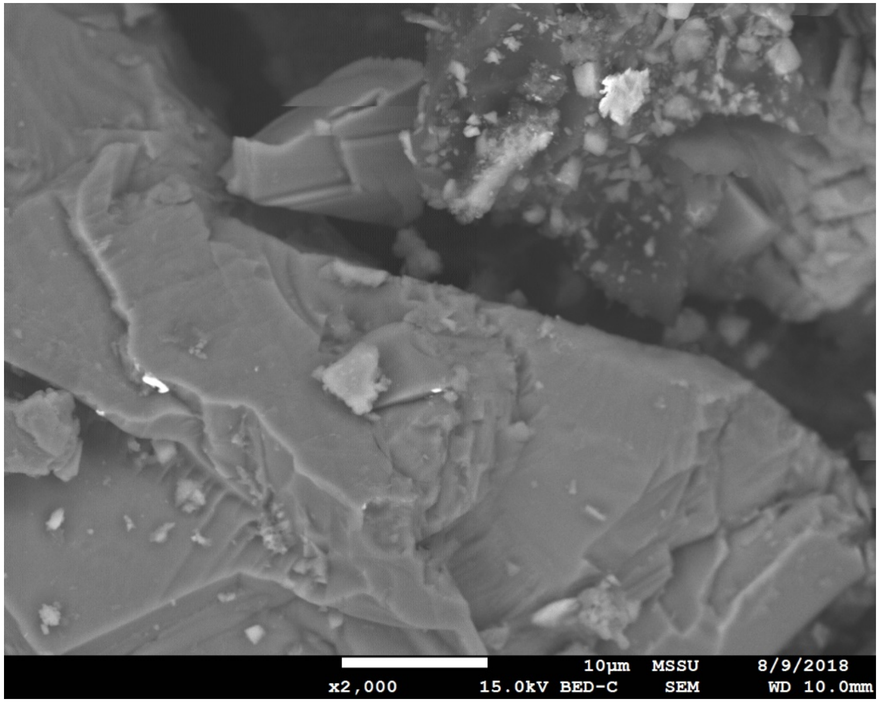

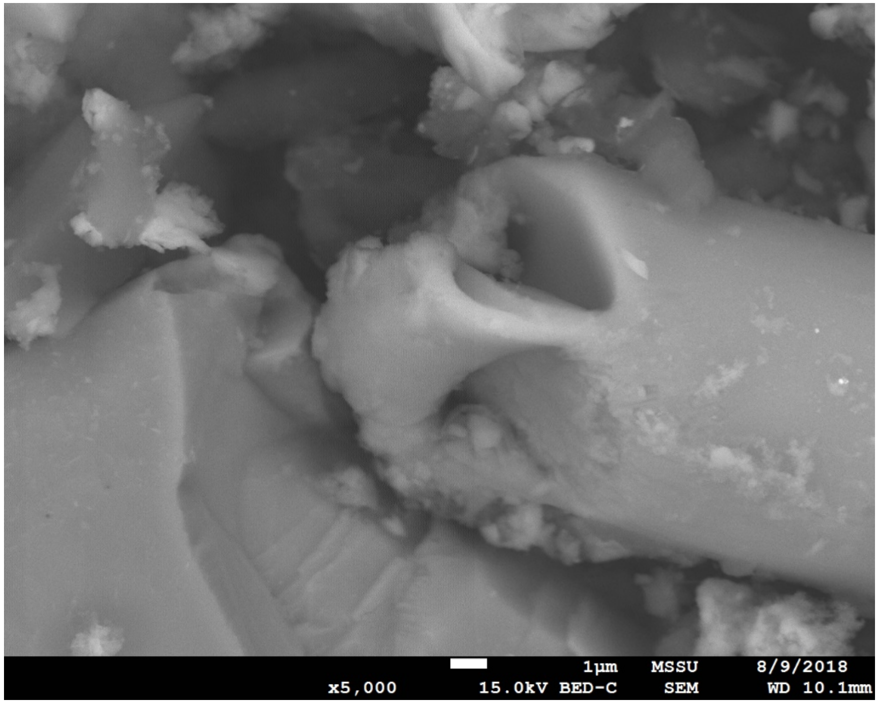

Fig. 7. Scanning electron micrograph of the selected area of extracted powder with a clear display of fiberglass fragment, individual particles as well as clusters of finer particles.

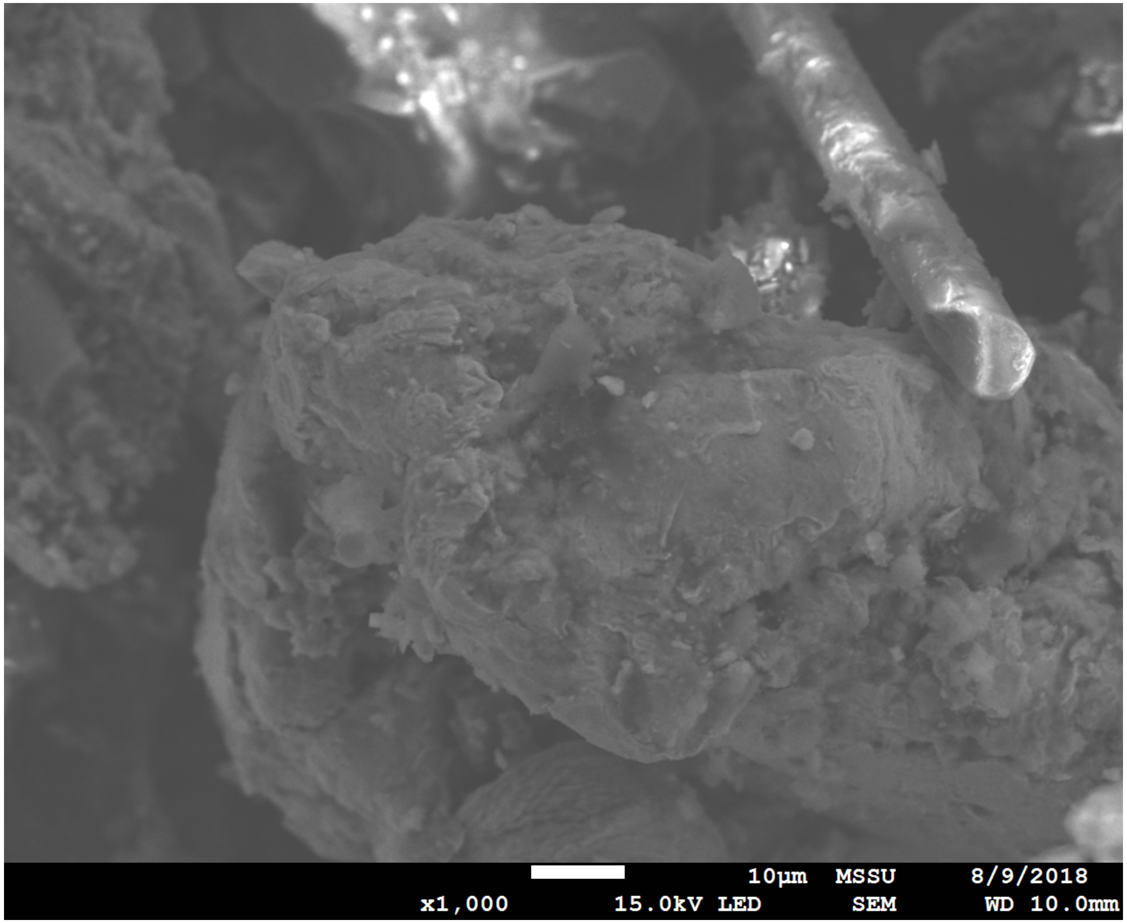

Supplement: Supplementary file 14 — Suppl_Information_C_SEM. [file 41598_2020_65976_MOESM14_ESM.pdf]
