# Supplementary material for: Non-cuttable material created through local resonance and strain rate effects
Source: Sci Rep. 2020 Jul 20;10:11539. doi: 10.1038/s41598-020-65976-0 (PMC7371712; doi:10.1038/s41598-020-65976-0)
Supplement: Supplementary file 15 — Supplementary information 15. [file 41598_2020_65976_MOESM15_ESM.docx]

# Supplementary Information

A) Set of images from CT-scans of the cylindrical sample,

B) Campbell, W. “Protection of Steam Turbine Disk Wheels from Axial Vibration.” ASME Transactions 46, 31–160 (1924),

C) Set of Scanning electron microscopy (SEM) micrographs.

# Supplementary Videos

[Movie 1. Angle Grinder cutting attack on our architected cellular metal-ceramic material.](https://www.dropbox.com/s/zyy010u1vu8xudd/Movie%2001.%20Angle%20Grinder%20cutting%20attack%20on%20our%20architected%20cellular%20metal-ceramic%20material.mp4?dl=0)

[Movie 2. Angle grinder cutting attack on armor steel plate.](https://www.dropbox.com/s/4zsdys0dctx3k2q/Movie%2002.%20Angle%20grinder%20cutting%20attack%20on%20armour%20steel%20plate.mp4?dl=0)

[Movie 3. Summary of CT scans of a cylindrical material sample subjected to angle grinder cutting tests.](https://www.dropbox.com/s/77446g4le8z7ocu/Movie%2003.%20Summary%20of%20CT%20scans%20of%20a%20cylindrical%20material%20sample%20subjected%20to%20angle%20grinder%20cutting%20tests.mp4?dl=0)

[Movie 4. CT sections across the angle grinder cuts.](https://www.dropbox.com/s/a2wrmjvg7edie0r/Movie%2004.%20CT%20sections%20across%20the%20angle%20grinder%20cuts.mp4?dl=0)

[Movie 5. CT scans into the angle grinder cuts.](https://www.dropbox.com/s/52zgtj83w9g8rl0/Movie%2005.%20CT%20scans%20into%20the%20angle%20grinder%20cuts.mp4?dl=0)

[Movie 6. CT scan of angle grinder attack applied to the center of the ceramic sphere.](https://www.dropbox.com/s/bk8k9hy1kktvx3i/Movie%2006.%20CT%20scan%20of%20angle%20grinder%20cut%20in%20the%20centre%20of%20the%20ceramic%20sphere.mp4?dl=0)

[Movie 7. CT scan of angle grinder cut in-between two ceramic spheres.](https://www.dropbox.com/s/pegbda2ac1ey5la/Movie%2007.%20CT%20scan%20of%20angle%20grinder%20cut%20in-between%20two%20ceramic%20spheres.mp4?dl=0)

[Movie 8. CT scan of angle grinder cut into a side of a ceramic sphere.](https://www.dropbox.com/s/84xng54xthp4ex3/Movie%2008.%20CT%20scan%20of%20angle%20grinder%20cut%20into%20a%20side%20of%20a%20ceramic%20sphere.mp4?dl=1)

[Movie 9. CT cross-sectional sweep from the top to the bottom of the cylindrical material sample.](https://www.dropbox.com/s/5z1a1b953onpkhi/Movie%2009.%20CT%20cross-sectional%20sweep%20from%20the%20top%20to%20the%20bottom%20of%20the%20cylindrical%20material%20sample.mp4?dl=1)

[Movie 10. Power Drill attack on our architected cellular metal-ceramic material.](https://www.dropbox.com/s/ru65tz0ebwj2hdq/Movie%2010.%20Power%20Drill%20attack%20on%20our%20architected%20cellular%20metal-ceramic%20material.mp4?dl=1)

[Movie 11. Modal shape of cutting discs free vibrations with a point constraint](https://www.dropbox.com/s/yw00vsd7ceoj0s0/Movie%2011.%20Mode%208%20-%2011.000rpm%20-%201mm.mp4?dl=1)
